# Pre-existing health conditions and severe COVID-19 outcomes: an umbrella review approach and meta-analysis of global evidence

**DOI:** 10.1186/s12916-021-02058-6

**Published:** 2021-08-27

**Authors:** Marina Treskova-Schwarzbach, Laura Haas, Sarah Reda, Antonia Pilic, Anna Borodova, Kasra Karimi, Judith Koch, Teresa Nygren, Stefan Scholz, Viktoria Schönfeld, Sabine Vygen-Bonnet, Ole Wichmann, Thomas Harder

**Affiliations:** Immunisation Unit, The Robert Koch Institute, Seestrasse 10, 13353 Berlin, Germany

**Keywords:** Umbrella review, Pre-existing health conditions, Comorbidities, COVID-19, SARS-CoV-2, Hospitalisation, Death

## Abstract

**Background:**

This study applies an umbrella review approach to summarise the global evidence on the risk of severe COVID-19 outcomes in patients with pre-existing health conditions.

**Methods:**

Systematic reviews (SRs) were identified in PubMed, Embase/Medline and seven pre-print servers until December 11, 2020. Due to the absence of age-adjusted risk effects stratified by geographical regions, a re-analysis of the evidence was conducted. Primary studies were extracted from SRs and evaluated for inclusion in the re-analysis. Studies were included if they reported risk estimates (odds ratio (OR), hazard ratio (HR), relative risk (RR)) for hospitalisation, intensive care unit admission, intubation or death. Estimated associations were extracted from the primary studies for reported pre-existing conditions. Meta-analyses were performed stratified for each outcome by regions of the World Health Organization. The evidence certainty was assessed using GRADE. Registration number CRD42020215846.

**Results:**

In total, 160 primary studies from 120 SRs contributed 464 estimates for 42 pre-existing conditions. Most studies were conducted in North America, European, and Western Pacific regions. Evidence from Africa, South/Latin America, and the Eastern Mediterranean region was scarce. No evidence was available from the South-East Asia region. Diabetes (HR range 1.2–2.0 (CI range 1.1–2.8)), obesity (OR range 1.5–1.75 (CI range 1.1–2.3)), heart failure (HR range 1.3–3.3 (CI range 0.9–8.2)), COPD (HR range 1.12–2.2 (CI range 1.1–3.2)) and dementia (HR range 1.4–7.7 (CI range 1.2–39.6)) were associated with fatal COVID-19 in different regions, although the estimates varied. Evidence from Europe and North America showed that liver cirrhosis (OR range 3.2–5.9 (CI range 0.9–27.7)) and active cancer (OR range 1.6–4.7 (CI range 0.5–14.9)) were also associated with increased risk of death. Association between HIV and undesirable COVID-19 outcomes showed regional heterogeneity, with an increased risk of death in Africa (HR 1.7 (CI 1.3–2.2)). GRADE certainty was moderate to high for most associations.

**Conclusion:**

Risk of undesirable COVID-19 health outcomes is consistently increased in certain patient subgroups across geographical regions, showing high variability in others. The results can be used to inform COVID-19 vaccine prioritisation or other intervention strategies.

**Supplementary Information:**

The online version contains supplementary material available at 10.1186/s12916-021-02058-6.

## Background

Early in 2020, the World Health Organization (WHO) declared the ongoing outbreak of Coronavirus Disease 2019 (COVID-19) to be a public health emergency of international concern [1]. Having spread around the world, the severe acute respiratory syndrome coronavirus 2 (SARS-CoV-2) causes rising numbers of COVID-19 cases and deaths. As of February 2021, the total number of confirmed cases has reached over 103 million, with over 2 million deaths worldwide [2]. With the ongoing pandemic, older people and people with chronic pre-existing conditions have been reported to be at higher risk of severe COVID-19 leading to hospitalisation, admission to intensive care, and death. Identified pre-existing conditions include hypertension, cardiovascular diseases, chronic kidney and liver diseases, cancer [3, 4], obesity, and immunosuppressed states [5, 6]. Around 22% of the global adult population is estimated to have at least one of the underlying health conditions, which can lead to severe COVID-19 if infected [7].

Since the start of the pandemic, multiple studies, which explore the association between pre-existing conditions and COVID-19 outcomes, have been conducted in different countries. Many literature reviews and meta-analyses further systematically examined the association for various health conditions. However, an overwhelming number of systematic reviews (SRs) with differences in outcomes definitions, selection criteria, synthesis and reporting complicate the interpretation of the overall body of evidence. It is unclear what pre-existing health conditions are associated with worse COVID-19 outcomes and whether the pre-existing conditions and the strength of associations differ between the geographical regions.

In this work, we aimed to summarise the evidence on age-adjusted associations between multiple pre-existing health conditions and clearly defined COVID-19 outcomes separating the effects between the geographical regions. To do it effectively and time-sparing, we conducted this study using an umbrella review approach.

Umbrella review is a systematic collection and assessment of evidence reported in systematic literature reviews. The methods of umbrella review allow for analysis of a large body of evidence on the strength of associations and the confidence in the estimates using the findings of SRs [8–12]. As previously discussed [13], umbrella reviews have been more frequently used to synthesise available evidence and inform clinical practice and public health policies. The methodology is valuable for compiling the findings for broad subject areas as it provides a uniform approach to evidence synthesis, increases statistical power and ensures confidence in the evidence. We applied the umbrella review methodology for this study because it allowed us to systematically and efficiently compile the published evidence on associations between a broad spectrum of pre-existing conditions and several COVID-19 outcomes across geographical regions into a single informative review.

## Methods

### Study design

We identified and analysed currently available SRs on pre-existing health conditions and risk of severe COVID-19, and the primary studies included therein using an umbrella review approach. The study protocol was registered in the International Prospective Register of Systematic Reviews (PROSPERO; registration no. CRD42020215846).

### Systematic reviews: search, selection, data extraction and quality assessment

We performed a systematic search for SRs in PubMed and Embase (including Medline), supplemented by hand searches on ArRvix, BioRvix, ChemRvix, MedRvix, Preprints.org, ResearchSquare and SSRN. All searches were done using the COVID-19 literature database constructed by the Robert Koch Institute library (date of the last search: 11 December 2020; search strings: Additional file 1 section 1.1).

To be eligible, an SR had to investigate the association between at least one pre-existing health condition (including but not limited to asthma, chronic obstructive pulmonary disease (COPD), cancer, diabetes mellitus, cardiovascular diseases, chronic kidney diseases, chronic liver diseases, chronic diseases of the digestive system, hypertension, obesity and immunocompromising conditions), and at least one of the following severe health outcomes due to COVID-19: hospitalisation, admission to ICU, ventilation (intubation) or death (Additional file 1: section 1.2). The titles and abstracts of identified SRs were screened independently by two investigators (MTS, SR). Data extraction (Additional file 1: section 1.3) and quality assessment were divided between the reviewers (AB, KK, SR, LH, AP, MTS) following the four-eyes principle, meaning that one reviewer performed the task and a second reviewer confirmed the results. All disagreements were resolved by discussion or by a third reviewer (TH). The methodological quality of included systematic reviews was assessed using the AMSTAR-2 (Assessment of Multiple Systematic Reviews-2) tool (Additional file 1 section 1.4) [14].

### Primary studies: search, selection, data extraction and risk of bias assessment

The analysed systematic reviews did not provide evidence on age-adjusted risk estimates across geographical regions. Therefore, we evaluated the primary studies included in SRs for a re-analysis. Primary studies reported in the included SRs were included in the re-analysis if they (i) reported at least one quantitative measure of association (risk ratio (RR), odds ratio (OR), or hazard ratio (HR)) in patients with a pre-existing health condition, compared to patients without this condition; (ii) reported age-adjusted estimates (multivariate models, cohort matching or age stratification) for at least one pre-existing condition and at least one health outcome (hospitalisation, admission to ICU, mechanical ventilation, or intubation, in-hospital mortality, mortality among SARS-CoV2-positively tested persons (case mortality)). Primary studies of all designs were eligible. Study selection and data extraction were performed as for SRs (Additional file 1: section 1.5). To be consistent, when several models were reported, we chose those that adjusted for age, sex and pre-existing conditions and avoided (if possible) adjustment for laboratory values, vital signs or socio-economic factors. For evaluation of the risk of bias in the primary studies, the results of the assessments performed by the authors of the respective SRs that reported the primary studies were used. In case a primary study was not evaluated in any included SR, we assessed the risk of bias using the Newcastle-Ottawa Scale (NOS) [15].

### Data analysis

If more than one estimate for an association between a pre-existing health condition and an outcome was reported, we used *I*^2^ (%) statistics to test whether a meta-analysis was appropriate to combine the estimates. If *I*^2^ was ≤ 40%, we decided that heterogeneity was low enough to allow a meta-analysis. Random-effects models (generic inverse variance method) were used to combine estimates by health outcome, the measure of association, and risk factor in R using the *metaphor* package, which allows the application of generic inverse variance methods for adjusted risk estimates for which confidence interval, but no standard error is reported. To consider the possible influence of setting-specific factors (geographical, healthcare and resources), we stratified meta-analyses by WHO regions. Countries were grouped into the WHO regions as follows. South Africa—the African Region (AFR). Kuwait and Iran—the Eastern Mediterranean Region (EMR). Denmark, France, Italy, Israel, Spain, Switzerland and the UK—the European Region (EUR). Republic of Korea, China and the Philippines—the Western Pacific Region (WPR). Regions of America (AMR) were divided into North America (the USA), and South/Latin America (Brazil, Mexico, Bolivia). There were no included studies from countries of the South-East Asia Region (SEAR). If *I*^2^ was > 40%, ranges (min–max) of the estimates were reported instead of the pooled results. If ten or more estimates for a given association and outcome were available, the likelihood of publication bias was assessed by inspection of funnel plots, followed by Egger’s test and Begg’s test. Due to the differences in methods of association estimation, the measures of associations were treated separately as odds ratio (OR), hazard ratio (HR) and risk ratio (RR).

### Analysis of subgroups

We conducted analyses of subgroups for those primary studies that used age stratification to control the confounding effect of age and the studies that selected a specific study population, e.g. patients with cancer. Meta-analyses were not performed here because of the limited number of studies and high heterogeneity in reporting. Instead, the evidence was narratively synthesised and presented.

### Certainty of the evidence (evidence quality)

Certainty of the evidence was assessed for each outcome using the methodology proposed by the Grading of Recommendations Assessment, Development and Evaluation (GRADE) Working Group for risk factor studies [16], as described in PRECEPT (Project on a Framework for Rating Evidence in Public Health) [17]. In data extraction, we looked at the covariates used in the multivariate regression models of the included studies. Observed variations in the number and nature of the included factors suggested different residual confounding of the resulted risk estimates. In the certainty assessment, for each estimate, we evaluated inconsistency, imprecision and bias. Downgrading factors included serious inconsistency defined as *I*^2^ > 40% heterogeneity, serious imprecision defined as confidence interval including 1.0, serious risk of bias judged based on the quality assessment extracted from SRs and risk of publication bias. For the estimates obtained from single studies, inconsistency was not graded.

## Results

### Characteristics of included systematic reviews

The systematic search identified a total of 3417 entries. After title/abstract and full-text screening, 120 reviews [3–6, 18–133] were found to be eligible (Additional file 1: Figure 1, Table 1). The majority of SRs explored a variety of risk factors for severe COVID-19 outcomes, including pre-existing conditions, vital signs and socio-economic factors. Other reviews focused on a specific pre-existing health condition such as obesity, diabetes, cancer or hypertension. The number of included primary studies varied between 3 and 212 (Additional file 1: Figure 3A). Only three reviews conducted literature searches up to September and October 2020, while the majority included data from primary studies published up to 31 May 2020 (Additional file 1: Table 1 and Figure 3B). The methodological quality of reviews was low to critically low, mostly due to the lack of a registered protocol and reporting on excluded studies or language restrictions (Additional file 1: Table 2 and Figure 3C).

### Characteristics of included primary studies

Based on included SRs, a total of 356 primary studies were assessed for eligibility. After the exclusion of 196 papers, 160 remained for data extraction [134–293] (Additional file 1: Figure 2). Of those, 59 studies were single-centre studies, 77 studies followed a multi-centre approach, while the remaining studies came from diverse settings (e.g. used electronic databases) (Additional file 1: Table 3). All but 15 studies had a cohort design. The number of participants in the primary studies varied between 36 and 89,756. To minimise methodological heterogeneity, we excluded nine [294–302] studies from the main analysis since they reported community-based estimates only (Additional file 1: section 2.2, Table 4). For each study, we provided information regarding the COVID-19 case definition (Additional file 1: Table 3). In over 90% of the articles polymerase chain reaction (PCR) and/or viral sequencing was used. In 5% of the studies, PCR was used in at least 82% of the included cases or PCR was used alongside with / or was substituted by a blood test for SARS-Cov-2 IgG/IgM for antibodies or radiologic findings. In 2.5% of the studies, ICD-10 codes or a mix of PCR, clinical diagnosis or ICD-10 codes were used; however, the proportion of each method was not stated. Only in 1.8% of the articles, the authors did not explicitly state how COVID-19 cases were diagnosed. We decided to include the latter studies to obtain a complete overview over analyses conducted regarding our research question.

Additionally, the definitions of the pre-existing studies were extracted from the studies which reported them (Additional file 1: Table 3 and section 2.3). In the meta-analyses, we did not conduct additional grouping into broader disease categories to avoid increased heterogeneity of the results stemming from the differences in the disease definitions. Instead, we addressed each health condition as it was reported in the primary studies. Similar descriptions of the diseases were grouped as described in the Additional file 1 (section 2.3).

### Data analyses

We extracted 1321 estimates for risk factors indicating pre-existing health conditions. Risk estimates that used comorbidity indexes (e.g. Charlson score) and age-stratified estimates were excluded from the meta-analyses (see below for separate reporting of age-stratified estimates). In total, 1019 extracted estimates for 54 pre-existing health conditions from 133 [134–143, 145–150, 152–170, 172–177, 180–182, 184–196, 198–200, 202–205, 207, 210–213, 216, 218–221, 225–230, 233–235, 237–242, 244–246, 248, 249, 251–281, 283, 284, 287–292] primary studies were included in the meta-analysis. The meta-analysis resulted in 521 estimates, of which 464 estimates for 42 pre-existing conditions for the primary outcomes were selected for further analysis, GRADE assessment and reporting (Table 1, excluded are listed in Additional file 1: section 2.4). For 281 associations, only single-study estimates were available. In total, 111 associations are reported as pooled estimates from at least two studies with low between-study heterogeneity. For 72 associations, heterogeneity was considerable (*I*^2^ > 40%). For the latter, the risk estimates were illustrated as ranges (min–max) (Additional file 1: section 2.5, Figures 4-6), and both the pooled estimates and the ranges are reported. All estimates are reported in Additional file 1 (section 2.5, Tables 5.1-9.7). The risk of bias was low in the majority of studies (Additional file 1: Table 3). Potential publication bias was detected only in one meta-analysis (risk of intubation for people with obesity) (Additional file 1: Table 9.4). Certainty of the evidence was high for 179 of the estimates, moderate for 234 and low for 51. None of the analysed bodies of evidence was assessed to have very low certainty. Studies that selected populations based on a pre-existing condition were summarised in Additional file 1 (section 2.8).

**Table 1:** Characteristics of analysed primary studies per pre-existing condition

Disease group	Pre-existing condition	Outcomes included	Number of systematic reviews reporting primary studies	Number of resulted estimates	Number of primary studies	Countries	Sample sizes (min – max)	GRADE confidence in estimate (min – max)
Circulatory diseases	Arrhythmia	Hospitalisation; case mortality; hospital mortality; intubation	13 [35, 39, 42, 54, 57, 70, 78, 90, 96, 97, 114, 124, 133]	9	7 [198, 210, 233, 240, 245, 262, 272]	Italy; UK; Denmark; Spain; USA; China	322–11,122	moderate - high
Circulatory diseases	Cardiovascular disease	Hospital mortality; hospitalisation; case mortality; intubation; ICU admission	57 [3, 4, 6, 18, 19, 23, 24, 26–29, 31, 34, 35, 37–42, 44, 46, 48, 53, 54, 57–60, 64, 66, 68–70, 74, 76, 78, 88, 90–92, 95–97, 103, 105, 111, 114, 116, 117, 120, 122, 124, 126, 127, 132, 133]	21	32 [135, 139, 140, 156, 157, 159, 160, 175, 182, 187, 190, 196, 199, 203, 216, 218, 220, 228, 235, 238, 241, 242, 245, 255, 256, 262, 264–266, 274, 276, 290]	Iran; France; Spain; Italy; Israel; USA; Brazil; Mexico; China; Korea	103–89,756	low - high
Circulatory diseases	Coronary artery disease	Hospital mortality; hospitalisation; case mortality; ICU admission; intubation	70 [4, 5, 18, 20, 21, 24, 25, 27–29, 31, 34, 35, 38–42, 47, 48, 50–52, 54, 55, 57–60, 63–66, 68, 70, 74, 77, 78, 80, 81, 83–85, 87–91, 94–97, 102–105, 111, 114, 116, 117, 120, 122, 124–126, 129–133]	17	26 [63, 138, 153, 157, 158, 161, 163, 176, 186, 193, 198, 205, 207, 210, 213, 230, 233, 234, 240, 245, 252, 267, 272, 283, 290, 291]	UK; Italy; Europe; Denmark; USA; China; Korea	112–11,210	low - high
Circulatory diseases	Heart disease	Hospital mortality; hospitalisation; case mortality; intubation; ICU admission	17 [21, 24, 31, 37, 38, 42, 54, 57, 64, 74, 78, 81, 93, 104, 114, 120, 122]	7	6 [149, 170, 192, 200, 254, 275]	Spain; UK; USA; China	103–15,194	low - high
Circulatory diseases	Heart failure	Hospital mortality; hospitalisation; case mortality; intubation; ICU admission	29 [4, 5, 21, 25, 28, 29, 35, 37–39, 42, 54, 57, 58, 64, 68, 70, 78, 81, 90, 96, 97, 111, 114, 117, 120, 124, 126, 133]	15	22 [138, 139, 162, 169, 173, 177, 191, 198, 205, 213, 230, 233, 234, 240, 245, 248, 251, 252, 267, 271, 272, 283]	Italy; UK; Denmark; USA; China	191–31,461	low - high
Circulatory diseases	Hypertension	ICU admission; hospital mortality; hospitalisation; case mortality; intubation	71 [3–6, 18, 19, 21, 23–31, 33–35, 37–42, 44, 46, 48, 50, 53–55, 57–59, 62–66, 68–70, 72, 74, 78, 81, 88, 90–93, 95–97, 99, 103–105, 107, 111, 114, 117, 120, 122–124, 127, 128, 132, 133]	37	71 [134, 137–139, 143, 145, 148, 149, 152, 153, 155, 156, 158–160, 163, 164, 167–169, 173–177, 182, 192–194, 198–200, 203, 205, 207, 210, 213, 216, 219–221, 226–228, 233–235, 239–242, 245, 246, 248, 251, 252, 255, 258, 262, 265, 267, 268, 271, 272, 274, 275, 278, 281, 284, 287, 290]	Kuwait; Spain; UK; Italy; Europe; Denmark; Israel; Switzerland; France; USA; Mexico; Bolivia; China; Korea; South Africa	103–89,756	low - high
Circulatory diseases	Infarction	Hospital mortality; case mortality	4 [37, 57, 68, 97]	4	4 [169, 177, 191, 271]	Italy; USA; China	2877 – 31,461	moderate - high
Circulatory diseases	Peripheral vascular disease	Hospital mortality; hospitalisation; intubation; case mortality	4 [37, 54, 97, 133]	5	3 [191, 271, 272]	USA	2015 – 31,461	moderate - moderate
Circulatory diseases	Venous thromboembolism	Hospital mortality; hospitalisation; intubation	9 [29, 39, 54, 70, 78, 97, 114, 120, 133]	4	3 [233, 235, 272]	UK; USA	238–3703	low - moderate
Immunodeficiency	Autoimmune condition	Hospital mortality; hospitalisation; case mortality	4 [54, 78, 90, 97]	7	4 [149, 187, 228, 262]	Spain; USA	322–9437	moderate - high
Immunodeficiency	HIV	Hospital mortality; ICU admission; case mortality; intubation	17 [5, 28, 35, 37–39, 42, 62, 64, 70, 90, 96, 97, 111, 114, 120, 124]	10	8 [138, 148, 180, 191, 199, 229, 233, 257]	UK; USA; South Africa	251–47,539	low - high
Immunodeficiency	Inflammatory bowel disease	Hospital mortality; intubation; hospitalisation	14 [5, 28, 35, 38, 39, 42, 90, 92, 96, 97, 111, 114, 120, 124]	3	2 [138, 261]	USA	464–841	moderate - moderate
Immunodeficiency	Immunosuppression	Hospital mortality; intubation; hospitalisation; ICU admission; case mortality	19 [5, 28, 29, 35, 38, 39, 42, 44, 57, 64, 68, 78, 90, 95–97, 111, 120, 124]	12	12 [138, 140, 142, 147, 152, 158, 182, 203, 226, 252, 265, 271]	Italy; USA; Brazil; Mexico	302–89,756	moderate - high
Immunodeficiency	Organ transplant recipients	Hospital mortality; hospitalisation; case mortality; intubation	13 [5, 28, 35, 38, 39, 42, 78, 90, 96, 97, 111, 120, 124]	6	3 [138, 240, 271]	Denmark; USA	841–11,122	moderate - high
Immunodeficiency	Rheumatological disease	Hospital mortality; hospitalisation; case mortality; intubation	15 [5, 28, 35, 37–39, 42, 78, 90, 96, 97, 111, 114, 120, 124]	8	5 [138, 166, 191, 203, 240]	Denmark; USA	156–31,461	moderate - high
Liver & Metabolic diseases	Chronic kidney disease	Hospital mortality; hospitalisation; case mortality; ICU admission; intubation	37 [4–6, 19, 21, 25, 28, 29, 35, 37–39, 42, 44, 46, 54, 55, 57, 58, 62, 64, 68, 70, 78, 81, 90, 95–97, 104, 111, 114, 117, 120, 124, 126, 133]	30	55 [137, 138, 140, 142, 148, 149, 152, 153, 156, 159, 160, 162, 163, 169, 170, 176, 177, 182, 186, 187, 190, 191, 193, 198, 199, 203, 205, 207, 218, 219, 226, 228, 230, 233–235, 240–242, 245, 246, 248, 251, 252, 256, 262, 264–267, 271–273, 283, 290]	Spain; UK; Italy; Europe; Denmark; USA; Brazil; Mexico; China; Korea; South Africa	112–89,756	low - high
Liver & Metabolic diseases	Chronic liver disease	Hospital mortality; hospitalisation; case mortality; intubation; ICU admission	16 [21, 24, 25, 37, 42, 57, 64, 78, 81, 90, 97, 104, 114, 117, 120, 124]	15	13 [140, 170, 187, 191, 192, 199, 228, 240, 251, 252, 260, 262, 290]	UK; Denmark; Spain; USA; Brazil; China	322–31,461	low - high
Liver & Metabolic diseases	Chronic liver disease/Cirrhosis	Hospital mortality; intubation	17 [5, 28, 35, 38, 39, 42, 57, 64, 70, 90, 96, 97, 111, 114, 120, 124, 133]	6	5 [138, 143, 192, 233, 248]	Spain; UK; USA	242–4035	moderate - high
Liver & Metabolic diseases	Chronic liver disease /Non-cirrhotic	Hospital mortality	4 [70, 97, 114, 120]	2	2 [192, 233]	UK; USA	363–614	moderate - moderate
Liver & Metabolic diseases	Diabetes	ICU admission; hospital mortality; hospitalisation; case mortality; intubation	69 [3–6, 18, 19, 21, 23–31, 33–35, 37–42, 44, 46, 48, 50, 53–55, 57–59, 62–64, 68–70, 72, 78, 81, 88, 90, 92, 93, 95–97, 99, 103–105, 107, 111, 114, 117, 120, 123, 124, 126–128, 132, 133]	38	80 [134, 135, 138–140, 142, 145, 148–150, 152, 153, 158–160, 163, 164, 167–170, 173, 175, 176, 182, 187, 191–193, 195, 196, 198–200, 202–205, 207, 210, 216, 218–221, 225–228, 230, 233–235, 238, 240–242, 245, 246, 248, 251, 252, 255, 256, 258, 262–267, 271, 272, 280, 283, 284, 289, 290, 292]	Kuwait; Iran; France; Spain; UK; Italy; Europe; Denmark; Israel; USA; Brazil; Mexico; China; Korea; South Africa	92–89,756	low - high
Liver & Metabolic diseases	Dyslipidemia or hyperlipidemia	Hospital mortality; hospitalisation; case mortality; intubation; ICU admission	32 [4–6, 21, 25, 27–29, 31, 33, 35, 39, 42, 54, 63, 64, 68, 70, 78, 81, 90, 93, 96, 97, 99, 111, 114, 120, 124, 126, 132, 133]	12	12 [149, 192, 205, 218, 230, 234, 235, 245, 258, 262, 263, 271]	Spain; Italy; France; USA	124–9437	low - high
Liver & Metabolic diseases	Hepatitis	Hospital mortality; intubation	13 [5, 28, 35, 38, 39, 42, 55, 90, 96, 97, 111, 120, 124]	3	2 [138, 219]	USA; China	841–2665	moderate - moderate
Neurological diseases & Mental health	Cerebrovascular/Stroke	Hospital mortality; ICU admission; hospitalisation; case mortality; intubation	44 [4–6, 19, 24, 27–29, 31, 34, 35, 37–42, 44, 48, 54, 55, 57, 58, 60, 64, 66, 68, 70, 74, 76, 78, 88, 90, 91, 95–97, 105, 111, 114, 116, 117, 120, 124, 133]	17	20 [138, 145, 155, 156, 186, 191, 196, 207, 210, 213, 219, 233, 235, 240, 246, 256, 262, 271, 272, 276]	Italy; UK; Denmark; Spain; USA; China; Korea	103–31,461	low - high
Neurological diseases & Mental health	Dementia	Hospital mortality; hospitalisation; case mortality; intubation	22 [21, 29, 35, 37, 39, 42, 54, 57, 64, 68, 70, 78, 81, 90, 96, 97, 104, 114, 117, 120, 124, 133]	12	14 [143, 146, 149, 150, 170, 191, 196, 199, 220, 233, 240, 245, 262, 272]	Spain; UK; Italy; Denmark; Israel; USA; Korea	92–31,461	low - high
Neurological diseases & Mental health	Depression	Hospitalisation	4 [78, 90, 97, 114]	2	3 [139, 220, 262]	Israel; Spain; USA	322–1052	low - moderate
Neurological diseases & Mental health	Neurological disease	Hospital mortality; ICU admission; case mortality	15 [21, 37, 39, 42, 57, 64, 68, 78, 81, 97, 104, 114, 120, 126, 133]	5	5 [140, 143, 170, 203, 266]	Spain; UK; USA; Brazil	2070–15,194	moderate - high
Neurological diseases & Mental health	Psychiatric disorder	Hospital mortality; hospitalisation; case mortality	2 [78, 124]	3	2 [220, 240]	Denmark; Israel	782–11,122	high - high
Oncological diseases	Cancer	Hospital mortality; hospitalisation; case mortality; intubation; ICU admission	38 [4–6, 19, 21, 24, 28–30, 35, 37, 39, 42, 44, 54, 55, 57, 58, 64, 68, 70, 72, 78, 81, 90, 96, 97, 104, 107, 111, 114, 117, 120, 123, 124, 126, 128, 133]	19	30 [139, 149, 156, 167, 169, 170, 187, 191, 198, 199, 205, 207, 210, 211, 218, 219, 221, 225, 228, 234, 235, 240, 245, 252, 256, 262, 267, 271, 272, 283, 290]	Spain; Italy; UK; Denmark; USA; China; Korea	238–31,461	low - high
Oncological diseases	Cancer/Active	Hospital mortality; ICU admission; intubation	18 [5, 25, 28, 35, 38, 39, 42, 57, 64, 78, 90, 96, 97, 104, 111, 120, 124, 133]	8	6 [138, 141, 143, 161, 176, 251]	Spain; Italy; UK; USA	407–4035	moderate - high
Oncological diseases	Cancer/ Haematological	Hospital mortality	5 [70, 97, 108, 114, 123]	2	3 [233, 249, 253]	UK; Spain	92–1183	low - high
Oncological diseases	Cancer/Solid	Hospital mortality; case mortality	4 [37, 70, 97, 114]	2	2 [191, 233]	UK; USA	614–31,461	moderate - high
Overweight, obesity, underweight	Obesity/BMI > 30	ICU admission; hospital mortality; hospitalisation; case mortality; intubation	39 [4–6, 21, 25, 27–29, 31, 33, 35, 37–39, 42, 44, 54, 57, 63, 64, 68, 70, 78, 81, 90, 93, 95–97, 99, 104, 111, 114, 117, 120, 124, 126, 132, 133]	27	53 [134, 137, 140–143, 149, 152, 154, 158, 163, 164, 168–170, 173, 181, 182, 184, 187, 192, 195, 198–200, 202, 203, 205, 213, 216, 218, 220, 226–228, 230, 234, 235, 237, 240, 245, 246, 251, 252, 258, 259, 262, 264–266, 271, 272, 283]	Kuwait; Spain; Italy; UK; France; Denmark; Israel; USA; Brazil; Mexico	103–89,756	low - high
Overweight, obesity, underweight	Obesity/BMI > 40	ICU admission; hospital mortality; hospitalisation; intubation; case mortality	19 [5, 21, 28, 29, 35, 39, 57, 58, 64, 68, 78, 81, 96, 97, 111, 114, 120, 124, 126]	7	8 [134, 184, 195, 205, 234, 252, 267, 271]	Kuwait; USA	463–6916	high - high
Overweight, obesity, underweight	Overweight	ICU admission; intubation; hospital mortality; hospitalisation; case mortality	25 [5, 6, 21, 25, 27, 29, 31, 33, 35, 39, 42, 54, 63, 78, 81, 93, 96, 97, 99, 114, 120, 124, 126, 132, 133]	11	11 [134, 184, 187, 200, 227, 228, 234, 251, 258, 271, 272]	Kuwait; France; USA	103–6916	low - high
Overweight, obesity, underweight	Underweight	Hospital mortality; hospitalisation; intubation; case mortality	12 [4, 21, 25, 28, 29, 37, 70, 78, 81, 97, 114, 120]	7	6 [184, 187, 199, 228, 230, 271]	USA	200–6916	low - moderate
Respiratory diseases	Asthma	Hospital mortality; hospitalisation; intubation; case mortality	19 [19, 29, 39, 42–44, 54, 57, 64, 70, 78, 90, 95, 97, 111, 114, 120, 126, 133]	12	16 [139, 140, 158, 182, 212, 218, 226, 228, 233, 252, 262, 265, 271–273, 283]	UK; Spain; USA; Brazil; Mexico	322–89,756	low - high
Respiratory diseases	COPD	Hospital mortality; hospitalisation; case mortality; intubation; ICU admission	54 [4, 6, 19, 21, 24, 25, 27–31, 34, 35, 38–42, 44, 46, 48, 54, 55, 57, 58, 60, 62, 64, 66, 68, 70, 72, 74, 76, 78, 81, 88, 90, 91, 95–97, 105, 107, 111, 114, 116, 117, 120, 123, 124, 126, 128, 133]	21	34 [137, 139, 142, 148, 149, 152, 156, 158, 160, 167, 182, 186, 207, 213, 218, 219, 226, 228, 230, 233, 239, 241, 245, 252, 256, 257, 262, 263, 265, 272, 276, 283, 288, 290]	Spain; Italy; UK; Switzerland; USA; Mexico; China; Korea	145–89,756	low - high
Respiratory diseases	COPD or Asthma	Hospital mortality; hospitalisation; intubation; ICU admission	17 [5, 21, 25, 29, 35, 38, 39, 64, 78, 81, 96, 97, 111, 114, 120, 124, 126]	5	5 [173, 198, 234, 248, 251]	USA	214–5279	moderate - moderate
Respiratory diseases	Interstitial lung disease	Hospital mortality	1 [97]	1	1 [228]	USA	5776	high - high
Respiratory diseases	Obstructive sleep apnea	Intubation; hospitalisation	11 [4, 21, 25, 28, 29, 70, 78, 81, 95, 111, 114]	2	2 [158, 230]	USA	200–1526	moderate - high
Respiratory diseases	Respiratory disease	Hospital mortality; ICU admission; hospitalisation; case mortality; intubation	33 [4, 5, 21, 28, 29, 31, 35, 37–39, 41, 42, 54, 57, 62, 64, 66, 68, 78, 81, 90, 93, 96, 97, 104, 111, 114, 117, 120, 124, 126, 133]	24	26 [138, 140, 142, 145, 148, 150, 169, 170, 176, 187, 191, 192, 196, 199, 200, 203, 216, 220, 235, 240, 242, 255, 264, 266, 268, 271]	Italy; UK; Spain; Denmark; Israel; USA; Brazil; Mexico; China; Korea; South Africa	92–51,633	low - high
Respiratory diseases	Tuberculosis	Hospital mortality; hospitalisation; case mortality	4 [55, 62, 68, 100]	6	3 [148, 219, 269]	China; Philippines; South Africa	330–22,308	moderate - high

*No* number, *ICU* intensive care unit

### Risk estimates

**Fig. 1:**
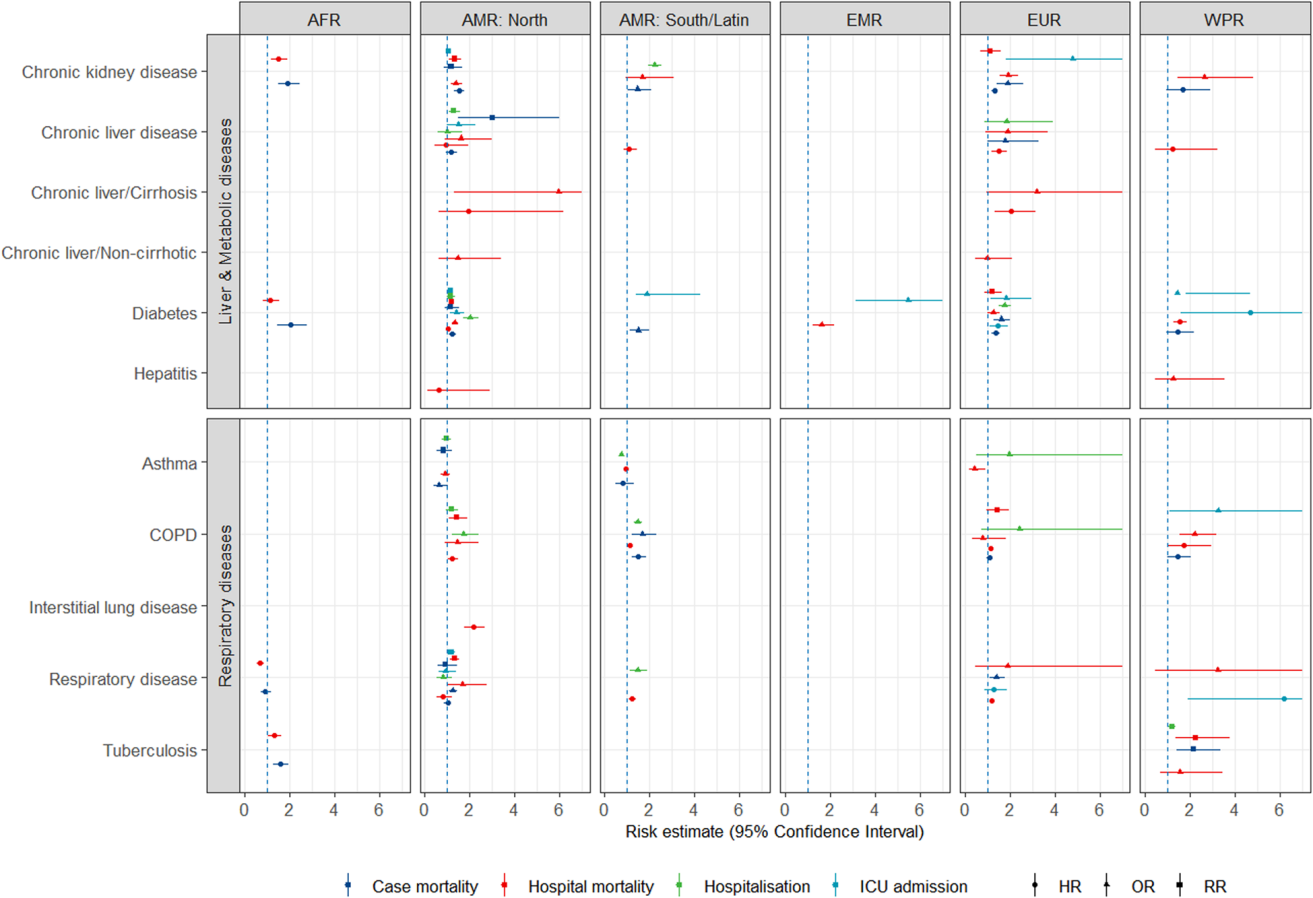
Results of the meta-analyses for pre-existing conditions: Liver and metabolic diseases (the upper panel) and respiratory diseases (the lower panel), by WHO region (excluding SEAR). The error bars represent 95% confidence intervals. The dashed line indicates 1.0 value. The estimates with error bars crossing the 1.0-line lack statistical significance

**Fig. 2:**
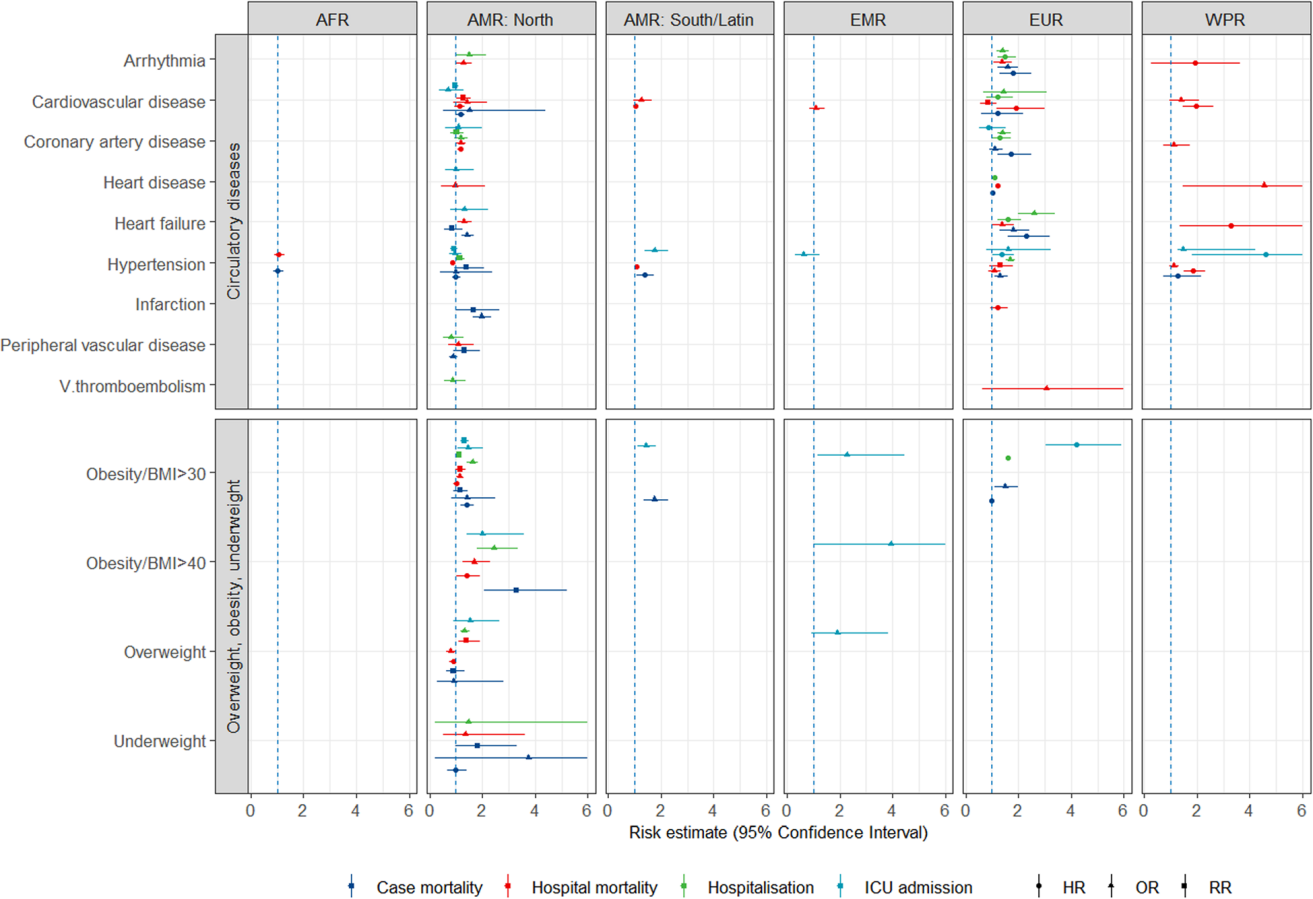
Results of the meta-analyses for pre-existing conditions: Circulatory diseases (the upper panel) and overweight/obesity/underweight (the lower panel), by WHO region (excluding SEAR). The error bars represent 95% confidence intervals. The dashed line indicates 1.0 value. The estimates with error bars crossing the 1.0-line lack statistical significance

**Fig. 3:**
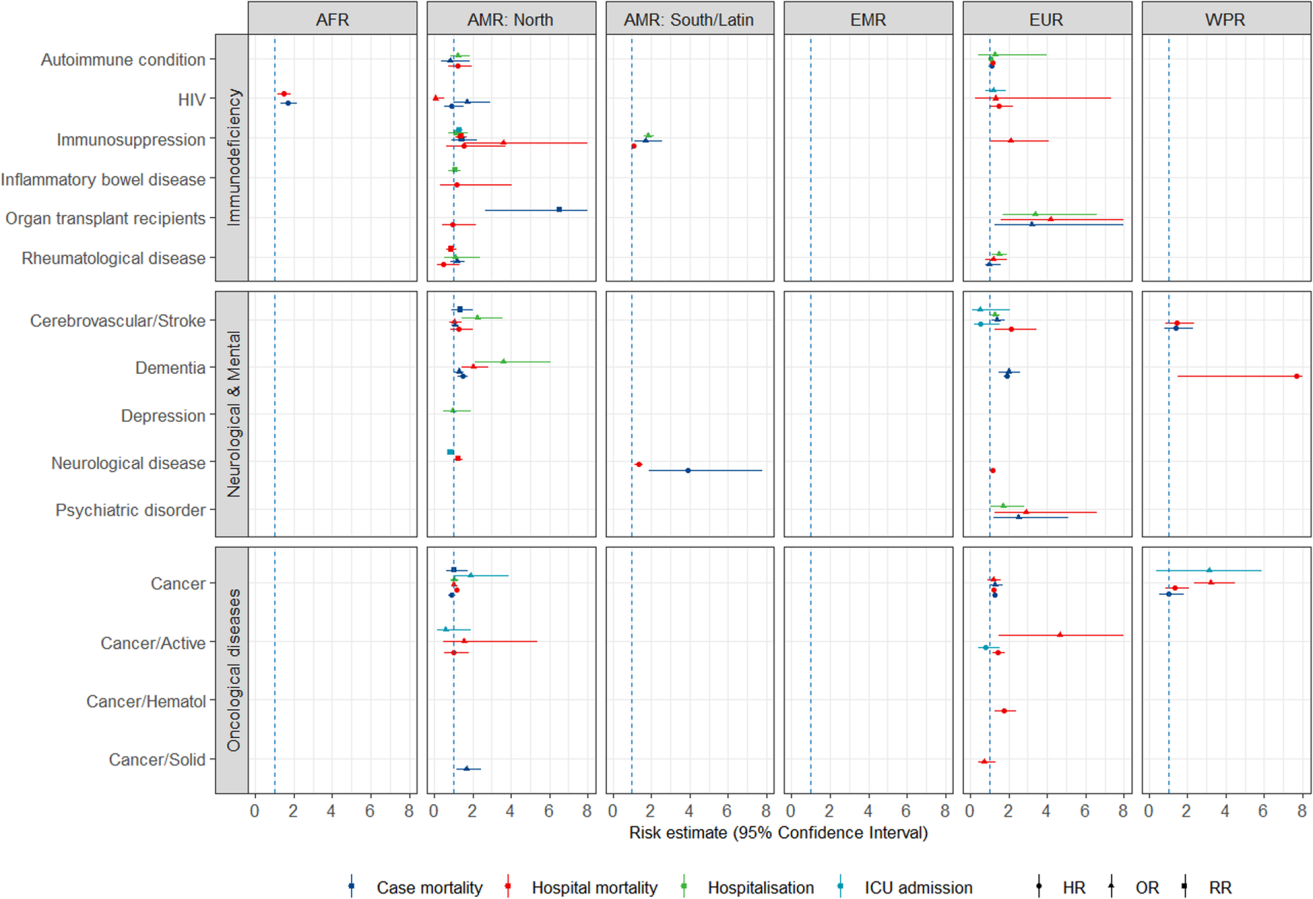
Results of the meta-analyses for pre-existing conditions: Immunodeficiency (the upper panel), neurological diseases and mental health (the middle panel) and oncological diseases (lower panel), by WHO region (excluding SEAR). The error bars represent 95% confidence intervals. The dashed line indicates 1.0 value. The estimates with error bars crossing the 1.0-line lack statistical significance

**Fig. 4:**
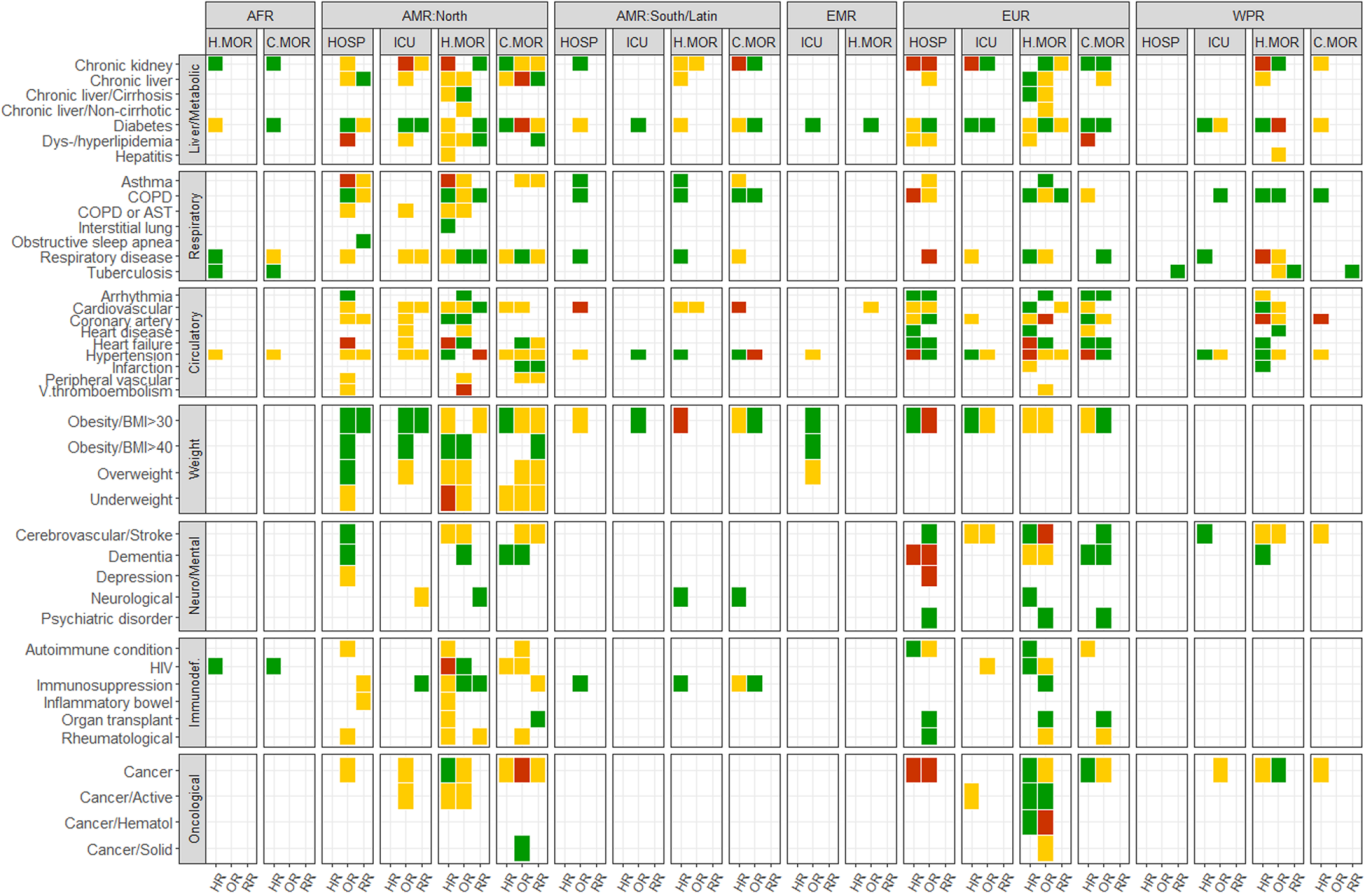
Summary of the GRADE assessment for each pre-existing condition, by health outcome and WHO region (red color - low,yellow color - moderate, green color - high). Outcomes include hospitalisation (HOSP), intensive care unit (ICU), in-hospital mortality (H.MOR), case mortality (C.MOR)

**Fig. 5:**
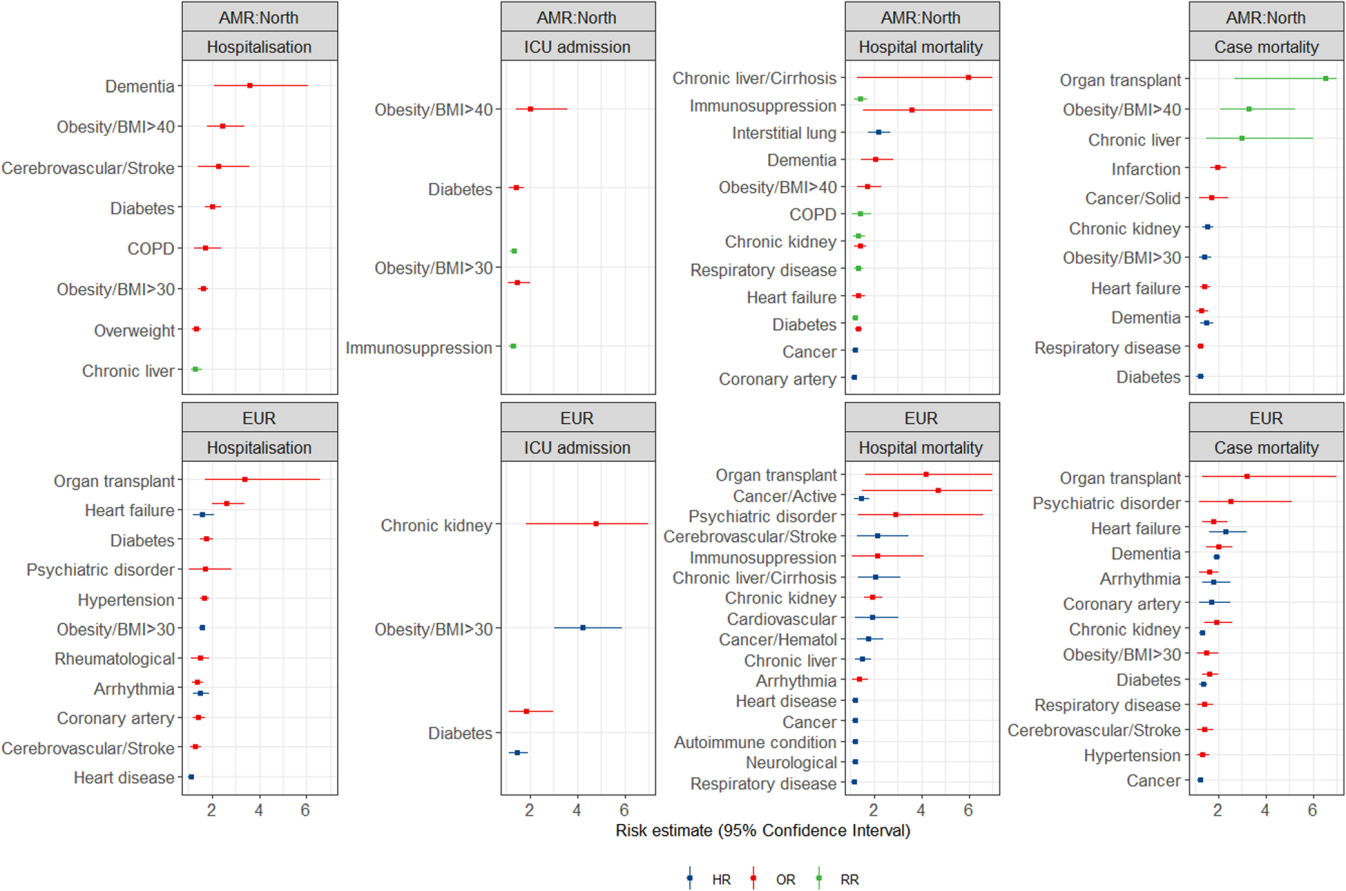
Estimated associations supported by high certainty of evidence (GRADE) presented for each pre-existing condition and outcome for the European region and North America. The estimated associations are arranged by ascending value of measures of effect. However, the presented order should be considered in the context of differences between the statistics (OR, RR, HR)

**Fig. 6:**
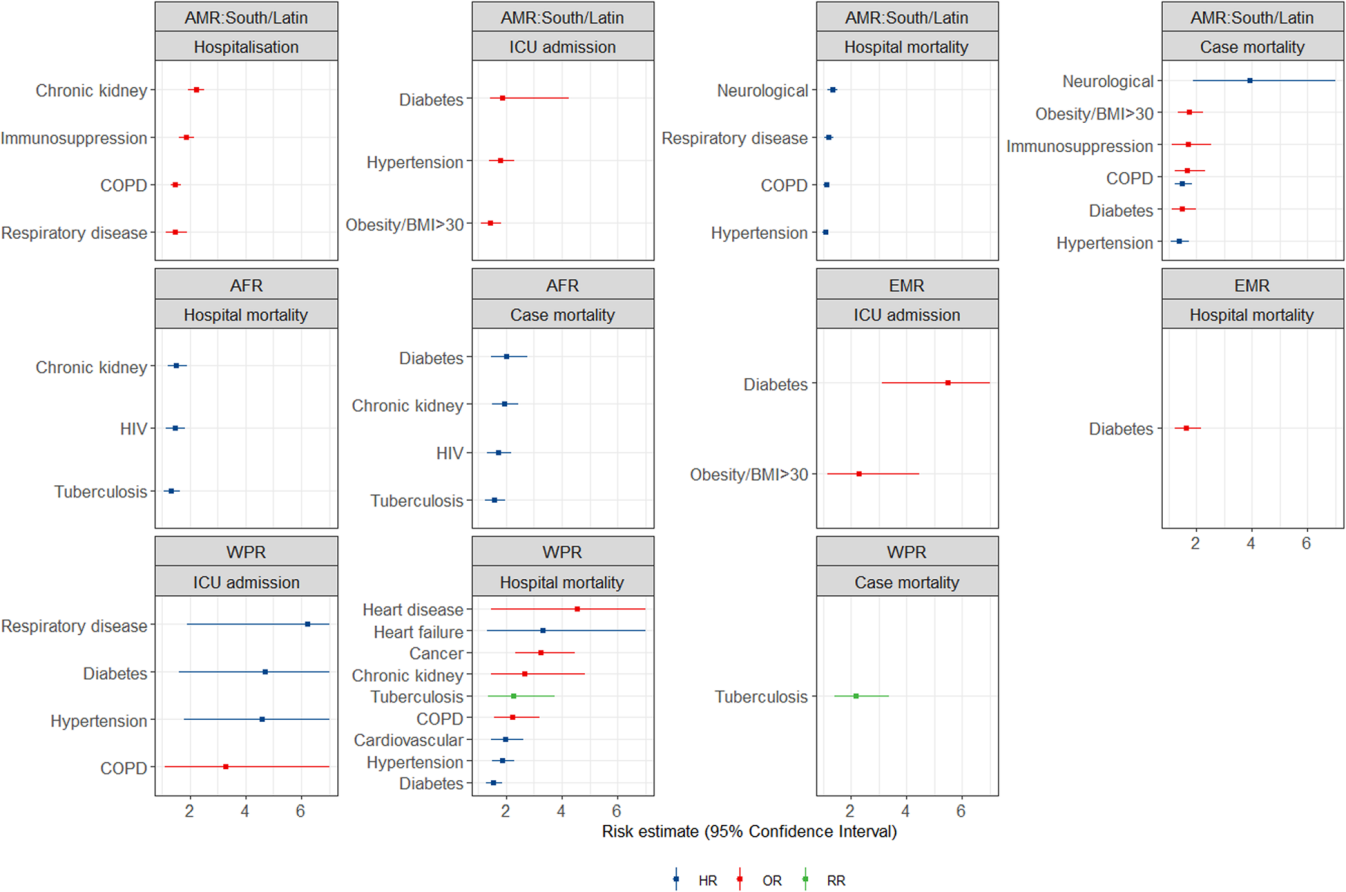
Estimated associations supported by high certainty of evidence (GRADE) presented for each pre-existing condition and outcome for the African, Eastern Mediterranean and Western Pacific regions. The estimated associations are arranged by ascending value of measures of effect. However, the presented order should be considered in the context of differences between the statistics (OR, RR, HR)

Figures 1, 2 and 3 show all estimates (RR, OR or HR) with 95% CI for risk of hospitalisation, ICU admission and death (separately for case mortality and in-hospital mortality) due to COVID-19 in patients with a pre-existing health condition, as compared to patients without the respective condition. Figure 4 illustrates a summary of the GRADE assessment for these estimates. Figures 5 and 6 show associations supported by evidence of high certainty based on the GRADE assessment (also see Additional file 1: Table 10).

Results from meta-analyses and single-study estimates are stratified by seven disease groups and six regions (AFR, EMR, EUR, North America, South/Latin America and WPR). No data was available for SEAR. This section presents the effect estimates with high certainty of evidence based on GRADE (Additional file 1: section 2.6, Table 10). To facilitate reporting, we consider a relative association of 1.5–2.0 as increased risk and of > 2.0 as markedly increased risk regardless of outcome and measure of association. It is, however, essential to note that odds ratio, risk ratio and hazard ratio differ in the interpretation of the estimated association [303].

#### Liver and metabolic diseases

In liver and metabolic diseases (Fig. 1, the upper panel), the evidence was available for the following pre-existing conditions: chronic kidney disease, chronic liver disease (including cirrhosis and hepatitis), diabetes and dyslipidemia or hyperlipidemia. The highest number of estimates (38 estimates) was available for diabetes, including data from all regions. *Diabetes* was associated with an increased risk of hospitalisation in Europe and death in Europe and South/Latin America. A higher risk of death was observed in the study from the African region. Increased risk and markedly increased risk of ICU admission associated with diabetes were found for South/Latin America, EMR and WPR.

The estimates for *chronic kidney disease* showed heterogeneity between the studies across the regions and the outcomes. A markedly increased risk was shown for hospitalisation in South/Latin AMR, ICU admission in EUR, and for in-hospital death in WPR. An increased risk of death was observed in the studies from AFR, AMR North and EUR. Among *chronic liver diseases*, viral hepatitis was the condition with the lowest number of available estimates, with studies from WPR and North America showing no significant increase in the risk of in-hospital death. Regarding other liver diseases, the risk of in-hospital death was markedly increased in patients with *liver cirrhosis* in EUR and North America. Fewest estimates were available from EMR.

#### Respiratory diseases

Regarding lung diseases (Fig. 1, the lower panel), the highest number of estimates was available for the (unspecified) diagnosis group of respiratory diseases (24 estimates), followed by COPD (21 estimates). For *COPD*, all but two risk estimates (for ICU admission and in-hospital mortality in WPR) were either below 2.0 or not significant. COPD was associated with an increased risk of hospitalisation in North America and death in South/Latin America. In patients with *asthma*, risk estimates from three regions showed no significant effect on any of the outcomes but rather a tendency towards decreased risk. Increased risk of hospitalisation and death due to COVID-19 was reported in patients with *tuberculosis* in AFR and WPR. Only one risk estimate from North America was available for *interstitial lung disease*, showing a more than a twofold increased risk of in-hospital death. No estimates were available from EMR. The estimates for respiratory diseases as a generic condition varied greatly, possibly due to differences in the definitions among the studies.

#### Overweight, obesity or underweight

For overweight/obesity/underweight (Fig. 2, the lower panel), most estimates (34 out of 52) came from North America. Patients with severe obesity (BMI ≥ 40 kg/m^2^) had a particularly high risk of severe COVID-19 outcomes, including a more than threefold increased risk of ICU admission and death. Likewise, patients with BMI ≥ 30 kg/m^2^ had a more than two- to fourfold increased risk of COVID-19-related ICU admission in studies from EUR and EMR. Estimates were smaller and mostly non-significant for overweight and underweight, compared to normal weight.

#### Circulatory diseases

For nine circulatory and heart diseases, most of 119 estimates came from North America and Europe (53 and 38 respectively; the upper panel of Fig. 2). In particular, *heart failure* was associated with an increased risk of hospitalisation in EUR and death in EUR and WPR. For *hypertension*, the picture was more heterogeneous. The studies from WPR indicated an association with an increased risk of ICU admission and in-hospital death. Only sparse data with inconsistent results were available for *infarction, peripheral vascular disease* and *venous thromboembolism*. In patients with *coronary artery disease*, mostly small increases in risk or non-significant estimates were observed.

#### Immunodeficiency related conditions

For conditions related to immunodeficiency, data from four regions were available (Fig. 3, the upper panel). Two- to sixfold increased risk was estimated for hospitalisation and death in *organ transplant recipients* in the studies from EUR and AMR North. Geographically heterogeneous results were obtained for people living with *HIV*, with an increased risk of death in AFR, but mostly non-significant estimates in Europe and North America. The estimates for the generic definition of *immunosuppression* vary considerably due to differences in the conditions which compose this group among the studies.

#### Neurological diseases or mental health disorders

In the group of neurological diseases/conditions related to mental health (Fig. 3, the middle panel), patients with *dementia* had a markedly increased risk of hospitalisation and death in North America. However, other available estimates showed some variability. Only one estimate was available for patients with *depression*, showing no increased risk of hospitalisation due to COVID-19 in studies from North America. Results were heterogeneous for *cerebrovascular disease and stroke*, with the majority of estimates being not significant. *Psychiatric disorders* were associated with increased risk of hospitalisation and markedly increased risk of ICU admission and death in EUR.

#### Oncological diseases

Data for patients with oncological diseases were available only from three regions (Fig. 3, the lower panel). *Active cancer* was associated with increased hospital mortality due to COVID-19 in studies from Europe and North America. For *haematological oncological conditions*, only one estimate was available, showing an increased risk of death in hospital in EUR. For unspecified oncological diseases (any cancer or history of), results were heterogeneous.

### Analyses of subgroups

**Fig. 7:**
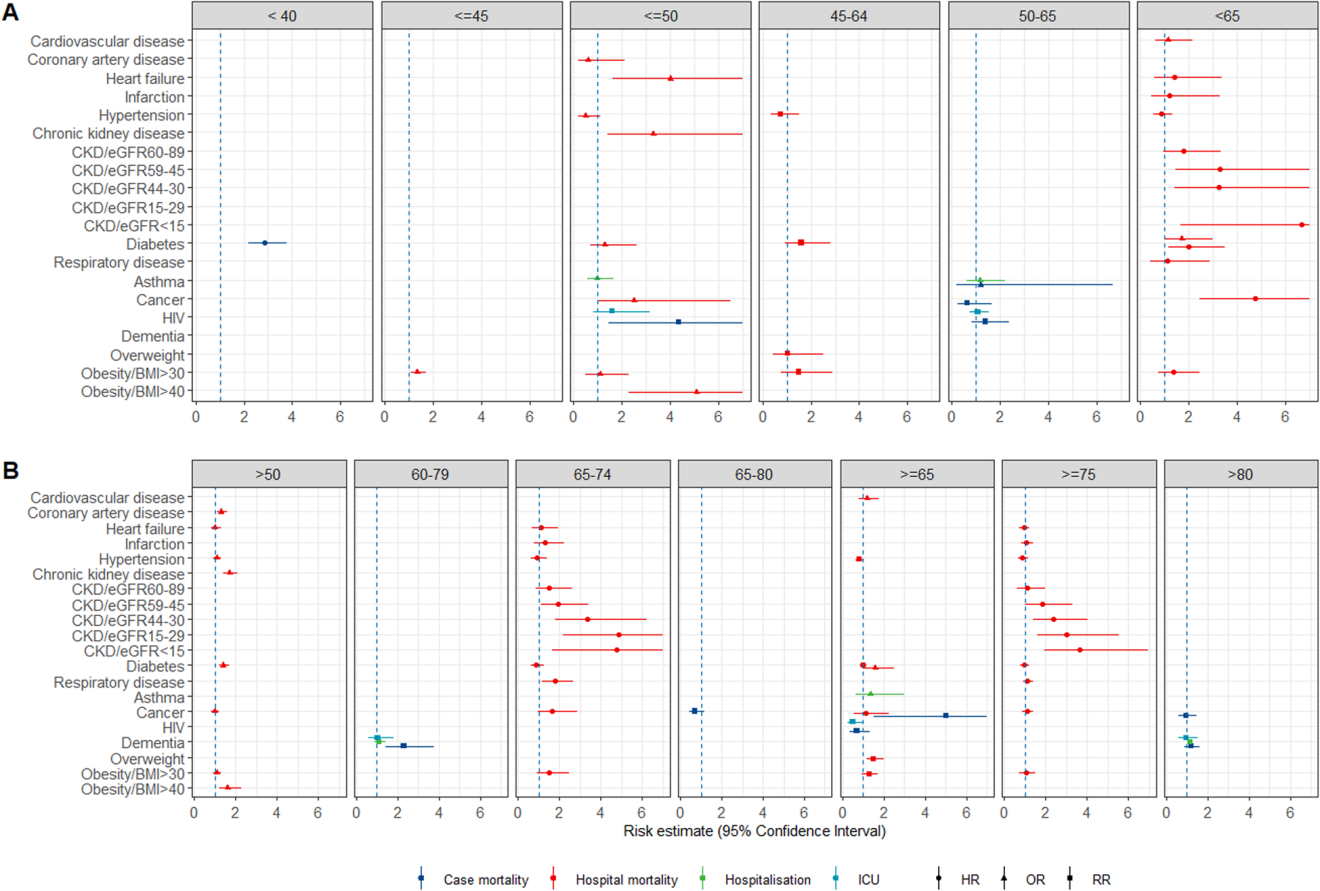
Age-stratified estimates for pre-existing conditions extracted from the single studies. Due to differences in the age groups, the estimates were not pooled. Age groups are illustrated in each column as reported in the primary studies. Panel A illustrates single-study estimates for younger age groups. Panel B gives the estimates for older age groups. The error bars represent 95% confidence intervals. The dashed line indicates 1.0 value. The estimates with error bars crossing the 1.0-line lack statistical significance

Age-stratified estimates from 11 [142, 169, 205, 211, 214, 222–224, 227, 238, 285] (Additional file 1: Table 3) primary studies were used for an analysis of subgroups. The extracted estimates for 20 pre-existing conditions were illustrated across the original age strata reported in the primary studies (Fig. 7, the estimates are given in Additional file 1: section 2.7, Table 11). We did not conduct meta-analyses of age-stratified effects due to a small number of the estimates and heterogeneity of age groups used for stratification.

Fewer estimates were available for people younger than 65 years of age (Fig. 7A). In this group, markedly increased risk of death among < 50-year-olds was reported for heart failure (OR 4.0 (1.6–10.4)), chronic kidney disease (OR 3.3 (1.4–7.7)), severe obesity (BMI ≥ 40 kg/m^2^, OR 5.1 (2.3–11.1)) and HIV (RR 4.36 (1.43–13.3)) in the studies from North America [205, 222] (Fig. 7A, third column from the left). Statistically significant increased risk of death was also seen for diabetes (HR 2.0 (1.15–3.5)) and cancer (HR 4.76 (2.46–9.21)) in one study from the European region [169] (Fig. 7A, the last column). One study from South/Latin America also reported a markedly increased risk of death from diabetes (HR 2.86 (2.19–3.76)) [142] (Fig. 7A, the first column).

In older adults (> 65 years; Fig. 7B), obesity and heart failure were no longer an apparent risk factor for severe COVID-19 outcomes. Chronic kidney disease, diabetes and cancer showed weaker associations. However, hospital mortality due to COVID-19 showed a stepwise increase with decreasing glomerular filtration rate (i.e. all values of eGFR less than 59) in people with chronic kidney diseases in both age groups (Fig. 7, age groups < 65, 65–75, ≥ 75 [169].

## Discussion

### Available evidence

In the COVID-19 pandemic, individuals with chronic pre-existing health conditions are potentially at higher risk for disease progression to severe stages requiring hospitalisation and intensive care and leading to death. However, the occurrence of severe cases predominately in older age groups suggests that the effect of age on poor COVID-19 outcomes is more pronounced [304, 305]. In this umbrella review, we explored the estimated associations of various pre-existing health conditions with COVID-19 outcomes adjusted for the confounding effect of age. Because none of the analysed 120 SRs presented age-adjusted effects for different geographic regions, we evaluated the primary studies in SRs for inclusion and re-analysis.

Most of the evidence was derived from studies conducted in European countries, the USA and China. In contrast, only a few studies were available from EMR and South/Latin America, and there were nearly no studies from AFR and countries of WPR outside of China, and no studies from SEAR.

The results of this review show that heart failure, obesity, diabetes, liver cirrhosis, chronic kidney disease, active and haematological cancer, and history of organ transplantation are associated with an increased risk of poor COVID-19-related outcomes such as hospitalisation, need for intensive care and death. We did not aim to identify the causal effects of the pre-existing conditions but rather to summarise the evidence on the diseases associated with the worsening of COVID-19. Therefore, we could not elicit other underlying factors that could determine the detected associations. However, regional heterogeneity observed for multiple associations suggests an influence of other factors outside the scope of this review.

For example, the association between HIV and COVID-19-related mortality was stronger in the African region than in the European region and North America. Prevalent progressed stages of HIV, poor nutritional status and limited access to antiviral treatment are likely to strengthen the association between HIV and COVID-19-related morality in the African region. Further epidemiological studies conducted in different areas are needed to untangle the effects of potential confounding factors and to estimate the causal effects of pre-existing health conditions.

### Heterogeneity of the effect estimates

Although we selected evidence for clearly defined outcomes and considered WHO regions separately, the strength of associations for the same pre-existing conditions was quite variable. This variability might have stemmed from differences in study designs, the included populations and methodological approaches. Regarding the type of included studies, nearly all studies had a retrospective cohort design. Also, most studies defined COVID-19 case based on PCR. Therefore, these factors are not likely to have influenced the results to a great extent.

Differences in study design and methods such as choice of model covariates, reference age group, age composition of the study population (younger and older groups) and definitions of pre-existing conditions might have driven the observed heterogeneity of the estimates. We placed only one restriction on the included primary studies, i.e. the adjustment for age. However, the majority of published models also adjusted for other factors (race, vital signs, socio-economic factors), which might have led to differences in residual confounding among the estimated effects. Whenever a choice was available, we avoided to include data from models with additional adjustment. However, most of the studies reported only one model structure. Therefore, a decision to conduct a meta-analysis presented a trade-off between reduction of the between-study variability and synthesis of evidence. We addressed the between-study variability by restricting acceptable heterogeneity to 40% and conducting the GRADE evaluation for each estimate.

Differences in the definitions of the pre-existing conditions and composition of the patient groups might have also contributed to the variability. For the meta-analyses, we did not compose larger disease groups and treated every individually defined chronic condition separately whenever possible. This approach allowed the identification of conditions, such as liver cirrhosis and interstitial lung disease, that are associated with a markedly increased risk of death due to COVID-19. Meta-analyses for associations between bodyweight, particularly obesity, and included COVID-19 outcomes presented a challenge due to differences in the definitions among the studies.

Most studies used bodyweight categories or BMI ranges to define underweight, overweight, obesity and severe obesity. A few included studies [135, 138, 179] used BMI as a numeric variable in their models, which were excluded from the meta-analyses of the bodyweight-related associations. We conducted meta-analyses using the bodyweight categories as reported in Additional file 1 (section 2.3). Pooled estimates for obesity showed higher heterogeneity than for other bodyweight categories (see Figs. 2 and 4). Among other bodyweight categories, obesity was mostly reported a pre-existing health condition; however, the definitions of obesity differed among the studies. The following deviations in the definition were observed: (1) Obesity was defined either using BMI metrics or reported as “obesity” without further detail. (2) For the categories built using BMI-based definitions, we included various BMI ranges that lie in BMI ≥ 30 area in the meta-analyses for obesity. If a study differentiated between BMI ≥ 30 and BMI ≥ 40, we included the estimate for BMI ≥ 40 in the respective meta-analysis for BMI ≥ 40. (3) The studies used different comparison categories, i.e. normal weight, BMI < 30 or not obese. Therefore, the associations between obesity and COVID-19 outcomes were likely to be stronger in the studies, which included all patients with obesity and severe obesity into the “obesity” category and compared to the patients with normal weight. These discrepancies in the definition of obesity and other differences in design and analyses likely contributed to the heterogeneity of the pooled estimates of associations for obesity.

Meta-analyses based on unspecific generic definitions of disease groups such as respiratory or cardiovascular diseases can be less informative. Due to the broad definition of the risk factor, individual conditions which are potentially associated with a higher risk of poor COVID-19 outcomes are not identifiable. However, it has to be noted that the broader definitions of risk factors had to be made in the studies with the smaller sample sizes. Nonetheless, the evidence on the strength of effects for disease groups available in the studies provides direction for further investigation when more data are available. Also, it has to be considered that inherent predispositions do not drive the associations with the undesirable COVID-19 outcomes alone. Instead, other factors such as local medical standards, therapeutic decisions made in hospitals and self-preserving behaviours of certain patient groups during the pandemic, including social distancing and wearing face masks, might have influenced the outcomes. These factors may contribute to observed geographical variation and explain paradox findings, such as unexpectedly lower risk estimates for certain pre-existing conditions such as asthma. The influence of these factors, however, could not be untangled in this review.

### Strengths and limitations

Our study has several strengths. To our knowledge, it is the first comprehensive global overview on this topic. It comprises information from 120 reviews conducted worldwide, providing a solid basis for decision-makers regarding the effects of pre-existing health conditions on severe and fatal COVID-19 outcomes. Applying a rigorous methodological approach regarding SRs and primary studies, the umbrella review also allowed for identifying gaps in the evidence and thus priorities for future research. The limitations of this umbrella review mainly stem from the limitations of the included SRs and primary studies. Lack of a registered protocol and reporting on excluded studies observed in the systematic review could have affected our initial pool of the primary studies.

Due to our approach, primary studies that were (for any reason) not included in the systematic reviews could also not be included in our analysis. Most primary studies included in SRs and analysed here were conducted in the first wave of the pandemic. In times of rapidly accumulating evidence, this might be seen as a weakness of our approach. To avoid additional heterogeneity, we excluded studies that used the rather unspecified outcome “severe COVID-19”. By further restricting our analysis to age-adjusted estimates, we might have excluded evidence from countries that are now not otherwise represented in our review. Although 14 [138, 145, 149, 171, 198, 207, 218, 232, 241, 259, 265, 273, 288, 293] of the primary studies in our selection have not yet been peer-reviewed (but were published on pre-print servers), they were included in our analyses to increase the evidence pool. These studies were reviewed and evaluated in the included systematic reviews; however, their inclusion might limit our work.

### Implication of evidence for policy

This review provides a global overview of currently available evidence across disease groups and geographic regions. Thereby, our work can support decision-makers worldwide in the process of identifying those who are at particularly high risk of hospitalisation, admission to intensive care unit and death related to COVID-19. This might be important for prioritising certain patient groups for vaccination against COVID-19, given the global vaccine supply shortage. It is, however, outside the scope of this review to provide guidance on prioritisation of the patient groups because the decision-making would require setting threshold values for relative risk measures that would indicate higher or lower priority. The evidence collected in this review allows identification of pre-existing conditions, which increase the risk of COVID-19-related health outcomes relevant to public health, and can facilitate decision-making. Further, the risk estimates combined with the disease definitions extracted from the primary studies may help clinicians to identify patients at higher risk for poor COVID-19-related health outcomes.

It is important to note that, despite the abundance of evidence, identification of further high-risk groups remains a challenging issue. Individuals with rare diseases or exacerbated health conditions are likely to be underrepresented in the populations included in the primary studies possibly due to self-preserving behavioural changes in response to the COVID-19 pandemic. As a consequence, rare chronic conditions may be overlooked in decision-making. Also, we are aware of the fact that in a given country context, a variety of other factors, including advocacy groups, special population groups, media press and lobbying, influence the prioritisation procedure. Therefore, it is important to support NITAGs with evidence on risk groups to enable them to develop evidence-based vaccination recommendation and defend them adequately in public discussions.

### Implication of evidence for research

The results of our review might be used to identify further research needs. The review shows a gap in evidence on age-adjusted effect estimates in the countries from the African and the South-East Asia regions and the countries of the Western Pacific region outside of China. As the profile of prevalent chronic pre-existing conditions and age structure in these populations differ from the European and American regions, further research would provide valuable information.

Currently, few studies conduct age-adjusted analysis using age-stratified data. Based on the current data, it is reasonable to suggest that age is also an effect modifier. Therefore, further investigation of the effects of pre-existing conditions on COVID-19 outcomes stratified by age groups is needed to unmask health condition effects existent in specific age bands.

Additionally, it can be informative to consider chronic conditions in greater granularity. For example, differentiation for severity for some pre-existing conditions (e.g. diabetes, HIV, depression, chronic kidney disease) can identify more specific target patient groups for policy actions or clinical practice. It is also helpful to explore the effects of multimorbidity and effect modifications among pre-existing health conditions. Furthermore, the standardisation or provision of complete definitions of COVID-19 disease and pre-existing conditions would allow for greater comparability across studies.

It is also desirable that future studies identify the reason for admission to hospital or intensive care. For severe episodes of pre-existing conditions such as active cancer, COPD, chronic kidney and liver disease, it was impossible to differentiate whether the admission was due to COVID-19 or due to worsening of the underlying health condition. The strength of association with COVID-19-related outcome might have been overestimated in the studies, which included patients admitted with an exacerbation of pre-existing condition and consequently found positive for the infection.

Also, an update and transformation of this review into a living review would be useful to add evidence that has emerged in the second and third waves of the COVID-19 pandemic.

Finally, this work summarises the evidence on the associations between the pre-existing health conditions and undesirable COVID-19 outcomes without looking into plausible biological factors that could provide an insight into the resulted estimates. However, it is known that SARS-CoV-2 targets epithelial cells of the nasal and bronchioalveolar tract where the spike-S-glycoprotein of the virus binds via the angiotensin-converting enzyme (ACE)-2 receptor [306]. High densities of ACE-2 receptors are present in the respiratory tract, the gut, vessels and kidneys. An upregulated expression of ACE-2 receptors is also associated with several pre-existing chronic health conditions: hypertension, COPD, diabetes, liver and kidney diseases [307]. Whether or not a strong expression of ACE-2 receptors offers a mechanistic explanation for the associations between chronic diseases and severe COVID-19 outcomes described here needs to be investigated.

## Conclusions

In this review, a number of pre-existing conditions were associated with hospitalisation, ICU admission and death in hospitalised with COVID-19 and SARS-CoV-2-positive individuals when controlled for age. The strength of associations varied supposedly due to differences in definitions of pre-existing conditions and methodological approaches. The review shows multiple gaps in evidence, including a pressing need for evidence from the African, South-East-Asian and Western Pacific regions, exploration of effects of multimorbidity and rare diseases. The results may serve as an efficient starting point for policy-makers to prioritise patient groups for protection via vaccination and other health interventions against COVID-19.

## Supplementary Information

**Additional file 1.** Pre-existing health conditions and severe COVID-19 outcomes: an umbrella review approach and meta-analysis of global evidence”. This supplementary file provides details on study search and selection, the results of the searches, data extraction from reviews and primary studies, and meta-analyses. The tables give all obtained pooled estimates of the main analyses, including the GRADE assessment. Age-stratified estimates and analysis of specific population groups are presented. The file also provides results of the risk of bias evaluation conducted in this review and the lists of excluded SRs and primary studies.

## Data Availability

All data generated and analysed during this study are included in this published article and its supplementary information file (Additional file 1).

## Abbreviations

ACE-2: Angiotensin-converting enzyme-2
AFR: African region
AMR: Regions of America
BMI: Body mass index
COPD: Chronic obstructive pulmonary disease
COVID-19: Coronavirus disease 2019
EMR: Eastern Mediterranean region
EUR: European region
GRADE: Grading of Recommendations Assessment, Development and Evaluation
HR: Hazard ratio
ICU: Intensive care unit
NITAGs: National Immunisation Technical Advisory Groups
NOS: The Newcastle-Ottawa Scale
OR: Odds ratio
PRECEPT: Project on a Framework for Rating Evidence in Public Health
RR: Relative risk
SARS-CoV-2: Severe acute respiratory syndrome coronavirus type 2
SEAR: South-East Asia region
SR: Systematic review
WHO: The World Health Organization
WHO SAGE: The World Health Organization’s Strategic Advisory Group of Experts on Immunisation
