# Supplementary material for: Pre-existing health conditions and severe COVID-19 outcomes: an umbrella review approach and meta-analysis of global evidence
Source: BMC Med. 2021 Aug 27;19:212. doi: 10.1186/s12916-021-02058-6 (PMC8390115; doi:10.1186/s12916-021-02058-6)
Supplement: Supplementary file 1 — Additional file 1. Pre-existing health conditions and severe COVID-19 outcomes: an umbrella review approach and meta-analysis of global evidence”. This supplementary file provides details on study search and selection, the results of the searches, data extraction from reviews and primary studies, and meta-analyses. The tables give all obtained pooled estimates of the main analyses, including the GRADE assessment. Age-stratified estimates and analysis of specific population groups are presented. The file also provides results of the risk of bias evaluation conducted in this review and the lists of excluded SRs and primary studies. [file 12916_2021_2058_MOESM1_ESM.pdf]

Additional File 1

Pre-existing health conditions and severe COVID-19 outcomes: an umbrella review approach and meta-analysis of global evidence

Marina Treskova-Schwarzbach, Laura Haas, Sarah Reda, Antonia Pilic, Anna Borodova, Kasra Karimi, Judith Koch, Teresa Nygren, Stefan Scholz, Viktoria Schönfeld, Sabine Vygen-Bonnet, Ole Wichmann, Thomas Harder

Robert Koch Institute, Immunisation Unit, 13353 Berlin, Germany

Contents

1. Methods 4
1.1. Search strategy 4
1.2. Study selection 4
1.3. Data extraction from systematic literature reviews 5
1.4. Evaluation with AMSTAR 2 5
1.5. Data extraction from primary studies 5
2. Results 5
2.1. Overview of included reviews and primary studies 5
Figure 1. Selection of systematic reviews 6
Figure 2. Selection of primary studies 7
Table 1 Overview of included systematic reviews (n=120). 8
Figure 3. Overview of included systematic reviews (SRs). 17
Table 2 Results of quality evaluation of the included systematic reviews 18
Table 3 Overview of included primary studies (n=160) 22
2.2. Summary of the studies based on the general population 47
Table 4 Overview of the studies which report community-based estimates (n=9) 48
2.3. Definitions of pre-existing conditions 51
2.4. List of estimates excluded from reporting 57
2.5. Results of the meta-analyses 58
Table 5. 1 Risk of hospitalisation in persons with liver and metabolic diseases: meta-analysis and confidence in the estimate (GRADE) 58
Table 5. 2 Risk of hospitalisation in persons with respiratory diseases: meta-analysis and confidence in the estimate (GRADE) 58
Table 5. 3 Risk of hospitalisation in persons with circulatory diseases: meta-analysis and confidence in the estimate (GRADE) 59
Table 5. 4 Risk of hospitalisation in persons with overweight, obesity or underweight: meta-analysis and confidence in the estimate (GRADE) 60
Table 5. 5 Risk of hospitalisation in persons with immunodeficiency: meta-analysis and confidence in the estimate (GRADE) 61
Table 5. 6 Risk of hospitalisation in persons with neurological diseases or mental health disorders: meta-analysis and confidence in the estimate (GRADE) 62
Table 5. 7 Risk of hospitalisation in persons with oncological diseases: meta-analysis and confidence in the estimate (GRADE) 62
Table 6. 1 Risk of death (in-hospital mortality) in persons with liver and metabolic diseases: meta-analysis and confidence in the estimate (GRADE) 62

Table 6. 2 Risk of death (in-hospital mortality) in persons with respiratory diseases: meta-analysis and confidence in the estimate (GRADE) 64

Table 6. 3 Risk of death (in-hospital mortality) in persons with circulatory diseases: meta-analysis and confidence in the estimate (GRADE) 65

Table 6. 4 Risk of death (in-hospital mortality) in persons with overweight, obesity or underweight: meta-analysis and confidence in the estimate (GRADE) 67

Table 6. 5 Risk of death (in-hospital mortality) in persons with immunodeficiency: meta-analysis and confidence in the estimate (GRADE) 68

Table 6. 6 Risk of death (in-hospital mortality) in persons with neurological diseases or mental health disorders: meta-analysis and confidence in the estimate (GRADE) 69

Table 6. 7 Risk of death (in-hospital mortality) in persons with oncological diseases: meta-analysis and confidence in the estimate (GRADE) 70

Table 7. 1 Risk of death (case mortality) in persons with liver and metabolic diseases: meta-analysis and confidence in the estimate (GRADE) 70

Table 7. 2 Risk of death (case mortality) in persons with respiratory diseases: meta-analysis and confidence in the estimate (GRADE) 71

Table 7. 3 Risk of death (case mortality) in persons with circulatory diseases: meta-analysis and confidence in the estimate (GRADE) 72

Table 7. 4 Risk of death (case mortality) in persons with overweight, obesity or underweight: meta-analysis and confidence in the estimate (GRADE) 73

Table 7. 5 Risk of death (case mortality) in persons with immunodeficiency: meta-analysis and confidence in the estimate (GRADE) 74

Table 7. 6 Risk of death (case mortality) in persons with neurological diseases or mental health disorders: meta-analysis and confidence in the estimate (GRADE) 75

Table 7. 7 Risk of death (case mortality) in persons with oncological diseases: meta-analysis and confidence in the estimate (GRADE) 75

Table 8. 1 Risk of ICU admission in persons with liver and metabolic diseases: meta-analysis and confidence in the estimate (GRADE) 76

Table 8. 2 Risk of ICU admission in persons with respiratory diseases: meta-analysis and confidence in the estimate (GRADE) 77

Table 8. 3 Risk of ICU admission in persons with circulatory diseases: meta-analysis and confidence in the estimate (GRADE) 77

Table 8. 4 Risk of ICU admission in persons with overweight, obesity or underweight: meta-analysis and confidence in the estimate (GRADE) 78

Table 8. 5 Risk of ICU admission in persons with immunodeficiency: meta-analysis and confidence in the estimate (GRADE) 78

Table 8. 6 Risk of ICU admission in persons with neurological diseases or mental health disorders: meta-analysis and confidence in the estimate (GRADE) 78

Table 8. 7 Risk of ICU admission in persons with oncological diseases: meta-analysis and confidence in the estimate (GRADE) 79

Table 9. 1 Risk of intubation in persons with liver and metabolic diseases: meta-analysis and confidence in the estimate (GRADE) 79

Table 9. 2 Risk of intubation in persons with respiratory diseases: meta-analysis and confidence in the estimate (GRADE) 80

Table 9. 3 Risk of intubation in persons with circulatory diseases: meta-analysis and confidence in the estimate (GRADE) 80

Table 9. 4 Risk of intubation in persons with overweight, obesity or underweight: meta-analysis and confidence in the estimate (GRADE) 81

Table 9. 5 Risk of intubation in persons with immunodeficiency: meta-analysis and confidence in the estimate (GRADE) 82

Table 9. 6 Risk of intubation in persons with neurological diseases or mental health disorders: meta-analysis and confidence in the estimate (GRADE) ... 82

Table 9. 7 Risk of intubation in persons with oncological diseases: meta-analysis and confidence in the estimate (GRADE) ... 82

Figure 4. Ranges of the estimates with considerable between-study heterogeneity for respiratory and liver and metabolic diseases ... 83

Figure 5. Ranges of the estimates with considerable between-study heterogeneity for circulatory diseases, overweight, obesity, and underweight ... 84

Figure 6. Ranges of the estimates with considerable between-study heterogeneity for immunodeficiency, oncological disease, neurological and mental health disorders ... 85

2.6. Estimated associations supported by high quality of evidence ... 86

Table 10 Estimated associations supported by high quality of evidence (GRADE) presented for each pre-existing condition and outcome across the WHO-regions ... 86

2.7. Age-stratified estimates ... 89

Table 11 Age-stratified estimates extracted from single studies (as reported by primary studies) ... 89

2.8. Summary of evidence for specific population groups ... 94

2.9. Risk of bias evaluation ... 95

Table 12 Results of risk of bias evaluation with the Newcastle-Ottawa Scale (NOS) (n=17) ... 95

2.10. Systematic reviews excluded after eligibility assessment ... 101

2.11. Excluded primary studies ... 102

3. References ... 109

1. Methods

1.1. Search strategy

Search Syntax PubMed 1:
("Severe Acute Respiratory Syndrome Coronavirus 2" [Supplementary Concept] OR "COVID-19" [Supplementary Concept] OR "COVID 19 diagnostic testing" [Supplementary Concept] OR "COVID 19 drug treatment" [Supplementary Concept] OR "COVID 19 serotherapy"[Supplementary Concept] OR "COVID 19 vaccine" [Supplementary Concept] OR "Severe Acute Respiratory Syndrome Coronavirus 2"[tiab] OR ncov*[tiab] OR COVID*[tiab] OR sars-cov-2[tiab] OR "sars cov 2"[tiab] OR "SARS Coronavirus 2"[tiab] OR "Severe Acute Respiratory Syndrome CoV 2"[tiab] OR "Wuhan coronavirus"[tiab] OR "Wuhan seafood market pneumonia virus"[tiab] OR "SARS2"[tiab] OR "2019-nCoV"[tiab] OR "hcov-19"[tiab] OR „novel 2019 coronavirus“[tiab] OR "2019 novel coronavirus*"[tiab] OR „novel coronavirus 2019*“[tiab] OR "2019 novel human coronavirus*"[tiab] OR „human coronavirus 2019“[tiab] OR "coronavirus disease-19"[tiab] OR "corona virus disease-19"[tiab] OR "coronavirus disease 2019"[tiab] OR "corona virus disease 2019"[tiab] OR "2019 coronavirus disease"[tiab] OR "2019 corona virus disease"[tiab] OR „novel coronavirus disease 2019“[tiab] OR „novel coronavirus infection 2019“[tiab] OR "new coronavirus*"[tiab] OR "coronavirus outbreak"[tiab] OR "coronavirus epidemic"[tiab] OR "coronavirus pandemic"[tiab] OR "pandemic of coronavirus"[tiab]) AND ("2019/12/01"[PDAT] : "2099/12/31"[PDAT])

Search Syntax PubMed 2:
("wuhan"[tiab] or china[tiab] or hubei[tiab]) AND ("Severe Acute Respiratory Syndrome Coronavirus 2"[Supplementary Concept] OR "COVID-19" [Supplementary Concept] OR "COVID 19 diagnostic testing"[Supplementary Concept] OR "COVID 19 drug treatment"[Supplementary Concept] OR "COVID 19 serotherapy"[Supplementary Concept] OR "COVID 19 vaccine"[Supplementary Concept] OR "coronavirus*"[tiab] OR "corona virus*"[tiab] OR ncov[tiab] OR COVID*[tiab] OR sars*[tiab])

Search Syntax Embase 1:
('severe acute respiratory syndrome coronavirus 2':ti,ab OR 'severe acute respiratory syndrome coronavirus 2'/exp OR 'COVID 19'/exp OR ncov*:ti,ab OR COVID*:ti,ab OR 'sars cov 2':ti,ab OR 'sars-cov-2':ti,ab OR 'sars coronavirus 2':ti,ab OR 'sars coronavirus 2'/exp OR 'severe acute respiratory syndrome cov 2':ti,ab OR 'wuhan coronavirus':ti,ab OR 'wuhan seafood market pneumonia virus':ti,ab OR sars2:ti,ab OR '2019-ncov':ti,ab OR 'hcov-19':ti,ab OR 'novel 2019 coronavirus':ti,ab OR '2019 novel coronavirus*':ti,ab OR 'novel coronavirus 2019'/exp OR '2019 novel human coronavirus*':ti,ab OR 'human coronavirus 2019':ti,ab OR 'coronavirus disease-19':ti,ab OR 'corona virus disease-19':ti,ab OR 'coronavirus disease 2019':ti,ab OR 'coronavirus disease 2019'/exp OR 'corona virus disease 2019':ti,ab OR '2019 coronavirus disease':ti,ab OR 'novel coronavirus 2019*':ti,ab OR 'novel coronavirus disease 2019':ti,ab OR 'novel coronavirus infection 2019':ti,ab OR '2019 corona virus disease':ti,ab OR 'new coronavirus*':ti,ab OR 'coronavirus outbreak':ti,ab OR 'coronavirus epidemic':ti,ab OR 'coronavirus pandemic':ti,ab OR 'pandemic of coronavirus':ti,ab OR 'severe acute respiratory syndrome coronavirus 2 vaccine'/exp OR 'COVID 19 vaccine'/exp) AND 2020:py

Search Syntax Embase 2:
(wuhan:ti,ab OR china:ti,ab OR hubei:ti,ab) AND ('severe acute respiratory syndrome coronavirus 2':ti,ab OR 'severe acute respiratory syndrome coronavirus 2'/exp OR 'severe acute respiratory syndrome coronavirus 2' OR 'COVID*':ti,ab OR 'COVID 19'/exp OR 'COVID 19' OR coronavirus*:ti,ab OR 'corona virus*':ti,ab OR ncov:ti,ab OR COVID*:ti,ab OR sars*:ti,ab OR 'sars coronavirus 2'/exp)

Manual search in ArRvix, BioRvix, ChemRvix, MedRvix, Preprints.org, ResearchSquare und SSRN

These search terms will be combined with "systematic review OR meta-analysis"[all fields] to identify eligible systematic reviews.

1.2. Study selection

For inclusion into this review, a paper has to meet the following criteria:

i. Systematic review with/without meta-analysis
ii. Investigate one or more risk factors such as age and underlying health conditions (including but not limited to asthma, chronic obstructive pulmonary disease, malignancy, diabetes mellitus, cardiovascular diseases, chronic renal disease, diseases of the liver, chronic diseases of the digestive system, hypertension, obesity, and immunocompromised conditions)
iii. Investigate the association between at least one of the risk factors and at least one of the following health outcomes:
    - SARS-CoV-2 infection: acquisition of symptomatic infection

- COVID-19 progression: development of moderate and severe symptoms, hospitalisation, admission to intensive care, ventilation, duration of hospital stay, and (or duration of) ventilation, death

iv. Report measures of associations (odds ratios, hazard ratios, risk ratios) for at least one risk factor and health outcome
v. No restrictions on the study design of the primary studies
vi. No date restriction
vii. No language restriction

Exclusion criteria are:

i. Reviews that summarise case reports
ii. Reviews that explore the complications of COVID-19 disease
iii. Reviews that examine or model predictors (defined as medical tests, vital signs) of COVID-19 progression
iv. Review that focus on biological properties of the virus
v. Reviews that investigate animal studies
vi. Conference abstracts and protocols of reviews

1.3. Data extraction from systematic literature reviews

One reviewer conducted data extraction using a predefined data extraction table. The second reviewer confirmed extracted data. The following information was extracted from full texts of included systematic reviews: Title, main author, publication date, date of last literature search, journal, study design (whether meta-analysis), names of databases searched, number of included primary studies, subject characteristics, explored risk factors, included COVID-19-related health outcomes, methods of quality assessment (if conducted), industry sponsoring (of review).

1.4. Evaluation with AMSTAR 2

At the beginning of quality appraisal, the full evaluation of all 16 item was conducted. Later, appraisals were abbreviated by focusing on the critical AMSTAR-2 items, using a cut-off as soon as more than one critical item was not met. More than one critical flaw in an AMSTAR-2 quality appraisal leads to a critically low quality rating.

1.5. Data extraction from primary studies

Authors, title, DOI, journal, country, language, region of country, industry funding, study design, setting, enrolled cohort/ study population, number of patients, age (mean (SD), median (IQR)), percentage of men, COVID-19 case (diagnosis), source of information for pre-existing conditions, outcome/ endpoints, measures of association, methods of adjustment, covariates in adjustment, number of comorbidities included in the model (including BMI and CCI), adjustment for smoking, adjustment for BMI/ obesity, reference age (group) and sex, whether definitions of comorbidities were given. From systematic reviews, we randomly extracted quality evaluation preferring the ones with ROBIN-2 or NOS assessment. Reviews which included the study were also listed.

2. Results

2.1. Overview of included reviews and primary studies

Figures 1 and 2 illustrate selection of systematic reviews and primary studies for re-analysis.
Table 1 gives an overview on the most relevant information from included systematic literature reviews as well as the AMSTAR-2 quality ratings (high, moderate, low or critically low).
Table 2 shows ratings of each AMSTAR-2 item for included systematic reviews.
Table 3 gives an overview of included primary studies.
Table 4 summarises the studies with community-based estimates.

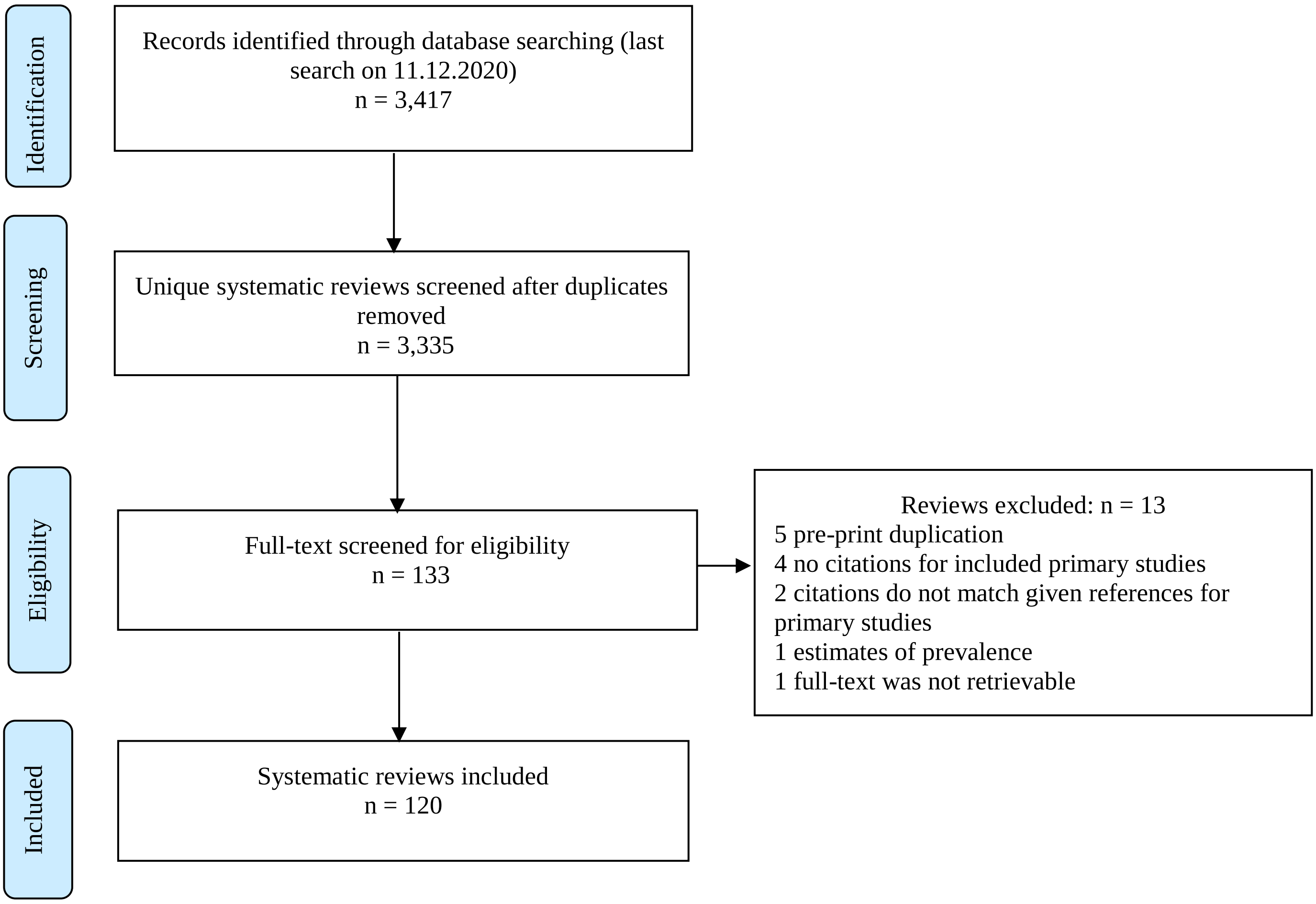

Figure 1. Selection of systematic reviews

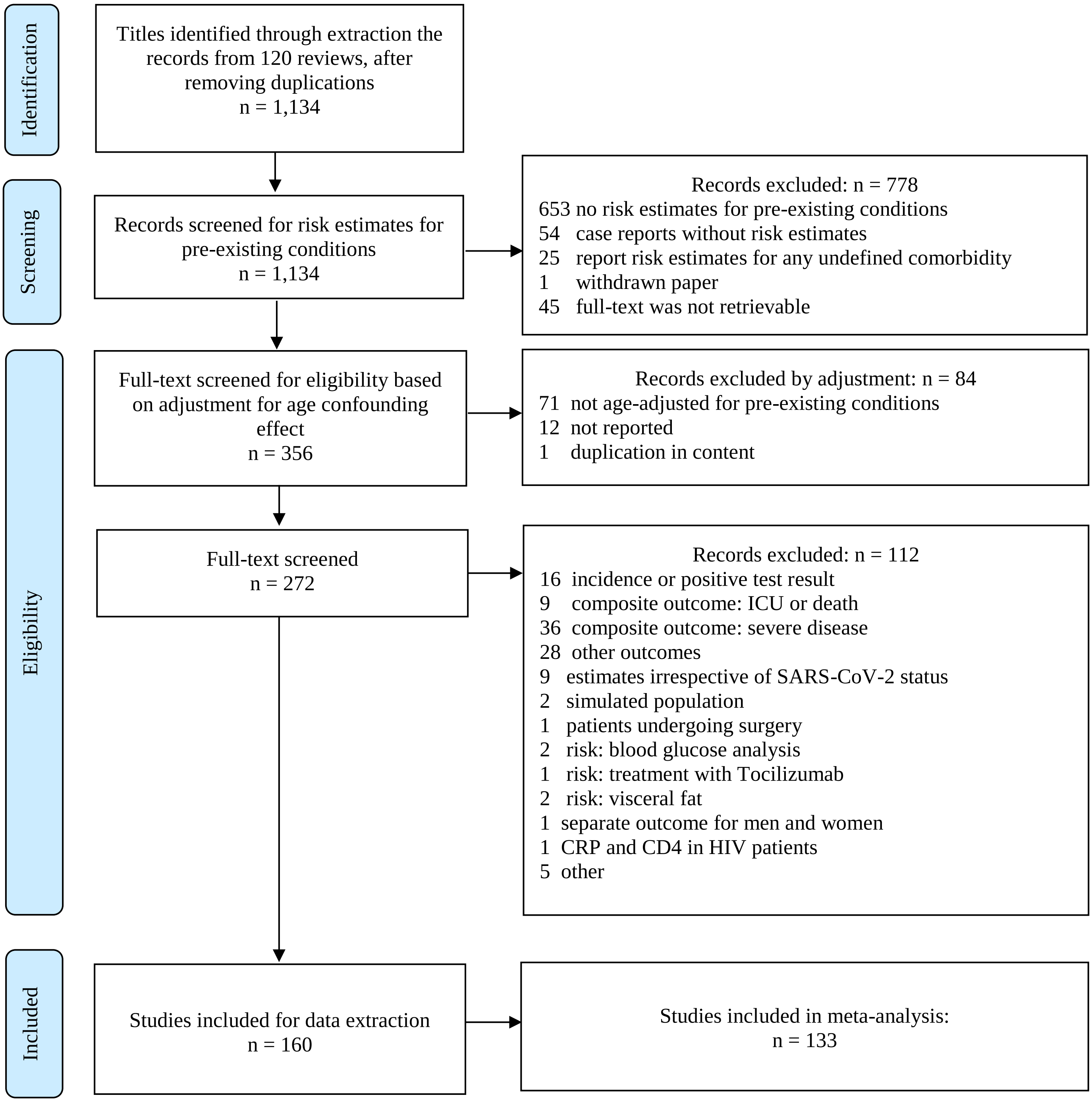

Figure 2. Selection of primary studies

Table 1 Overview of included systematic reviews (n=120).

Author	Last date of search	Names of databases searched	Risk factors	Outcomes related to COVID-19	Instrument of Quality appraisal	Age-adjusted pooled estimates	AMSTAR rating	Citation
Aggarwal	20.04.20	PubMed, Embase, CENTRAL	comorbidities (cardiovascular disease)	severity, mortality	NOS	unadjusted	critically low	[1]
Awortwe	17.06.20	PubMed, MEDLINE, Scopus, Google Scholar	comorbidities (cardiovascular disease, cerebrovascular disease, hypertension, diabetes, chronic kidney disease, COPD)	severity, mortality, ICU admission	nr	not considered	critically low	[2]
Bellou	19.04.20	PubMed	comorbidities (cerebrovascular disease, COPD, diabetes), sex, lab values, symptoms	severity, mortality, ICU admission, intubation	nr	unadjusted	critically low	[3]
Biswas (1)	21.05.20	PubMed	comorbidities (diabetes, hypertension, cardiovascular disease, respiratory disease, cerebrovascular disease, chronic kidney disease, chronic liver disease, cancer), age, sex	severity	NOS	unadjusted	critically low	[4]
Biswas (2)	25.03.20	PubMed, The Cochrane Library, Scinapse, New Engl, Journal of Medicine, Lancet, Journal of American Medical Association, Nature, British Medical Journal	comorbidities (hypertension, diabetes, respiratory disease, cardio-cerebrovascular disease, kidney disease, coronary heart disease), age, sex	mortality	NOS, Jadad	unadjusted	critically low	[5]
Chang	30.05.20	MEDLINE, Embase, The Cochrane Library	obesity	severity, hospitalisation*, intubation	NOS	unadjusted	critically low	[6]
Chen	06.03.20	PubMed, WoS, CNKI, Wanfang Data, VIP	comorbidities (hypertension, diabetes, coronary heart disease), lab values	severity	NOS	unadjusted	critically low	[7]
Cheruiyot	26.05.20	PubMed, CNKI, MEDLINE	comorbidities (cancer)	severity, mortality	MINORS	unadjusted	critically low	[8]
Chidambaram	08.05.20	PubMed, Embase, WHO	comorbidites (diabetes, hypertension, heart failure), lab values	severity, mortality	NOS	unadjusted	critically low	[9]
Chu	24.05.20	PubMed, Embase, WoS	obesity	severity, mortality, in-hospital mortality, ICU admission, intubation, other	NOS	considered	critically low	[10]
Das	02.10.20	PubMed, Google Scholar, ScienceDirect	obesity	severity	NOS	unadjusted	critically low	[11]
De Almeida-Pititto	06.05.20	PubMed, Cochrane Libray, SciELO	comorbidities (diabetes, hypertension, cardiovascular disease), treatment	severity, mortality, ICU admission, intubation	nr	unadjusted	critically low	[12]

Degarege	06.05.20	PubMed, Embase, The Cochrane Library, CINHAL	comorbidities (hypertension, cardiovascular disease, respiratory disease), age, sex	severity, mortality	other	considered; pooled adjusted and unadjusted	critically low	[13]
Dorjee	31.08.20	MEDLINE, Embase, WoS	comorbidities (COPD, cardiovascular disease, chronic kidney disease, diabetes, hypertension), complications	mortality*, other	NOS	considered	critically low	[14]
Du	27.08.20	MEDLINE, PubMed, Embase, WoS	comorbidities (diabetes, hypertension, cardiovascular disease), age, sex, heightened BMI	severity, mortality	NOS	adjusted	critically low	[15]
Elgohary	20.04.20	PubMed, WoS, Scopus	comorbidities (cancer), lab values	severity, mortality, ICU admission, intubation	nr	unadjusted	critically low	[16]
Figliozzi	24.04.20	MEDLINE, WoS, Scopus, CINAHL	comorbidities (cardiovascular disease, immune and metabolic disorders, respiratory disease, cerebrovascular disease, cancer, renal disease, liver disease)	in-hospital mortality	NOS	unadjusted	critically low	[17]
Florez-Perdomo	01.05.20	PubMed, Embase, EBSCO Host, Scopus, ScienceDirect, MEDLINE, Lilacs	comorbidities (cerebrovascular disease, stroke)	severity, mortality	ROBINS-I, NOS	unadjusted	critically low	[18]
Földi	11.05.20	PubMed/MEDLINE, Embase, CENTRAL, Scopus, WoS	obesity	ICU admission, intubation	QUIPS	unadjusted	critically low	[19]
Gao	12.04.20	PubMed, Embase, CENTRAL, WoS, CBM, CNKI, Wanfang Data	comorbidities (cancer)	severity, mortality	NOS	not considered	critically low	[20]
Giannakoulis	27.04.20	PubMed, MedRxiv, COVID-19 Open research Dataset (CORD-19)	comorbidities (cancer), age	severity, mortality, ICU admission	other	unadjusted	critically low	[21]
Guo	30.05.20	PubMed, WoK, MedRxiv, bioRxiv, CNKI, Wanfang Data	comorbidities (diabetes)	severity, mortality	nr	unadjusted	critically low	[22]
Hariyanto	25.10.20	PubMed, Europe PMC	comorbidities (dementia)	severity, mortality, other	NOS	not considered	critically low	[23]
Hessami	27.03.20	PubMed, Embase, The Cochrane Library, Scopus, WoS, MedRxiv, Google Scholar, other journal websites	comorbidities (hypertension, heart failure, coronary artery disease, cardiovascular disease)	severity, mortality, ICU admission	NOS	not considered	critically low	[24]
Huang	10.08.20	PubMed, Embase, WoS, Chinese National Knowledge Infrastructure (CNKI), Wanfang Data, SinoMed, MedRxiv	obesity, heightened BMI, lab values	severity, mortality, hospitalisation*, ICU admission, intubation	NOS	considered	low	[25]
Hussain	26.04.20	PubMed	comorbidities (diabetes)	ICU admission, hospitalisation*	NOS	not considered	critically low	[26]
Islam	17.05.20	PubMed, ScienceDirect, SAGE	comorbidities (hypertension, cardiovascular disease, diabetes, cerebrovascular disease, respiratory disease, kidney disease, liver disease, cancer), age, sex, symptoms	severity, mortality	NOS	considered	critically low	[27]
Izcovich	28.04.20	PubMed/MEDLINE, CENTRAL, Embase	comorbidities (cerebrovascular disease, COPD, chronic kidney disease, cardiovascular disease,	severity, mortality	QUIPS	considered	critically low	[28]

			hypertension, diabetes, dementia, cancer), age, complications, sex, lab values, symptoms, vital signs					
Kahathuduwa	07.03.20	PubMed, Scopus, WoS	comorbidities (hypertension), age, lab values, symptoms	severity, mortality	NHLBI	unadjusted	critically low	29
Khan	01.05.20	MEDLINE, WoS, Scopus, CINAHL databases, MedRxiv, BioRxiv, SSRN	comorbidities (hypertension, cardiovascular disease, diabetes, immune and metabolic disorders, respiratory disease, cerebrovascular disease, cancer, renal disease, liver disease)	mortality of COVID-19 patients	NOS	unadjusted	critically low	30
Khunti	23.04.20	MEDLINE, Scopus, WHO	comorbidities (hypertension, diabetes, cardiovascular disease, COPD, chronic kidney disease, cancer)	severity, mortality	NOS	unadjusted	critically low	31
Kovalic	16.05.20	MEDLINE, PubMed, Embase, MedRxiv	comorbidities (chronic liver disease)	severity, mortality, ICU admission, intubation	nr	unadjusted	critically low	32
Kumar (a)	17.03.20	PubMed	comorbidities (hypertension, diabetes, cardiovascular disease, endocrine disease, gastrointestinal disease, COPD), age, sex, lab values, symptoms	severity	NIH	considered in quality assessment	critically low	33
Kumar (b)	22.04.20	PubMed	comorbidities (diabetes)	severity	NIH	considered in quality assessment	critically low	34
Li, B.	01.02.20	Embase, PubMed	comorbidities (hypertension, cardiovascular disease, cerebrovascular disease, diabetes), symptoms, age	severity	nr	unadjusted	critically low	35
Li, J.	06.04.20	PubMed, Embase, Scopus, The Cochrane Library, Chinese Medical Journal, BioRxiv, MedRxiv	comorbidities (diabetes, cancer, hypertension), age, sex, lab values, symptoms, treatment	severity, mortality, ICU admission, intubation	NOS	not considered	low	36
Li, X.	14.04.20	PubMed, Embase, WoS	comorbidities (cardiovascular disease, hypertension, acute cardiac injury)	in-hospital mortality	NOS	unadjusted	critically low	37
Lippi	26.03.20	Scopus, MEDLINE, WoS	comorbidities (hypertension), age	severity, mortality	nr	considered, recommend	critically low	38
Liu, H.	25.04.20	PubMed, MEDLINE (via Ovid), Embase, CDC, NIH	comorbidities (diabetes, cardiovascular disease, hypertension, COPD), age, symptoms	severity, mortality, ICU admission	NOS	not considered	critically low	39
Liu, N.	01.08.20	PubMed, Embase, WoS	comorbidities (dementia)	mortality*, other	NOS	not considered	critically low	40
Liu, Y.	31.07.20	PubMed, The Cochrane Library, Embase (via Ovid)	comorbidities (hypertension, COPD), sex, treatment, symptoms	mortality	NOS	considered, not included	low	41
Liu, Y.-F.	13.04.20	PubMed, MedRxiv, CNKI, Wanfang Data	comorbidities (chronic kidney disease), complications, lab values	severity	NOS	unadjusted	critically low	42
Luo	01.07.20	PubMed, Embase, WoS, The Cochrane Library	comorbidities (hypertension, cardiovascular diseases, COPD, chronic kidney disease, cancer), complications	severity, mortality	NOS	considered, not included	critically low	43
Malik	15.08.20	PubMed, WoS, Scopus, MedRxiv	comorbidities (obesity)	severity	NOS	considered, not included	critically low	44
Mantovani	15.05.20	PubMed, Scopus, WoS	comorbidities (diabetes)	severity, in-hospital mortality	NOS	considered, not pooled	critically low	45

Matsushita	03.04.20	PubMed, Embase	comorbidities (hypertension, diabetes, and cardiovascular disease), age, sex	severity	NOS	considered, pooled for CVD	critically low	46
Mehraeen	27.06.20	PubMed, Scopus, Embase, Google Scholar, WoS	comorbidities (hypertension, diabetes), age, countries' income, lab values, symptoms, vital signs	mortality	other	unadjusted	critically low	47
Mellor	26.08.20	Embase, MEDLINE, MedRxiv, Google Scholar	comorbidities (HIV), treatment	severity	JBI	considered, reported	critically low	48
Meng	05.07.20	PubMed, Cochrane, WoS, Wanfang Data, CNKI	comorbidities (cardiovascular disease, hypertension, diabetes), complications	severity	other	not considered	critically low	49
Mesas	27.07.20	MEDLINE, Scopus, WoS	comorbidities (kidney disease, hypertension, cancer, diabetes, pulmonary disease), age, sex, lab values, obesity, symptoms	in-hospital mortality	QUIPS	adjusted the pooled-OR for age, sex and health condition in the review	low	50
Momtazmanesh	21.04.20	PubMed, Embase	comorbidities (cardiovascular disease, hypertension, diabetes), complications, lab values	severity, mortality, ICU admission	NOS	not considered	critically low	51
Moula	18.05.20	PubMed	comorbidities (cardiovascular disease, coronary artery disease, hypertension, cerebrovascular disease, diabetes), age, sex	mortality	ROBINS-I	considered, not reported	critically low	52
Nandy	28.04.20	PubMed, CENTRAL	comorbidities (hypertension, diabetes, cardiovascular disease, COPD, chronic kidney disease)	severity	NOS	unadjusted	critically low	53
Noor	11.08.20	PubMed, ScienceDirect, Google Scholar	comorbidities (obesity, hypertension, diabetes, cardiovascular disease, cancer, cerebrovascular disease, COPD, coronary heart disease, chronic renal disease, chronic liver disease, chronic lung disease, chronic kidney disease), age, sex	mortality, ICU admission*	NOS	not considered	critically low	54
Ofori-Asenso	28.04.20	MEDLINE, Embase, ScienceDirect	comorbidities (cancer)	severity	NOS	not considered	critically low	55
Palaiodimos	10.05.20	MEDLINE, Embase, Google Scholar, MedRxiv	comorbidities (diabetes)	in-hospital mortality	QUIPS	considered, pooled unadjusted	critically low	56
Pan	06.07.20	PubMed, Embase, MEDLINE (via Ovid), MedRxiv	comorbidities (metabolic-associated fatty liver disease)	severity	NOS	unadjusted	critically low	57
Park (1)	01.07.20	PubMed, Embase, ASCO, ESMO, AACR, MedRxiv, BioRxiv	comorbidities, sex, treatment	mortality	NOS	adjusted, but treatment OR	critically low	58
Park (2)	01.06.20	PubMed, Embase, ASCO 2020 Virtual Annual Conference, AACR 2020 COVID-19, Cancer, ESMO conferences	comorbidities (cancer), sex	severity, mortality	NOS	considered, pooled for cancer	critically low	59
Parohan	01.05.20	WoS, PubMed, Scopus, The Cochrane Library, Google Scholar	comorbidities (hypertension, cardiovascular disease, diabetes, COPD, cancer), age, sex	mortality	NOS	adjusted	critically low	60
Parveen	31.03.20	PubMed, The Cochrane Library	comorbdities (diabetes, hypertension)	severity	NIH	unadjusted	critically low	61

Patel (a)	30.04.20	PubMed, WoS, Scopus	comorbidities (cerebrovascular disease)	severity, in-hospital mortality, ICU admission, intubation	NOS	adjusted in regression	critically low	62
Patel (b)	31.05.20	PubMed, WoS, Scopus, MedRxiv	comorbidities (cerebrovascular disease, cardiovascular disease, chronic liver disease), symptoms	severity, mortality, intubation, ICU admission*	NOS	age-adjusted meta-regression models in review	critically low	63
Popkin	15.07.20	PubMed, Google Scholar, MedRxiv, BioRxiv, Wanfang Data, CNKI, ICNARC and others	obesity	mortality, hospitalisation*, ICU admission	nr	considered	critically low	64
Pranata (1)	23.04.20	PubMed, SCOPUS, EuropePMC, CENTRAL	comorbidites (chronic kidney disease, renal replacement therapy)	severity	nr	not considered	critically low	65
Pranata (2)	01.04.20	PubMed, EuropePMC, SCOPUS, CENTRAL	comorbidities (COPD)	severity, mortality, ICU admission, other	nr	unadjusted	critically low	66
Pranata (3)	14.04.20	PubMed, SCOPUS, Europe PMC, CENTRAL, Google Scholar, Pre-Print Servers	comorbidities (hypertension, diabetes, cardiovascular disease, respiratory disease), sex	severity	NOS	unadjusted	critically low	67
Pranata (a)	07.04.20	PubMed, SCOPUS, EuropePMC, Google Scholar	comorbidities (hypertension), sex	mortality of COVID-19 patients	nr	not considered	critically low	68
Pranata (b)	nr	PubMed, Europe PMC, ProQuest, CENTRAL	comorbidities (obesity)	severity, mortality	NOS	considered, used	critically low	69
Rahman	18.04.20	PubMed, Google Scholar, Embase, The Cochrane Library	comorbidities (hypertension, diabetes), sex, symptoms	severity	nr	unadjusted	critically low	70
Roncon	25.03.20	MEDLINE, Scopus, WoS	comorbidities (diabetes, hypertension)	mortality, ICU admission	NOS	unadjusted	critically low	71
Sabatino	11.06.20	PubMed, Scopus, Google Scholar	comorbidities (cardiovascular disease), age, complications	mortality	QAT-OC/CSS	considered in quality assessment	critically low	72
Salunke	16.04.20	PubMed, CENTRAL	comorbidites (cancer)	mortality, ICU admission	NOS	not considered	critically low	73
Sanchez-Ramirez	15.04.20	PubMed, WoS, MEDLINE (via Ovid)	comorbidities (COPD), age, sex	severity	nr	not considered	critically low	74
Sepandi	23.03.20	MEDLINE/PubMed, Scopus, Google Scholar	comorbidities (diabetes, hypertension, kidney disease, respiratory disease, heart disease), age, sex	mortality	NOS	unadjusted	critically low	75
Shang	10.07.20	PubMed, WoS, MedRxiv, COVID-19 academic research communication platform	comorbidities (diabetes)	severity, mortality	NOS	not considered	critically low	76
Shi	29.04.20	PubMed, Embase, The Cochrane Library, three electronic Chinese databases	comorbidities (chronic kidney disease, respiratory disease, cardio-cerebrovascular disease), age, complications, sex, lab values, treatment, symptoms, vital signs	mortality	QUIPS	considered, pooled	critically low	77

Singh	29.07.20	PubMed, Embase	comorbidities (inflammatory bowel disease)	mortality, hospitalisation*, ICU admission, other	JBI	not considered	critically low	78
Soeroto	28.07.20	PubMed, Embase	comorbidities (diabetes, hypertension), age, sex	severity	nr	not considered	critically low	79
Sreenivasan	30.03.20	MEDLINE, Scopus, Embase	comorbidities (cardiovascular disease, heart failure, hypertension, diabetes), sex, lab values, vital signs	severity, mortality, intubation	nr	not considered	critically low	80
Ssentongo	07.07.20	MEDLINE, SCOPUS, OVID, The Cochrane Library, MedRxiv	comorbidities (hypertension, heart failure, diabetes, chronic kidney disease, cancer, cardiovascular disease)	mortality	NOS	considered	critically low	81
Su	03.05.20	PubMed, Embase, MedRxiv	comorbidities (cancer, hypertension, diabetes, respiratory disease)	mortality, ICU admission	JBI	not considered	critically low	82
Sze	31.08.20	MEDLINE, Embase, PROSPERO, The Cochrane Library, MedRxiv	ethnicity	mortality, ICU admission	JBI	adjusted	critically low	83
Tabrizi	12.03.20	PubMed, Scopus, Embase, WoS, Google Scholar	comorbidities (COPD, diabetes, hypertension, cardiovascular disease, chronic kidney disease), symptoms	severity	NOS	not considered	critically low	84
Tamara	14.04.20	Cochrane, MEDLINE, Embase, PubMed	comorbidities (obesity)	severity	NOS	recommend adjustment	critically low	85
Tamuzi	01.07.20	CENTRAL, PubMed, MedRxiv, Google Scholar, Clinical Trials Registry databases	comorbidities (HIV, tuberculosis)	severity, mortality	NOS	not considered	critically low	86
Tavan	nr	Scopus, PubMed, Cochrane, WoS, Google Scholar	comorbidities (hypertension, diabetes, cardiovascular disease), symptoms	mortality	STROBE	unadjusted	critically low	87
Tian, W.	24.04.20	PubMed, Google Scholar, WoS, CNKI	comorbidities (hypertension, diabetes, coronary heart disease), lab values	in-hospital mortality	AHRQ	unadjusted	critically low	88
Tian, Y. (1)	23.04.20	PubMed, Elsevier, WoS, CNKI, Wanfang Data, VIP	comorbidities (cancer)	severity, mortality	nr	adjusted prevalence	critically low	89
Tian, Y. (2)	30.06.20	Embase, PubMed	comorbidities (hypertension, cardiovascular disease, diabetes, respiratory disease, cancer)	severity	nr	not considered	low	90
Toraih	08.05.20	WoS, PubMed	comorbidities (cardiovascular disease, chronic heart disease, COPD, hypertension, diabetes, cancer), age, complications, lab values, treatment	severity	nr	suggest confounding of AST	critically low	91
Varikasuvu	15.05.20	Scopus, ScienceDirect	comorbidities (diabetes)	severity, mortality	NOS	unadjusted	critically low	92

Venkatesulu	23.05.20	PubMed/MEDLINE, Embase, Cochrane Central, Google Scholar, MedRxiv	comorbidities (cancer with subtypes)	mortality, ICU admission, intubation	nr	not considered	critically low	93
Vijenthira	20.08.20	PubMed, Embase	comorbidities (cancer), age, treatment	mortality, ICU admission, intubation	JBI	unadjusted	critically low	94
Villalobos	29.04.20	MEDLINE, bioRXiv, MedRxiv, national (public) health instititutions	comorbidities (cerebrovascular disease, cardiovascular disease, hypertension, renal disease), healthcare workers	severity, mortality, hospitalisation*	ROBINS-I	not considered	critically low	95
Wang, B.	01.03.20	PubMed, The Cochrane Library, Embase and others	comorbidities (hypertension, diabetes, COPD, cardiovascular disease, cerebrovascular disease)	severity, ICU admission, other	NOS	unadjusted	critically low	96
Wang, X.	06.04.20	PubMed, Embase, WoS, MedRxiv, BioRxiv	comorbidities (hypertension, cardiovascular disease, chronic kidney disease, diabetes), complications	severity	nr	unadjusted	critically low	97
Wang, Y. (1)	05.08.20	PubMed, Embase, The Cochrane Library, WoS , CNKI	comorbidities (asthma)	severity, mortality	NOS	unadjusted	critically low	98
Wang, Y. (2)	15.06.20	PubMed, Scopus, Embase, MedRxiv, Google Scholar	comorbidities (asthma)	mortality	Ro-BANS	not considered	critically low	99
Wang, Z.	16.03.20	PubMed, WoS, The Cochrane Library, CBM, CNKI, Wanfang Data, VIP	comorbidities (hypertension, diabetes, cardiovascular disease), age, complications, sex, symptoms	severity, mortality	NOS	unadjusted	critically low	100
Wingert	12.06.20	MEDLINE (via Ovid), Epistemonikos COVID-19 in L·OVE Platform, McMaster COVID-19 Evidence Alerts, selected websites	comorbidities (liver disease, obesity, diabetes, heart failure, chronic kidney disease, dementia), age, ethnicity, homelessness, income	severity, mortality, hospitalisation*, ICU admission, intubation	other	considered, not pooled	low	101
Wu, J.	09.03.20	Medical Records from Huai'an Fourth People's Hospital	comorbidities (diabetes), age, sex	severity	nr	unadjusted	critically low	102
Wu, Z. H.	14.04.20	MEDLINE/ PubMed, Embase, WoS	comorbidities (diabetes)	mortality	NOS	adjusted	critically low	103
Xu, J.	10.08.20	PubMed, WoS, Embase	comorbidities (cerebrovascular disease)	severity, mortality	NOS	considered	critically low	104
Xu, L.	23.02.20	CNKI, Wanfang Data, Weipu Database, CBM, PubMed, Embase, CENTRAL, WoS	comorbidities (hypertension, diabetes, cardiovascular disease, COPD), age, sex, lab values, symptoms, vital signs	severity	AHRQ	unadjusted	critically low	105
Yang, J. (1)	22.04.20	PubMed, Embase, WOS, Cochrane, CNKI, Wanfang, Sinomed	obesity	severity	NOS	not considered	critically low	106

Yang, J. (2)	27.07.20	PubMed, Embase, WoS	obesity	hospitalisation*, in-hospital mortality, ICU admission, intubation, other	NOS	considered; not in meta-analysis	critically low	107
Yang, J. (3)	25.02.20	PubMed, Embase, WoS	comorbidities (hypertension, respiratory disease, cardiovascular disease)	severity	none	unadjusted	critically low	108
Yang, S.	02.04.20	PubMed, WoS, BioRxiv, MedRxiv, CNKI	comorbidities (coronary heart disease, cardiovascular disease, cerebrovascular disease, lung disease, cancer, hypertension, heart disease, chronic kidney disease, diabetes), age, complications, sex, lab values, symptoms, vital signs	mortality	NOS	unadjusted	critically low	109
Yekedüz	01.09.20	MEDLINE	comorbidities (cancer)	severity, mortality	NOS	considered, reported in figure	critically low	110
Yonas	nr	PubMed, EuropePMC, SCOPUS, CENTRAL, MedRxiv	comorbidities (heart failure)	severity, mortality	none	not considered	critically low	111
Youseef	16.04.20	PubMed, WoS, Scopus, ScienceDirect	comorbidities (hypertension, chronic kidney disease, diabetes, chronic liver disease, cardiovascular disease, cerebrovascular disease, cancer), complications, lab values, treatment	severity, mortality	NOS	unadjusted	critically low	112
Yu	25.07.20	PubMed, WoS, CNKI	comorbidities (cardio-cerebrovascular disease)	severity, mortality	NOS	not considered	critically low	113
Zaki	20.05.20	Europe PMC, Google Scholar, PubMed	comorbidities (COPD, cardiovascular disease, cancer, kidney disease, coronary heart disease), symptoms	severity	nr	unadjusted	critically low	114
Zhang, H.	09.06.20	PubMed, Embase, conference proceedings	comorbidities (cancer, hypertension, diabetes), age	severity, mortality	NOS	not considered	critically low	115
Zhang, J.	20.03.20	PubMed, ScienceDirect, WoS, Wiley Online Library, Embase, CNKI	comorbidities (hypertension), age	severity, mortality	NOS	not considered	critically low	116
Zhao	22.03.20	PubMed, WoS, Cochrane, WanFang Data, CNKI	comorbidities (COPD)	severity	nr	unadjusted	critically low	117
Zheng	20.03.20	PubMed, Embase, WoS, CNKI	comorbidities (hypertension, diabetes, cardiovascular disease, respiratory disease), age, sex, lab values, symptoms	severity, other	MINORS	unadjusted	critically low	118
Zhou	25.04.20	PubMed, Embase, The Cochrane Library	comorbidities (obesity, hypertension, diabetes, cardiovascular disease, respiratory disease,	severity, mortality	nr	considered in limitations	critically low	119

			cerebrovascular disease, cancer, chronic kidney disease, liver disease)					
Zuin	01.07.20	The Cochrane Library, Embase, PubMed, Google Scholar	comorbidities (dementia)	mortality	NOS	considered, age as moderator	critically low	[120]

*of COVID-19 patients
AHRQ, Agency for Healthcare Research and Quality; CBM, Chinese Biomedical Literature Database; CDC, Centers for Disease Control and Prevention; CENTRAL, Cochrane Central Register of Controlled Trials; CNKI, China National Knowledge Infrastructure; COPD, chronic obstructive pulmonary disease; JBI, Critical appraisal tools by the Joanna Briggs Institute; MINORS, Methodological Index for Non-Randomized Studies; NHI, National Institutes of Health; NIH/NHLBI, Quality Assessment Tools; NOS, Newcastle Ottawa Scale; nr, not reported; QAT-OC/CSS, Quality Assessment Tool for Observational and Cross-Sectional Studies; QUIPS, Quality In Prognosis Studies; ROBINS-I, Risk Of Bias In Non-randomized Studies of Interventions; SAGE, Strategic Advisory Group of Experts on Immunization; WHO, World Health Organization; WoK, Web of Knowledge; WoS, Web of Science

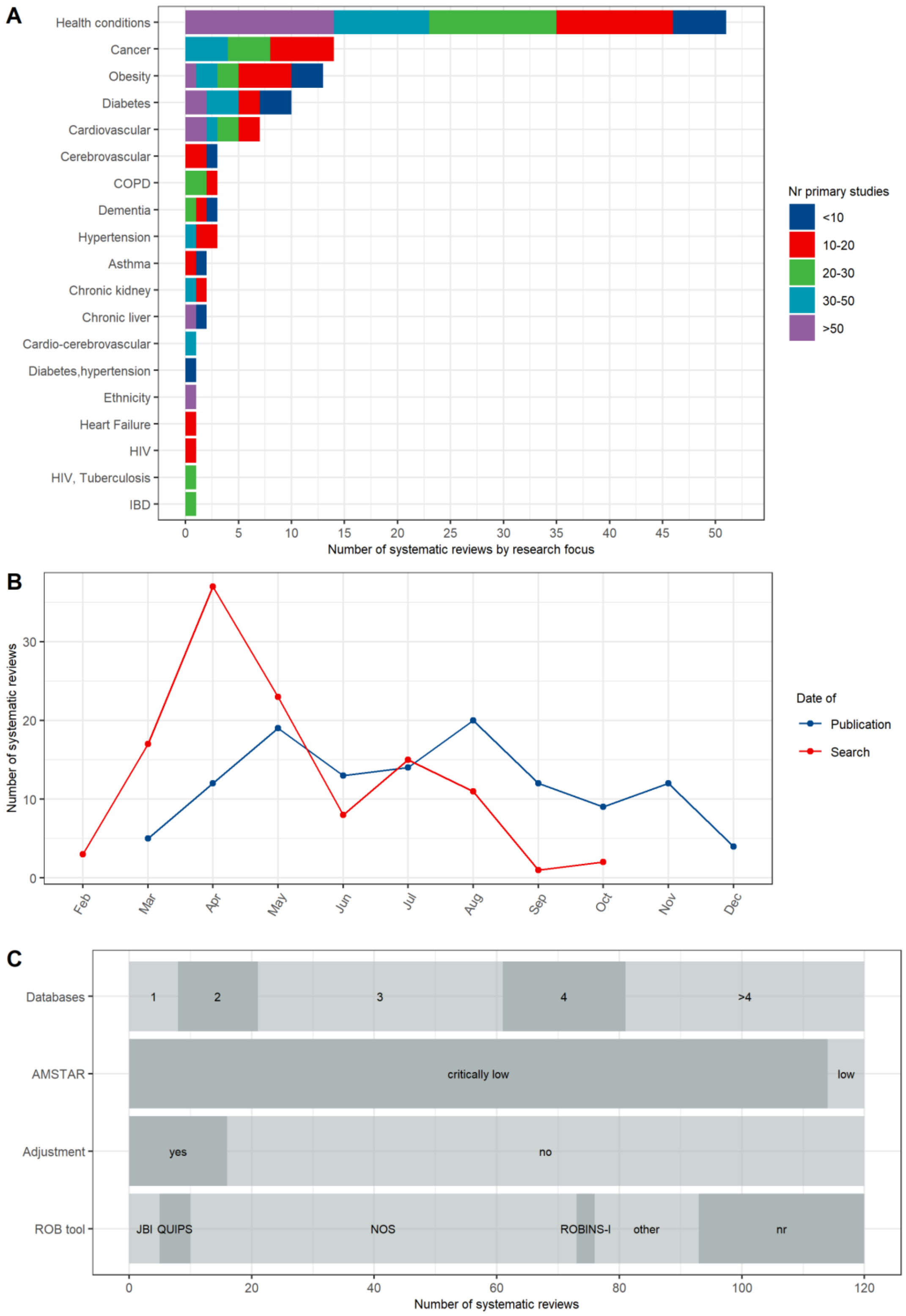

Figure 3. Overview of included systematic reviews (SRs).

(A) Number of SRs by research focus (“health conditions” denotes different pre-existing health conditions) and number of included primary studies. (B) Number of SRs by date of last search (in red), and date either of publication or pre-print availability (in blue). (C) Methodological characteristics of included SRs: number of searched databases, results of the evaluation with AMSTAR-2, presentation of age-adjusted risk estimates, and risk of bias (ROB) tool (nr, not reported)

Table 2 Results of quality evaluation of the included systematic reviews

Item1. Did the report of the review contain an explicit statement that the review methods were established prior to the conduct of the review and did the report justify any significant deviations from the protocol?
Item2. Did the review authors use a comprehensive literature search strategy?
Item3. Did the review authors provide a list of excluded studies and justify the exclusions?
Item4.Did the review authors use a satisfactory technique for assessing the risk of bias (RoB) in individual studies that were included in the review?
Item5. If meta-analysis was performed, did the review authors use appropriate methods for statistical combination of results?
Item6. If they performed quantitative synthesis did the review authors carry out an adequate investigation of publication bias (small study bias) and discuss its likely impact on the results of the review?
Item7. If they performed quantitative synthesis did the review authors carry out an adequate investigation of publication bias (small study bias) and discuss its likely impact on the results of the review?

Author	Item 1	Item 2	Item 3	Item 4	Item 5	Item 6	Item 7	Quality rating	Citation
Aggarwal	yes	partially yes	yes	yes	no	no	yes	critically low	1
Awortwe	no	no	no	-	-	-	-	critically low	2
Bellou	no	no	no	no	yes	yes	no	critically low	3
Biswas (1)	no	partially yes	no	partially yes	-	-	yes	critically low	4
Biswas (2)	no	no	no	-	-	-	-	critically low	5
Chang	no	partially yes	no	yes	yes	yes	yes	critically low	6
Chen	no	no	no	yes	-	-	yes	critically low	7
Cheruiyot	no	partially yes	no	-	-	-	-	critically low	8
Chidambaram	no	no	no	yes	yes	yes	yes	critically low	9
Chu	no	-	no	-	-	-	-	critically low	10
Das	no	-	no	-	-	-	-	critically low	11
de Almeida-Pititto	partially yes	partially yes	no	no	yes	no	yes	critically low	12
Degarege	-	no	no	-	-	-	-	critically low	13
Dorjee	no	-	no	-	-	-	-	critically low	14
Du	no	no	no	yes	yes	no	yes	critically low	15
Elgohary	no	partially yes	no	no	yes	no	no	critically low	16
Figliozzi	partially yes	partially yes	no	yes	no	yes	yes	critically low	17
Florez-Perdomo	no	partially yes	no	yes	-	yes	yes	critically low	18
Földi	yes	partially yes	no	yes	no	no	yes	critically low	19
Gao	yes	no	no	yes	-	-	-	critically low	20
Giannakoulis	yes	no	no	yes	yes	no	no	critically low	21
Guo	no	partially yes	no	no	-	no	yes	critically low	22
Hariyanto	no	no	-	-	-	-	-	critically low	23
Hessami	no	partially yes	no	-	-	-	-	critically low	24
Huang	no	partially yes	yes	yes	yes	yes	yes	low	25
Hussain	no	no	no	yes	no	no	no	critically low	26
Islam	no	-	no	-	-	-	-	critically low	27
Izkovich	partially yes	partially yes	no	partially yes	no	-	no	critically low	28
Kahathuduwa	no	partially yes	no	partially yes	yes	-	-	critically low	29

Khan	no	no	no	yes	yes	yes	yes	critically low	30
Khunti	no	-	no	-	-	-	-	critically low	31
Kovalic	no	-	no	no	-	-	-	critically low	32
Kumar (a)	partially yes	no	no	yes	yes	no	yes	critically low	33
Kumar (b)	no	no	-	-	-	-	-	critically low	34
Li, B.	no	-	no	no	-	-	-	critically low	35
Li, J	yes	partially yes	no	yes	-	yes	yes	low	36
Li, X.	no	-	no	-	-	-	-	critically low	37
Lippi	no	-	no	no	-	-	yes	critically low	38
Liu, H.	no	partially yes	no	yes	yes	yes	yes	critically low	39
Liu, N.	no	-	no	-	-	-	-	critically low	40
Liu, Y.	yes	partially yes	no	yes	yes	yes	yes	low	41
Liu, Y.-F.	no	-	no	-	-	-	-	critically low	42
Luo	no	no	-	-	-	-	-	critically low	43
Malik	no	-	no	-	-	-	-	critically low	44
Mantovani	no	no	-	-	-	-	-	critically low	45
Matsushita	-	no	no	-	-	-	-	critically low	46
Mehraeen	no	no	-	-	-	-	-	critically low	47
Mellor	no	-	no	-	-	-	-	critically low	48
Meng	no	-	no	-	-	-	-	critically low	49
Mesas	yes	partially yes	no	yes	yes	yes	yes	low	50
Momtazmanesh	no	partially yes	no	yes	-	yes	yes	critically low	51
Moula	no	no	no	yes	yes	yes	yes	critically low	52
Nandy	no	partially yes	no	no	yes	-	no	critically low	53
Noor	no	no	no	yes	yes	yes	yes	critically low	54
Ofori-Asenso	no	partially yes	no	no	-	-	-	critically low	55
Palaiodimos	no	partially yes	no	yes	no	yes	yes	critically low	56
Pan	no	-	no	-	-	-	-	critically low	57
Park (1)	no	no	no	no	yes	yes	yes	critically low	58
Park (2)	no	no	no	yes	yes	yes	yes	critically low	59
Parohan	no	partially yes	yes	no	yes	yes	yes	critically low	60
Parveen	no	no	no	yes	-	-	no	critically low	61
Patel (a)	no	no	-	-	-	-	-	critically low	62
Patel (b)	no	-	no	-	-	-	-	critically low	63
Popkin	no	partially yes	no	yes	no	yes	no	critically low	64
Pranata (1)	no	no	-	-	-	-	-	critically low	65
Pranata (2)	no	no	no	yes	no	no	yes	critically low	66

Pranata (3)	no	no	-	-	-	-	-	critically low	67
Pranata (a)	no	-	no	-	-	-	-	critically low	68
Pranata (b)	no	no	-	-	-	-	-	critically low	69
Rahman	no	no	-	-	-	-	-	critically low	70
Roncon	no	no	-	-	-	-	-	critically low	71
Sabatino	no	no	no	-	-	-	-	critically low	72
Salunke	no	partially yes	no	no	yes	-	yes	critically low	73
Sanchez-Ramirez	no	-	no	-	-	-	-	critically low	74
Sepandi	no	-	no	-	-	-	-	critically low	75
Shang	no	no	no	yes	yes	yes	yes	critically low	76
Shi	no	partially yes	no	-	-	-	-	critically low	77
Singh	yes	no	no	no	yes	yes	yes	critically low	78
Soeroto	no	no	no	no	no	no	yes	critically low	79
Sreenivasan	yes	partially yes	no	no	-	-	-	critically low	80
Ssentongo	yes	partially yes	no	yes	yes	yes	yes	critically low	81
Su	no	-	no	-	-	-	-	critically low	82
Sze	yes	no	no	-	-	-	-	critically low	83
Tabrizi	no	no	-	-	-	-	-	critically low	84
Tamara	yes	no	no	yes	-	-	-	critically low	85
Tamuzi	yes	no	yes	-	-	-	yes	critically low	86
Tavan	no	partially yes	no	-	-	-	yes	critically low	87
Tian, W.	no	-	no	-	-	-	-	critically low	88
Tian, Y. (1)	no	no	-	-	-	-	-	critically low	89
Tian, Y. (2)	yes	yes	no	yes	yes	yes	yes	low	90
Toraih	no	-	no	-	-	-	-	critically low	91
Varikasuvu	yes	partially yes	no	yes	no	no	yes	critically low	92
Venkatesulu	yes	partially yes	no	no	-	-	-	critically low	93
Vijenthira	no	partially yes	no	-	-	-	-	critically low	94
Villalobos	yes	no	no	yes	no	no	no	critically low	95
Wang, B.	no	-	no	-	-	-	-	critically low	96
Wang, X.	no	-	no	-	-	-	-	critically low	97
Wang, Y. (1)	yes	-	no	yes	-	-	no	critically low	98
Wang, Y. (2)	no	no	no	-	-	-	-	critically low	99
Wang, Z.	yes	no	no	-	-	-	-	critically low	100
Wingert	yes	no	yes	partially yes	NA	yes	NA	low	101
Wu, J.	no	no	no	-	-	-	-	critically low	102
Wu, Z. H.	no	-	no	-	-	-	-	critically low	103
Xu, J.	no	partially yes	no	-	-	-	-	critically low	104

Xu, L.	no	-	no	-	-	-	-	critically low	105
Yang, J. (1)	no	no	-	-	-	-	-	critically low	106
Yang, J. (2)	yes	no	no	-	-	-	-	critically low	107
Yang, J. (3)	no	no	no	-	-	-	-	critically low	108
Yang, S.	no	-	no	-	-	-	-	critically low	109
Yekedüz	no	no	no	-	-	-	-	critically low	110
Yonas	no	partially yes	no	-	-	-	-	critically low	111
Youssef	no	-	no	-	-	-	-	critically low	112
Yu	no	partially yes	no	yes	yes	yes	yes	critically low	113
Zaki	-	-	no	no	-	-	-	critically low	114
Zhang, H.	no	-	no	-	-	-	-	critically low	115
Zhang, J.	no	no	no	-	-	-	-	critically low	116
Zhao	no	-	no	-	-	-	-	critically low	117
Zheng	no	-	no	-	-	-	-	critically low	118
Zhou	no	partially yes	no	no	no	yes	yes	critically low	119
Zuin	no	no	-	-	-	-	-	critically low	120

- , not rated; NA, not applicable

Table 3 Overview of included primary studies (n=160)

Study	Study design; setting, number of hospitals; period; Study population	Sample size; age (median (IQR), mean (SD or CI) or % in certain age group(s)); % of males	Given definition of COVID-19/ pre-existing condition (if yes, see section 2.3 for detail)	Outcomes relevant for review	Adjustment method (Regression Model) / Covariates / Reference age and sex category	Risk of bias	Analyses the study participated in [Main meta-analyses reported as: number of the resulted effect estimates the study contributed to; a meta-analysis is described as Condition-Outcome-Measure of association-Region-Total number of studies in the analysis-I^2%]
**African Region**							
Boulle; South Africa[121]	cohort; multi-center; 1. Mar - 9. Jun; individuals utilizing public sector health services diagnosed with COVID-19	22308; SARS-CoV-2 cases: 9.6%>60yo, Hospitalized: 61%>60yo; 38%	PCR / yes, summary of evidence used to infer different comorbidities	case mortality, hospital mortality	Cox / all models: age, sex, location and comorbidities (DM, HTN, CKD, Resp, HIV, tuberculosis (previous,current)) / age 20-39/ female	JBI= 11	12 estimates Diabetes-Case mortality-HR-AFR-1-0 // Hypertension-Case mortality-HR-AFR-1-0 // Chronic kidney disease-Case mortality-HR-AFR-1-0 // Respiratory disease-Case mortality-HR-AFR-1-0 // Tuberculosis-Case mortality-HR-AFR-1-0 // HIV-Case mortality-HR-AFR-1-0 // Diabetes-Hospital mortality-HR-AFR-1-0 // Hypertension-Hospital mortality-HR-AFR-1-0 // Chronic kidney disease-Hospital mortality-HR-AFR-1-0 // Respiratory disease-Hospital mortality-HR-AFR-1-0 // Tuberculosis-Hospital mortality-HR-AFR-1-0.3 // HIV-Hospital mortality-HR-AFR-1-0
**Eastern Mediterranean Region**							
Al-Sabah; Kuwait[122]	cohort; single-center; 24. Feb - 7. Apr; hospitalised individuals diagnosed with COVID-19	1158; median 40 (31-52), nr (nr); 82%	PCR / yes, obesity categories described, definition for HTN and DM provided	ICU admission	Log / model 1 ("obesity model"): age, sex, obesity and overweight, comorbidities (HTN); model 2 ("diabetes model"): age, sex, comorbidities (DM, HTN) / na	NOS =9	5 estimates: Overweight-ICU admission-OR-EMR-1-0 // Obesity/BMI>30-ICU admission-OR-EMR-1-0 // Obesity/BMI>40-ICU admission-OR-EMR-1-0 // Diabetes-ICU admission-OR-EMR-1-0 // Hypertension-ICU admission-OR-EMR-1-0
Rastad; Iran[123]	cohort; multi-center; 20. Feb - 25. Mar; individuals hospitalised with COVID-19 diagnosis	2957; nr (nr), mean 55 (17); 54%	PCR / no	in-hospital mortality	Log / model 1: comorbidities (DM, CVD, other comorbidities); model 2: sex, comorbidities (DM, CVD, other comorbidities), model 3: sex, age, comorbidities (DM, CVD, other comorbidities), laboratory values; different models estimated for age groups <65 and >=65: one of the following comorbidities included in the models: DM (with or without other comorbidities), CVD (with or without other comorbidities), DM as single disease, CVD as single disease, CVD or DM, age, sex / age>65 years / female	QUIPS= low	2 estimates Diabetes-Hospital mortality-OR-EMR-1-0 // Cardiovascular disease-Hospital mortality-OR-EMR-1-0 and Analysis of age-stratified estimates
**European Region**							
Al-Salameh; France[124]	cohort; single-center; nr - 21. Apr; follow-up until 1. May ; individuals diagnosed with COVID-19 hospitalised/admitted to ICU	total n=433, n= 326 (model admission ICU); n=370 (model in-hospital mortality); median 72 (nr), nr (nr); 55%	PCR / no	ICU admission, in-hospital mortality (both ICU/non-ICU deaths)	Cox, Log / model ICU admission: age, sex, BMI and comorbidities (DM), laboratory values; model in-hospital mortality: age, sex, laboratory values, comorbidities (DM, CVD) / age numeric / sex na	NOS =6	3 estimates: Cardiovascular disease-Hospital mortality-HR-EUR-2-0 // Diabetes-Hospital mortality-HR-EUR-9-49.7 // Diabetes-ICU admission-OR-EUR-2-0
Amit; Israel[125]	case series; multi-center, 13; 5. Mar - 27. April; individuals diagnosed with COVID-19 in ICU	156; median 72 (60-82), nr (nr); 69%	PCR / no	ICU mortality	Log / age, sex, comorbidities (HTN, DM, IHD), complications, medication, laboratory values, ICU stay / age numeric / male	other = low	The outcome excluded from the reporting
Bellan; Italy[126]	cohort; multi-center, 3; 1. Mar - 28. Apr; individuals hospitalised with COVID-19 diagnosis	1697; median 71 (58-80), nr (nr); 59%	PCR / no	in-hospital mortality	Log / age, CANCact, obesity, smoking / age <58yo /sex na	NOS =8	2 estimates Cancer/Active-Hospital mortality-OR-EUR-1-0 // Obesity/BMI>30-Hospital mortality-OR-EUR-3-75.5

Study	Study design; setting, number of hospitals; period;<br>Study population	Sample size; age (median (IQR), mean (SD or CI) or % in certain age group(s)); % of males	Given definition of COVID-19/ pre-existing condition (if yes, see section 2.3 for detail)	Outcomes relevant for review	Adjustment method (Regression Model) / Covariates / Reference age and sex category	Risk of bias	Analyses the study participated in<br>[Main meta-analyses reported as: number of the resulted effect estimates the study contributed to; a meta-analysis is described as Condition-Outcome-Measure of association-Region-Total number of studies in the analysis-I^2%]
Berenguer; Spain[127]	cohort; multi-center, 127; nr - 17. Mar; follow-up until 17. Apr;<br>individuals hospitalised with COVID-19 diagnosis	4035; median 70 (56-80), nr (nr); 0,61	PCR / yes, partially, definitions for CANC, BMI provided	in-hospital mortality	Cox / age, sex, comorbidities (HTN, obesity, CLD/cirrhosis, chronic neurological disorder, active CANC, DEM), symptoms, laboratory values / age 0-49 / female	NOS =7	6 estimates<br>Cancer/Active-Hospital mortality-HR-EUR-3-25.6 // Neurological disease-Hospital mortality-HR-EUR-2-0 // Chronic liver/Cirrhosis-Hospital mortality-HR-EUR-1-0 // Dementia-Hospital mortality-HR-EUR-3-75.1 // Hypertension-Hospital mortality-HR-EUR-8-58 // Obesity/BMI>30-Hospital mortality-HR-EUR-6-89.2
Bezzio; Italy[128]	cohort; multi-center, 24; 11. Mar - 29. Mar; individuals with inflammatory bowel disease with diagnosed COVID-19	79; median 45 (18-80), nr (nr); 0,66	PCR / yes, definition for IBD provided, use of CCI	case mortality	Log / age, CCI score, active IBD, UC, corticosteroids, antitumor necrosis factor / age <61 / sex na	NOS =8	Analysis of evidence for specific population groups - individuals with inflammatory bowel
Bianchetti; Italy[129]	cohort; single-center; 22. Feb - 8. Apr;<br>individuals hospitalised with COVID-19 diagnosis	627; nr (nr), mean 71 (13); 47%	PCR / yes, partially, some information on the identification of dementia	in-hospital mortality	Log / age, sex, comorbidities (DEM) / age 1-year increase / male	NOS =7	1 estimate<br>Dementia-Hospital mortality-OR-EUR-4-49.7
Borghesi; Italy[130]	cohort; single-center; 4. Mar - 24. Mar;<br>individuals hospitalised with COVID-19 diagnosis	302; median 67 (57-77), nr (nr); 64%	PCR / no	in-hospital mortality	Log / Brixia score, age, comorbidities (immun) / na	NOS =7	1 estimate<br>Immunosuppression-Hospital mortality-OR-EUR-1-0
Burn; Spain[131] pre-print	cohort; electronic database; 1. Mar - 6. May;<br>individuals with COVID-19 diagnosis as recorded in primary care records covering >80% of the population in Catalonia, Spain	model diagnosed to death (case mortality):2794 hospitalised to death (In-hospital mortality):2791; diagnosed to hospitalisation (hospitalisation): 9437; median 72 (58-81), nr (nr); 56%	PCR / yes, partially, BMI; CCI was used, information on obesity and autoimmune condition	hospitalisation, in-hospital mortality, case mortality	Cox / age, sex, inclusion of 1 comorbidity or number of comorbidities (comorbidities: Auto, CKD, COPD, DEM, cardiac diseases, hyperlipidemia, HTN, CANC, obesity, DM, number of Charlson comorbidities 1:0, 2:0,3+:0) / na	NOS =9	30 estimates<br>Diabetes-Hospital mortality-HR-EUR-9-49.7 // Dementia-Hospital mortality-HR-EUR-3-75.1 // Hypertension-Hospital mortality-HR-EUR-8-58 // Obesity/BMI>30-Hospital mortality-HR-EUR-6-89.2 // Autoimmune condition-Hospital mortality-HR-EUR-1-0 // Chronic kidney disease-Hospital mortality-HR-EUR-9-94.5 // COPD-Hospital mortality-HR-EUR-1-0 // Heart disease-Hospital mortality-HR-EUR-2-33.8 // Dyslipidemia or hyperlipidemia-Hospital mortality-HR-EUR-1-0 // Cancer-Hospital mortality-HR-EUR-3-32.1 // Autoimmune condition-Hospitalisation-HR-EUR-1-0 // Chronic kidney disease-Hospitalisation-HR-EUR-2-76.3 // COPD-Hospitalisation-HR-EUR-2-94.1 // Dementia-Hospitalisation-HR-EUR-2-93.4 // Diabetes-Hospitalisation-HR-EUR-2-44.2 // Heart disease-Hospitalisation-HR-EUR-1-0 // Hypertension-Hospitalisation-HR-EUR-2-74.7 // Dyslipidemia or hyperlipidemia-Hospitalisation-HR-EUR-2-37.5 // Cancer-Hospitalisation-HR-EUR-2-80.1 // Obesity/BMI>30-Hospitalisation-HR-EUR-2-0 // Autoimmune condition-Case mortality-HR-EUR-1-0 // Chronic kidney disease-Case mortality-HR-EUR-2-0 // COPD-Case mortality-HR-EUR-2-0 // Dementia-Case mortality-HR-EUR-2-0 // Diabetes-Case mortality-HR-EUR-2-16.8 // Heart disease-Case mortality-HR-EUR-1-0 // Hypertension-Case mortality-HR-EUR-2-91.9 // Dyslipidemia or hyperlipidemia-Case mortality-HR-EUR-2-48.6 // Cancer-Case mortality-HR-EUR-2-0 // Obesity/BMI>30-Case mortality-HR-EUR-2-0
Busetto; Italy[132]	cohort; single-center; 23. Mar - 11. Apr;<br>individuals hospitalised with COVID-19 diagnosis and severe acute respiratory syndrome	92; nr (nr), mean 70 (13); 62%	PCR / yes, partially, only on obesity	in-hospital mortality	Log / age, sex, BMI, comorbidities (Resp, DM, DEM) / age numeric / male	NOS =6	3 estimates<br>Dementia-Hospital mortality-OR-EUR-4-49.7 // Diabetes-Hospital mortality-OR-EUR-5-19.6 // Respiratory disease-Hospital mortality-OR-EUR-2-30.7
Cariou; France[133]	cohort; multi-center, 53; 10. Mar - 31. Mar;<br>individuals with diabetes hospitalised with COVID-19 diagnosis	1317; nr (nr), mean 70 (13); 65%	PCR / no	in-hospital mortality on day 7, intubation and/or death	Log / age, sex, comorbidities (HTN, CHF, CANCact), complications, treatment / age 1SD / male	NOS =8	Analysis of evidence for specific population groups - diabetes
Carter; UK, Italy[134]	cohort; multi-center, 10; 27. Feb - 28. Apr;<br>individuals hospitalised with COVID-19 diagnosis	1564; median 74 (61-83), nr (nr); 58%	PCR (95.1%) /clinical diagnosis (4.9%) / no	in-hospital mortality	Cox / age, sex, comorbidities (DM, CAD, HTN, reduced renal function) smoking, location of infection aquisition, laboratory values, Clinical Frailty Scale / age<65 / female	other = low	4 estimates<br>Diabetes-Hospital mortality-HR-EUR-9-49.7 // Hypertension-Hospital mortality-HR-EUR-8-58 // Chronic kidney disease-Hospital mortality-HR-EUR-9-94.5 // Coronary artery disease-Hospital mortality-HR-EUR-4-83.3

Study	Study design; setting, number of hospitals; period; Study population	Sample size; age (median (IQR), mean (SD or CI) or % in certain age group(s)); % of males	Given definition of COVID-19/ pre-existing condition (if yes, see section 2.3 for detail)	Outcomes relevant for review	Adjustment method (Regression Model) / Covariates / Reference age and sex category	Risk of bias	Analyses the study participated in [Main meta-analyses reported as: number of the resulted effect estimates the study contributed to; a meta-analysis is described as Condition-Outcome-Measure of association-Region-Total number of studies in the analysis-I^2%]
Caussy; France[135]	cohort; single-center; nr -27. Mar; individuals hospitalised with COVID-19 diagnosis	340; 68%>=65yrs; 58%	PCR / no	ICU admission vs non-ICU ward	Log / age, sex, obesity / na	NOS >=3	1 estimate<br>Obesity/BMI>30-ICU admission-OR-EUR-2-56.2
Ciardullo; Italy[136]	cohort; single-center; 22. Feb - 15. May; individuals hospitalised with COVID-19 diagnosis	339; nr (nr), mean 72 (14); 65%	PCR / no	in-hospital mortality	Log / age, sex, comorbidities (HTN, CKD, CVD, COPD), another model with additional laboratory values / age 1-year increase / male	NOS >=5 - <=8	5 estimates<br>Chronic kidney disease-Hospital mortality-RR-EUR-1-0 // COPD-Hospital mortality-RR-EUR-1-0 // Cardiovascular disease-Hospital mortality-RR-EUR-1-0 // Diabetes-Hospital mortality-RR-EUR-1-0 // Hypertension-Hospital mortality-RR-EUR-1-0
Ciceri; Italy[137]	cohort; single-center; 25. Feb - 24. Mar; individuals hospitalised with COVID-19 diagnosis	410; median 65 (56-75), nr (nr); 73%	PCR / no	in-hospital mortality	Cox / age, sex, ethnicity, presence of comorbidity, BMI, comorbidities (HTN, DM, CAD, CKD, CANCact), laboratory values radiographic assessment of lung Edema (RALE) score / age <65 / female	NOS =8	2 estimates<br>Cancer/Active-Hospital mortality-HR-EUR-3-25.6 // Coronary artery disease-Hospital mortality-HR-EUR-4-83.3
Conversano; Italy[138]	cohort; single-center; 27. Feb - 17. Mar; individuals hospitalised with COVID-19 diagnosis	191; nr (nr), mean 63 (15); 69%	PCR / yes, partially, heart failure and chronic kidney disease	in-hospital mortality	Cox / model 1: age, comorbidities (HTN, CHF, DM), ACE inhibitor/ARBs; model 2: age, comorbidities (CKD, COPD, CANC), beta-blocker / age numeric / sex na	NOS =8	2 estimates<br>Chronic kidney disease-Hospital mortality-HR-EUR-9-94.5 // Heart failure-Hospital mortality-HR-EUR-2-80.9
Di Castelnuovo; Italy[139]	cohort; multi-center, 30; 19. Feb - 23. Mar; individuals hospitalised with COVID-19 diagnosis	3894, Complete-case 3454; median 67 (nr), nr (nr); 62%	PCR / yes, partially, only on obesity and CKD	in-hospital mortality	Cox / age, sex, comorbidities (HTN, DM, Myo, CHF, CANC, Resp, Obesity), smoking, CKD stage; results for 3 types of imputation (complete-case (3) is included in meta-analysis); model estimated for age groups 18-64, 65-74 and >=75 (same comorbidites as in main model) / age 18-44 / female	NOS =9	8 estimates<br>Diabetes-Hospital mortality-HR-EUR-9-49.7 // Hypertension-Hospital mortality-HR-EUR-8-58 // Obesity/BMI>30-Hospital mortality-HR-EUR-6-89.2 // Chronic kidney disease-Hospital mortality-HR-EUR-9-94.5 // Cancer-Hospital mortality-HR-EUR-3-32.1 // Heart failure-Hospital mortality-HR-EUR-2-80.9 // Infarction-Hospital mortality-HR-EUR-1-0 // Respiratory disease-Hospital mortality-HR-EUR-4-0<br>and<br>Analysis of age-stratified estimates
Docherty; UK[140]	cohort; multi-center, 208; 6. Feb - 19. Apr; individuals hospitalised with COVID-19 diagnosis	15194; median 73 (58–82), nr (nr); 60%	PCR / yes, partially, since CCI was used	in-hospital mortality	Cox / age, sex, comorbidities (CANCact, cardiac diseases, neurological, CKD, CLD, DEM, DM, obesity, Resp) / age<50; 10-year increment for ORs /male	NOS =8	9 estimates<br>Diabetes-Hospital mortality-HR-EUR-9-49.7 // Neurological disease-Hospital mortality-HR-EUR-2-0 // Dementia-Hospital mortality-HR-EUR-3-75.1 // Obesity/BMI>30-Hospital mortality-HR-EUR-6-89.2 // Chronic kidney disease-Hospital mortality-HR-EUR-9-94.5 // Heart disease-Hospital mortality-HR-EUR-2-33.8 // Cancer-Hospital mortality-HR-EUR-3-32.1 // Respiratory disease-Hospital mortality-HR-EUR-4-0 // Chronic liver disease-Hospital mortality-HR-EUR-1-0
Galloway; UK[141]	cohort; 2 hospitals; 1. Mar - 17. Apr; critically ill patients diagnosed with COVID-19 admitted to hospital	1157; median 71 (57-82), nr (nr); 58%	PCR / yes, partially, on chronic lung disease	admission to ICU, in-hospital mortality	Competing risk model / models death and critical care: sex, age, comorbidities (CANCact, CKD, DM, HTN, IHD, chronic lung disease) / age numeric / female	NOS =8	12 estimates<br>Diabetes-Hospital mortality-HR-EUR-9-49.7 // Cancer/Active-Hospital mortality-HR-EUR-3-25.6 // Hypertension-Hospital mortality-HR-EUR-8-58 // Chronic kidney disease-Hospital mortality-HR-EUR-9-94.5 // Coronary artery disease-Hospital mortality-HR-EUR-4-83.3 // Respiratory disease-Hospital mortality-HR-EUR-4-0 // Cancer/Active-ICU admission-HR-EUR-1-0 // Chronic kidney disease-ICU admission-HR-EUR-2-86.1 // Diabetes-ICU admission-HR-EUR-2-0 // Hypertension-ICU admission-HR-EUR-2-0 // Coronary artery disease-ICU admission-HR-EUR-1-0 // Respiratory disease-ICU admission-HR-EUR-1-0

Study	Study design; setting, number of hospitals; period; Study population	Sample size; age (median (IQR), mean (SD or CI) or % in certain age group(s)); % of males	Given definition of COVID-19/ pre-existing condition (if yes, see section 2.3 for detail)	Outcomes relevant for review	Adjustment method (Regression Model) / Covariates / Reference age and sex category	Risk of bias	Analyses the study participated in [Main meta-analyses reported as: number of the resulted effect estimates the study contributed to; a meta-analysis is described as Condition-Outcome-Measure of association-Region-Total number of studies in the analysis-I^2%]
Geretti; UK[142]	cohort; multi-center, 207; 17. Jan - 18. Jun; follow-up ended at death, or 3 days after discharge to palliative care or at day 28 for those remaining alive as an inpatient; individuals hospitalised with COVID-19 diagnosis	47592; HIV positive: median 56 (49-62), nr (nr); HIV negative: median 74 (60-68), nr (nr); 66% HIV positive group, 57% HIV-negative group	PCR (90.5%) /clinical diagnosis (9.5%) / yes, partially, only for HIV	in-hospital mortality (by day 28); admission to critical care	Cox, Log / model Cox: sex, age, ethnicity, other: indeterminate/probable hospital acquisition of COVID-19, comorbidities at admission (HIV, chronic neurological disorder, chronic haematological disease, chronic cardiac disease, chronic pulmonary disease, chronic renal disease, DM, obesity, DEM, liver disease (mild, moderate or severe), CANC), baseline date (date of symptom start or positive PCR test); Model LOG: sex, age, ethnicity, baseline date, indeterminate/probable hospital acquisition of COVID-19, and 10 comorbidities besides HIV-status	JBI=9	2 estimates<br>HIV-Hospital mortality-HR-EUR-1-0 // HIV-ICU admission-OR-EUR-1-0
Giacomelli ; Italy[143]	cohort; single-center; 21. Feb - 19. Mar; follow-up until 20. Apr; individuals hospitalised with COVID-19 diagnosis	233; median 61 (50-72), nr (nr); 70%	PCR / yes	in-hospital mortality	Cox / age, sex, comorbidities (obesity, age, unadjusted Charlson comorbidity index), anti-hypertensive agent, COVID-19 disease severity, anaemia, laboratory values / age per 10 years more / female	NOS =7	1 estimate<br>Obesity/BMI>30-Hospital mortality-HR-EUR-6-89.2
Goicoechea; Spain[144]	cohort; single-center; 12. Mar - 10. Apr; individuals hospitalised with COVID-19 diagnosis	36; nr (nr), mean 71 (12); 64%	PCR / NA (no comorbidity in model)	in-hospital mortality	Cox / 5 models: 1) age, haemodialysis time; 2) and 3) age, laboratory findings; 4) and 5) age, treatment / age 1-year increase / sex na	NOS =7	Analysis of evidence for specific population groups – patients receiving haemodialysis
Grasselli; Italy[145]	cohort; ICUs; 20. Feb - 22. Apr; follow-up until 30. May; critically ill individuals with COVID-19 in ICU	3988; median 63 (56-69), nr (nr); 80%	PCR / yes	ICU mortality	Cox / age, sex, laboratory values at admission, respiratory support, medication/therapy, comorbidities (HTN, hypercholesterolemia, cardiac diseases, DM, CANC, COPD) / age in 10-y increments / female	NOS =9	The outcome was excluded from the reporting
Halasz; Italy[146]	cohort; single-center; Feb – Apr; critically ill individuals with COVID-19 in ICU	242; median 64 (56-71), nr (nr); 80%	PCR / yes, partially, only for obesity	ICU mortality	Log / age, sex, BMI, comorbidities (HTN, CVD, COPD, DM) / na	NOS =7	The outcome was excluded from the reporting
Hewitt; Europe[147]	cohort; multi-center, 11; 27. Feb - 28. Apr; individuals hospitalised with COVID-19 diagnosis	1564; median 74 (61-83), nr (nr); 58%	PCR /clinical diagnosis (% unknown) / yes, partially, only for reduced renal function	in-hospital mortality	Cox, Log / all models: age, sex, smoking status, comorbidities (DM, CAD, HTN, reduced renal function (eGRF laboratory value)) and clinical frailty score, laboratory values / age <65 / female	NOS =9	8 estimates<br>Diabetes-Hospital mortality-HR-EUR-9-49.7 // Hypertension-Hospital mortality-HR-EUR-8-58 // Chronic kidney disease-Hospital mortality-HR-EUR-9-94.5 // Coronary artery disease-Hospital mortality-HR-EUR-4-83.3 // Diabetes-Hospital mortality-OR-EUR-5-19.6 // Coronary artery disease-Hospital mortality-OR-EUR-3-55.8 // Hypertension-Hospital mortality-OR-EUR-4-24.6 // Chronic kidney disease-Hospital mortality-OR-EUR-4-0
Khalil; UK[148]	cohort; single-center; 7. Mar - 7. Apr; follow-up until 8. May; individuals hospitalised with COVID-19 diagnosis	220; nr (nr), mean 67 (65–69); 59%	PCR / NA (nr of comorbidities was included in model only)	in-hospital mortality	Cox / age, sex, number of comorbidities / age nr / female	NOS =6	Risk “number of comorbidities” was excluded from meta-analyses
Lee (a); UK[149]	cohort; multi-center, 55; 18. Mar - 26. Apr; individuals with active cancer diagnosed with COVID-19	800; median 69 (59-76), nr (nr); 56%	PCR / yes, partially, for CANC	case mortality	Log / age, sex, comorbidities (DM, HTN, COPD, other) / na	NOS =7	Analysis of evidence for specific population groups – patients with cancer

Study	Study design; setting, number of hospitals; period;<br>Study population	Sample size; age (median (IQR), mean (SD or CI) or % in certain age group(s)); % of males	Given definition of COVID-19/ pre-existing condition (if yes, see section 2.3 for detail)	Outcomes relevant for review	Adjustment method (Regression Model) / Covariates / Reference age and sex category	Risk of bias	**Analyses the study participated in**<br>[Main meta-analyses reported as: number of the resulted effect estimates the study contributed to; a meta-analysis is described as Condition-Outcome-Measure of association-Region-Total number of studies in the analysis-I^2%]
Lee (b); UK[150]	cohort; multi-center, 66; 18. Mar - 18. May;<br>individuals with active cancer diagnosed with COVID-19	1044; median 70 (60-77), nr (nr); 57%	PCR / ICD-10 for cancer type (variable of interest);	case mortality	Log / tumor subtype adjusted by age and sex / na	NOS =8	Analysis of evidence for specific population groups – patients with cancer
Merzon; Israel[151]	cohort; electronic database; 1. Feb - 30. Apr;<br>health services members with COVID-19	782; nr (nr), mean 36 (CI: 34-37); 49%	PCR / no, however, information provided that comorbidities were selected based on ICD-10	hospitalisation	Log / age (<50), sex, comorbidities (depression, schizophrenia, DEM, DM, HTN, CVD, Resp), BMI, smoking, laboratory values / na	NOS =8	8 estimates<br>Depression-Hospitalisation-OR-EUR-2-77 // Psychiatric disorder-Hospitalisation-OR-EUR-2-15.6 // Dementia-Hospitalisation-OR-EUR-3-50 // Diabetes-Hospitalisation-OR-EUR-3-0 // Hypertension-Hospitalisation-OR-EUR-3-0 // Cardiovascular disease-Hospitalisation-OR-EUR-2-12.3 // Respiratory disease-Hospitalisation-OR-EUR-2-76.4 // Obesity/BMI>30-Hospitalisation-OR-EUR-3-96.6
Passamonti ; Italy[152]	cohort; multi-center, 66; 25. Feb - 18. May;<br>individuals with haematological malignancy diagnosed with COVID-19 admitted to hospital	536, 451 hospitalised; median 68 (58-77), nr (nr); 63%	PCR / yes, on type of haematological malignancy and CCI	case mortality	Cox / age, sex, CCI, haematological malignancy, types (vs myeloproliferative neoplasms), time since diagnosis, COVID-19 severity / age numeric / male	JBI=7	Analysis of evidence for specific population groups – patients with cancer
Perez-Guzman; UK[153]	cohort; multi-center, 3; 25. Feb - 5. Apr; follow-up until 1. May;<br>individuals hospitalised with COVID-19 diagnosis	614; median 69 (54–79), nr (nr); 62%	PCR / yes, defined by ECI score	in-hospital mortality	Log / for each comorbidity age-adjusted model: comorbidities: ART, AST, CHF, CKD, CLD/Cirrhosis, CLD/noncirrhotic, CRB, DEM, DM, DVT/PE, haematological malignancy, HIV, HTN, IHD, solid malignancy, COPD, any comorbidity / age numeric / sex na	JBI=8 6%	16 estimates<br>Dementia-Hospital mortality-OR-EUR-4-49.7 // Diabetes-Hospital mortality-OR-EUR-5-19.6 // Coronary artery disease-Hospital mortality-OR-EUR-3-55.8 // Hypertension-Hospital mortality-OR-EUR-4-24.6 // Chronic kidney disease-Hospital mortality-OR-EUR-4-0 // Arrhythmia-Hospital mortality-OR-EUR-2-0 // Asthma-Hospital mortality-OR-EUR-1-0 // Heart failure-Hospital mortality-OR-EUR-2-0 // Chronic liver/Cirrhosis-Hospital mortality-OR-EUR-1-0 // Chronic liver/Non-cirrhotic-Hospital mortality-OR-EUR-1-0 // Cerebrovascular/Stroke-Hospital mortality-OR-EUR-3-79.8 // V.thromboembolism-Hospital mortality-OR-EUR-1-0 // Cancer/Hematol-Hospital mortality-OR-EUR-2-64.8 // HIV-Hospital mortality-OR-EUR-1-0 // Cancer/Solid-Hospital mortality-OR-EUR-1-0 // COPD-Hospital mortality-OR-EUR-1-0
Pinato; Europe[154]	cohort; multi-center, 19; 26. Feb - 1. Apr; follow-up until 11. May;<br>patients with COVID-19 and cancer identified from registry, treated as inpatients (ward, ICU) and outpatients	890; median 68 (13), nr (nr); 56%	PCR / only for tumor status	case mortality	Cox / age, number comorbidities (categorical, >=2 vs 0-1), comorbidities: CANCact "tumor status" (active malignancy vs remission/no measurable disease), severity of cancer: tumor stage; drugs: anticancer therapy / age<65 / sex na	NOS =7	Analysis of evidence for specific population groups – patients with cancer
Regina; Switzerland[155]	cohort; single-center; 1. Mar - 25. Mar; 14 day follow-up;<br>individuals hospitalised with COVID-19 diagnosis	145; median 62 (52-74), nr (nr); 62%	PCR / yes, partially, CCI was used for number of comorbidities	intubation	Log / age, sex, comorbidities (HTN, COPD), symptoms: more than 7 days of symptoms, laboratory values: dyspnea, temperature, heart rate, qSOFA score, bilateral radiological infiltrate, acute kidney injury on admission, C-reactive protein / age numeric / female	NOS =9	2 estimates<br>COPD-Intubation-OR-EUR-1-0 // Hypertension-Intubation-OR-EUR-2-84.2

Study	Study design; setting, number of hospitals; period; Study population	Sample size; age (median (IQR), mean (SD or CI) or % in certain age group(s)); % of males	Given definition of COVID-19/ pre-existing condition (if yes, see section 2.3 for detail)	Outcomes relevant for review	Adjustment method (Regression Model) / Covariates / Reference age and sex category	Risk of bias	Analyses the study participated in<br>[Main meta-analyses reported as: number of the resulted effect estimates the study contributed to; a meta-analysis is described as Condition-Outcome-Measure of association-Region-Total number of studies in the analysis-I^2%]
Reilev; Denmark[156]	population-based study; electronic database; 27. Feb - 19. May; individuals community-managed / hospitalised with COVID-19 diagnosis	n= 9519 for case mortality; 2090 hospital mortality; 9519 for hospitalisation; hospitalized: median 71 (56-80), nr (nr); 54%	PCR / yes, comorbidities defined with ICD-10 codes	hospitalisation, in-hospital mortality, case mortality	Log / age, sex, 1 of the following comorbidities: ART, CANC, CHF, CKD, CLD, CRB, DEM, DM, HTN, IHD, obesity, Organ, Resp, Rheuma / age 50-59 / female	NOS =8	45 estimates<br>Obesity/BMI>30-Hospital mortality-OR-EUR-3-75.5 // Dementia-Hospital mortality-OR-EUR-4-49.7 // Diabetes-Hospital mortality-OR-EUR-5-19.6 // Respiratory disease-Hospital mortality-OR-EUR-2-30.7 // Coronary artery disease-Hospital mortality-OR-EUR-3-55.8 // Hypertension-Hospital mortality-OR-EUR-4-24.6 // Chronic kidney disease-Hospital mortality-OR-EUR-4-0 // Arrhythmia-Hospital mortality-OR-EUR-2-0 // Heart failure-Hospital mortality-OR-EUR-2-0 // Cerebrovascular/Stroke-Hospital mortality-OR-EUR-3-79.8 // Cancer-Hospital mortality-OR-EUR-1-0 // Chronic liver disease-Hospital mortality-OR-EUR-1-0 // Organ transplant recipients-Hospital mortality-OR-EUR-1-0 // Rheumatological disease-Hospital mortality-OR-EUR-1-0 // Psychiatric disorder-Hospital mortality-OR-EUR-1-0 // Psychiatric disorder-Hospitalisation-OR-EUR-2-15.6 // Dementia-Hospitalisation-OR-EUR-3-50 // Diabetes-Hospitalisation-OR-EUR-3-0 // Hypertension-Hospitalisation-OR-EUR-3-0 // Respiratory disease-Hospitalisation-OR-EUR-2-76.4 // Obesity/BMI>30-Hospitalisation-OR-EUR-3-96.6 // Arrhythmia-Hospitalisation-OR-EUR-2-0 // Cancer-Hospitalisation-OR-EUR-2-67 // Heart failure-Hospitalisation-OR-EUR-1-0 // Chronic kidney disease-Hospitalisation-OR-EUR-2-88.7 // Chronic liver disease-Hospitalisation-OR-EUR-2-38.8 // Cerebrovascular/Stroke-Hospitalisation-OR-EUR-2-0 // Coronary artery disease-Hospitalisation-OR-EUR-1-0 // Organ transplant recipients-Hospitalisation-OR-EUR-1-0 // Rheumatological disease-Hospitalisation-OR-EUR-1-0 // Psychiatric disorder-Case mortality-OR-EUR-1-0 // Arrhythmia-Case mortality-OR-EUR-1-0 // Cancer-Case mortality-OR-EUR-1-0 // Heart failure-Case mortality-OR-EUR-1-0 // Chronic kidney disease-Case mortality-OR-EUR-1-0 // Chronic liver disease-Case mortality-OR-EUR-1-0 // Cerebrovascular/Stroke-Case mortality-OR-EUR-1-0 // Dementia-Case mortality-OR-EUR-1-0 // Diabetes-Case mortality-OR-EUR-1-0 // Hypertension-Case mortality-OR-EUR-1-0 // Coronary artery disease-Case mortality-OR-EUR-1-0 // Obesity/BMI>30-Case mortality-OR-EUR-1-0 // Organ transplant recipients-Case mortality-OR-EUR-1-0 // Respiratory disease-Case mortality-OR-EUR-1-0 // Rheumatological disease-Case mortality-OR-EUR-1-0
Rivera-Izquierdo; Spain[157]	case series; single-center; 16. Mar - 10. Apr; individuals hospitalised with COVID-19 diagnosis	238; nr (nr), mean 65 (15); 55%	PCR / no	in-hospital mortality	Cox / 3 models: 1) adjustment by age; 2) age, basic activities of daily life dependence, DM, ageusia, laboratory values, interstitial opacities; 3) age, DM, laboratory values. Model 1 is included / age per unit increase / sex na	NOS =5	5 estimates<br>Cardiovascular disease-Hospital mortality-HR-EUR-2-0 // Diabetes-Hospital mortality-HR-EUR-9-49.7 // Hypertension-Hospital mortality-HR-EUR-8-58 // Chronic kidney disease-Hospital mortality-HR-EUR-9-94.5 // Respiratory disease-Hospital mortality-HR-EUR-4-0
Rossi, A; Italy[158]	cohort; single-center; 8. Mar - 30. Mar; critically ill individuals hospitalised with COVID-19 diagnosis	95; nr (nr), mean 62 (12); 81%	PCR / yes, partially, for BMI categories / obesity	in-hospital mortality (28 day survival)	Cox / age, sex, smoking status, comorbidities (obesity, coronary heart disease, CHF, HTN, DM, COPD, chronic renal failure, Immun, immunodeficiency or immunosuppressed state and CANC) / na	NOS =8	The outcome was excluded from the reporting
Rossi, P; Italy[159]	cohort; population-based; 27. Feb - 2. Apr; symptomatic patients who tested positive for COVID-19	2143 for hospitalisation,2362 for mortality (for models including single comorbidities); 1866 for hospitalization for model w CCI and 2025 for case mortality for model with cci; nr (nr), mean 63 (nr); 50%	PCR / no	case mortality, hospitalization	Multivariate proportional hazard models / 2 models with age, sex and one of the following: ART, CANC, CHF, CKD, COPD, DEM, DM, dyslipidemia, HTN, IHD, obesity, vascular disease; 2 models with sex, age, calendar period, time from symptom to diagnosis, place of birth, CCI / na	NOS =9	24 estimates<br>Chronic kidney disease-Hospitalisation-HR-EUR-2-76.3 // COPD-Hospitalisation-HR-EUR-2-94.1 // Dementia-Hospitalisation-HR-EUR-2-93.4 // Diabetes-Hospitalisation-HR-EUR-2-44.2 // Hypertension-Hospitalisation-HR-EUR-2-74.7 // Dyslipidemia or hyperlipidemia-Hospitalisation-HR-EUR-2-37.5 // Cancer-Hospitalisation-HR-EUR-2-80.1 // Obesity/BMI>30-Hospitalisation-HR-EUR-2-0 // Arrhythmia-Hospitalisation-HR-EUR-1-0 // Coronary artery disease-Hospitalisation-HR-EUR-1-0 // Heart failure-Hospitalisation-HR-EUR-1-0 // Cardiovascular disease-Hospitalisation-HR-EUR-1-0 // Chronic kidney disease-Case mortality-HR-EUR-2-0 // COPD-Case mortality-HR-EUR-2-0 // Dementia-Case mortality-HR-EUR-2-0 // Diabetes-Case mortality-HR-EUR-2-16.8 // Hypertension-Case mortality-HR-EUR-2-91.9 // Dyslipidemia or hyperlipidemia-Case mortality-HR-EUR-2-48.6 // Cancer-Case mortality-HR-EUR-2-0 // Obesity/BMI>30-Case mortality-HR-EUR-2-0 // Arrhythmia-Case mortality-HR-EUR-1-0 // Heart failure-Case mortality-HR-EUR-1-0 // Coronary artery disease-Case mortality-HR-EUR-1-0 // Cardiovascular disease-Case mortality-HR-EUR-1-0

Study	Study design; setting, number of hospitals; period; Study population	Sample size; age (median (IQR), mean (SD or CI) or % in certain age group(s)); % of males	Given definition of COVID-19/ pre-existing condition (if yes, see section 2.3 for detail)	Outcomes relevant for review	Adjustment method (Regression Model) / Covariates / Reference age and sex category	Risk of bias	Analyses the study participated in [Main meta-analyses reported as: number of the resulted effect estimates the study contributed to; a meta-analysis is described as Condition-Outcome-Measure of association-Region-Total number of studies in the analysis-I^2%]
Rottoli; Italy[160]	cohort; single-center; 1. Mar - 20. Apr; follow-up until 27. Apr; individuals hospitalised with COVID-19 diagnosis	482; nr (nr), mean 66 (17); 63%	PCR / BMI is defined obesity >=30; no definition of other comorbidities	In-hospital mortality, ICU admission	Cox, Log / age, sex, comorbidities (CKD, CRB, DM, HTN, obesity) / age<60 / female	NOS =6	20 estimates Diabetes-Hospital mortality-HR-EUR-9-49.7 // Hypertension-Hospital mortality-HR-EUR-8-58 // Obesity/BMI>30-Hospital mortality-HR-EUR-6-89.2 // Chronic kidney disease-Hospital mortality-HR-EUR-9-94.5 // Cerebrovascular/Stroke-Hospital mortality-HR-EUR-1-0 // Chronic kidney disease-ICU admission-HR-EUR-2-86.1 // Diabetes-ICU admission-HR-EUR-2-0 // Hypertension-ICU admission-HR-EUR-2-0 // Obesity/BMI>30-ICU admission-HR-EUR-1-0 // Cerebrovascular/Stroke-ICU admission-HR-EUR-1-0 // Diabetes-ICU admission-OR-EUR-2-0 // Obesity/BMI>30-ICU admission-OR-EUR-2-56.2 // Cerebrovascular/Stroke-ICU admission-OR-EUR-1-0 // Hypertension-ICU admission-OR-EUR-1-0 // Chronic kidney disease-ICU admission-OR-EUR-1-0 // Obesity/BMI>30-Hospital mortality-OR-EUR-3-75.5 // Diabetes-Hospital mortality-OR-EUR-5-19.6 // Hypertension-Hospital mortality-OR-EUR-4-24.6 // Chronic kidney disease-Hospital mortality-OR-EUR-4-0 // Cerebrovascular/Stroke-Hospital mortality-OR-EUR-3-79.8
Russell; UK[161]	cohort; single-center; 29. Feb - 12. May; cancer patients with a confirmed COVID-19 diagnosis	156; nr (nr), mean 65 (15); 58%	PCR / yes, partially, for cancer type yes, (nr of comorbidities-com. Are listed but not defined)	case mortality	Cox / Effects of comorbidities are adjusted for age, deprivation Index, ethnicity, sex, smoking / age<60 / male	NOS =7	Analysis of evidence for specific population groups – patients with cancer
Sanchez-Pina; Spain[162]	case-control; single-center; 7. Mar - 7. Apr; individuals hospitalised with COVID-19 diagnosis	cases: 39, controls: 53; cases: nr (nr), mean 65 (36-88); cases: 59%	PCR / yes, haematological malignancies are listed	in-hospital mortality	Log / age, haematological disease / age<70 / sex na	NOS =6	1 estimate Cancer/Hematol-Hospital mortality-OR-EUR-2-64.8
Sapey; UK[163]	cohort; single-center; 10. Mar -17. Apr; individuals hospitalised with COVID-19 diagnosis	1448; median 68 (52-80), nr (nr); 56%	PCR / no	in-hospital mortality	Cox / age, sex, comorbidity counts, deprivation / age z-score / female	NOS =9	Risk "comorbidity counts" was excluded from meta-analyses
Shah, V; UK[164]	cohort; single-center; 13. Mar - 5. May; individuals hospitalised with COVID-19 diagnosis	1183; median 71 (57-82), nr (nr); 58%	PCR / yes, on malignancies (supplementary results)	in-hospital mortality	Cox /age, sex, haematological malignancy / na	JBI=8	1 estimate Cancer/Hematol-Hospital mortality-HR-EUR-1-0
Simonnet; France[165]	cohort; single-center; 27. Feb - 5. Apr; individuals with COVID-19 diagnosis admitted to ICU	124; median 60 (51-70), nr (nr); 73%	PCR / yes, partially, only for BMI categories not for any other comorbidities	intubation	Log / age, sex, comorbidities (DM, HTN, dyslipidemia) and BMI categories / age numeric / female	NOS =8	5 estimates Hypertension-Intubation-OR-EUR-2-84.2 // Obesity/BMI>30-Intubation-OR-EUR-1-0 // Overweight-Intubation-OR-EUR-1-0 // Diabetes-Intubation-OR-EUR-1-0 // Dyslipidemia or hyperlipidemia-Intubation-OR-EUR-1-0
Sisó-Almirall; Spain[166]	case series; multi-center, 4; 29. Feb - 4. Apr; hospitalised individuals diagnosed with COVID-19	322; nr (nr), mean 57 (18); 50%	PCR / no	hospitalisa tion	Log / age and sex adjusted models with the following comorbidities (one per model): Alzheimer disease, ART, AST, Auto, CANC, CKD, CLD, COPD, CRB, CVD, depression, DM, dyslipidemia, HTN, obesity, thyroid alterations / na	NOS =7	15 estimates Depression-Hospitalisation-OR-EUR-2-77 // Dementia-Hospitalisation-OR-EUR-3-50 // Diabetes-Hospitalisation-OR-EUR-3-0 // Hypertension-Hospitalisation-OR-EUR-3-0 // Cardiovascular disease-Hospitalisation-OR-EUR-2-12.3 // Obesity/BMI>30-Hospitalisation-OR-EUR-3-96.6 // Arrhythmia-Hospitalisation-OR-EUR-2-0 // Cancer-Hospitalisation-OR-EUR-2-67 // Chronic kidney disease-Hospitalisation-OR-EUR-2-88.7 // Chronic liver disease-Hospitalisation-OR-EUR-2-38.8 // Cerebrovascular/Stroke-Hospitalisation-OR-EUR-2-0 // Asthma-Hospitalisation-OR-EUR-1-0 // Autoimmune condition-Hospitalisation-OR-EUR-1-0 // COPD-Hospitalisation-OR-EUR-1-0 // Dyslipidemia or hyperlipidemia-Hospitalisation-OR-EUR-1-0
Wendel Garcia; Europe[167]	cohort; multi-center, 54; nr - 22. Apr; critically ill patients with COVID-19 in Europe as captured in registry	639; nr (nr), mean 63 (53-71); 75%	PCR / no	ICU mortality	Cox / laboratory values and comorbidities (IHD) / na	NOS =7	The outcome was excluded from the reporting

Study	Study design; setting, number of hospitals; period; Study population	Sample size; age (median (IQR), mean (SD or CI) or % in certain age group(s)); % of males	Given definition of COVID-19/ pre-existing condition (if yes, see section 2.3 for detail)	Outcomes relevant for review	Adjustment method (Regression Model) / Covariates / Reference age and sex category	Risk of bias	**Analyses the study participated in**<br>[Main meta-analyses reported as: number of the resulted effect estimates the study contributed to; a meta-analysis is described as Condition-Outcome-Measure of association-Region-Total number of studies in the analysis-I^2%]
Yarza; Spain[168]	cohort; single-center; 9. Mar - 19. Apr;<br>individuals with cancer undergoing treatment hospitalised with COVID-19 diagnosis	63; 57%=50-70; 54%	PCR / no	in-hospital mortality	Log / each comorbidity (HTN, DM, CKD, COPD, previous venous thromboembolic disease, chronic anaemia, ACEI/ARBsI) adjusted by age, sex, hypertension, diabetes, CKD, COPD, previous VTE, smoking habit; primary tumour types (5 different types/categories) adjusted by age, sex, CT; metastatic disease adjusted by age, sex, COPD, previous venous thromboembolic disease, metastatsis, ECOG; visceral metastatasis adjusted by age, sex, previous VTE, pulmonary involvement; cancer treatment (chemotherapy alone, immunotherapy and chemotherapy, immunotherapy alone) adjusted by age, sex, ECOG, metastasis, previous VTE, COPD / age<65 / male	NOS =6	Analysis of evidence for specific population groups – patients with cancer
**Regions of America: North**							
Antwi-Amoabeng ; USA[169]	cohort; single-center; 12. Mar - 8. May;<br>individuals hospitalised with COVID-19 diagnosis	172; median 53 (33-68), nr (nr); 56%	PCR / no	in-hospital mortality	Log / age, sex, ethnicity, comorbidities (DM, HTN, Obesity, CKD, COPD), ICU stay / age <61 / male	NOS =8	5 estimates<br>Chronic kidney disease-Hospital mortality-OR-AMR: North-11-19.7 // COPD-Hospital mortality-OR-AMR: North-5-37.9 // Diabetes-Hospital mortality-OR-AMR: North-12-0 // Hypertension-Hospital mortality-OR-AMR: North-12-68.6 // Obesity/BMI>30-Hospital mortality-OR-AMR: North-11-0
Argenziano; USA[170] pre-print	cohort; single-center; 1. Mar - 5. Apr;<br>individuals hospitalised with COVID-19 diagnosis	1000, 841 in the regression; median 63 (50-75), nr (nr); 60%	PCR / yes, partially	in-hospital mortality, intubation	Cox/ age, sex, BMI, smoking, comorbidities (CAD, CHF, CRB, HTN, DM, cirrhosis, HIV, IBD, Resp, CKD, viral hepatitis, Organ, CANC, Rheuma, Immun, number comorbidities) / age numeric / male	NOS =5	30 estimates<br>Coronary artery disease-Hospital mortality-HR-AMR: North-3-0 // Cancer/Active-Hospital mortality-HR-AMR: North-1-0 // Heart failure-Hospital mortality-HR-AMR: North-3-87.2 // Chronic kidney disease-Hospital mortality-HR-AMR: North-4-71.9 // Chronic liver/Cirrhosis-Hospital mortality-HR-AMR: North-1-0 // Cerebrovascular/Stroke-Hospital mortality-HR-AMR: North-1-0 // Diabetes-Hospital mortality-HR-AMR: North-5-0 // HIV-Hospital mortality-HR-AMR: North-2-52.9 // Hypertension-Hospital mortality-HR-AMR: North-4-0 // Inflammatory bowel disease-Hospital mortality-HR-AMR: North-1-0 // Immunosuppression-Hospital mortality-HR-AMR: North-1-0 // Organ transplant recipients-Hospital mortality-HR-AMR: North-1-0 // Respiratory disease-Hospital mortality-HR-AMR: North-1-0 // Rheumatological disease-Hospital mortality-HR-AMR: North-1-0 // Hepatitis-Hospital mortality-HR-AMR: North-1-0 // Coronary artery disease-Intubation-HR-AMR: North-1-0 // Cancer/Active-Intubation-HR-AMR: North-1-0 // Heart failure-Intubation-HR-AMR: North-1-0 // Chronic kidney disease-Intubation-HR-AMR: North-1-0 // Chronic liver/Cirrhosis-Intubation-HR-AMR: North-1-0 // Cerebrovascular/Stroke-Intubation-HR-AMR: North-1-0 // Diabetes-Intubation-HR-AMR: North-1-0 // HIV-Intubation-HR-AMR: North-1-0 // Hypertension-Intubation-HR-AMR: North-1-0 // Inflammatory bowel disease-Intubation-HR-AMR: North-1-0 // Immunosuppression-Intubation-HR-AMR: North-1-0 // Organ transplant recipients-Intubation-HR-AMR: North-1-0 // Respiratory disease-Intubation-HR-AMR: North-1-0 // Rheumatological disease-Intubation-HR-AMR: North-1-0 // Hepatitis-Intubation-HR-AMR: North-1-0
Azar; USA[171]	cohort; electronic database; 1. Jan - 8. Apr;<br>individuals diagnosed with COVID-19	1052; nr (nr), mean 53 (CI: 52-54); 49%	PCR / no	hospitalisation	Log / age (6 groups), sex, SES, race/ethnicity, comorbidities (DM, HTN, CHF, CVD, CANC, COPD, AST, depression), smoking / age 18-39 / female	other =good	8 estimates<br>Asthma-Hospitalisation-OR-AMR: North-3-66.6 // Cancer-Hospitalisation-OR-AMR: North-5-17.8 // Heart failure-Hospitalisation-OR-AMR: North-4-80.7 // COPD-Hospitalisation-OR-AMR: North-4-15.2 // Cardiovascular disease-Hospitalisation-OR-AMR: North-4-81.1 // Depression-Hospitalisation-OR-AMR: North-1-0 // Diabetes-Hospitalisation-OR-AMR: North-8-37.6 // Hypertension-Hospitalisation-OR-AMR: North-5-59.3
Chhiba; USA[172]	cohort; multi-center, 10; 1. Mar - 15. Apr;<br>individuals diagnosed with COVID-19	1526; 73%<40; 18%<70; 47%	PCR / yes, ICD codes provided; obesity is defined	hospitalisation	Poisson / age, sex, race/ethnicity, smoking, comorbidities (AST, obesity, CAD, diabetes, HTN, obstructive sleep apnea, COPD, allergic rhinitis, rhinosinusitis, and immunodeficiency) / age >= 70<br>/male	ROBINS= 2xhigh 2xlow 2xunclear	8 estimates<br>Asthma-Hospitalisation-RR-AMR: North-1-0 // Coronary artery disease-Hospitalisation-RR-AMR: North-1-0 // COPD-Hospitalisation-RR-AMR: North-1-0 // Diabetes-Hospitalisation-RR-AMR: North-1-0 // Hypertension-Hospitalisation-RR-AMR: North-1-0 // Immunosuppression-Hospitalisation-RR-AMR: North-1-0 // Obesity/BMI>30-Hospitalisation-RR-AMR: North-2-0 // Obstructive sleep apnea-Hospitalisation-RR-AMR: North-1-0

Study	Study design; setting, number of hospitals; period; Study population	Sample size; age (median (IQR), mean (SD or CI) or % in certain age group(s)); % of males	Given definition of COVID-19/ pre-existing condition (if yes, see section 2.3 for detail)	Outcomes relevant for review	Adjustment method (Regression Model) / Covariates / Reference age and sex category	Risk of bias	Analyses the study participated in [Main meta-analyses reported as: number of the resulted effect estimates the study contributed to; a meta-analysis is described as Condition-Outcome-Measure of association-Region-Total number of studies in the analysis-I^2%]
Chilimuri; USA[173]	cohort; single-center; 9. Mar - 9. Apr; individuals hospitalised with COVID-19 diagnosis	375; median 63 (52-72), nr (nr); 63%	PCR / no	in-hospital mortality	Log / age, sex, comorbidities (HTN, DM, CVD, CKD), laboratory values / age numeric / female	NOS =8	4 estimates Chronic kidney disease-Hospital mortality-OR-AMR: North-11-19.7 // Diabetes-Hospital mortality-OR-AMR: North-12-0 // Hypertension-Hospital mortality-OR-AMR: North-12-68.6 // Cardiovascular disease-Hospital mortality-OR-AMR: North-4-0
Costa Monteiro; USA[174]	cohort; single-center; 12. Mar -16. Apr; individuals hospitalised with COVID-19 diagnosis	112; median 61 (45-74), nr (nr); 66%	PCR / no	mechanica l intubation	Log / age, sex, comorbidities (obesity, DM, HTN, CAD, CKD), tobacco exposure history, race/ethnicity / age numeric / female	JBI=8 2%	5 estimates Coronary artery disease-Intubation-OR-AMR: North-3-18.1 // Chronic kidney disease-Intubation-OR-AMR: North-5-72.5 // Diabetes-Intubation-OR-AMR: North-11-0 // Hypertension-Intubation-OR-AMR: North-9-0 // Obesity/BMI>30-Intubation-OR-AMR: North-10-0
Crouse; USA[175]	cohort; electronic database; 25. Feb -22. Jun; individuals with COVID-19 tested at center	604; nr (nr), nr (nr); 45%	PCR / no	case mortality	Log / age, sex, comorbidities (DM, obesity, HTN), race / age var1: <50; age var2: 50-70 / female	JBI=7 5%	3 estimates Diabetes-Case mortality-OR-AMR: North-3-75.4 // Hypertension-Case mortality-OR-AMR: North-1-0 // Obesity/BMI>30-Case mortality-OR-AMR: North-2-5.6
Cummings ; USA[176]	cohort; 2 hospitals; 2. Mar - 1. Apr; follow-up until 28. Apr; critically ill individuals hospitalised with COVID-19 diagnosis	257; median 62 (51-72), nr (nr); 67%	PCR / no	ICU mortality	Cox / age, sex, symptom duration before hospital presentation (per day), comorbidities (HTN, cardiac diseases, COPD, DM), laboratory values / age per 10 year increase / female	NOS =7	The outcome was excluded from the reporting
D'Silva; USA[177]	case-control; multi-center; 30. Jan - 8. Apr; individuals registered as patients in the health care provider's network diagnosed with COVID-19	52 cases, 104 controls; cases: nr (nr), mean 62 (15); controls: nr (nr), mean 63 (15); 69%	PCR / yes	case mortality, hospitalisa tion, ICU / intubation	Matching / model 1 (mortality): age, BMI; model 2: age, BMI, number of comorbidities; model 3: age, comorbidities (HTN, CAD, Resp, Rheuma) (model 3 included) / na	other =fair	2 estimates Rheumatological disease-Hospitalisation-OR-AMR: North-1-0 // Rheumatological disease-Case mortality-OR-AMR: North-2-0
Ebinger; USA[178]	cohort; multi-center; 8. Mar - 21. Mar; individuals registered as patients in the health care provider's network diagnosed with COVID-19	442 (model hospitalization); 214 (models intubation; model ICU admission; nr (nr), mean 53 (20); 58%	PCR / yes, partially, according to ECI, obesity is defined, no definition on the other comorbidities included in the model	hospitalisa tion, ICU admission, intubation	Log / model hospitalisation: age, sex, race, ethnicity, obesity, HTN, DM, comorbidity score (ECI), CHF, COPD or AST, ACE inhibitor use, Angiotensin receptor blocker use; 2 different models; considers interactions b/w age, sex, obesity; model intubation and model ICU admission: age, sex, 1 of the following comorbidities (obesity, HTN, DM, ECI, CHF, prior COPD or AST) / age per 10 years / female	NOS =9	15 estimates Heart failure-Hospitalisation-OR-AMR: North-4-80.7 // Diabetes-Hospitalisation-OR-AMR: North-8-37.6 // Hypertension-Hospitalisation-OR-AMR: North-5-59.3 // COPD or AST-Hospitalisation-OR-AMR: North-2-0 // Obesity/BMI>30-Hospitalisation-OR-AMR: North-7-18 // Diabetes-Intubation-OR-AMR: North-11-0 // Hypertension-Intubation-OR-AMR: North-9-0 // Obesity/BMI>30-Intubation-OR-AMR: North-10-0 // Heart failure-Intubation-OR-AMR: North-5-0 // COPD or AST-Intubation-OR-AMR: North-3-0 // Heart failure-ICU admission-OR-AMR: North-2-0 // COPD or AST-ICU admission-OR-AMR: North-2-0 // Diabetes-ICU admission-OR-AMR: North-6-0 // Hypertension-ICU admission-OR-AMR: North-6-0 // Obesity/BMI>30-ICU admission-OR-AMR: North-5-27.6
Gayam; USA[179]	cohort; single-center; 1. Mar - 9. Apr: individuals hospitalised with COVID-19 diagnosis	408; median 67 (nr), nr (nr); 57%	PCR / no	in-hospital mortality	Log / age, BMI, symptoms at presentation, laboratory values / age numeric / sex na	other =low-mode rate	The study is excluded from meta-analyses due to reporting of the risk as a unit increase in BMI index
Goyal; USA[180]	cohort; 2 hospitals; 3. Mar - 15. May; individuals hospitalised with COVID-19 diagnosis	1687; median 66 (54-77), nr (nr); 60%	PCR / yes, partially, on obesity	in-hospital mortality	Cox / age, sex, race, smoking, comorbidities (DM, HTN, COPD, AST, CKD, CAD, CHF, CANC) / na	NOS =7	4 estimates Underweight-Hospital mortality-HR-AMR: North-2-87.2 // Obesity/BMI>40-Hospital mortality-HR-AMR: North-2-0 // Overweight-Hospital mortality-HR-AMR: North-3-0 // Obesity/BMI>30-Hospital mortality-HR-AMR: North-4-0

Study	Study design; setting, number of hospitals; period; Study population	Sample size; age (median (IQR), mean (SD or CI) or % in certain age group(s)); % of males	Given definition of COVID-19/ pre-existing condition (if yes, see section 2.3 for detail)	Outcomes relevant for review	Adjustment method (Regression Model) / Covariates / Reference age and sex category	Risk of bias	Analyses the study participated in<br>[Main meta-analyses reported as: number of the resulted effect estimates the study contributed to; a meta-analysis is described as Condition-Outcome-Measure of association-Region-Total number of studies in the analysis-I^2%]
Gu, T, Mack; USA[181]	cohort; single-center; 10. Mar - 22. Apr; patients tested or treated for COVID-19 at the University of Michigan	tested cohort n=5698; positive cohort n=1119; unmatched control group n=7211, frequency-matched control group n=13,351; nr (nr), mean 53 (18); 47%	PCR / yes	hospitalisation, case mortality, ICU admission	Log / 3 models: 1) age, race/ethnicity, sex, 2) adjustment (1) +SES, and 3) adjustment (2) +comorbidity score. Model 2 is used for meta-analysis / age <50 / female; age 1 SD/female for mortality outcome	JBI=73%	20 estimates<br>Cancer-Hospitalisation-OR-AMR: North-5-17.8 // Cardiovascular disease-Hospitalisation-OR-AMR: North-4-81.1 // Diabetes-Hospitalisation-OR-AMR: North-8-37.6 // Obesity/BMI>30-Hospitalisation-OR-AMR: North-7-18 // Autoimmune condition-Hospitalisation-OR-AMR: North-1-0 // Underweight-Hospitalisation-OR-AMR: North-1-0 // Overweight-Hospitalisation-OR-AMR: North-3-0 // Chronic kidney disease-Hospitalisation-OR-AMR: North-5-81.5 // Chronic liver disease-Hospitalisation-OR-AMR: North-1-0 // Respiratory disease-Hospitalisation-OR-AMR: North-1-0 // Diabetes-Case mortality-OR-AMR: North-3-75.4 // Obesity/BMI>30-Case mortality-OR-AMR: North-2-5.6 // Autoimmune condition-Case mortality-OR-AMR: North-1-0 // Cancer-Case mortality-OR-AMR: North-2-66.7 // Chronic kidney disease-Case mortality-OR-AMR: North-3-76.9 // Chronic liver disease-Case mortality-OR-AMR: North-2-71.3 // Cardiovascular disease-Case mortality-OR-AMR: North-1-0 // Respiratory disease-Case mortality-OR-AMR: North-2-0 // Underweight-Case mortality-OR-AMR: North-1-0 // Overweight-Case mortality-OR-AMR: North-1-0
Gupta; USA[182]	cohort; multi-center, 65; 4. Mar - 4. Apr; critically ill patients diagnosed with COVID-19 in ICU	2215; 2078 for Cox regression, 1494 for mortality in mechanically ventilated patients; nr (nr), mean 60 (14); 65%	PCR / yes, partially, for some comorbidities information was provided	28-day in-hospital mortality, ICU mortality and invasive mechanical ventilation (IMV) mortality	Cox, Log / age, sex, race, BMI, comorbidities (HTN, DM, CAD, CHF, COPD, CANCact), smoking, laboratory values, symptoms, SOFA score / age 18-39 / female	NOS =9	Excluded from the meta-analyses due to population selection – critically ill patients
Harmouch; USA[183]	cohort; single-center; 1. Mar - 15. Apr; individuals hospitalised with COVID-19 diagnosis	560; median 63 (24), nr (nr); 57%	PCR / yes	in-hospital mortality, ICU admission, mechanical ventilation	Log / model mortality: age, CKD stage>=3, vascular disease, laboratory values/ na	QUIPS= low-moderate	2 estimates<br>Chronic kidney disease-Hospital mortality-OR-AMR: North-11-19.7 // Cardiovascular disease-Hospital mortality-OR-AMR: North-4-0
Harrison; USA[184]	cohort; multi-center; 20. Jan - 26. May; individuals diagnosed with COVID-19 identified from federated electronic medical records	31461; median 50 (35-63), nr (nr); 45%	PCR, ICD-10 code (% unknown),/ based on Charlson comorbidity index	case mortality	Log / age, sex, ethnicity, Charlson comorbidities / numeric age / sex female	NOS =8	13 estimates<br>Diabetes-Case mortality-OR-AMR: North-3-75.4 // Rheumatological disease-Case mortality-OR-AMR: North-2-0 // Cancer-Case mortality-OR-AMR: North-2-66.7 // Chronic kidney disease-Case mortality-OR-AMR: North-3-76.9 // Chronic liver disease-Case mortality-OR-AMR: North-2-71.3 // Respiratory disease-Case mortality-OR-AMR: North-2-0 // Heart failure-Case mortality-OR-AMR: North-1-0 // Cerebrovascular/Stroke-Case mortality-OR-AMR: North-1-0 // Dementia-Case mortality-OR-AMR: North-1-0 // HIV-Case mortality-OR-AMR: North-1-0 // Cancer/Solid-Case mortality-OR-AMR: North-1-0 // Infarction-Case mortality-OR-AMR: North-1-0 // Peripheral vascular disease-Case mortality-OR-AMR: North-1-0
Hashemi; USA[185]	cohort; multi-center, 9; 11. Mar - 2. Apr; individuals hospitalised with COVID-19 diagnosis	363; nr (nr), mean 63 (16); 55%	PCR / yes, partially, only on CLD	in-hospital mortality, ICU admission, mechanical ventilation	Log / age, sex, comorbidities (CLD, cardiac diseases, HTN, DM, hyperlipidaemia, Resp, obesity) / age na / female	NOS =8	23 estimates<br>Diabetes-Hospital mortality-OR-AMR: North-12-0 // Hypertension-Hospital mortality-OR-AMR: North-12-68.6 // Obesity/BMI>30-Hospital mortality-OR-AMR: North-11-0 // Heart disease-Hospital mortality-OR-AMR: North-1-0 // Chronic liver disease-Hospital mortality-OR-AMR: North-3-0 // Chronic liver/Cirrhosis-Hospital mortality-OR-AMR: North-2-29.1 // Chronic liver/Non-cirrhotic-Hospital mortality-OR-AMR: North-1-0 // Dyslipidemia or hyperlipidemia-Hospital mortality-OR-AMR: North-3-0 // Respiratory disease-Hospital mortality-OR-AMR: North-3-0 // Diabetes-Intubation-OR-AMR: North-11-0 // Hypertension-Intubation-OR-AMR: North-9-0 // Obesity/BMI>30-Intubation-OR-AMR: North-10-0 // Heart disease-Intubation-OR-AMR: North-2-83.5 // Chronic liver disease-Intubation-OR-AMR: North-2-48 // Dyslipidemia or hyperlipidemia-Intubation-OR-AMR: North-2-0 // Respiratory disease-Intubation-OR-AMR: North-3-0 // Diabetes-ICU admission-OR-AMR: North-6-0 // Hypertension-ICU admission-OR-AMR: North-6-0 // Obesity/BMI>30-ICU admission-OR-AMR: North-5-27.6 // Heart disease-ICU admission-OR-AMR: North-2-0 // Chronic liver disease-ICU admission-OR-AMR: North-2-2 // Dyslipidemia or hyperlipidemia-ICU admission-OR-AMR: North-1-0 // Respiratory disease-ICU admission-OR-AMR: North-3-0

Study	Study design; setting, number of hospitals; period; Study population	Sample size; age (median (IQR), mean (SD or CI) or % in certain age group(s)); % of males	Given definition of COVID-19/ pre-existing condition (if yes, see section 2.3 for detail)	Outcomes relevant for review	Adjustment method (Regression Model) / Covariates / Reference age and sex category	Risk of bias	Analyses the study participated in [Main meta-analyses reported as: number of the resulted effect estimates the study contributed to; a meta-analysis is described as Condition-Outcome-Measure of association-Region-Total number of studies in the analysis-I^2%]
Hur; USA[186]	cohort; multi-center, 10; 1. Mar - 8. Apr; individuals hospitalised with COVID-19 diagnosis	486; median 59 (47-69), nr (nr); 56%	PCR / yes, partially, for CVD and Pulmonary disease	Intubation in hospitalise d individuals	Log / age, sex, race, hospital type, BMI and comorbidities (diabetes), shortness of breath, pulse, temperature, respiratory rate / age<60 / female	NOS =9	3 estimates<br>Diabetes-Intubation-OR-AMR: North-11-0 // Obesity/BMI>30-Intubation-OR-AMR: North-10-0 // Obesity/BMI>40-Intubation-OR-AMR: North-2-6.5
Imam; USA[187]	cohort; multi-center, 8; 1. Mar - 1. Apr; individuals hospitalised with COVID-19 diagnosis	1305; nr (nr), mean 61 (16); 54%	PCR / no	in-hospital mortality	Log / age, Charlson Comorbidity Index >3 / age <60 / sex na	NOS =6	Risk “Charlson Comorbidity Index” was excluded from meta-analyses
Jun; USA[188] pre-print	cohort; multi-center, 5; 21. Mar - 28. Apr; follow-up until 3. Jun; individuals hospitalised with COVID-19 diagnosis, admitted through the emergency room	3086; median 66 (56-77), nr (nr); 60%	PCR / no	in-hospital mortality	Log / age, sex, race, comorbidities (HTN, DM, CAD, CHF, ART, CKD, COPD or AST, obesity, CANC) / age numeric/ female	JBI=7 7%	9 estimates<br>Chronic kidney disease-Hospital mortality-OR-AMR: North-11-19.7 // Diabetes-Hospital mortality-OR-AMR: North-12-0 // Hypertension-Hospital mortality-OR-AMR: North-12-68.6 // Obesity/BMI>30-Hospital mortality-OR-AMR: North-11-0 // Arrhythmia-Hospital mortality-OR-AMR: North-2-0 // Coronary artery disease-Hospital mortality-OR-AMR: North-6-10.2 // Cancer-Hospital mortality-OR-AMR: North-5-0 // Heart failure-Hospital mortality-OR-AMR: North-8-31.6 // COPD or AST-Hospital mortality-OR-AMR: North-3-0
Kabarriti; USA[189]	cohort; single-center; 14. Mar - 15. Apr; follow-up until 27. Apr; individuals diagnosed with COVID-19	5902; median 58 (44-71), nr (nr); 47%	PCR / based on the Charlson Comorbidity Index	case mortality	Cox / age, sex, race/ethnicity, socioeconomic status and comorbidities (Charlson Index: HTN, CVD, DM, CANC, liver disease, DEM, chronic pulmonary disease, peptic ulcer, hemiplegia or paraplegia, kidney disease, HIV/AIDS and in addition BMI, HTN) / age <=40 / men	NOS =8	11 estimates<br>Underweight-Case mortality-HR-AMR: North-1-0 // Obesity/BMI>30-Case mortality-HR-AMR: North-1-0 // Cancer-Case mortality-HR-AMR: North-1-0 // Chronic liver disease-Case mortality-HR-AMR: North-1-0 // Chronic kidney disease-Case mortality-HR-AMR: North-1-0 // Cardiovascular disease-Case mortality-HR-AMR: North-1-0 // Dementia-Case mortality-HR-AMR: North-1-0 // Diabetes-Case mortality-HR-AMR: North-1-0 // HIV-Case mortality-HR-AMR: North-1-0 // Hypertension-Case mortality-HR-AMR: North-1-0 // Respiratory disease-Case mortality-HR-AMR: North-1-0
Kalligeros; USA[190]	cohort; multi-center, 3; Feb. 17 - 5. Apr; individuals hospitalised with COVID-19 diagnosis	103; median 60 (50-72), nr (nr); 61%	PCR / no	ICU admission, intubation	Log / ICU admission model/intubation model: age, ethnicity, sex, BMI, comorbidities (DM, HTN, cardiac diseases, Resp, pulmonary disease or respiratory disease not defined) / age numeric / female	NOS =9	12 estimates<br>Diabetes-Intubation-OR-AMR: North-11-0 // Hypertension-Intubation-OR-AMR: North-9-0 // Obesity/BMI>30-Intubation-OR-AMR: North-10-0 // Heart disease-Intubation-OR-AMR: North-2-83.5 // Respiratory disease-Intubation-OR-AMR: North-3-0 // Overweight-Intubation-OR-AMR: North-3-21.9 // Diabetes-ICU admission-OR-AMR: North-6-0 // Hypertension-ICU admission-OR-AMR: North-6-0 // Obesity/BMI>30-ICU admission-OR-AMR: North-5-27.6 // Heart disease-ICU admission-OR-AMR: North-2-0 // Respiratory disease-ICU admission-OR-AMR: North-3-0 // Overweight-ICU admission-OR-AMR: North-2-0
Killerby; USA[191]	cohort; multi-center, 6; 1. Mar - 7. Apr; individuals diagnosed with COVID-19	531, 368 in regression; non-hospitalized: median 45 (33-58), nr (nr); hospitalized: median 61 (45-70), nr (nr); Non-hospitalized: 37%; Hospitalized: 52%	PCR / partially, on obesity, CVD, CKD	hospitalisa tion	Log / age, sex, race, smoking status, comorbidities (obesity, HTN, DM, CVD, Resp, CKD) / age 18-44 / female	NOS =6	2 estimates<br>Diabetes-Hospitalisation-OR-AMR: North-8-37.6 // Obesity/BMI>30-Hospitalisation-OR-AMR: North-7-18

Study	Study design; setting, number of hospitals; period; Study population	Sample size; age (median (IQR), mean (SD or CI) or % in certain age group(s)); % of males	Given definition of COVID-19/ pre-existing condition (if yes, see section 2.3 for detail)	Outcomes relevant for review	Adjustment method (Regression Model) / Covariates / Reference age and sex category	Risk of bias	Analyses the study participated in [Main meta-analyses reported as: number of the resulted effect estimates the study contributed to; a meta-analysis is described as Condition-Outcome-Measure of association-Region-Total number of studies in the analysis-I^2%]
Kim.L; USA[192]	cohort; multi-center, 154; 1. Mar - 2. May; individuals hospitalised with COVID-19 diagnosis	2491; median 62 (50–75), nr (nr); 53%	PCR / yes	ICU admission, in-hospital mortality	Poisson / model ICU admission 1: age, sex, race/ethnicity, smoker, comorbidities (BMI>30, CKD, DM, HTN, Immun, neurologic disease, Resp, CVD), medication; model in-hospital mortality 2: age, sex, race/ethnicity, smoker, comorbidities (chronic neurological disorder, CKD, CVD, DM, HTN, Immun, obesity, Resp, Rheuma, hematologic), drugs; model ICU admission 3: age, sex, race/ethnicity, number of underlying medical conditions ; model in-hospital mortality 4: age, sex, race/ethnicity, nr of underlying medical conditions / age 18–39 years / female	NOS =9	17 estimates<br>Neurological disease-Hospital mortality-RR-AMR: North-1-0 // Chronic kidney disease-Hospital mortality-RR-AMR: North-1-0 // Cardiovascular disease-Hospital mortality-RR-AMR: North-1-0 // Diabetes-Hospital mortality-RR-AMR: North-3-0 // Hypertension-Hospital mortality-RR-AMR: North-2-54.5 // Immunosuppression-Hospital mortality-RR-AMR: North-1-0 // Obesity/BMI>30-Hospital mortality-RR-AMR: North-2-15.9 // Respiratory disease-Hospital mortality-RR-AMR: North-1-0 // Rheumatological disease-Hospital mortality-RR-AMR: North-1-0 // Obesity/BMI>30-ICU admission-RR-AMR: North-1-0 // Chronic kidney disease-ICU admission-RR-AMR: North-1-0 // Diabetes-ICU admission-RR-AMR: North-1-0 // Hypertension-ICU admission-RR-AMR: North-1-0 // Immunosuppression-ICU admission-RR-AMR: North-1-0 // Neurological disease-ICU admission-RR-AMR: North-1-0 // Respiratory disease-ICU admission-RR-AMR: North-1-0 // Cardiovascular disease-ICU admission-RR-AMR: North-1-0
Klang; USA[193]	cohort; multi-center, 5; 1. Mar - 17. May; individuals hospitalised with COVID-19 diagnosis with data available on BMI	2834; survivors: median 68 (60-77), nr (nr); nonsurvivors: median 76 (67-84), nr (nr); 54% of survivors and 57% of nonsurvivors	PCR / yes, partially, for obesity	in-hospital mortality	Log / models estimated for age groups >50 and <=50 (age, sex, ethnicity, comorbidities (obesity, CAD, CANC, CHF, CKD, HTN, hyperlipidemia) / age decile / female	NOS =8	9 estimates<br>Chronic kidney disease-Hospital mortality-OR-AMR: North-11-19.7 // Diabetes-Hospital mortality-OR-AMR: North-12-0 // Hypertension-Hospital mortality-OR-AMR: North-12-68.6 // Obesity/BMI>30-Hospital mortality-OR-AMR: North-11-0 // Dyslipidemia or hyperlipidemia-Hospital mortality-OR-AMR: North-3-0 // Coronary artery disease-Hospital mortality-OR-AMR: North-6-10.2 // Cancer-Hospital mortality-OR-AMR: North-5-0 // Heart failure-Hospital mortality-OR-AMR: North-8-31.6 // Obesity/BMI>40-Hospital mortality-OR-AMR: North-2-0<br>and<br>Analysis of age-stratified estimates
Lieberman-Cribbin; USA[194]	cohort; multi-center; 29. Feb - 24. Apr; individuals hospitalised with COVID-19 diagnosis	6245; patients without/with COVID-19: nr (nr), mean 57 (33); only reported for sample of patients without/with COVID-19: 49%	PCR / asthma	in-hospital mortality	Log / age, sex, race, comorbidities (AST) / age<40 / male	QUIPS= low-moderate	1 estimate<br>Asthma-Hospital mortality-OR-AMR: North-3-0
Magleby; USA[195]	cohort; 2 hospitals; 30. Mar - 30. Apr; individuals hospitalised with COVID-19 diagnosis	678; three cycle threshold (CT)-value groups: median 72 (60-81), nr (nr); median 69 (58-79), nr (nr); median 63 (50-73), nr (nr); 3 CT-value groups: 63%; 61%, 59%	PCR / yes, partially, only for obesity	Intubation, in-hospital mortality	Log / model mortality: age, white race, comorbidities (CAD, CHF, COPD, CRB, HTN), use of oral steroids as an outpatient, days of symptoms prior to admission, symptoms on admission, highest level of supplemental oxygen within 3 hours of arrival to the ED, chest xray findings, viral load by nasal pharyngeal swab; model intubation: age, race, obesity, use of steroids as an outpatient, symptoms on admission, chest xray findings, viral load by nasal pharyngeal swab / age numeric / sex na	NOS =8	6 estimates<br>COPD-Hospital mortality-OR-AMR: North-5-37.9 // Hypertension-Hospital mortality-OR-AMR: North-12-68.6 // Coronary artery disease-Hospital mortality-OR-AMR: North-6-10.2 // Heart failure-Hospital mortality-OR-AMR: North-8-31.6 // Cerebrovascular/Stroke-Hospital mortality-OR-AMR: North-3-0 // Obesity/BMI>30-Intubation-OR-AMR: North-10-0
Mahdavinia; USA[196]	cohort; electronic database; 12. Mar - 3. Apr; patients with COVID-19 diagnosis selected from acute care hospitals and outpatient clinics	935; nr (nr), nr (nr); with AST: 67%; without AST: 51%	PCR / no	hospitalisation, mechanical intubation, case mortality	Stratification / AST / Age groups: 18-49, 50-64, >65 / sex na	other =high	Analysis of age-stratified estimates – asthma

Study	Study design; setting, number of hospitals; period; Study population	Sample size; age (median (IQR), mean (SD or CI) or % in certain age group(s)); % of males	Given definition of COVID-19/ pre-existing condition (if yes, see section 2.3 for detail)	Outcomes relevant for review	Adjustment method (Regression Model) / Covariates / Reference age and sex category	Risk of bias	Analyses the study participated in [Main meta-analyses reported as: number of the resulted effect estimates the study contributed to; a meta-analysis is described as Condition-Outcome-Measure of association-Region-Total number of studies in the analysis-I^2%]
McCarty; USA[197]	cohort; multi-center, 9; 22. Mar - 2. Apr; individuals hospitalised with COVID-19 diagnosis	379; nr (nr), mean 63 (16); 56%	PCR / yes, partially, on history of cardiac disease, pulmonary disease, obesity; no info on HTN and DM	in-hospital mortality, ICU admission, mechanical ventilation	Log / age, sex, race/ethnicity, comorbidities (obesity, CVD, Resp, HTN, DM) / age na/ female	JBI=7 3%	15 estimates Diabetes-Hospital mortality-OR-AMR: North-12-0 // Hypertension-Hospital mortality-OR-AMR: North-12-68.6 // Obesity/BMI>30-Hospital mortality-OR-AMR: North-11-0 // Cardiovascular disease-Hospital mortality-OR-AMR: North-4-0 // Respiratory disease-Hospital mortality-OR-AMR: North-3-0 // Diabetes-Intubation-OR-AMR: North-11-0 // Hypertension-Intubation-OR-AMR: North-9-0 // Obesity/BMI>30-Intubation-OR-AMR: North-10-0 // Respiratory disease-Intubation-OR-AMR: North-3-0 // Cardiovascular disease-Intubation-OR-AMR: North-1-0 // Diabetes-ICU admission-OR-AMR: North-6-0 // Hypertension-ICU admission-OR-AMR: North-6-0 // Obesity/BMI>30-ICU admission-OR-AMR: North-5-27.6 // Respiratory disease-ICU admission-OR-AMR: North-3-0 // Cardiovascular disease-ICU admission-OR-AMR: North-1-0
Mehta; USA[198]	cohort; single-center; 18. Mar - 8. Apr; individuals with a malignant diagnosis with COVID-19	218; median 69 (nr), nr (nr); 58%	PCR / no	case mortality	Log / age, sex, ICU admission, laboratory values, comorbidity score (DM, HTN, Resp, chronic kidney disease, CAD and CHF capped at a maximum of 3) / age>65 / sex na	NOS =7	Analysis of evidence for specific population groups – patients with cancer
Mendy; USA[199] pre-print	cohort; multi-center, 4; 13. Mar - 31. May; COVID-19 patients diagnosed at the University of Cincinnati health system	689; median 49 (35-67), nr (nr); 53%	PCR / yes, ICD-10 for all comorbidities	hospitalisation, ICU admission/ death	Log / age, sex, race/ethnicity, smoker, comorbidities (pure hypercholesteromia, AST, COPD, CKD, CVD, neoplasm or history of neoplasm, DM, osteoarthritis, vitamin D deficiency, obesity), hematological disorders / age (per 10 years increase) / female	NOS =7	8 estimates Asthma-Hospitalisation-OR-AMR: North-3-66.6 // Cancer-Hospitalisation-OR-AMR: North-5-17.8 // COPD-Hospitalisation-OR-AMR: North-4-15.2 // Cardiovascular disease-Hospitalisation-OR-AMR: North-4-81.1 // Diabetes-Hospitalisation-OR-AMR: North-8-37.6 // Obesity/BMI>30-Hospitalisation-OR-AMR: North-7-18 // Chronic kidney disease-Hospitalisation-OR-AMR: North-5-81.5 // Dyslipidemia or hyperlipidemia-Hospitalisation-OR-AMR: North-2-91.6
Mikami; USA[200]	cohort; multi-center, 8 + 400 ambulant; 13. Mar - 17. Apr; individuals hospitalised with COVID-19 diagnosis	2820; median 66 (55-78), nr (nr); 57%	PCR / no	in-hospital mortality	Cox / age, sex, race, smoking, comorbidities (HTN, diabetes, or CANC), vital signs, BMI, initial laboratory values (lymphocyte proportion, D -dimer IL-6), and hydroxychloroquine use / age < 50 / male	NOS =7	3 estimates Diabetes-Hospital mortality-HR-AMR: North-5-0 // Hypertension-Hospital mortality-HR-AMR: North-4-0 // Cancer-Hospital mortality-HR-AMR: North-4-0
Miyashita (a); USA[201]	cohort; multi-center, 8 + more than 400 ambulant; 1. Mar - 30. Apr; follow up until 1. May; individuals diagnosed with COVID-19	total n=8912, in analysis n=3992: n for ICU admission=1982, for Intubation n=752, for death n=1258; ICU admission: 49%>=66; Intubation: 501% >=66; death: 74%>=66; nr	PCR / no	case mortality, risk of intubation, risk of ICU admission	Stratification / age, HIV / na	JBI=7 5%	Analysis of age-stratified estimates – HIV
Miyashita (b); USA[202]	cohort; multi-center; 1. Mar - 6. Apr; individuals diagnosed with COVID-19	5688; Intubation: 14%>=81; death: 36%>=81; nr	PCR / no	case mortality, risk of intubation	Stratification / age, CANC / na	QUIPS= low-moderate-high	Analysis of age-stratified estimates – cancer
Miyashita (c); USA[203]	cohort; electronic database; 1. Mar - 2. Apr; individuals >=60 years diagnosed with COVID-19	2071; with DEM 68%>80; without DEM 23% >80; with DEM: 44%, without DEM: 51%	PCR / no	case mortality, ICU admission, mechanical ventilation	Stratification / Dementia, age 60-79, >80 / sex na	NOS =7	Analysis of age-stratified estimates – dementia

Study	Study design; setting, number of hospitals; period; Study population	Sample size; age (median (IQR), mean (SD or CI) or % in certain age group(s)); % of males	Given definition of COVID-19/ pre-existing condition (if yes, see section 2.3 for detail)	Outcomes relevant for review	Adjustment method (Regression Model) / Covariates / Reference age and sex category	Risk of bias	**Analyses the study participated in**<br>[Main meta-analyses reported as: number of the resulted effect estimates the study contributed to; a meta-analysis is described as Condition-Outcome-Measure of association-Region-Total number of studies in the analysis-I^2%]
Nakeshbandi; USA[204]	cohort; single-center; 10. Mar - 13. Apr; individuals hospitalised with COVID-19 diagnosis	504; nr (nr), mean 68 (15); 52%	PCR / yes, partially, only for obesity	in-hospital mortality (30-day)	Log / age, sex, comorbidities (HTN, DM, BMI), qSOFA; subgroup analysis / age<45 / female	NOS =4	8 estimates<br>Diabetes-Hospital mortality-RR-AMR: North-3-0 // Hypertension-Hospital mortality-RR-AMR: North-2-54.5 // Obesity/BMI>30-Hospital mortality-RR-AMR: North-2-15.9 // Overweight-Hospital mortality-RR-AMR: North-1-0 // Obesity/BMI>30-Intubation-RR-AMR: North-2-10.7 // Overweight-Intubation-RR-AMR: North-1-0 // Diabetes-Intubation-RR-AMR: North-1-0 // Hypertension-Intubation-RR-AMR: North-1-0<br>and<br>Analysis of age-stratified estimates
Narain; USA[205]	cohort; multi-center, 12; 1. Mar - 24. Apr; individuals hospitalised with COVID-19 diagnosis	5776; nr (nr), mean 62 (nr)-67 (nr); 61%-72% reported by treatment groups	PCR / no	in-hospital mortality	Cox / age, sex, race/ethnicity, smoking, insurance, treatment site, laboratory findings, comorbidities (AST, COPD, CLD, DM, HTN, ILD, Auto, CVD, CKD, CANC, BMI), haemodialysis, CCI, intubation / age na/ female	JBI=68%	13 estimates<br>Chronic kidney disease-Hospital mortality-HR-AMR: North-4-71.9 // Diabetes-Hospital mortality-HR-AMR: North-5-0 // Hypertension-Hospital mortality-HR-AMR: North-4-0 // Underweight-Hospital mortality-HR-AMR: North-2-87.2 // Overweight-Hospital mortality-HR-AMR: North-3-0 // Obesity/BMI>30-Hospital mortality-HR-AMR: North-4-0 // Cancer-Hospital mortality-HR-AMR: North-4-0 // Asthma-Hospital mortality-HR-AMR: North-2-79.1 // Autoimmune condition-Hospital mortality-HR-AMR: North-1-0 // Chronic liver disease-Hospital mortality-HR-AMR: North-1-0 // COPD-Hospital mortality-HR-AMR: North-3-0 // Cardiovascular disease-Hospital mortality-HR-AMR: North-1-0 // Interstitial lung disease-Hospital mortality-HR-AMR: North-1-0
Okoh; USA[206]	cohort; single-center; 10. Mar - 10. Apr; individuals (Black/African American and Latino Hispanic) hospitalised with COVID-19	251; median 62 (49-74), nr (nr); 51%	PCR / no	in-hospital mortality	Log / age, HIV, laboratory findings / age numeric/ female	NOS =6	1 estimate<br>HIV-Hospital mortality-OR-AMR: North-1-0
Palaiodimos; USA[207]	cohort; single-center; 9. Mar - 22. Mar; follow-up until 12. Apr; individuals hospitalised with COVID-19 diagnosis admitted through the emergency room	200; median 64 (50–73), nr (nr); 49%	PCR / yes, partially, BMI categories provided	in-hospital mortality, intubation	Log / model in-hospital mortality: sex, age, comorbidities (BMI<25vs25-34, BMI>=35vs25-34, CAD, CHF, CKD, COPD, DM), smoker; model intubation: sex, age, comorbidities (BMI<25vs25-34, BMI>=35vs25-34, DM, hyperlipidemia, obstructive sleep apnea), smoker / age (quartiles)/female	NOS =9	12 estimates<br>Chronic kidney disease-Hospital mortality-OR-AMR: North-11-19.7 // COPD-Hospital mortality-OR-AMR: North-5-37.9 // Diabetes-Hospital mortality-OR-AMR: North-12-0 // Obesity/BMI>30-Hospital mortality-OR-AMR: North-11-0 // Coronary artery disease-Hospital mortality-OR-AMR: North-6-10.2 // Heart failure-Hospital mortality-OR-AMR: North-8-31.6 // Underweight-Hospital mortality-OR-AMR: North-1-0 // Diabetes-Intubation-OR-AMR: North-11-0 // Obesity/BMI>30-Intubation-OR-AMR: North-10-0 // Dyslipidemia or hyperlipidemia-Intubation-OR-AMR: North-2-0 // Underweight-Intubation-OR-AMR: North-1-0 // Obstructive sleep apnea-Intubation-OR-AMR: North-1-0
Patel, N; USA[208] pre-print	cohort; multi-center, 10; 9. Mar - 26. Jun; individuals on anticoagulation treatment hospitalised with COVID-19 diagnosis	1716; 48%>60; 54%	ICD-10 code / ICD-10 code / yes, CCI score was used, BMI	intubation	Log / age, sex, race, BMI, CCI, glucose on admission, use of antiplatelet agents / age 45-59 / female	JBI=73%	Analysis of evidence for specific population groups – patients on anticoagulation treatment
Petrilli; USA[209]	cohort; single-center; 1. Mar - 8. Apr; follow-up until 5. May; individuals with COVID-19 diagnosis /individuals hospitalised	5279 tested positive (model hospitalisation) ; 2737 inpatients (model death); median 54 (38-66), nr (nr); 49%	PCR / yes, partially, only BMI categories	in-hospital mortality, hospitalisation	Log, competing risk models: in-hospital mortality /hospitalisation: time variable, age, sex, race/ethnicity, smoking, comorbidities (obesity, CAD, CHF, CKD, COPD or AST, DM, HTN, hyperlipidemia, CANC) / age 19-44 / female	NOS =8	22 estimates<br>Cancer-Hospitalisation-OR-AMR: North-5-17.8 // Heart failure-Hospitalisation-OR-AMR: North-4-80.7 // Diabetes-Hospitalisation-OR-AMR: North-8-37.6 // Hypertension-Hospitalisation-OR-AMR: North-5-59.3 // COPD or AST-Hospitalisation-OR-AMR: North-2-0 // Obesity/BMI>30-Hospitalisation-OR-AMR: North-7-18 // Overweight-Hospitalisation-OR-AMR: North-3-0 // Chronic kidney disease-Hospitalisation-OR-AMR: North-5-81.5 // Dyslipidemia or hyperlipidemia-Hospitalisation-OR-AMR: North-2-91.6 // Obesity/BMI>40-Hospitalisation-OR-AMR: North-1-0 // Coronary artery disease-Hospitalisation-OR-AMR: North-2-0 // Coronary artery disease-Hospital mortality-HR-AMR: North-3-0 // Heart failure-Hospital mortality-HR-AMR: North-3-87.2 // Chronic kidney disease-Hospital mortality-HR-AMR: North-4-71.9 // Diabetes-Hospital mortality-HR-AMR: North-5-0 // Hypertension-Hospital mortality-HR-AMR: North-4-0 // Obesity/BMI>40-Hospital mortality-HR-AMR: North-2-0 // Overweight-Hospital mortality-HR-AMR: North-3-0 // Obesity/BMI>30-Hospital mortality-HR-AMR: North-4-0 // Cancer-Hospital mortality-HR-AMR: North-4-0 // COPD or AST-Hospital mortality-HR-AMR: North-1-0 // Dyslipidemia or hyperlipidemia-Hospital mortality-HR-AMR: North-1-0

Study	Study design; setting, number of hospitals; period; Study population	Sample size; age (median (IQR), mean (SD or CI) or % in certain age group(s)); % of males	Given definition of COVID-19/ pre-existing condition (if yes, see section 2.3 for detail)	Outcomes relevant for review	Adjustment method (Regression Model) / Covariates / Reference age and sex category	Risk of bias	Analyses the study participated in [Main meta-analyses reported as: number of the resulted effect estimates the study contributed to; a meta-analysis is described as Condition-Outcome-Measure of association-Region-Total number of studies in the analysis-I^2%]
Pettit; USA[210]	cohort; single-center; 1. Mar - 18. Apr; individuals hospitalised with COVID-19 diagnosis	238; nr (nr), mean 58 (17); 47%	PCR / yes, partially, on obesity, cardiovascular disease and pulmonary disease only	in-hospital mortality	Log / age, sex, comorbidities (CANC, CKD, CRB, CVD, DM, HTN, hyperlipidemia, obesity, Resp, venous thromboembolism) / numeric age / female	NOS =9	10 estimates Chronic kidney disease-Hospital mortality-OR-AMR: North-11-19.7 // Diabetes-Hospital mortality-OR-AMR: North-12-0 // Hypertension-Hospital mortality-OR-AMR: North-12-68.6 // Obesity/BMI>30-Hospital mortality-OR-AMR: North-11-0 // Cardiovascular disease-Hospital mortality-OR-AMR: North-4-0 // Dyslipidemia or hyperlipidemia-Hospital mortality-OR-AMR: North-3-0 // Respiratory disease-Hospital mortality-OR-AMR: North-3-0 // Cancer-Hospital mortality-OR-AMR: North-5-0 // Cerebrovascular/Stroke-Hospital mortality-OR-AMR: North-3-0 // V.thromboembolism-Hospital mortality-OR-AMR: North-2-66.3
Price-Haywood; USA[211]	cohort; multi-center; 1. Mar - 11. Apr; follow-up until 7. May; health services members diagnosed with COVID-19	3481; White Non-Hispanic: ne (nr), mean 55 (18); Black Non-Hispanic: nr (nr), mean 54 (16); White Non-Hispanic: 46%; Black Non-Hispanic: 38%	PCR / yes partially, obesity and CCI are defined	hospitalisation, in-hospital mortality	Cox / 3 models: 1) race only 2) race, age, sex, 3) race, age, sex, CCI, obesity, SES. Model 3 is included / age 5-yr units / female	JBI=91%	2 estimates Obesity/BMI>30-Hospital mortality-OR-AMR: North-11-0 // Obesity/BMI>30-Hospitalisation-OR-AMR: North-7-18
Rentsch; USA[212] pre-print	cohort; electronic database; 8. Feb - 30. Mar; individuals (veterans, 90% male, aged 54-75 years old) with COVID-19 diagnosis	585; median 66 (60-71), nr (nr); 95%	PCR / yes	case ICU, hospitalisation	Log / age, race/ethnicity, medication history, vital signs, laboratory values, comorbidities (CKD, COPD, DM, HTN, vascular disease) / age 5-yr increase/sex na	JBI=88%	5 estimates COPD-Hospitalisation-OR-AMR: North-4-15.2 // Cardiovascular disease-Hospitalisation-OR-AMR: North-4-81.1 // Diabetes-Hospitalisation-OR-AMR: North-8-37.6 // Hypertension-Hospitalisation-OR-AMR: North-5-59.3 // Chronic kidney disease-Hospitalisation-OR-AMR: North-5-81.5
Robilotti; USA[213]	cohort; single-center; 10. Mar - 7. Apr; patients with cancer diagnosed with COVID-19	423; 411 in regression; 56%>60; 50%	PCR / yes, partially, for cancer solid, cancer hematologic, cardiac dysfunction only; no further definitions for metastastic disease, asthma, or for COPD, DM, CKD, HTN	hospitalisation, severe respiratory illness	Log / age, race, smoking, comorbidities (COPD or AST, cancer (metastatic solid), cancer (hematologic), cardiac disorder, HTN or CKD) chronic lymphopenia or corticosteroids, treatment with immune check-point inhibitors / age<65 / sex na	NOS =5	Analysis of evidence for specific population groups – patients with cancer
Salacup; USA[214]	cohort; single-center; 1. Mar -24. Apr; individuals (70% African-American) hospitalised with COVID-19	242; nr (nr), mean 66 (15); 50%	PCR / no	in-hospital mortality, intubation	Log / age, sex, race, BMI, comorbidities (COPD or AST, DM, HTN, CHF, CLD/cirrhosis, CKD) / age numeric / male	NOS =7	12 estimates Chronic kidney disease-Hospital mortality-OR-AMR: North-11-19.7 // Diabetes-Hospital mortality-OR-AMR: North-12-0 // Hypertension-Hospital mortality-OR-AMR: North-12-68.6 // Chronic liver/Cirrhosis-Hospital mortality-OR-AMR: North-2-29.1 // Heart failure-Hospital mortality-OR-AMR: North-8-31.6 // COPD or AST-Hospital mortality-OR-AMR: North-3-0 // Chronic kidney disease-Intubation-OR-AMR: North-5-72.5 // Diabetes-Intubation-OR-AMR: North-11-0 // Hypertension-Intubation-OR-AMR: North-9-0 // Heart failure-Intubation-OR-AMR: North-5-0 // COPD or AST-Intubation-OR-AMR: North-3-0 // Chronic liver/Cirrhosis-Intubation-OR-AMR: North-1-0

Study	Study design; setting, number of hospitals; period;<br>Study population	Sample size; age (median (IQR), mean (SD or CI) or % in certain age group(s)); % of males	Given definition of COVID-19/ pre-existing condition (if yes, see section 2.3 for detail)	Outcomes relevant for review	Adjustment method (Regression Model) / Covariates / Reference age and sex category	Risk of bias	**Analyses the study participated in**<br>[Main meta-analyses reported as: number of the resulted effect estimates the study contributed to; a meta-analysis is described as Condition-Outcome-Measure of association-Region-Total number of studies in the analysis-I^2%]
Seiglie; USA[215]	cohort; single-center; 11. Mar - 30. Apr;<br>individuals hospitalised with COVID-19 diagnosis	450; without DM: nr (nr), mean 61 (19); with DM: nr (nr), mean 67 (14); 55% group without DM; 62% group with DM	PCR / yes, partially, on BMI and DM, not on the other comorbidities	in-hospital mortality, ICU admission, intubation (14-day outcomes)	Log / age, sex, race, BMI, comorbidities (DM, CAD or Myo, CHF, HTN, COPD or AST, CANCact, CLD, CKD) / age <50 / female	NOS =9	27 estimates<br>Chronic kidney disease-Hospital mortality-OR-AMR: North-11-19.7 // Diabetes-Hospital mortality-OR-AMR: North-12-0 // Hypertension-Hospital mortality-OR-AMR: North-12-68.6 // Obesity/BMI>30-Hospital mortality-OR-AMR: North-11-0 // Chronic liver disease-Hospital mortality-OR-AMR: North-3-0 // Heart failure-Hospital mortality-OR-AMR: North-8-31.6 // COPD or AST-Hospital mortality-OR-AMR: North-3-0 // Overweight-Hospital mortality-OR-AMR: North-2-0 // Cancer/Active-Hospital mortality-OR-AMR: North-1-0 // Chronic kidney disease-Intubation-OR-AMR: North-5-72.5 // Diabetes-Intubation-OR-AMR: North-11-0 // Hypertension-Intubation-OR-AMR: North-9-0 // Obesity/BMI>30-Intubation-OR-AMR: North-10-0 // Heart failure-Intubation-OR-AMR: North-5-0 // COPD or AST-Intubation-OR-AMR: North-3-0 // Chronic liver disease-Intubation-OR-AMR: North-2-48 // Overweight-Intubation-OR-AMR: North-3-21.9 // Cancer/Active-Intubation-OR-AMR: North-1-0 // Heart failure-ICU admission-OR-AMR: North-2-0 // COPD or AST-ICU admission-OR-AMR: North-2-0 // Diabetes-ICU admission-OR-AMR: North-6-0 // Hypertension-ICU admission-OR-AMR: North-6-0 // Obesity/BMI>30-ICU admission-OR-AMR: North-5-27.6 // Chronic liver disease-ICU admission-OR-AMR: North-2-2 // Overweight-ICU admission-OR-AMR: North-2-0 // Cancer/Active-ICU admission-OR-AMR: North-1-0 // Chronic kidney disease-ICU admission-OR-AMR: North-2-76.9
Shah, P; USA[216]	cohort; electronic database; 2. Mar - 6. May;<br>individuals hospitalised with COVID-19 diagnosis	522; median 63 (50-72), nr (nr); 42%	PCR / yes, partially, only for BMI, not for any other comorbidities	in-hospital mortality	Log / age (2 groups), sex, race,smoking, comorbidities: HTN, CAD, CHF, COPD, AST, CKD, DM, immunosupression, liver disease, CANC, BMI (3 groups) / age<65 / male	NOS =7	12 estimates<br>Chronic kidney disease-Hospital mortality-OR-AMR: North-11-19.7 // COPD-Hospital mortality-OR-AMR: North-5-37.9 // Diabetes-Hospital mortality-OR-AMR: North-12-0 // Hypertension-Hospital mortality-OR-AMR: North-12-68.6 // Obesity/BMI>30-Hospital mortality-OR-AMR: North-11-0 // Chronic liver disease-Hospital mortality-OR-AMR: North-3-0 // Coronary artery disease-Hospital mortality-OR-AMR: North-6-10.2 // Cancer-Hospital mortality-OR-AMR: North-5-0 // Heart failure-Hospital mortality-OR-AMR: North-8-31.6 // Obesity/BMI>40-Hospital mortality-OR-AMR: North-2-0 // Asthma-Hospital mortality-OR-AMR: North-3-0 // Immunosuppression-Hospital mortality-OR-AMR: North-1-0
Sigel; USA[217]	cohort; multi-center, 5; 12. Mar - 23. Apr;<br>individuals hospitalised with COVID-19 diagnosis	493, cases with HIV: 88, without HIV: 405; cases with HIV: median 61 (54-67), nr (nr); without HIV: median 60 (55-67), nr (nr); 75%	PCR / yes, partially, info for HIV, but not on COPD	in-hospital mortality	Competing risk model / age, sex, race/ethnicity, COVID-19 severity, COPD, HIV, smoking, laboratory values / age numeric / female	JBI=6	2 estimates<br>HIV-Hospital mortality-HR-AMR: North-2-52.9 // COPD-Hospital mortality-HR-AMR: North-3-0
Singh(a); USA[218] pre-print	cohort; electronic database; 20. Jan - 16. May;<br>health services members diagnosed with COVID-19	Obesity group (n = 2459); Control group (n = 2459); nr (nr), mean 51 (19); 45%	PCR / yes, partially, only for BMI, not for any other comorbidities	case mortality, hospitalisation	Matching / all models: age, race, ethnicity, comorbidities used for matching; comorbidity of interest in analysis: obesity / na	NOS =8	2 estimates<br>Obesity/BMI>30-Hospitalisation-RR- AMR: North-2-0 // Obesity/BMI>30- Case mortality -RR- AMR: North-2-0
Singh(b); USA[219]	cohort; electronic database; 20. Jan - 12. April;<br>health services members diagnosed with COVID-19	250 with liver disease, 250 without liver disease; LD group: nr (nr), mean 55 (14); non-LD group: nr (nr), mean 57 (15); 44%	PCR / yes, partially, on liver disease, not on any other comorbidities	hospitalisation, case mortality	Matching / age, race, nicotine use, CLD, BMI, HTN, and diabetes / na	other =good	2 estimates<br>Chronic liver disease -Hospitalisation-RR- AMR: North-1-0 // Chronic liver disease-Case mortality -RR- AMR: North-1-0
Singh(c); USA[220]	cohort; electronic database; 20. Jan - 26. May;<br>health services members diagnosed with COVID-19	232 with IBD, 232 without IBD; IBD group: nr (nr), mean 51 (18); no IBD group: nr (nr), mean 51 (19); 37% group with IBD; 36% without IBD	PCR / no	hospitalisation	Matching / IBD as variable of interest; matching for demographics and comorbid conditions (age, sex, race, BMI, HTN, AST and COPD, DM, CKD, CHF, CRB, nicotine dependence, alcohol-related disorders) / na	other =good	1 estimate<br>Inflammatory bowel disease-Hospitalisation-RR- AMR: North-1-0

Study	Study design; setting, number of hospitals; period; Study population	Sample size; age (median (IQR), mean (SD or CI) or % in certain age group(s)); % of males	Given definition of COVID-19/ pre-existing condition (if yes, see section 2.3 for detail)	Outcomes relevant for review	Adjustment method (Regression Model) / Covariates / Reference age and sex category	Risk of bias	Analyses the study participated in [Main meta-analyses reported as: number of the resulted effect estimates the study contributed to; a meta-analysis is described as Condition-Outcome-Measure of association-Region-Total number of studies in the analysis-I²%]
Smith; USA[221]	cohort; multi-center, 4; 1. Mar - 22. Apr; individuals hospitalised with COVID-19 diagnosis	346; nr (nr), mean 67 (65–69); 56%	PCR / no	in-hospital mortality	Log / age, COPD, DM, hyperlipidemia / age<60 / sex na	NOS =7	3 estimates<br>Diabetes-Hospital mortality-RR-AMR: North-3-0 // COPD-Hospital mortality-RR-AMR: North-1-0 // Dyslipidemia or hyperlipidemia-Hospital mortality-RR-AMR: North-1-0
Suleyman; USA[222]	case series; multi-center, 5; 9. Mar - 27. Mar; individuals with COVID-19 diagnosis evaluated at emergency departments or hospitals	463; nr (nr), mean 57 (17); 44%	PCR / yes, partially, only for obesity, not for any other comorbidities	ICU admission, intubation	Log / model ICU admission: age, age, race, comorbidities (severe obesity, CKD, CANC, DM, HTN, CAD); model Intubation: same variables as in model ICU admission, in addition CHF and tobacco use / age<=60 and female	NOS =6	13 estimates<br>Coronary artery disease-Intubation-OR-AMR: North-3-18.1 // Chronic kidney disease-Intubation-OR-AMR: North-5-72.5 // Diabetes-Intubation-OR-AMR: North-11-0 // Hypertension-Intubation-OR-AMR: North-9-0 // Heart failure-Intubation-OR-AMR: North-5-0 // Obesity/BMI>40-Intubation-OR-AMR: North-2-6.5 // Cancer-Intubation-OR-AMR: North-2-86.9 // Diabetes-ICU admission-OR-AMR: North-6-0 // Hypertension-ICU admission-OR-AMR: North-6-0 // Chronic kidney disease-ICU admission-OR-AMR: North-2-76.9 // Coronary artery disease-ICU admission-OR-AMR: North-1-0 // Cancer-ICU admission-OR-AMR: North-1-0 // Obesity/BMI>40-ICU admission-OR-AMR: North-1-0
Tartof; USA[223]	cohort; electronic database; 13. Feb - 2. May; health services members diagnosed with COVID-19	6916; median 49 (nr), nr (nr); 45%	PCR (82%) / no information or unclear (18%) / yes, CCI used; DM defined and BMI categories given	case mortality	Poisson / age, sex, race/ethnicity, smoking, comorbidities (CCI: CAN, hyperlipidemia, Myo, Immun, immunodeficiency or immunosuppressed state, Organ, organ transplant history, CHF, PVD, cerebrovascular disease, Resp, pulmonary disease or respiratory disease not defined, renal disease, HTN, AST, DM status (with hemoglobin A1c level <7.5%; with hemoglobin A1c level >=7.5%; No DM), BMI, time variable (in weeks from week of March 22 to April 26) / age 0-40 / female	JBI=91%	17 estimates<br>Obesity/BMI>30-Case mortality-RR-AMR: North-2-0 // Asthma-Case mortality-RR-AMR: North-1-0 // Underweight-Case mortality-RR-AMR: North-1-0 // Obesity/BMI>40-Case mortality-RR-AMR: North-1-0 // Overweight-Case mortality-RR-AMR: North-1-0 // Cancer-Case mortality-RR-AMR: North-1-0 // Heart failure-Case mortality-RR-AMR: North-1-0 // Chronic kidney disease-Case mortality-RR-AMR: North-1-0 // Cerebrovascular/Stroke-Case mortality-RR-AMR: North-1-0 // Diabetes-Case mortality-RR-AMR: North-1-0 // Hypertension-Case mortality-RR-AMR: North-1-0 // Dyslipidemia or hyperlipidemia-Case mortality-RR-AMR: North-1-0 // Immunosuppression-Case mortality-RR-AMR: North-1-0 // Infarction-Case mortality-RR-AMR: North-1-0 // Organ transplant recipients-Case mortality-RR-AMR: North-1-0 // Peripheral vascular disease-Case mortality-RR-AMR: North-1-0 // Respiratory disease-Case mortality-RR-AMR: North-1-0
van Gerwen; USA[224]	cohort; health system; 1. Mar -1. Apr; individuals identified via the electronic medical record system across a large New York City health system diagnosed with COVID-19	3703, hospitalised 2015; nr (nr), mean 57 (18); 55%	PCR / yes, partially, only for BMI, not for any other comorbidities	hospitalisation, in-hospital mortality, mechanical ventilation	Log / age, sex, race, BMI, smoking, comorbidities (HTN, CAD, ART, CHF, peripheral vascular disease, CVA/TIA, DEM, DM, hypothyroidism, CKD, CANC, AST, COPD, VTE) / age 18-40 / female	NOS =7	45 estimates<br>Chronic kidney disease-Hospital mortality-OR-AMR: North-11-19.7 // COPD-Hospital mortality-OR-AMR: North-5-37.9 // Diabetes-Hospital mortality-OR-AMR: North-12-0 // Hypertension-Hospital mortality-OR-AMR: North-12-68.6 // Obesity/BMI>30-Hospital mortality-OR-AMR: North-11-0 // Arrhythmia-Hospital mortality-OR-AMR: North-2-0 // Coronary artery disease-Hospital mortality-OR-AMR: North-6-10.2 // Cancer-Hospital mortality-OR-AMR: North-5-0 // Heart failure-Hospital mortality-OR-AMR: North-8-31.6 // Asthma-Hospital mortality-OR-AMR: North-3-0 // Cerebrovascular/Stroke-Hospital mortality-OR-AMR: North-3-0 // V.thromboembolism-Hospital mortality-OR-AMR: North-2-66.3 // Overweight-Hospital mortality-OR-AMR: North-2-0 // Dementia-Hospital mortality-OR-AMR: North-1-0 // Peripheral vascular disease-Hospital mortality-OR-AMR: North-1-0 // Asthma-Hospitalisation-OR-AMR: North-3-66.6 // Cancer-Hospitalisation-OR-AMR: North-5-17.8 // Heart failure-Hospitalisation-OR-AMR: North-4-80.7 // COPD-Hospitalisation-OR-AMR: North-4-15.2 // Diabetes-Hospitalisation-OR-AMR: North-8-37.6 // Hypertension-Hospitalisation-OR-AMR: North-5-59.3 // Obesity/BMI>30-Hospitalisation-OR-AMR: North-7-18 // Overweight-Hospitalisation-OR-AMR: North-3-0 // Chronic kidney disease-Hospitalisation-OR-AMR: North-5-81.5 // Coronary artery disease-Hospitalisation-OR-AMR: North-2-0 // Arrhythmia-Hospitalisation-OR-AMR: North-1-0 // Cerebrovascular/Stroke-Hospitalisation-OR-AMR: North-1-0 // Dementia-Hospitalisation-OR-AMR: North-1-0 // Peripheral vascular disease-Hospitalisation-OR-AMR: North-1-0 // V.thromboembolism-Hospitalisation-OR-AMR: North-1-0 // Coronary artery disease-Intubation-OR-AMR: North-3-18.1 // Chronic kidney disease-Intubation-OR-AMR: North-5-72.5 // Diabetes-Intubation-OR-AMR: North-11-0 // Hypertension-Intubation-OR-AMR: North-9-0 // Obesity/BMI>30-Intubation-OR-AMR: North-10-0 // Heart failure-Intubation-OR-AMR: North-5-0 // Overweight-Intubation-OR-AMR: North-3-21.9 // Cancer-Intubation-OR-AMR: North-2-86.9 // Arrhythmia-Intubation-OR-AMR: North-1-0 // Asthma-Intubation-OR-AMR: North-1-0 // COPD-Intubation-OR-AMR: North-1-0 // Cerebrovascular/Stroke-Intubation-OR-AMR: North-1-0 // Dementia-Intubation-OR-AMR: North-1-0 // Peripheral vascular disease-Intubation-OR-AMR: North-1-0 // V.thromboembolism-Intubation-OR-AMR: North-1-0
Wang, A-L; USA[225] pre-print	cohort; multi-center, 8 + hospitals and more than 400 ambulant; nr - 15. Apr; health services members diagnosed with COVID-19	7592; 49%<60; 55%	PCR / no	case mortality	Log / age, nr of comorbidities (0/1-3/4+: HTN, CKD, COPD, AST, obesity, DM, HIV/AIDS, CANC), comorbidities (AST, CKD), drugs administered at provider, vital signs, smoking status, residential area / age <40 /sex na	JBI=64%	2 estimates<br>Chronic kidney disease-Case mortality-OR-AMR: North-3-76.9 // Asthma-Case mortality-OR-AMR: North-1-0

Study	Study design; setting, number of hospitals; period; Study population	Sample size; age (median (IQR), mean (SD or CI) or % in certain age group(s)); % of males	Given definition of COVID-19/ pre-existing condition (if yes, see section 2.3 for detail)	Outcomes relevant for review	Adjustment method (Regression Model) / Covariates / Reference age and sex category	Risk of bias	Analyses the study participated in [Main meta-analyses reported as: number of the resulted effect estimates the study contributed to; a meta-analysis is described as Condition-Outcome-Measure of association-Region-Total number of studies in the analysis-I²%]
Yehia; USA[226]	cohort; multi-center, 92; 19. Feb - 31. May; follow-up until 25. Jun; individuals hospitalised with COVID-19 diagnosis	11210; median 61 (46-74), nr (nr) ; 50%	PCR / yes, comorbidites were used according to Elixhauser Index	in-hospital mortality	Cox / age, sex, comorbidities (Elixhauser Comorbidity Score ECI, AST, CANC, CKD, CLD, COPD, CHF, CAD, DM, HTN, obesity, Organ, organ transplant history), other: race, Insurance, neighbourhood deprivation index NDI / age 18-49 / female	JBI=82%	8 estimates Coronary artery disease-Hospital mortality-HR-AMR: North-3-0 // Heart failure-Hospital mortality-HR-AMR: North-3-87.2 // Chronic kidney disease-Hospital mortality-HR-AMR: North-4-71.9 // Diabetes-Hospital mortality-HR-AMR: North-5-0 // Obesity/BMI>30-Hospital mortality-HR-AMR: North-4-0 // Cancer-Hospital mortality-HR-AMR: North-4-0 // Asthma-Hospital mortality-HR-AMR: North-2-79.1 // COPD-Hospital mortality-HR-AMR: North-3-0
Zimmerman; USA[227] pre-print	cohort; multi-center, 7; 1. Mar -17. Jul; individuals hospitalised with COVID-19 diagnosis and on invasive mechanical ventilation	304; nr (nr), mean 63 (13); 63%	PCR / yes, for overweight and obesity	in-hospital mortality of patients who required mechanical ventilation (Intubation mortality)	Log/ age, sex, race, ethnicity, BMI categories / age 61-70 / male	JBI=82%	Analysis of evidence for specific population groups – patients on invasive mechanical ventilation
**Regions of America: South/Latin**							
Baqui; Brazil[228]	case series; multi-center; 27. Feb - 4. May; individuals hospitalised with COVID-19 diagnosis	11321; North Brazil - survivors: nr (nr), mean 47 (19), non-survivors: nr (nr), mean 65 (16); Central-south Brazil: survivors: nr (nr), mean 52 (17), non survivors: nr (nr), mean 67 (16); North: survivors: 33%, non-survivors: 67%; Central-south: survivors: 58%, non survivors: 42%	PCR / no	in-hospital mortality	Cox / age, sex, ethnic group, comorbidities (CVD, AST, DM, Resp, obesity, immun, renal disease, liver disease, neurological disease) / age <40 /sex na	other = low-moderate	9 estimates Neurological disease-Hospital mortality-HR-AMR: South/Latin-1-0 // Obesity/BMI>30-Hospital mortality-HR-AMR: South/Latin-2-79.5 // Respiratory disease-Hospital mortality-HR-AMR: South/Latin-1-0 // Chronic kidney disease-Hospital mortality-HR-AMR: South/Latin-2-74.6 // Diabetes-Hospital mortality-HR-AMR: South/Latin-2-73.7 // Immunosuppression-Hospital mortality-HR-AMR: South/Latin-2-0 // Chronic liver disease-Hospital mortality-HR-AMR: South/Latin-1-0 // Cardiovascular disease-Hospital mortality-HR-AMR: South/Latin-1-0 // Asthma-Hospital mortality-HR-AMR: South/Latin-2-0
Bello-Chavolla; Mexico[229]	cohort; electronic database; nr; individuals with COVID-19 diagnosis as reported by Mexican Ministry of Health	51633; nr (nr), mean 47 (16); 58%	PCR / no	case mortality	Cox, Log / age, comorbidities (DM, obesity, pneumonia, CKD, COPD, Immun) / age >=65, <40 / sex na	NOS =6	6 estimates Diabetes-Case mortality-HR-AMR: South/Latin-3-52.6 // Obesity/BMI>30-Case mortality-HR-AMR: South/Latin-3-77 // Respiratory disease-Case mortality-HR-AMR: South/Latin-2-80 // Chronic kidney disease-Case mortality-HR-AMR: South/Latin-3-88.9 // COPD-Case mortality-HR-AMR: South/Latin-2-39.8 // Immunosuppression-Case mortality-HR-AMR: South/Latin-2-43.4 and Analysis of age-stratified estimates

Study	Study design; setting, number of hospitals; period; Study population	Sample size; age (median (IQR), mean (SD or CI) or % in certain age group(s)); % of males	Given definition of COVID-19/ pre-existing condition (if yes, see section 2.3 for detail)	Outcomes relevant for review	Adjustment method (Regression Model) / Covariates / Reference age and sex category	Risk of bias	Analyses the study participated in [Main meta-analyses reported as: number of the resulted effect estimates the study contributed to; a meta-analysis is described as Condition-Outcome-Measure of association-Region-Total number of studies in the analysis-I^2%]
Carrillo-Vega; Mexico[230]	cohort; multi-center; nr - 23. April; diagnosed or suspected COVID-19 cases	10544; nr (nr), mean 46 (16); 58%	no information or unclear / no	hospitalisation, case mortality	Log / model hospitalisation: age, sex, comorbidities (CKD, COPD, HTN, obesity, DM, DM_HTN_obesity, DM_HTN, DM_obesity, HTN_obesity), complication (pneumonia), private services; model risk of death (case mortality): sex, age, CKD, COPD, HTN obesity, DM, Immuno-Suppressed, complication (pneumonia), hospitalisation, intubation, ICU, private services / age 25-49 / female	NOS =6	11 estimates Chronic kidney disease-Hospitalisation-OR-AMR: South/Latin-3-0 // COPD-Hospitalisation-OR-AMR: South/Latin-2-0 // Hypertension-Hospitalisation-OR-AMR: South/Latin-2-73 // Obesity/BMI>30-Hospitalisation-OR-AMR: South/Latin-3-56.2 // Diabetes-Hospitalisation-OR-AMR: South/Latin-3-88.8 // Chronic kidney disease-Case mortality-OR-AMR: South/Latin-1-0 // COPD-Case mortality-OR-AMR: South/Latin-1-0 // Hypertension-Case mortality-OR-AMR: South/Latin-2-70.7 // Obesity/BMI>30-Case mortality-OR-AMR: South/Latin-1-0 // Diabetes-Case mortality-OR-AMR: South/Latin-1-0 // Immunosuppression-Case mortality-OR-AMR: South/Latin-1-0
Denova-Gutiérrez; Mexico[231]	cohort; multi-center; 27. Feb - 10. Apr; individuals hospitalised with COVID-19 diagnosis	3,844; nr (nr), mean 45 (16); 58%	PCR / no	ICU admission (=severe COVID-19)	Log / age, sex, smoking status, obesity, history of chronic diseases (HTN, DM, obesity, CVD, CKD, immunosuppression), place of care, selected health unit that monitors respiratory disease and drug treatment (USMER) / age /different OR for each sex (men and women) are in subgroups	NOS =8	3 estimates Obesity/BMI>30-ICU admission-OR-AMR: South/Latin-1-0 // Diabetes-ICU admission-OR-AMR: South/Latin-1-0 // Hypertension-ICU admission-OR-AMR: South/Latin-1-0
Escalera-Antezana; Bolivia[232]	cohort; multi-center; 2. Mar - 29. Mar; first individuals in Bolivia registered by Ministry of Health diagnosed with COVID-19	107; median 44 (18), nr (nr); 51%	PCR / no	case mortality	Log / age, comorbidities (HTN) / age<60 / male	NOS =6	1 estimate Hypertension-Case mortality-OR-AMR: South/Latin-2-70.7
Giannouchou; Mexico[233]	case series; multi-center; nr - 31. May; suspected COVID-19 cases in a publicly available individual level dataset released by the Mexican Health Ministry	236?439 / 89?756 COVID -19 positive; nr (nr), mean 46 (16); 51% all, 56% COVID-19 positive	PCR / no	hospitalisation	Log / model 1: Age, sex, Mexican nationality, smoking, comorbidities (obesity, DM, HTN, Immun, CVD, AST, COPD, chronic renal disease); Model 2: age, sex, Mexican nationality, smoking, overall number of comorbidities / age 18 - 44 / male	NOS =8	8 estimates Chronic kidney disease-Hospitalisation-OR-AMR: South/Latin-3-0 // COPD-Hospitalisation-OR-AMR: South/Latin-2-0 // Hypertension-Hospitalisation-OR-AMR: South/Latin-2-73 // Obesity/BMI>30-Hospitalisation-OR-AMR: South/Latin-3-56.2 // Diabetes-Hospitalisation-OR-AMR: South/Latin-3-88.8 // Immunosuppression-Hospitalisation-OR-AMR: South/Latin-1-0 // Cardiovascular disease-Hospitalisation-OR-AMR: South/Latin-2-64.7 // Asthma-Hospitalisation-OR-AMR: South/Latin-1-0
Murillo-Zamora; Mexico[234]	cohort; electronic database; 4. Mar - 15. Aug; individuals hospitalised with COVID-19 diagnosis	5393; nr (nr), nr (nr); 61%	PCR / no	in-hospital mortality	Cox / sex, age (30-44,45-59,60+), comorbidities (obesity, AST, COPD, DM, HTN, immunosuppression, CKD), smoking/ age 20-29/female	NOS =9	7 estimates Obesity/BMI>30-Hospital mortality-HR-AMR: South/Latin-2-79.5 // Chronic kidney disease-Hospital mortality-HR-AMR: South/Latin-2-74.6 // Diabetes-Hospital mortality-HR-AMR: South/Latin-2-73.7 // Immunosuppression-Hospital mortality-HR-AMR: South/Latin-2-0 // Asthma-Hospital mortality-HR-AMR: South/Latin-2-0 // COPD-Hospital mortality-HR-AMR: South/Latin-1-0 // Hypertension-Hospital mortality-HR-AMR: South/Latin-1-0
Soares; Brazil[235]	cohort; electronic database; 29. Feb - 11. Jun; individuals from Espírito Santo State, Brazil, in registry diagnosed with COVID-19	10713; nr; 45%	PCR / no	hospitalisation, in-hospital mortality	Log / model 1: age, sex, race, comorbidities (CVD, DM, kidney diseases, obesity, Resp), smoking, symptoms; model 2: age, comorbidities (CVD, kidney diseases), symptoms / model 1: age <60/female; model 2: age<60/ sex na	NOS =8	7 estimates Chronic kidney disease-Hospitalisation-OR-AMR: South/Latin-3-0 // Obesity/BMI>30-Hospitalisation-OR-AMR: South/Latin-3-56.2 // Diabetes-Hospitalisation-OR-AMR: South/Latin-3-88.8 // Cardiovascular disease-Hospitalisation-OR-AMR: South/Latin-2-64.7 // Respiratory disease-Hospitalisation-OR-AMR: South/Latin-1-0 // Cardiovascular disease-Hospital mortality-OR-AMR: South/Latin-1-0 // Chronic kidney disease-Hospital mortality-OR-AMR: South/Latin-1-0
Solís; Mexico[236]	cohort; electronic database; nr - 18. Apr; individuals hospitalised with COVID-19 diagnosis	7497; median 46 (nr), nr (nr); 58%	PCR / no	in-hospital mortality	Cox / sex, age, comorbidities (HTN, obesity, CVD, Immun, COPD, DM, AST, CKD), smoking, institution / age 45-49/sex na	NOS =5	8 estimates Diabetes-Case mortality-HR-AMR: South/Latin-3-52.6 // Obesity/BMI>30-Case mortality-HR-AMR: South/Latin-3-77 // Chronic kidney disease-Case mortality-HR-AMR: South/Latin-3-88.9 // COPD-Case mortality-HR-AMR: South/Latin-2-39.8 // Immunosuppression-Case mortality-HR-AMR: South/Latin-2-43.4 // Hypertension-Case mortality-HR-AMR: South/Latin-1-0 // Cardiovascular disease-Case mortality-HR-AMR: South/Latin-2-98.2 // Asthma-Case mortality-HR-AMR: South/Latin-1-0

Study	Study design; setting, number of hospitals; period; Study population	Sample size; age (median (IQR), mean (SD or CI) or % in certain age group(s)); % of males	Given definition of COVID-19/ pre-existing condition (if yes, see section 2.3 for detail)	Outcomes relevant for review	Adjustment method (Regression Model) / Covariates / Reference age and sex category	Risk of bias	Analyses the study participated in [Main meta-analyses reported as: number of the resulted effect estimates the study contributed to; a meta-analysis is described as Condition-Outcome-Measure of association-Region-Total number of studies in the analysis-I^2%]
Sousa; Brazil[237]	cohort; electronic database; nr - 14. Apr; individuals with diagnosed COVID-19 identified from registry	2070; median 44 (34-59; 76%), nr (nr); ≥ 60 (24%); 49%	PCR / no	case mortality	Cox / age (elderly), comorbidities (CVD, DM, hematologic disease, neurologic disease, obesity, pneumopathies, renal disease) / na	NOS =8	6 estimates Diabetes-Case mortality-HR-AMR: South/Latin-3-52.6 // Obesity/BMI>30-Case mortality-HR-AMR: South/Latin-3-77 // Respiratory disease-Case mortality-HR-AMR: South/Latin-2-80 // Chronic kidney disease-Case mortality-HR-AMR: South/Latin-3-88.9 // Cardiovascular disease-Case mortality-HR-AMR: South/Latin-2-98.2 // Neurological disease-Case mortality-HR-AMR: South/Latin-1-0
**Western Pacific Region**							
Bi; China[238] pre-print	cohort; single-center; 11. Jan - 10. Mar; patients with diagnosed COVID-19 hospitalised in Shenzen	420; nr (nr), mean 45 (35); 48%	PCR / no	ICU admission	competing risk model / model 1: age, sex, any comorbidity; model 2: age, sex, comorbidities (HTN, DM, cardiac diseases, Resp, pulmonary disease or respiratory disease not defined, CRB, number of comorbidities) / age 0-59 / female; model 2: age 0-39 / female	other = low	5 estimates Dementia-Hospital mortality-OR-EUR-4-49.7 // Hypertension-ICU admission-HR-WPR-1-0 // Diabetes-ICU admission-HR-WPR-1-0 // Respiratory disease-ICU admission-HR-WPR-1-0 // Cerebrovascular/Stroke-ICU admission-HR-WPR-1-0
Chen.F; China[239]	cohort; single-center; 1. Jan - 15. Feb; individuals hospitalised with COVID-19 diagnosis	660; median 55 (34?68), nr (nr); 45%	no information or unclear / no	in-hospital mortality	Log / age, comorbidities (HTN, CRB), SOFA, laboratory values / age>60 vs. <=60 / sex na	NOS =7	2 estimates Hypertension-Hospital mortality-OR-WPR-9-0 // Cerebrovascular/Stroke-Hospital mortality-OR-WPR-3-61.1
Chen.J; China[240]	cohort; single-center; 18. Jan - 27. Mar; individuals hospitalised with COVID-19 diagnosis	3309; median 62 (49-69), nr (nr); 50%	PCR , clinical diagnosis (% unknown) / types of cancer	in-hospital mortality	Log / age, sex, comorbidities (HTN, CVD, CRB, CANC, CKD, COPD), days from onset to clinical presentation / to admission / Age<=45 / male	NOS =7	6 estimates Hypertension-Hospital mortality-OR-WPR-9-0 // Cerebrovascular/Stroke-Hospital mortality-OR-WPR-3-61.1 // Cardiovascular disease-Hospital mortality-OR-WPR-2-0 // Cancer-Hospital mortality-OR-WPR-4-0 // Chronic kidney disease-Hospital mortality-OR-WPR-2-0 // COPD-Hospital mortality-OR-WPR-4-0
Chen.R; China[241]	cohort; multi-center, 575; nr - 31. Jan; individuals hospitalised with COVID-19 diagnosis	1590; fatal cases: median 69 (51-86), nr (nr); non-fatal cases: median 48 (1-94), nr (nr); fatal cases: 78%, non-fatal cases: 57%	PCR / no	in-hospital mortality	Cox / age, laboratory values, complications, comorbidities (CHD, CVD) / age 65-74 vs <65 and >=75 vs <65 / sex na	NOS =7	2 estimates Coronary artery disease-Hospital mortality-HR-WPR-3-63.7 // Cardiovascular disease-Hospital mortality-HR-WPR-7-0
Dai; China[242]	cohort; multi-center, 14; 1. Jan - 24. Feb; individuals hospitalised with COVID-19 diagnosis	641 (105 cancer patients matched to 536 non-cancer); cancer patients: median 64 (14), nr (nr); non-cancer: median 63 (14), nr (nr); 55% cancer group, 46% non-cancer group	PCR / cancer types	in-hospital mortality, ICU admission, intubation	Log / age, sex, comorbidities (CANC, DM, HTN, COPD), smoking / na	NOS =9	11 estimates Hypertension-Hospital mortality-OR-WPR-9-0 // Cancer-Hospital mortality-OR-WPR-4-0 // COPD-Hospital mortality-OR-WPR-4-0 // Diabetes-Hospital mortality-OR-WPR-4-84.8 // Cancer-ICU admission-OR-WPR-1-0 // Diabetes-ICU admission-OR-WPR-1-0 // Hypertension-ICU admission-OR-WPR-1-0 // COPD-ICU admission-OR-WPR-1-0 // Cancer-Intubation-OR-WPR-1-0 // Diabetes-Intubation-OR-WPR-1-0 // Hypertension-Intubation-OR-WPR-1-0
Du, R; China[243] pre-print	case series; single-center; 25. Dec - 7. Feb; individuals hospitalised with COVID-19 diagnosis	179; median 57 (49-68), nr (nr); 54%	PCR / no	ICU admission	Log / age, number of comorbidities, laboratory values, detection of SARS-CoV-2 / age< 60 / nr	NOS = high	Risk “number of comorbidities” was excluded from meta-analyses
Du, RH; China[244]	cohort; single-center; 25. Dec - 7. Feb; individuals hospitalised with COVID-19 diagnosis	179 (136 COVID-19 positive); nr (nr), mean 58 (14); 54%	PCR / no	in-hospital mortality	Log / age, comorbidities (CVD or CBR), laboratory values / Age >=65 years /sex nr	NOS =8	Risk “CVD or CBR” due to very composite definition of pre-existing condition. i.e. either cardio-vascular od cerebra-vascular disease

Study	Study design; setting, number of hospitals; period; Study population	Sample size; age (median (IQR), mean (SD or CI) or % in certain age group(s)); % of males	Given definition of COVID-19/ pre-existing condition (if yes, see section 2.3 for detail)	Outcomes relevant for review	Adjustment method (Regression Model) / Covariates / Reference age and sex category	Risk of bias	Analyses the study participated in [Main meta-analyses reported as: number of the resulted effect estimates the study contributed to; a meta-analysis is described as Condition-Outcome-Measure of association-Region-Total number of studies in the analysis-I^2%]
Feng; China[245]	cohort; multi-center, 3; 1. Jan - 15. Feb; individuals hospitalised with COVID-19 diagnosis	476 / 337 included in adjusted analysis; median 53 (40-64), nr (nr); 57%	PCR / no	in-hospital mortality	Cox / age, laboratory values, comorbidities (HTN, CVD, DM) / age< 75 / sex na	NOS = high	3 estimates<br>Cardiovascular disease-Hospital mortality-HR-WPR-7-0 // Hypertension-Hospital mortality-HR-WPR-8-20.3 // Diabetes-Hospital mortality-HR-WPR-10-17.5
Gao; China[246]	cohort; single-center; 5. Feb - 15. Mar; individuals hospitalised with COVID-19 diagnosis	2877 / 850 patients with history of hypertension; without HTN: nr (nr), mean 55 (15); with HTN: nr (nr), mean 64 (11); 51% group without HTN, 52% group with HTN	PCR / yes, partially, for HTN only	in-hospital mortality	Cox / age, comorbidities (fatigue, HTN, Myo, Renal failure, chronic heart failure), laboratory values / age per year / sex na	NOS >=5- <=8	4 estimates<br>Hypertension-Hospital mortality-HR-WPR-8-20.3 // Infarction-Hospital mortality-HR-WPR-1-0 // Chronic kidney disease-Hospital mortality-HR-WPR-3-92.4 // Heart failure-Hospital mortality-HR-WPR-1-0
Gu, T, Chu; China[247]	case-control; multi-center; 18. Dec - 8. Mar; individuals diagnosed with COVID-19 identified from the news or the national/provincial/municipal health commissions of China	275 (cases: 94 controls: 181; median 68 (22), nr (nr); cases: median 72 (16), nr (nr); controls: median 67 (22), nr (nr); 63%; cases: 60%, controls: 65%	PCR / no	case mortality	Cox / model 1: age, sex, time, comorbidities (cardiac diseases, CRB, COPD, renal failure); model 2: age, sex, time, comorbidity score / na	NOS =7	4 estimates<br>Coronary artery disease-Case mortality-HR-WPR-2-87.7 // Cerebrovascular/Stroke-Case mortality-HR-WPR-2-31.9 // COPD-Case mortality-HR-WPR-2-0 // Chronic kidney disease-Case mortality-HR-WPR-2-0
Huang; China[248]	cohort; 2 hospitals; after outbreak 2020 in Wuhan (no specific date given); individuals hospitalised with COVID-19 diagnosis	310; median 62 (49-70), nr (nr); 56%	PCR / no	in-hospital mortality	Log / age, sex, comorbidities (HTN) / age numeric / male	MINORS =10	1 estimate<br>Hypertension-Hospital mortality-OR-WPR-9-0
Hwang; Korea[249]	cohort; 2 hospitals; 1. Feb - 25. Mar; individuals hospitalised with COVID-19 diagnosis	103; nr (nr), mean 68 (15); 50%	PCR / yes, partially, chronic lung disease, dementia= Alzheimers	in-hospital mortality	Cox / age, comorbidities (DM, chronic lung disease, CVD, DEM (Alzheimer), stroke) / na	NOS =8	5 estimates<br>Cardiovascular disease-Hospital mortality-HR-WPR-7-0 // Diabetes-Hospital mortality-HR-WPR-10-17.5 // Respiratory disease-Hospital mortality-HR-WPR-2-61.2 // Dementia-Hospital mortality-HR-WPR-1-0 // Cerebrovascular/Stroke-Hospital mortality-HR-WPR-4-0
Kim.M; Korea[250]	cohort; multi-center, 5; 18. Feb - 31. Mar; individuals hospitalised with COVID-19 diagnosis	470, non-DM und DM each 235; non-DM: nr (nr), mean 56 (18); DM: nr (nr), mean 68 (12); non-DM: 40%, DM: 45%	PCR / yes, for DM	in-hospital mortality	Cox, Matching / age, sex, comorbidities (DM, HTN, CRD, CVD, CKD, Resp, CANC) / na	NOS =8	1 estimate<br>Diabetes-Hospital mortality-HR-WPR-10-17.5
Lee, HY; Korea[251]	cohort; electronic database; 19. Jan - 16. Mar; individuals diagnosed with COVID-19	8266; nr (nr), mean 44 (19); 39%	PCR / no	case mortality	Cox / age (per year increase), age, sex, comorbidities (HTN, DM, CAD, stroke, COPD, CANC, CKD), medications / age numeric/male	other =high	7 estimates<br>Coronary artery disease-Case mortality-HR-WPR-2-87.7 // Cerebrovascular/Stroke-Case mortality-HR-WPR-2-31.9 // COPD-Case mortality-HR-WPR-2-0 // Chronic kidney disease-Case mortality-HR-WPR-2-0 // Hypertension-Case mortality-HR-WPR-1-0 // Diabetes-Case mortality-HR-WPR-1-0 // Cancer-Case mortality-HR-WPR-1-0

Study	Study design; setting, number of hospitals; period; Study population	Sample size; age (median (IQR), mean (SD or CI) or % in certain age group(s)); % of males	Given definition of COVID-19/ pre-existing condition (if yes, see section 2.3 for detail)	Outcomes relevant for review	Adjustment method (Regression Model) / Covariates / Reference age and sex category	Risk of bias	Analyses the study participated in [Main meta-analyses reported as: number of the resulted effect estimates the study contributed to; a meta-analysis is described as Condition-Outcome-Measure of association-Region-Total number of studies in the analysis-I²%]
Li.J; China[252]	cohort; single-center; 23. Jan - 14. Mar; individuals hospitalised with COVID-19 diagnosis	596, with CVD: 215, without CVD: 384; nr (nr), mean 58 (47-68); 47%	PCR / no	in-hospital mortality	Cox / sex, age, comorbidities (DM, malignancy, HTN, coronary heart disease, ART, CVD) / age<65 / sex na	NOS =8	6 estimates<br>Coronary artery disease-Hospital mortality-HR-WPR-3-63.7 // Hypertension-Hospital mortality-HR-WPR-8-20.3 // Diabetes-Hospital mortality-HR-WPR-10-17.5 // Cerebrovascular/Stroke-Hospital mortality-HR-WPR-4-0 // Cancer-Hospital mortality-HR-WPR-4-8.9 // Arrhythmia-Hospital mortality-HR-WPR-1-0
Li.Q; China[253]	cohort; multi-center, 5; 20. Jan - 4. Apr; individuals hospitalised with COVID-19 diagnosis	1859, cancer: 65; non-cancer: 1794; median 59 (45-68), nr (nr); 50%	PCR and/or blood test for SARS-Cov-2 IgG/IgM antibodies /yes	in-hospital mortality	Cox / model 1: age (per year increase), sex, comorbidities (CANC), COVID-19 severity, smoking, laboratory values; model 2: age (<65), sex, comorbidities (CANC), COVID-19 severity, smoking, laboratory values; model 3: age (>=65), sex, comorbidities (CANC), COVID-19 severity, smoking, laboratory values / age numeric/female	NOS =6	1 estimate<br>Cancer-Hospital mortality-HR-WPR-4-8.9<br>and<br>Analysis of age-stratified estimates
Meng; China[254]	cohort; single-center; 18. Jan - 27. Mar; individuals hospitalised with COVID-19 diagnosis	2665; cancer: median 62 (16), nr (nr); non-cancer: median 58 (16), nr (nr); cancer: 56%; non-cancer: 50%	PCR / no	in-hospital mortality	Log / sex, age, (4 groups), comorbidities (HTN, coronary heart disease, DM, COPD, CKD, CRB, hepatitits, tuberculosis, tumor) / age <=49/male	NOS =7	9 estimates<br>Hypertension-Hospital mortality-OR-WPR-9-0 // Cerebrovascular/Stroke-Hospital mortality-OR-WPR-3-61.1 // Cancer-Hospital mortality-OR-WPR-4-0 // Chronic kidney disease-Hospital mortality-OR-WPR-2-0 // COPD-Hospital mortality-OR-WPR-4-0 // Diabetes-Hospital mortality-OR-WPR-4-84.8 // Coronary artery disease-Hospital mortality-OR-WPR-2-0 // Hepatitis-Hospital mortality-OR-WPR-1-0 // Tuberculosis-Hospital mortality-OR-WPR-1-0
Moon; Korea[255]	cohort; 2 hospitals; 18. Feb - 30. Jun; individuals hospitalised with COVID-19 diagnosis	352; median 57 (38-72), nr (nr); 41%	PCR / no	in-hospital mortality	Log / age, comorbidities (malignancy, DM), body temperature / age < 70 /sex na	NOS =7	2 estimates<br>Cancer-Hospital mortality-OR-WPR-4-0 // Diabetes-Hospital mortality-OR-WPR-4-84.8
Shang; China[256]	cohort; multi-center, 3; 1. Jan - 31. Mar; individuals hospitalised with COVID-19 diagnosis (severe COVID-19)	2529; median 66 (57-73), nr (nr); 65%	PCR or viral sequencing / no	in-hospital mortality	Log / age, comorbidities (cardiac diseases), laboratory values / age<60 / sex na	other =low	1 estimate<br>Heart disease-Hospital mortality-OR-WPR-2-0
Shi, Q; China[257]	cohort; 2 hospitals; 1. Jan - 8. Mar; individuals hospitalised with COVID-19 diagnosis	306; median 64 (56-72), nr (nr); 49%	PCR / yes, partially, information on patients with DM provided	in-hospital mortality	Cox / age, sex, comorbidities (HTN, CVD, Resp), laboratory values / age>=70 /na	NOS =9	4 estimates<br>Cardiovascular disease-Hospital mortality-HR-WPR-7-0 // Hypertension-Hospital mortality-HR-WPR-8-20.3 // Diabetes-Hospital mortality-HR-WPR-10-17.5 // Respiratory disease-Hospital mortality-HR-WPR-2-61.2
Shi, S; China[258]	cohort; single-center; 20. Jan - 10. Feb; individuals hospitalised with COVID-19 diagnosis	416; median 64 (21-95), nr (nr); 49%	PCR / no	in-hospital mortality	Cox /age, comorbidities (CVD, CRB, DM, COPD, CANC, Resp), laboratory values / na	NOS =6	6 estimates<br>Cardiovascular disease-Hospital mortality-HR-WPR-7-0 // Diabetes-Hospital mortality-HR-WPR-10-17.5 // Chronic kidney disease-Hospital mortality-HR-WPR-3-92.4 // Cerebrovascular/Stroke-Hospital mortality-HR-WPR-4-0 // Cancer-Hospital mortality-HR-WPR-4-8.9 // COPD-Hospital mortality-HR-WPR-3-0
Sun; China[259]	case-control; single-center; 29. Jan - 5. Mar; individuals hospitalised with COVID-19 diagnosis	244; Discharged: median 67 (64-72), nr (nr); Deceased: median 72 (66-78), nr (nr); 54%	no information or unclear / no	in-hospital mortality	Log / age, sex, comorbidities (HTN, Resp), laboratory values / age<60 / male	NOS =7	2 estimates<br>Hypertension-Hospital mortality-OR-WPR-9-0 // Respiratory disease-Hospital mortality-OR-WPR-1-0
Sy; Philippines[260]	cohort; electronic database; 17. May - 15. Jun; individuals hospitalised with COVID-19 diagnosis	530; nr (nr), mean 49 (19); 0,7	PCR / no	in-hospital mortality	Log, Matching / age, sex, and other comorbid conditions (COPD, AST, DM, HTN, CANC, renal disease, cardiac disease, and autoimmune disorders), tuberculosis variable of interest in model / na	NOS =8	3 estimates<br>Tuberculosis-Hospitalisation-RR-WPR-1-0 // Tuberculosis-Case mortality-RR-WPR-1-0 // Tuberculosis-Hospital mortality-RR-WPR-1-0

Study	Study design; setting, number of hospitals; period; Study population	Sample size; age (median (IQR), mean (SD or CI) or % in certain age group(s)); % of males	Given definition of COVID-19/ pre-existing condition (if yes, see section 2.3 for detail)	Outcomes relevant for review	Adjustment method (Regression Model) / Covariates / Reference age and sex category	Risk of bias	Analyses the study participated in [Main meta-analyses reported as: number of the resulted effect estimates the study contributed to; a meta-analysis is described as Condition-Outcome-Measure of association-Region-Total number of studies in the analysis-I^2%]
Tai; China[261]	cohort; electronic database; 5. Feb - 10. Mar; individuals hospitalised with COVID-19 diagnosis	332; median 51 (40-59), nr (nr); 39%	PCR / yes, partially on CVD	case ICU	Log / age, sex, comorbidities (CVD, DM, lung diseases), chest tightness / na	NOS =8	The outcome was excluded from the reporting
Wang, D; China[262]	case series; 2 hospitals; nr - 10. Feb; individuals hospitalised with COVID-19 diagnosis	107; median 51 (36-65), nr (nr); 53%	PCR / no	in-hospital mortality	Log / age, sex, comorbidities (HTN, CVD), laboratory values / na	ROBINSI=6	2 estimates Hypertension-Hospital mortality-OR-WPR-9-0 // Cardiovascular disease-Hospital mortality-OR-WPR-2-0
Wang, K; China[263]	cohort; single-center; 7. Jan - 11. Feb; individuals hospitalised with COVID-19 diagnosis	296; nr (nr), mean 55 (17); 54%	PCR / no	in-hospital mortality	Log / clinical model: age, comorbidities (HTN, CHD), laboratory values / na	NOS =7	2 estimates Hypertension-Hospital mortality-OR-WPR-9-0 // Heart disease-Hospital mortality-OR-WPR-2-0
Wang, L; China[264]	cohort; single-center; 1. Jan - 6. Feb; individuals hospitalised with COVID-19 diagnosis	339; median 69 (75-76), nr (nr); 49%	PCR / no	in-hospital mortality	Cox / model 1: age, comorbidities (CVD, CRB, COPD); model 2: age, complications / na	NOS =8	3 estimates Cardiovascular disease-Hospital mortality-HR-WPR-7-0 // Cerebrovascular/Stroke-Hospital mortality-HR-WPR-4-0 // COPD-Hospital mortality-HR-WPR-3-0
Xie; China[265]	cohort; single-center; 28. Jan - 28. Feb; individuals hospitalised with COVID-19 diagnosis (moderate to critical COVID-19)	140; median 60 (47-68), nr (nr); 51%	PCR / no	in-hospital mortality	Cox / age, sex, comorbidities (HTN, any comorbidity), laboratory values, complications / age<60/female	other =low-moderate	1 estimate Hypertension-Hospital mortality-HR-WPR-8-20.3
Xu, J; China[266]	cohort; multi-center, 3; 12. Jan - 3. Feb; ICU care patients with COVID-19 diagnosis	239; nr (nr), mean 62 (13); 60%	PCR / no	ICU mortality	Cox / age, comorbidities (malignancy, liver dysfunction), laboratory values, complications / na	NOS =7	The outcome was excluded from the reporting
Yan, Y; China[267]	cohort; single-center; 10. Jan - 24. Feb; individuals hospitalised with COVID-19 diagnosis	193; median 64 (49-73), nr (nr); 59%	PCR / yes, some information on DM	in-hospital mortality	Cox / model 1: age, sex, comorbidities (DM); model 2: age, sex, comorbidities (DM, CVD, CRB) / na	NOS =8	2 estimates Hypertension-Hospital mortality-HR-WPR-8-20.3 // Diabetes-Hospital mortality-HR-WPR-10-17.5
Yang, Q; China[268]	cohort; single-center; 1. Jan - 29. Feb; individuals hospitalised with COVID-19 diagnosis	226; nr (nr), mean 50 (15); nr (nr), mean 68 (16); 47%; 62%	PCR / no	in-hospital mortality	Cox, Matching / model 1 (with matching design): sex, age, comorbidities (HTN), laboratory values; model 2 (with matching design) for critically ill patients: sex, age, comorbidities (HTN), laboratory values, Acute Physiology And Chronic Health Evaluation (APACHE) II score, duration of methylprednisolone use / na	other =low	1 estimate<br><br>Hypertension-Hospital mortality-HR-WPR-8-20.3
Yu; China[269]	cohort; single-center; 14. Jan - 28. Feb; individuals hospitalised with COVID-19 diagnosis	1464; median 64 (51-71), nr (nr); 50%	PCR / no	in-hospital mortality	Log / model 1: age, sex, comorbidities (HTN, DM), laboratory values: model 2: age, sex, comorbidities (DM), laboratory values / age<65/female	NOS =7	2 estimates Hypertension-Hospital mortality-OR-WPR-9-0 // Diabetes-Hospital mortality-OR-WPR-4-84.8
Zhang, F; China[270]	case-control; 2 hospitals; 7. Feb - 27. Mar; young individuals hospitalised with COVID-19 diagnosis	53 (cases: 13, controls: 40); nr (nr), nr (nr); nr	PCR / yes, definition of obesity provided	in-hospital mortality	Log / BMI, laboratory values / na	NOS =8	Analysis of age-stratified estimates

Study	Study design; setting, number of hospitals; period; Study population	Sample size; age (median (IQR), mean (SD or CI) or % in certain age group(s)); % of males	Given definition of COVID-19/ pre-existing condition (if yes, see section 2.3 for detail)	Outcomes relevant for review	Adjustment method (Regression Model) / Covariates / Reference age and sex category	Risk of bias	**Analyses the study participated in** [Main meta-analyses reported as: number of the resulted effect estimates the study contributed to; a meta-analysis is described as Condition-Outcome-Measure of association-Region-Total number of studies in the analysis-I^2%]
Zhang, H; China[271]	cohort; multi-center, 5; 5. Jan - 18. Mar; individuals with cancer diagnosed with COVID-19	107; median 66 (37-98), nr (nr); 56%	PCR and/or based on radiologic findings (% unclear) / yes, cancer defined as receiving ongoing active anticancer treatment	case mortality	Cox / age, receiving anticancer treatment / age numeric/sex na	NOS =8	Analysis of evidence for specific population groups – patients with cancer
Zhang, P; China[272]	cohort; multi-center, 9; 31. Dez - 20. Feb; individuals with hypertension hospitalised with COVID-19 diagnosis	1128; median 64 (57-69), nr (nr); 53%	PCR and/or based on radiologic findings (% unclear)/ yes, partially, for HTN	in-hospital mortality	Cox, Matching / model 1: age, sex, comorbidities (coronary heart disease, CRB), in-hospital medication, use of ACEI/ARB; model 2 match: age, sex, in-hospital medication, laboratory values (DM, coronary heart disease, CRB, chronic renal disease), medications / na	NOS =>8	1 estimate<br>Hypertension-Hospital mortality-HR-WPR-8-20.3
Zhang, X; China[273] pre-print	cohort; single-center; 2. Feb - 25. Mar; 23. Feb - 24. Mar; 21. Feb - 25. Mar; 11. Feb - 21. Mar; 8. Feb - 15. Mar; critically ill patients diagnosed with COVID-19 admitted to ICU	282; median 69 (60-78), nr (nr); 55%	PCR / no	hospitalisation, in-hospital mortality	Log / model inhospital mortality: COPD, AKI stage, SOFA score / na	QUIPS=moderate	1 estimate<br>COPD-Hospital mortality-OR-WPR-4-0
Zhang, Y; China[274]	cohort; single-center; 29. Jan - 12. Feb; individuals with COVID-19 diagnosis hospitalised	258; median 64 (23-91), nr (nr); 53%	PCR / no	case mortality	Cox / age, comorbidities (DM, CVD, CKD), laboratory values / na	NOS =8	1 estimate<br>Diabetes-Hospital mortality-HR-WPR-10-17.5
Zhao; China[275]	cohort; single-center; 1. Jan - 14. Feb; individuals hospitalised with COVID-19 diagnosis	1000; median 61 (46-70), nr (nr); 47%	PCR or viral sequencing/ no	in-hospital mortality	Cox / age (3 groups: <60, 60<age<75, <75); comorbidities (HTN, DM, CHD, COPD, CVD, chronic renal disease, chronic liver disease, malignancy) / age<60/na	NOS =7	8 estimates<br>Coronary artery disease-Hospital mortality-HR-WPR-3-63.7 // Cardiovascular disease-Hospital mortality-HR-WPR-7-0 // Hypertension-Hospital mortality-HR-WPR-8-20.3 // Diabetes-Hospital mortality-HR-WPR-10-17.5 // Chronic kidney disease-Hospital mortality-HR-WPR-3-92.4 // Cancer-Hospital mortality-HR-WPR-4-8.9 // COPD-Hospital mortality-HR-WPR-3-0 // Chronic liver disease-Hospital mortality-HR-WPR-1-0
Zhou; China[276]	cohort; 2 hospitals; 29. Dec - 31. Jan; individuals hospitalised with COVID-19 diagnosis	171; median 56 (46-67), nr (nr); 62%	PCR / no	in-hospital mortality	Log / age, comorbidities (CHD), SOFA score, laboratory values / age na/male	NOS =7	1 estimate<br>Coronary artery disease-Hospital mortality-OR-WPR-2-0
Zhu; China[277]	cohort; multi-center, 19; 30. Dez - 20. Mar; individuals hospitalised with COVID-19 diagnosis	7337 (952 diabetic, 6385 non-diabetic); diabetic: median 62 (55-68), nr (nr); non-diabetic: median 53 (40-63); nr (nr); 54% group with DM; 47% without DM	PCR or viral sequencing/ yes, definition of types of DM provided	in-hospital mortality	Cox / age, sex, comorbidities (DM), hospital site; model 2: age, sex, comorbidities (DM), hospital site, severity of COVID-19 / na	NOS =8	1 estimates<br>Diabetes-Hospital mortality-HR-WPR-10-17.5
**International**							

Study	Study design; setting, number of hospitals; period; Study population	Sample size; age (median (IQR), mean (SD or CI) or % in certain age group(s)); % of males	Given definition of COVID-19/ pre-existing condition (if yes, see section 2.3 for detail)	Outcomes relevant for review	Adjustment method (Regression Model) / Covariates / Reference age and sex category	Risk of bias	Analyses the study participated in [Main meta-analyses reported as: number of the resulted effect estimates the study contributed to; a meta-analysis is described as Condition-Outcome-Measure of association-Region-Total number of studies in the analysis-I^2%]
Garassino; Global (Europe, USA, China)[278]	cohort; multi-center, 87; 26. Mar - 12. Apr; patients with thoracic malignancies diagnosed with COVID-19, identified from registry	200; median 68 (62 -75), nr (nr); 70%	PCR (90%) /diagnosis based on radiologic findings (7%) / clinical diagnosis (3%) / no	case mortality	Log / age, sex, comorbidities (HTN, COPD), smoking / age <65 / male	NOS =6	Analysis of evidence for specific population groups – patients with cancer
Kuderer; International[279]	cohort; electronic database; 17. Mar - 16. Apr; individuals with cancer hospitalised with COVID-19 diagnosis	928; median 66 (57-76), nr (nr); 50%	PCR / no	in-hospital mortality	Log / age, sex, race and ethnicity, region of patient residence, smoking, comorbidities (obesity, number of comorbidities 1->=4), type of malignancy, cancer status, Eastern Co-operative Oncology Group (ECOG) performance status, type of anticancer status, recent surgery, treatment of COVID-19 / age per 10 years/female	NOS =8	Analysis of evidence for specific population groups – patients with cancer
Mato; International[280]	cohort; multi-center, 43; 17. Feb - 30. Apr; patients with history of chronic lymphocytic leukemia hospitalised with COVID-19 diagnosis	198; median 63 (35-92), nr (nr); 63%	PCR / no	in-hospital mortality	Cox / age, cumulative illness rating scale (CIRS), comorbidities (DM, AST, Resp) / age >=75/sex na	NOS =6	International studies were excluded from meta-analyses

ACEI, Angiotensin Converting Enzyme Inhibitor; AIDS, acquired immunodeficiency syndrome; ARBs, angiotensin receptor blocker; ART, arrhythmia or atrial fibrillation; AST, asthma; Auto, Autoimmune condition; BMI, body mass index; CAD, coronary artery disease; CANC, cancer -- not specified whether active or not, any cancer; CANCact, active cancer; CCI, Charlson Comorbidity Index; CHF, congestive heart failure;CKD, chronic kidney disease; CLD, chronic liver disease; COPD, chronic obstructive pulmonary disease; Cox, Cox proportional hazards model; CRB, cerebrovascular diseases or stroke; CVA/TIA, cerebrovascular accident/transient ischemic attack; CVD, cardiovascular disease; DEM, dementia; DM, diabetes mellitus; ECI, Elixhauser Comorbidity Index; eGFR, Estimated glomerular filtration rate; GLM, generalized linear model; HIV, human immunodeficiency virus; HTN, hypertension; IBD, inflammatory bowel disease; IHD, Ischemic heart disease; ILD, interstitial lung disease; Immun, immunodeficiency or immunosuppressed state; IQR, interquartile range; JBI, Joanna Briggs Institute Score; Log, logistic regression model; MINORS, methodological index for non-randomized studies tool; Myo, Myocardial infarction; Na, not available; NOS, Newcastle Ottawa Scale; Nr, not reported; Organ, organ transplant history; PVD, peripheral vascular disease; QUIPS, Quality In Prognosis Studies Tool; Resp, Pulmonary Disease or respiratory disease not defined; Rheuma, rheumatological disease; ROBINS, Risk Of Bias in Non-randomized Studies tool; ROBINSI, Risk Of Bias in Non-randomized Studies - of Interventions tool; SES, socioeconomic status; SOFA, sequential organ failure assessment; UC, ulcerative colitis; VTE, venous thromboembolism;

2.2. Summary of the studies based on the general population

Nine[281-289] studies which reported community-based estimates were excluded from the analysis due to incomparability. This section thus serves as a short presentation of the results of these nine studies (see Table 4 table).

Six[281,284-288] studies were conducted using a population from the UK Biobank, two[282,289] studies were based on an English, and one study on a Spanish cohort[283].The observation periods used in the studies ranged from February 01, 2020 to June 22, 2020. Two English studies enrolled general populations identified via "OpenSAFELY England"—a health analytics platform that provides primary data of 40% of the English population (https://opensafely.org/), and the Spanish study included individuals identified via a database containing the data from 60 hospitals in Spain. The sample sizes were large (at least N=269,070 up to over 17 million).

The outcomes used that were of interest to our research question were hospitalisation (in 6/9 studies,) and case mortality (3/9). Other outcomes such as in-hospital mortality or ICU admission were used in 1/9 studies (see Table 4). Results are presented from multivariate models only, at least adjusting for sex and age.

Increasing risks for hospitalisation were seen for the pre-existing conditions dementia[281], type 2 diabetes[281,288], COPD[281,286], pneumonia[281], depression[281], atrial fibrillation[281], hypertension[281,286], chronic kidney disease in women only[281] and in general[288], asthma in women only[281], overweight and obesity[284-286,288]. Lassale et al.[287] found that "ever seen a psychiatrist", chronic bronchitis, CVD and hypertension were not associated with an increased risk of hospitalisation.

In-hospital mortality was used as an outcome in the study of Atkins et al.[281], only. The authors showed that the pre-existing conditions dementia, type 2 diabetes, COPD, pneumonia and depression were associated with an increased risk of in-hospital mortality.

In the study by Bhaskaran et al.[282], case mortality was associated with HIV. Del Amo et al.[283] found that the risk standardised to the age and sex of the general population in Spain aged 20 to 79 years old for death among HIV-positive persons receiving antiretroviral therapy (ART) was 3.7 (CI 3.6-3.8). For comparison, in the Spanish general population aged 20 to 79 years during the same period the risk for death was 2.1 per 10,000. Other pre-existing conditions associated with an increased risk of case mortality were determined by Williamson et al.[289]: obesity with a BMI>40, diabetes, severe asthma (defined as asthma with recent use of an oral corticosteroid), respiratory disease, chronic heart disease, liver disease, stroke, dementia, other neurological diseases, reduced kidney function (greater hazard ratio associated with a lower estimated glomerular filtration rate; eGFR), autoimmune diseases (rheumatoid arthritis, lupus or psoriasis) and other immunosuppressive conditions, a recent (that is, in the last five years) history of haematological malignancy, other cancers, a history of dialysis or end-stage renal failure.

Table 4 Overview of the studies which report community-based estimates (n=9)

Study	Study design	Study population	Sample size, age (mean (SD or CI) or median (IQR)), % of males	Outcomes relevant for review	Adjustment	Covariates	Results	Conclusion	Risk of bias
Atkins; UK[281]	cohort; electronic database; 16. Mar - 26. Apr	community-based population (UK Biobank)	269070 (507 COVID-19 positive); COVID-19 positive mean 74 (5); rest of cohort mean 73 (4); COVID-19 positive: 61%; rest of cohort: 45%	hospitalisation, in-hospital mortality	Log	age group, sex, ethnicity, education, comorbidities (CHD, atrial fibrillation, CRB, HTN, DM, CKD, depression, DEM, AST, COPD, osteoporosis, osteoarthritis), previous disease/condition (delirium, pneumonia, falls/fragility, fractures)	dementia, type 2 diabetes, COPD, pneumonia, depression, atrial fibrillation and hypertension are independent risk factors for hospitalisation, the first five remaining statistically significant for related mortality. Chronic kidney disease and asthma were risk factors for hospitalisation in women, but not in men	specific pre-existing comorbidities are disproportionally common in hospitalised COVID-19 older adults	NOS =8
Hamer, Gale; UK[284]	cohort; electronic database; 16. Mar - 26. Apr	community-based population (UK Biobank)	334329; mean 56 (8); 46%	hospitalisation	Log	age, sex, comorbidities (underweight, overweight, obese I, obese II, DM, CVD, HTN), smoking, physical activity, alcohol abstainer, education, ethnicity	upward linear trend in likelihood of hospitalisation with increasing BMI, evident in overweight, obesity stage I and II compared to normal weight	higher likelihood of hospitalisation with increasing adiposity, even in participants with modest weight gain	NOS =7
Hamer, Kivimaeki; UK[285]	cohort; electronic database; 16. Mar - 26. Apr	community-based population (UK Biobank)	387109; mean 56 (8); 45%	hospitalisation	Log	total life score, individual behaviours (smoking, physical activity, alcohol consumption, BMI) adjusted for age, sex, education, ethnicity, comorbidities (DM, HTN, CVD)	risk ratios were raised for obesity in relation to COVID-19 hospitalisation	data suggest that adopting simple lifestyle changes could lower the risk of severe COVID-19 infection and COVID-19 hospitalisation	na
Khawaja; UK[286]	cohort; electronic database; 16. Mar - 16. Apr	community-based population (UK Biobank)	406793 (605 cases, 406188 controls); mean 68 (range 48-85); 45%	hospitalisation	Log	age, sex, ethnicity, education, townsend deprivation index, comorbidities (HTN, IHD, CRB, COPD), BMI (3 groups), alcohol consumption, smoking, laboratory values, medication	higher BMI, hypertension or COPD were major independent risk factors for hospitalisation with COVID-19	Understanding why factors increase risk of severe COVID-19 may help elucidate mechanisms and inform strategy for disease control	na
Lassale; UK[287]	cohort; electronic database; 16. Mar - 26. Apr	community-based population (UK Biobank)	340966; mean 56; not hospitalised 45%; hospitalised 56%	hospitalisation	Log	ethnicity, age, sex, education, number in household, townsend score, physical activity, alcohol, smoking, BMI, waist-to-hip-ratio, comorbidities (HTN; CVD, chronic bronchitis, ever seen a psychiatrist), laboratory values	pre-existing conditions adjusted for in the multivariate model "hospitalisation" were not found to be significantly associated with the outcome; in the age and sex adjusted model the authors found differences in risk depending on ethnicity, with black, Asian individuals and individuals categorised as having another ethnicity as compared to white, being under an increased risk of hospitalisation.	the authors found clear ethnic differences in the risk of COVID-19 hospitalisation which may have implications for health policy including the provision of prevention advice and targeted vaccination coverage	na

Study	Study design	Study population	Sample size, age (mean (SD or CI) or median (IQR)), % of males	Outcomes relevant for review	Adjust ment	Covariates	Results	Conclusion	Risk of bias
Patel; UK[288]	cohort; electronic database; 16. Mar - 14. Apr	community-based population (UK Biobank)	418794 (549 COVID-19 positive, 418245 without test); mean 65; 45%	hospitalisat ion	Log	age, sex, ethnicity, region, comorbidities (CAD, HTN, DM, heart failure, ischemic stroke, COPD, previous pneumonia, DEM (or Alzheimer), CKD), BMI, smoking, statin usage, alcohol consumption, tonwsend index, average income	race and socioeconomic deprivation were associated with an increased risk of hospitalisation. Increased risk of hospitalisation was suggested by the fully adjusted model for the following pre-existing conditions: diabetes mellitus, BMI, chronic kidney disease	higher morbidity in non-white individuals in the context of a large population	na
Bhaskaran; England[282]	cohort; electronic database; 1. Feb - 22. Jun	community-based population (OpenSAFELY England)	with HIV: 27480, without HIV 17225425; median with HIV: 48 (40-55), median without HIV 49 (34-64); HIV group: 65%; no HIV group: 50%	case mortality	Cox	model death: HIV versus no HIV adjusted for 1) age, sex; 2) in addition to model 1 index of multiple derivation, ethnicity, 3) model 2 in addition all covariates (HTN, chronic respiratory disease, asthma, chronic cardiac disease, diabetes, non-haematological cancer, haematological cancer, chronic liver disease, stroke/dementia, other neurological disease, reduced kidney function, organ transplant, asplenia, rheumatoid arthritis/lupus/psoriasis, other immunosuppressive conditions); hospitalisation: ;	people with HIV (as opposed to without) have a higher risk (nearly 3-fold) of COVID-19 death adjusting for age and sex (HR=2.90, 95% CI 1.96-4.30), this attenuated slightly after adjusting for additional variables such as multiple deprivation and ethnicity (HR=2.52, 1.70-3.73), then obesity, smoking, comorbidities (HR=2.30, 1.55-3.41). The authors also found that the association between HIV and death was larger in the group of black individuals compared with other ethnic groups (HR=3.80, 2.15-6.74 vs. 1.64, 0.92-2.90)	people with HIV might be a high-risk group for COVID-19 death, indicating a need to consider targeted policies for this group.	JBIC =11
Del Amo; Spain[283]	cohort; multicenter, 60; 1. Feb - 15. Apr	individuals with HIV receiving antiretroviral therapy	77590; 56%>=50 years; 75%	hospitalisat ion, ICU admission, and death (case mortality)	Poisso n regress ion	standardized to age and sex of the general population of Spain, estimates for HIV vs. no HIV for the outcomes COVID-19 hospital admission, COVID-19 ICU admission, COVID-19 death (case mortality) are presented	the risk standardized to the age and sex of the general population in Spain aged 20 to 79 years old for hospitalisation, ICU admission and death among HIV-positive persons receiving ART was 17.8 (CI 17.7-18.0), 2.5 (CI 2.4-2.6) and 3.7 (CI 3.6-3,8) respectively. For comparison, in the Spanish general population aged 20 to 79 years during the same period, the risk for COVID-19 diagnosis was 41.7 per 10 000 (33.0 per 10 000 after health care workers were excluded) and the risk for death was 2.1 per 10 000. The risk of hospitalisation was greater in men and person older than 70 years. There were no other pre-existing comorbidities included in the model.	The risk of adverse events (hospitalization, ICU admission, and death) among HIV-positive individuals treated with ART in Spain was increased in men and those older than 70 years (note: focus in article was different).	JBIC =7

Study	Study design	Study population	Sample size, age (mean (SD or CI) or median (IQR)), % of males	Outcomes relevant for review	Adjust ment	Covariates	Results	Conclusion	Risk of bias
Williams on; England[289]	cohort; electronic database; 1. Feb - 25. Apr	community-based population (OpenSAFELY England)	17278392; 6 groups: 18-39, 40-49, 50-59, 60-69, 70-79, 80+; 49%>=50 years; 50%	case mortality	Cox	age, sex, ethnicity, comorbidities (obesity (3 groups), DM, CANC, haematological malignancy, reduced kidney function, AST, chronic Resp, chronic cardiac disease, HTN, chronic liver disease, stroke or DEM, asplenia, Organ transplant, Rheuma, Immun, other neurological disease), smoking, IMD quintile	increasing risk for COVID-19 death associated with obesity (BMI of over 40), diabetes, severe asthma, respiratory disease, chronic heart disease, liver disease, stroke, dementia, other neurological diseases, chronic heart diseases, reduced kidney function, autoimmune diseases and other immunosuppressive condition, 2.5 fold increased risk with history of haematological malignancy; with hypertension higher risk of mortality up to the age of 70 years	early insights into factors associated with COVID-19 related deaths using detailed primary care records of 17 million patients; based on results suggest targeted use of additional protection measures for people in the identified groups	JBIC = 95 %

2.3. Definitions of pre-existing conditions

The pre-existing conditions were extracted as reported in the primary studies. For meta-analyses, the estimates for similar definitions were pooled. The following grouping was performed:

Pre-existing condition	Conditions (indications) extracted from the primary studies which falling in the category
**Circulatory diseases**	
Arrhythmia	arrhythmia, atrial fibrillation
Cardiovascular disease	cardiovascular disease, vascular disease (not further defined)
Coronary artery disease	coronary artery disease, ischemic heart disease, coronary heart disease
Heart disease	heart disease (not further defined)
Heart failure	chronic heart failure, heart failure, congestive heart failure
Hypertension	hypertension
Infarction	myocardial infarction, infarction
Peripheral vascular disease	peripheral vascular disease
Venous thromboembolism	venous thromboembolism, DVT/PE
**Immunodeficiency**	
Autoimmune condition	autoimmune condition
HIV	HIV, HIV/AIDS
Inflammatory bowel disease	inflammatory bowel disease
Immunosuppression	immunodeficiency, immunosuppressed state
Organ transplant recipients	organ transplant recipients, organ transplant history
Rheumatological disease	rheumatological disease
**Liver & Metabolic diseases**	
Chronic kidney disease	chronic kidney disease, renal disease, reduced renal function, renal failure, CKD>3, moderate/severe renal disease, kidney diseases, chronic renal disease,
Chronic liver disease	chronic liver disease, liver disease, mild liver disease, moderate/severe liver disease
Chronic liver disease/Cirrhosis	cirrhotic liver disease, liver cirrhosis
Chronic liver disease /Non-cirrhotic	non-cirrhotic liver disease
Diabetes	diabetes
Dyslipidemia or hyperlipidemia	hyperlipidemia, hypercholesterolemia, hyperlipidaemia, pure hypercholesterolemia, dyslipidemia
Hepatitis	viral hepatitis, hepatitis
**Neurological diseases & Mental health**	
Cerebrovascular or Stroke	stroke, cerebrovascular diseases, cerebral infarction, cerebrovascular accident/transient ischemic attack
Dementia	dementia, Alzheimer disease
Depression	depression
Neurological disease	neurological disease, chronic neurological disorder
Psychiatric disorder	major psychiatric disorder, schizophrenia
**Oncological diseases**	
Cancer	malignant neoplasm, malignancy, tumor, cancer any, history of cancer
Cancer/Active	cancer active, active malignancy
Cancer/ Hematological	haematological malignancy, lymphoid, myeloid
Cancer/Solid	solid tumor, solid malignancy
**Overweight, obesity, underweight**	
Obesity/BMI≥30	BMI≥30, obesity, BMI30-40vs<18.5-25, BMI30-35vs<25, BMI≥30vs<18.5-25, BMI30-40vs<30, BMI35-39vs18.5-24, BMI30-34.9vs18.5-24.9, BMI≥30vs<27, BMI≥35vs<30
Obesity/BMI≥40	BMI≥40, BMI≥40vs<25, BMI≥40vs<18.5-25, BMI≥40vs<30, severe obesity (BMI>=40 vs BMI<40), BMI40-44vs18.5-24
Overweight	BMI25-30vs<25, BMI25-30vs<18.5-25, BMI25-29.9vs<25, BMI27-29.9vs<27, Overweight
Underweight	BMI<18.5vs18.5-24, BMI<18.5vs18.5-25, BMI<18.5vs18.5-35, BMI<25vs25-34, BMI<18.5vs18.5-35, underweight
**Respiratory diseases**	
Asthma	asthma
COPD	COPD
COPD or Asthma	COPD or asthma
Interstitial lung disease	interstitial lung disease
Obstructive sleep apnea	obstructive sleep apnea
Respiratory disease	pulmonary disease, respiratory disease, lung disease, chronic lung disorder (not further defined)
Tuberculosis	tuberculosis, previous tuberculosis, current tuberculosis

Al-Sabah et al. Kuwait [122]

- Obesity: normal weight (BMI of 18.5-24.9 kg/m2), overweight (BMI of 25.0-29.9 kg/m2) and obese (BMI ≥30 kg/m2). The subjects with obesity were further stratified into classes: class I obesity was defined as a BMI of 30-34.9 kg/m2; class II obesity, by a BMI of 35-39.9 kg/m2; and morbid obesity, by a BMI ≥40 kg/m2.6
- Hypertension: as systolic blood pressure of ≥130 mmHg or diastolic blood pressure of ≥85 mmHg.7 Diabetes: a fasting blood glucose level of ≥126 mg/dL

Argenziano et al. USA [170]

- Pulmonary disease: documentation of any pulmonary disease in the patient's chart as past medical history including, but not limited to asthma, Chronic Obstructive Pulmonary Disease (COPD), obstructive sleep apnea (OSA), or interstitial lung disease (ILD).
- Renal disease: documentation of chronic kidney disease (CKD) or end stage renal disease (ESRD) in the patient's chart as past medical history.
- Rheumatological disease: documentation of any autoimmune disease in the patient's chart as past medical history including, but not limited to rheumatoid arthritis or systemic lupus erythematosus (SLE), scleroderma, sarcoidosis.

Berenguer et al. Spain [127]

- Cancer: the presence of an active solid or haematologic malignant neoplasm.
- Obesity: as a body mass index of >30 kg/m2.

Bezzio et al. Italy [128]

- IBD: clinical activity defined as a partial Mayo score ≥3 with a rectal bleeding subscore ≥1 for UC,14 15 and a Harvey- Bradshaw Index for CD ≥516

Bianchetti et al. Italy [129]

- Dementia: diagnosed according to clinical history and results of the cognitive assessment

Burn et al. Spain [131]

- Autoimmune condition: type 1 diabetes, rheumatoid arthritis, psoriasis, psoriatic arthritis, multiple sclerosis, systemic lupus erythematosus, Addison's disease, Grave's disease, Sjorgen's syndrome, Hashimoto thyroiditis, Myasthenia gravis, vasculitis, pernicious anemia, celiac disease, scleroderma, sarcoidosis, ulcerative colitis, and Crohn's disease
- Obesity: either by a diagnosis code, a record of a body mass index measurement between 30 and 60 kg/m2, or a recorded weight between 120 and 200 kg within 5 years of the index date

Busetto et al. Italy [132]

- Weight: normal weight (BMI < 25 kg/m2), overweight (BMI from 25 to < 30 kg/m2), and obesity (BMI ≥ 30 kg/m2)

Chhiba et al. USA [172]

ICD-10 code	Code diagnosis
496, 491.xx, 492.xx, any J44.x	Chronic obstructive pulmonary disease
327.23, G47.33	Obstructive sleep apnea
472, J31.0	Nonallergic rhinitis
297.06, D83.9, 279.xx, D80.6, D80.3	Common variable immunodeficiency, antibody deficiency, IgA deficiency
250, E11.9	Diabetes mellitus
414.01, 125.10	Coronary artery disease
401.9, R03.0	Hypertension

D'Silva et al. USA [177]

- Pulmonary disease included interstitial lung disease, asthma, chronic obstructive pulmonary disease or obstructive sleep apnoea.

- Rheumatic disease terms used to identify patients with systemic rheumatic disease and their associated ICD-9 and ICD-10 codes

Category	Rheumatic Disease (ICD codes)
Inflammatory arthritis	• Rheumatoid arthritis (M05%, M06%, 714%)<br>• Inflammatory arthritis or inflammatory polyarthropathy (M06.4, 714.89, 714.9)<br>• Juvenile idiopathic arthritis (M08.20, 714.3)<br>• Psoriatic arthritis or arthropathic psoriasis (L40.50, 696.0)<br>• Ankylosing spondylitis (M45.9, 720.0)
Vasculitis	• ANCA-associated vasculitis: granulomatosis with polyangiitis, eosinophilic granulomatosis with polyangiitis, microscopic polyangiitis (M31.3, M31.7, M30.0, 446.0, 446.4, 446.7, 447.6)<br>• Kawasaki disease (M30.3, 446.1)<br>• Takayasu arteritis (M31.4, 446.7)<br>• Polyarteritis nodosa (M30.0, 446.0)<br>• Giant cell arteritis/polymyalgia rheumatica (M31.6, 446.5, M35.3, 725%)*<br>• Behçet disease (M35.2, 136.1)<br>• Unspecified arteritis (I77.6, 447.6)
Other Systemic Autoimmune Diseases	• Systemic lupus erythematosus (M32%, 710.0)<br>• Sjogren's syndrome (M35.0, 710.2)<br>• Idiopathic inflammatory myositis: dermatomyositis, polymyositis, statin-associated autoimmune myositis, unspecified myositis (G72.49, G72.41, M33, 710.3, 710.4)<br>• Systemic sclerosis (M34.0, M34.1, M34.8%, M34.9, 710.1)<br>• Mixed connective tissue disease (M35.1, 710.9)<br>• Antiphospholipid syndrome (D68.61, 239.81)<br>• Sarcoidosis (D86.0, D86.9, 135%)<br>• IgG4-related disease (M35)

[illegible]

Di Castelnuovo et al. Italy [139]

- Chronic kidney disease was classified as: stage 1: normal or increased glomerular filtration rate (eGFR) (≥90 mL/min/1.73 m2); stage 2: kidney damage with mild reduction in eGFR (60-89 mL/min/1.73 m2); stage 3a: moderate reduction in eGFR (45-59 mL/min/1.73 m2); stage 3 b: moderate reduction in eGFR (30-44 mL/min/1.73 m2); stage 4: severe reduction in eGFR (15-29 mL/min/1.73 m2); stage 5: kidney failure (eGFR <15 mL/min/1.73 m2 or dialysis)

Docherty et al. UK [140]

- Comorbidities: as per Charlson Comorbidities Index. Obesity - clinician defined

Galloway et al. UK [141]

- Chronic lung disease: predominantly asthma, COPD and interstitial lung disease

Gu, T, Mack et al. USA [290]

- Based on ICD- 9

Disease	Code
Respiratory Diseases	At least one of the following observed phecodes and their subcodes: 464, 465, 465.2, 465.4, 470, 471, 472, 473, 473.1, 473.3, 473.4, 474, 474.1, 474.2, 475, 475.9, 476, 477, 478, 479, 480, 480.1, 480.11, 480.12, 480.13, 480.2, 480.3, 480.5, 481, 483, 495, 495.1, 495.11, 495.2, 496, 496.1, 496.2, 496.21, 496.3, 497, 498, 499, 500, 500.1, 500.2, 501, 502, 503, 504, 504.1, 505, 506, 507, 508, 509, 509.1, 509.2, 509.3, 509.5, 509.8, 510, 510.2, 512, 512.1, 512.2, 512.3, 512.7, 512.8, 512.9, 513, 513.3, 513.31, 513.32, 513.4, 513.8, 514, 514.1, 514.2, 516, 516.1, 519, 519.1, 519.2, 519.8, 519.9
Circulatory Diseases	At least one of the following observed phecodes and their subcodes: 394, 394.1, 394.2, 394.3, 394.4, 394.7, 395, 395.1, 395.2, 395.3, 395.4, 395.6, 396, 401, 401.1, 401.2, 401.21, 401.22, 401.3, 411, 411.1, 411.2, 411.3, 411.4, 411.41, 411.8, 411.9, 414, 414.2, 415, 415.1, 415.11, 415.2, 415.21, 416, 418, 418.1, 420, 420.1, 420.2, 420.21, 420.22, 420.3, 425, 425.1, 425.11, 425.12, 425.2, 425.8, 426, 426.2, 426.21, 426.22,

	426.23, 426.24, 426.25, 426.3, 426.31, 426.32, 426.4, 426.7, 426.8, 426.9, 426.91, 426.92, 427, 427.1, 427.11, 427.12, 427.2, 427.21, 427.22, 427.3, 427.4, 427.41, 427.42, 427.5, 427.6, 427.61, 427.7, 427.8, 427.9, 428, 428.1, 428.2, 428.3, 428.4, 429, 429.1, 429.2, 429.3, 429.9, 430, 430.1, 430.2, 430.3, 433, 433.1, 433.11, 433.12, 433.2, 433.21, 433.3, 433.31, 433.32, 433.5, 433.6, 433.8, 440, 440.1, 440.2, 440.21, 440.22, 440.9, 441, 441.1, 441.2, 442, 442.1, 442.11, 442.2, 442.3, 442.4, 442.8, 443, 443.1, 443.7, 443.8, 443.9, 444, 444.1, 444.2, 444.5, 446, 446.1, 446.2, 446.3, 446.4, 446.5, 446.6, 446.7, 446.8, 446.9, 447, 447.1, 447.7, 448, 450, 451, 451.2, 452, 452.1, 452.2, 452.8, 453, 454, 454.1, 454.11, 455, 456, 457, 457.2, 457.3, 458, 458.1, 458.2, 458.9, 459, 459.1, 459.7, 459.9
Any Cancer	At least one of the following observed phecodes and their subcodes: 145, 145.2, 145.3, 145.4, 149, 149.1, 149.2, 149.3, 149.4, 149.5, 149.9, 150, 151, 153, 153.2, 153.3, 155, 155.1, 157, 158, 159, 159.2, 159.3, 159.4, 164, 165, 165.1, 170, 170.1, 170.2, 172, 172.1, 172.11, 172.2, 172.21, 172.22, 172.3, 174, 174.1, 174.11, 175, 180, 180.1, 180.3, 182, 184, 184.1, 184.11, 184.2, 185, 187, 187.1, 187.2, 189, 189.1, 189.11, 189.12, 189.2, 189.21, 189.4, 190, 191, 191.1, 191.11, 193, 194, 195, 195.1, 195.3, 196, 197, 198, 198.1, 198.2, 198.3, 198.4, 198.5, 198.6, 198.7, 199.4, 200, 200.1, 201, 202, 202.2, 202.21, 202.22, 202.23, 202.24, 204, 204.1, 204.11, 204.12, 204.2, 204.21, 204.22, 204.3, 204.4, 209
Type 2 Diabetes	At least one of the following observed phecode and their subcodes: 250.2
Kidney Diseases	At least one of the following observed phecodes and their subcodes: 585
Liver Diseases	At least one of the following observed phecodes and their subcodes: 571
Autoimmune Diseases	At least one of the following observed phecodes and their subcodes: 242.1, 250.1, 335, 557.1, 694.1, 695.4, 696.4, 697, 704.1, 714.1, 717

Gupta et al. USA [182]

Conditions	Definition
Asthma	Per chart review
Atrial fibrillation/flutter	Per chart review
Bone marrow transplant	Per chart review
Cancer	Per chart review; active malignancy (other than non-melanoma skin cancer) treated in the past year. Defined as cancer of the lung, breast, colorectal, prostate, gastric, pancreatic, melanoma, ovarian, brain, or other
Chronic kidney disease	Baseline eGFR< 60 on at least two consecutive values at least 12 weeks apart prior to hospital admission. If not available, defined as per chart review
Chronic liver disease	Cirrhosis, alcohol-related liver disease, nonalcoholic fatty liver disease, autoimmune hepatitis, hepatitis B or hepatitis C, primary biliary cirrhosis, or other
Chronic obstructive pulmonary disease	Per chart review
Congestive heart failure	Per chart review; heart failure with preserved versus reduced ejection fraction
Coronary artery disease	Per chart review; any history of angina, myocardial infarction, or coronary artery bypass graft surgery
Diabetes mellitus	Per chart review; insulin versus non-insulin dependent
End-stage kidney disease	Per chart review; on hemodialysis or peritoneal dialysis
HIV/AIDS	Per chart review
Hypertension	Per chart review
Solid organ transplant	Per chart review (kidney, liver, heart, lung, other)

Halasz et al. Italy [146]

- Weight: underweight (under 18.5 kg/m2); normal weight (18.5–25 kg/m2); overweight (25–30 kg/m2); obese class I (30–35 kg/m2); obese class II (35–40 kg/m2) and obese class III (>40 kg/m2)

Harmouch et al. USA [183]

Only the conditions reported in the multivariate regression model are extracted.

Category Description	ICD-10-CM Codes
Vascular Disease	E08.51, E08.52, E09.51, E09.52, E10.51, E10.52, E11.51, E11.52, E13.51, E13.52, I70.0, I70.1, I70.20-, I70.21-, I70.22-, I70.29-, I70.30-, I70.31-, I70.32-, I70.39-, I70.40, I70.41-, I70.42-, I70.49-, I70.50-, I70.51-, I70.52-, I70.59-, I70.60-, I70.61-, I70.62-, I70.69-, I70.70-, I70.71-, I70.72-, I70.79-, I70.92, I71.2, I71.4, I71.6, I71.9, I72.-, I73.1, I73.8-, I73.9, I77.0-I77.6, I77.81-, I77.89, I78.0, I79.-, I80.1-, I80.20-, I80.21-, I80.23-, I80.29-, I82.0, I82.21-, I82.22-, I82.29-, I82.3, I82.40-, I82.41-, I82.42-, I82.43-, I82.44-, I82.49-, I82.4Y-, I82.4Z-, I82.50-, I82.51-, I82.52-, I82.53-, I82.54-, I82.59-, I82.5Y-, I82.5Z-, I82.62-, I82.72-, I82.A1-, I82.A2-, I82.B1-, I82.B2-, I82.C1-, I82.C2-, K55.1, K55.8, K55.9, M31.8, M31.9
Chronic Kidney Disease, Stage 3	N18.3

Hur et al. USA [186]

- Cardiovascular disease: myocardial infarction, cerebrovascular accident, congestive heart failure, valvular heart disease, and arrhythmias.

- Pulmonary disease: asthma, chronic obstructive pulmonary disease, and interstitial lung disease.

Kim L. et al. USA [192]

Chronic Lung Disease	Active tuberculosis, Asbestosis, Asthma/Reactive airway disease<br>Bronchiectasis, Bronchiolitis obliterans, Chronic bronchitis<br>Chronic respiratory failure, Cystic fibrosis, Emphysema/Chronic Obstructive Pulmonary Disease, Interstitial lung disease, Obstructive sleep apnea<br>Oxygen dependent, Pulmonary fibrosis, Restrictive lung disease<br>Sarcoidosis
Diabetes Mellitus	
Other Chronic Metabolic Disease (except Diabetes Mellitus)	Adrenal Disorders (Addison's, Adrenal Insufficiency, Cushing Syndrome, Congenital Adrenal Hyperplasia), Glycogen or other storage diseases,<br>Hyper/Hypo function of pituitary gland, Inborn errors of metabolism<br>Metabolic Syndrome, Parathyroid Dysfunction (Hyperparathyroidism, Hypoparathyroidism), Thyroid dysfunction (Grave's disease, Hashimoto's Disease, Hyperthyroidism, Hypothyroidism)
Blood Disorders/Hematologic Conditions	Alpha thalassemia, Aplastic anemia, Beta thalassemia,<br>Coagulopathy (Factor V Leiden, Von Willebrand Disease), Hemoglobin S-beta thalassemia, Leukopenia, Myelodysplastic syndrome, Neutropenia, Pancytopenia, Polycythemia vera, Sickle cell disease, Splenectomy/Asplenia<br>Thrombocytopenia
Cardiovascular Disease (except hypertension)	Aortic aneurysm, history of; Aortic/Mitral/Tricuspid/Pulmonic valve replacement, history of; Aortic regurgitation; Aortic stenosis; Atherosclerotic cardiovascular disease; Atrial fibrillation; Atrioventricular blocks; Automated implantable devices/Pacemaker; Bundle branch block; Cardiomyopathy; Carotid stenosis; Cerebral vascular accident/Incident/Stroke, history of; Congenital heart disease; Coronary artery bypass grafting, history of; Coronary artery disease; Deep vein thrombosis, history of; Heart failure/congestive heart failure; Myocardial infarction, history of; Mitral regurgitation; Mitral stenosis; Peripheral artery disease; Peripheral vascular disease; Pulmonary embolism, history of; Pulmonary hypertension; Pulmonic regurgitation; Pulmonic stenosis; Transient ischemic attack, history of; Tricuspid regurgitation; Tricuspid stenosis; Ventricular fibrillation, history of; Ventricular tachycardia, history of
Hypertension	
Neurologic Disorder	Amyotrophic lateral sclerosis, Cerebral palsy, Cognitive dysfunction, Dementia/Alzheimer's disease, Developmental delay, Down syndrome/Trisomy 21, Edward's syndrome/Trisomy 18, Epilepsy/seizure/seizure disorder, Mitochondrial disorder, Multiple sclerosis,<br>Muscular dystrophy, Myasthenia gravis, Neural tube defects/Spina bifida,<br>Neuropathy, Parkinson's disease, Plegias/Paralysis/Quadriplegia, Scoliosis/Kyphoscoliosis, Traumatic brain injury, history of
Immunocompromised Conditions	AIDS or CD4 count <200, Complement deficiency,<br>Graft vs. host disease, HIV Infection, Immunoglobulin, deficiency/Immunodeficiency, Immunosuppressive therapy (within the last 12 months prior to admission), Leukemia, Lymphoma/Hodgkins/Non-Hodgkins, Metastatic cancer, Multiple myeloma, Solid organ malignancy, Steroid therapy (within 2 weeks of admission), Transplant, hematopoietic stem cell (Bone marrow transplant, peripheral stem cell transplant), history of ; Transplant, solid organ, history of
Renal Disease	Chronic kidney disease/chronic renal insufficiency; Dialysis ; End stage renal disease ; Glomerulonephritis; Nephrotic syndrome; Polycystic kidney disease
Gastrointestinal/Liver Disease	Alcoholic hepatitis; Autoimmune hepatitis, Barrett's esophagitis, Chronic liver disease, Chronic pancreatitis, Cirrhosis/End stage liver disease,<br>Crohn's disease, Esophageal varices, Esophageal strictures, Hepatitis B, chronic ; Hepatitis C, chronic; Non-alcoholic fatty liver disease/Non-alcoholic steatohepatitis; Ulcerative colitis
Rheumatologic/Autoimmune Conditions	Ankylosing spondylitis, Dermatomyositis, Juvenile idiopathic arthritis,<br>Kawasaki disease, Microscopic polyangiitis, Polyarteritis nodosum,<br>Polymyalgia rheumatica, Polymyositis, Psoriatic arthritis, Rheumatoid arthritis, Systemic Lupus Erythematosus/Lupus, Systemic sclerosis<br>Takayasu arteritis , Temporal/Giant Cell arteritis,Vasculitis, other

Lee et al. UK [149]

- Prostate (C61), Lung (C34), Mesothelial and soft tissue (C45–C49), Urinary tract (C64–C68), Colorectal (C18–C21), CNS (C69–C72), Respiratory organs and intrathoracic organs (not lung; C30–C39), Lip, oral cavity, and pharynx (C00–C14), Breast (C50), Female genital organs (C51–C58), Myeloma (C90), Leukaemia (C91–C95), Lymphoma (C81–C85), Other haematological (C86, C88, C96), Digestive organs, non-colorectal (C15–C17, C22–C26)

Mendy et al. USA [199]

- ICD-10: obesity (E66), diabetes (E10 and E11), pure hypercholesterolemia (E78.0), asthma (J45), chronic obstructive pulmonary disease (COPD) (J44), chronic kidney disease, (N18), cardiovascular disease (I00-I99), neoplasm or a history of neoplasm (C00-D49), osteoarthritis (M15-M19), and vitamin D deficiency (E55).

Nakeshbandi et al. USA [204]

- BMI: three groups: normal (BMI 18.50–24.99 kg/m2), overweight (BMI 25.00–29.99 kg/m2), and obese (BMI ≥ 30.00 kg/m2).

Price-Haywood et al. USA [211]

- Obesity was determined by the presence of diagnosis codes or a body-mass index (BMI) of 30 or more that was recorded during previous clinical encounters (1727 patients had a BMI of ≥30; 1071 had a BMI of <30; and 683 had missing data).E66, Z72.0, J45, J44, E10, E11, I10, I50, I25, N18, Z94, K70 through K77, C0 through D49, and B20

Reilev et al. Denmark [156]

ICD-10- and ATC-codes used to define drug and comorbidity.

	Coding system	Codes
Medical history[2]		
Chronic lung disease	ICD-10<br>ATC	J41-J47<br>R03AK, R03AL, R03BA, R03AC12, R03AC13, R03AC18, R03AC19, R03CC12, R03BB04, R03BB05, R03BB06, R03BB07
Hypertension	ICD-10<br>ATC	I10 I11 I12 I13 I15<br>C08, C03A, C07, C09
Ischemic heart disease	ICD-10<br>ATC	I20 I21 I22 I23 I24 I25<br>N02BA C01DA B01AC24
Heart failure	ICD-10	I099A I110 I130 I132 I50
Atrial fibrillation	ICD-10	I48
Stroke	ICD-10	I60 I61 I62 I63 I64 I69
Diabetes	ICD-10<br>ATC	E10 E11 E13 E14<br>A10
Dementia	ICD-10<br>ATC	F00 F01 F02 F03 F1073 F1173 F1273 F1373 F1473 F1573 F1673 F1873 F1973<br>N06D
Any cancer	ICD-10	C00-C97, excluding C44
Chronic liver disease	ICD-10	K700-K704 K709 K71-K74 K760 K766 B150 B160 B162 B18 B190 I85
Hospital-diagnosed kidney disease	ICD-10	I12 I13 N00-N05 N07 N08 N11 N14 N18 N19 E102 E112 E142
Alcohol abuse	ICD-10<br>ATC	F10 E244 G312 G621 G721 I426 K292 K70 K852 K860 Q860 Z502 Z714 Z721<br>N07BB
Substance abuse	ICD-10<br>ATC	F11-F19<br>N07BC
Organ transplantation	ICD-10	Z94
Medical overweight and obesity	ICD-10<br>ATC	E66<br>A08
Severe mental illness (schizophrenia, schizoaffective disorder, or bipolar disorder)	ICD-10<br>ATC	F20 F25 F30 F31<br>N05AN

[2]Medical history is based on an ever-recording of hospital discharge diagnoses, with or without combination with drug redemption data.
ACE: angiotensin-converting enzyme inhibitor; ARB: angiotensin receptor blocker; NSAID: non-steroidal anti-inflammatory drugs.

Rentsch et al. USA [212]

- Conditions based on International Classification of Diseases, Tenth Revision, Clinical Modification (ICD-10-CM) Diagnosis Codes

Comorbid conditions			ICD-10-CM codes
	Asthma		J45.X
	Cancer		
		Cancer	C00.X-C43.X, C45.X-C76.X, C80.X-C96.X, C7A.X
		Metastatic cancer	C77.X-C79.X
	Chronic obstructive pulmonary disease		J41.X, J42.X, J43.X, J44.X
	Chronic kidney disease		I12.0X, I13.1X, N03.2X-N03.7X, N18.X, N19.X, N05.2X-N05.7X, N25.0X, Z49.0X - Z49.2X, Z94.0X, Z99.2X
	Diabetes mellitus		E08.X, E10.X, E11.X, E13.X
	Hypertension		I10.X-I13.X, I15.X, I16.X
	Liver disease		
		Hepatitis B virus	B16.X, B18.0X, B18.1X, B19.1X, Z22.51
		Hepatitis C virus	B17.10, B17.11, B18.2, B19.20, B19.21, Z22.52
		Hepatic decompensation	I85.01, K65.2, K70.31, K72.1X, K72.9X, K76.7, R18.8
		Other mild liver disease	B18.8X, B18.9X, K70.0X-K70.2X, K70.30, K70.9X, K71.3X-K71.5X, K71.7X, K73.X, K74.X, K76.0X, K76.2X-K76.4X, K76.8X, K76.9X, Z94.4

		Other severe liver disease	K76.6, I85.00, I85.9X, I86.4, I98.2X, K70.4X, K71.1X, K76.5X
	Vascular disease		
		Acute myocardial infarction	I21.X (not including I21.AX), I22.X
		Cardiomyopathy	I42.X, I43.X
		Coronary heart disease	I20.X, I24.X, I25.10, I25.110, I25.2, I25.3, I25.41, I25.42, I25.5, I25.700, I25.710, I25.720, I25.730, I25.750, I25.760, I25.790, I25.8X, I25.9
		Heart failure	I09.9, I11.0, I25.5, I13.0, I13.2, I50.X, P29.0
		Cerebrovascular accident	I60.X-I69.X, G45.X, G46.X, H34.0
		Peripheral vascular disease	I70.X, I71.X, I73.1-I73.9, I77.1, I79.0, I79.2, K55.1X, K55.8X, K55.9X, Z95.8X, Z95.9

Robilotti et al. USA [213]

- Cardiac disorder: Heart failure, myocardial infarction, valvular replacement or cardiomyopathy.
- Corticosteroids (equivalent of prednisone 20 mg or higher) for at least 10 d.
- Chronic lymphopenia: Absolute lymphocyte count <500 per microliter over five previous consecutive measurements.
- ICI therapy consisted of the following, given within 90 d: pembrolizumab (18), nivolumab (5), atezolizumab (3), avelumab (1), durvalumab (1), ipilimumab (1), nivolumab + ipilimumab (1) and pembrolizumab followed by nivolumab (1)

Seiglie et al. USA [215]

- Diabetes: 1) past medical history of diabetes as documented in the medical record and manually retrieved on chart review, 2) HbA1c≥6.5% during the index hospitalization, or 3) random blood glucose≥200 mg/dL at admission to the hospital with supportive history by chart review.

2.4. List of estimates excluded from reporting

If indications were considered not explicitly defined or surrogate for a pre-existing condition, they were excluded from the reporting. The estimates for the following conditions were excluded from the reporting:

- Benzodiazepines and derivates use, Antipsychotic use, Antidepressant use, Vitamin D Deficiency were considered surrogate
- Thyroid alterations, Osteoarthritis, Hematologic disease, CVD or CBR (composite of cardiovascular and cerebrovascular diseases) were considered not specifically defined
- Hemiplegia or paraplegia were considered syndrome or symptom but not a comorbidity
- Peptic ulcer disease, Allergic rhinitis, Rhinosinusitis were considered not relevant for decision-making
- Due to increasing complexity of the review, the following outcomes were excluded from the reporting: ICU mortality, mortality in the patients undergoing mechanical ventilation, ICU admission among SARS-CoV-2-positive individuals.

2.5. Results of the meta-analyses

The following tables present the pooled estimates per outcome and group of pre-existing conditions: (i) Tables 5.1 – 5.7 for hospitalisation; (ii) Tables 6.1 – 6.7 for in-hospital mortality; (iii) Tables 7.1 – 7.7 for mortality among SARS-CoV-2-positively tested persons (case-mortality); (iv) Tables 8.1 – 8.7 for admission to an intensive care unit (ICU) among hospitalised patients, and (v) Tables 9.1 – 9.7 for mechanical ventilation or intubation. Figures 3-5 illustrate the ranges for the estimates with between-study heterogeneity I^2 >40%.

Table 5. 1 Risk of hospitalisation in persons with liver and metabolic diseases: meta-analysis and confidence in the estimate (GRADE)

Region	Condition	Estimate (95% CI)	I^2 (%)	Studies	Patients	Indirectness	Inconsistency	Imprecision	Bias	Publ. Bias	Quality
AMR: North	Chronic kidney disease	OR range 0.84-3.47	81.5	5[181,199,209,212,224]	11,055	no	yes	no	no	no	+++
AMR: North	Chronic liver disease	OR 1 (0.59-1.68)	0.0	1[181]	799	no	na	yes	no	no	+++
AMR: North	Chronic liver disease	RR 1.3 (1.1-1.6)	0.0	1[219]	500	no	na	no	no	no	++++
AMR: North	Diabetes	OR 2.03 (1.73-2.38)	37.6	8[171,178,181,191,199,209,212,224]	12,917	no	no	no	no	no	++++
AMR: North	Diabetes	RR 1.16 (1-1.36)	0.0	1[172]	1,526	no	na	yes	no	no	+++
AMR: North	Dyslipidemia or hyperlipidemia	OR range 0.62-9.3	91.6	2[199,209]	5,968	no	yes	yes	no	no	++
AMR: South/Latin	Chronic kidney disease	OR 2.21 (1.94-2.51)	0.0	3[230,233,235]	111,013	no	no	no	no	no	++++
AMR: South/Latin	Diabetes	OR range 1.34-2.14	88.8	3[230,233,235]	111,013	no	yes	no	no	no	+++
EUR	Chronic kidney disease	HR range 1.24-1.9	76.3	2[131,159]	11,580	no	yes	yes	no	no	++
EUR	Chronic kidney disease	OR range 0.65-2.9	88.7	2[156,166]	11,444	no	yes	yes	no	no	++
EUR	Chronic liver disease	OR 1.86 (0.89-3.9)	38.8	2 [156,166]	11,444	no	no	yes	no	no	+++
EUR	Diabetes	HR range 1.31-1.50	44.2	2[131,159]	11,580	no	yes	no	no	no	+++
EUR	Diabetes	OR 1.77 (1.52-2.07)	0.0	3[151,156,166]	12,226	no	no	no	no	no	++++
EUR	Dyslipidemia or hyperlipidemia	HR 1.13 (0.98-1.3)	37.5	2[131,159]	11,580	no	no	yes	no	no	+++
EUR	Dyslipidemia or hyperlipidemia	OR 0.74 (0.36-1.54)	0.0	1[166]	322	no	na	yes	no	no	+++

AMR, regions of America; AFR, African region; HR, hazard ratio; EMR, Eastern Mediterranean region; EUR, European region; na, not applicable; OR, odds ratio; RR, risk ratio; WPR, Western Pacific region; WPR, Western Pacific region

Table 5. 2 Risk of hospitalisation in persons with respiratory diseases: meta-analysis and confidence in the estimate (GRADE)

Region	Condition	Estimate (95% CI)	I^2 (%)	Studies	Patients	Indirectness	Inconsistency	Imprecision	Bias	Publ. Bias	Quality
AMR: North	Asthma	OR range 0.94-1.92	66.6	3[171,199,224]	5,444	no	yes	yes	no	no	++
AMR: North	Asthma	RR 0.96 (0.77-1.19)	0.0	1[172]	1,526	no	na	yes	no	no	+++
AMR: North	COPD	OR 1.72 (1.23-2.4)	15.2	4[171,199,224]	6,029	no	no	no	no	no	++++
AMR: North	COPD	RR 1.18 (0.93-1.5)	0.0	1[172]	1,526	no	na	yes	no	no	+++
AMR: North	COPD or asthma	OR 1.06 (0.87-1.29)	0.0	2[178,209]	5,721	no	no	yes	no	no	+++
AMR: North	Obstructive sleep apnea	RR 1.23 (1.01-1.49)	0.0	1[172]	1,526	no	na	no	no	no	++++
AMR: North	Respiratory disease	OR 0.81 (0.53-1.23)	0.0	1[181]	799	no	na	yes	no	no	+++
AMR: South/Latin	Asthma	OR 0.73 (0.65-0.81)	0.0	1[233]	89,756	no	na	no	no	no	++++
AMR: South/Latin	COPD	OR 1.47 (1.3-1.67)	0.0	2[230,233]	100,300	no	no	no	no	no	++++
AMR: South/Latin	Respiratory disease	OR 1.46 (1.12-1.9)	0.0	1[235]	10,713	no	na	no	no	no	++++
EUR	Asthma	OR 1.98 (0.52-7.43)	0.0	1[166]	322	no	na	yes	no	no	+++
EUR	COPD	HR range 1-1.9	94.1	2[131,159]	11,580	no	yes	yes	no	no	++
EUR	COPD	OR 2.43 (0.73-11.09)	0.0	1[166]	322	no	na	yes	no	no	+++
EUR	Respiratory disease	OR range 0.94-1.8	76.4	2[151,156]	11,904	no	yes	yes	no	no	++
WPR	Tuberculosis	RR 1.2 (1.04-1.38)	0.0	1[260]	530	no	na	no	no	no	++++

AMR, regions of America; AFR, African region; HR, hazard ratio; EMR, Eastern Mediterranean region; EUR, European region; na, not applicable; OR, odds ratio; RR, risk ratio; WPR, Western Pacific region; WPR, Western Pacific region

Table 5. 3 Risk of hospitalisation in persons with circulatory diseases: meta-analysis and confidence in the estimate (GRADE)

Region	Condition	Estimate (95% CI)	I^2 (%)	Studies	Patients	Indirectness	Inconsistency	Imprecision	Bias	Publ. Bias	Quality
AMR: North	Arrhythmia	OR 1.49 (1.03-2.14)	0.0	1[224]	3,703	no	na	no	no	no	++++
AMR: North	Cardiovascular disease	OR range 1.35-4.39	81.1	4[171,181,199,212]	3,125	no	yes	no	no	no	+++
AMR: North	Coronary artery disease	OR 1.18 (0.95-1.46)	0.0	2[209,224]	8,982	no	no	yes	no	no	+++
AMR: North	Coronary artery disease	RR 1.02 (0.8-1.29)	0.0	1[172]	1,526	no	na	yes	no	no	+++

Region	Condition	Estimate (95% CI)	I^2 (%)	Studies	Patients	Indirectness	Inconsistency	Imprecision	Bias	Publ. Bias	Quality
AMR: North	Heart failure	OR range 0.69-4.43	80.7	4[128,178,209,224]	10,476	no	yes	yes	no	no	++
AMR: North	Hypertension	OR range 1.14-1.78	59.3	5[128,178,209,212,224]	11,061	no	yes	no	no	no	+++
AMR: North	Hypertension	RR 1.14 (0.97-1.33)	0.0	1[172]	1,526	no	na	yes	no	no	+++
AMR: North	Peripheral vascular disease	OR 0.81 (0.5-1.3)	0.0	1[224]	3,703	no	na	yes	no	no	+++
AMR: North	V.thromboembolism	OR 0.87 (0.55-1.37)	0.0	1[224]	3,703	no	na	yes	no	no	+++
AMR: South/Latin	Cardiovascular disease	OR range 1.09-1.3	64.7	2[233,235]	100,469	no	yes	yes	no	no	++
AMR: South/Latin	Hypertension	OR range 1.25-1.54	73.0	2[230,233]	100,300	no	yes	no	no	no	+++
EUR	Arrhythmia	HR 1.5 (1.2-1.9)	0.0	1[159]	2,143	no	na	no	no	no	++++
EUR	Arrhythmia	OR 1.39 (1.17-1.65)	0.0	2[229,166]	11,444	no	no	no	no	no	++++
EUR	Cardiovascular disease	HR 1.2 (0.8-1.8)	0.0	1[159]	2,143	no	na	yes	no	no	+++
EUR	Cardiovascular disease	OR 1.43 (0.66-3.09)	12.3	2[151,166]	1,104	no	no	yes	no	no	+++
EUR	Coronary artery disease	HR 1.3 (1-1.7)	0.0	1[159]	2,143	no	na	yes	no	no	+++
EUR	Coronary artery disease	OR 1.4 (1.2-1.7)	0.0	1[229]	11,122	no	na	no	no	no	++++
EUR	Heart disease	HR 1.1 (1.05-1.16)	0.0	1[131]	9,437	no	na	no	no	no	++++
EUR	Heart failure	HR 1.6 (1.2-2.1)	0.0	1[159]	2,143	no	na	no	no	no	++++
EUR	Heart failure	OR 2.6 (2-3.4)	0.0	1[229]	11,122	no	na	no	no	no	++++
EUR	Hypertension	HR range 1.05-1.4	74.7	2[131,159]	11,580	no	yes	yes	no	no	++
EUR	Hypertension	OR 1.69 (1.51-1.89)	0.0	3[151,229,166]	12,226	no	no	no	no	no	++++

AMR, regions of America; AFR, African region; HR, hazard ratio; EMR, Eastern Mediterranean region; EUR, European region; na, not applicable; OR, odds ratio; RR, risk ratio; WPR, Western Pacific region; WPR, Western Pacific region

Table 5. 4 Risk of hospitalisation in persons with overweight, obesity or underweight: meta-analysis and confidence in the estimate (GRADE)

Region	Condition	Estimate (95% CI)	I^2 (%)	Studies	Patients	Indirectness	Inconsistency	Imprecision	Bias	Publ. Bias	Quality
AMR: North	Obesity/BMI≥30	OR 1.63 (1.43-1.84)	18.0	7[178,181,191,199,209,211,224]	14,761	no	no	no	no	no	++++
AMR: North	Obesity/BMI≥30	RR 1.12 (1.04-1.21)	0.0	2[172,218]	6,444	no	no	no	no	no	++++
AMR: North	Obesity/BMI≥40	OR 2.45 (1.78-3.36)	0.0	1[209]	5,279	no	na	no	no	no	++++

Region	Condition	Estimate (95% CI)	I^2 (%)	Studies	Patients	Indirectness	Inconsistency	Imprecision	Bias	Publ. Bias	Quality
AMR: North	Overweight	OR 1.33 (1.16-1.53)	0.0	3[181,209,224]	9,781	no	no	no	no	no	++++
AMR: North	Underweight	OR 1.48 (0.19-11.8)	0.0	1[181]	799	no	na	yes	no	no	+++
AMR: South/Latin	Obesity/BMI≥30	OR range 1.4-1.74	56.2	3[230,233,235]	111,013	no	yes	no	no	no	+++
EUR	Obesity/BMI≥30	HR 1.59 (1.52-1.66)	0.0	2[131,159]	11,580	no	no	no	no	no	++++
EUR	Obesity/BMI≥30	OR range 0.99-2.1	96.6	3[151,156,166]	12,226	no	yes	yes	no	no	++

AMR, regions of America; AFR, African region; HR, hazard ratio; EMR, Eastern Mediterranean region; EUR, European region; na, not applicable; OR, odds ratio; RR, risk ratio; WPR, Western Pacific region

Table 5. 5 Risk of hospitalisation in persons with immunodeficiency: meta-analysis and confidence in the estimate (GRADE)

Region	Condition	Estimate (95% CI)	I^2 (%)	Studies	Patients	Indirectness	Inconsistency	Imprecision	Bias	Publ. Bias	Quality
AMR: North	Autoimmune condition	OR 1.24 (0.83-1.85)	0	1[181]	799	no	na	yes	no	no	+++
AMR: North	Inflammatory bowel disease	RR 1.1 (0.74-1.4)	0	1[220]	464	no	na	yes	no	no	+++
AMR: North	Immunosuppression	RR 1.14 (0.75-1.75)	0	1[172]	1,526	no	na	yes	no	no	+++
AMR: North	Rheumatological disease	OR 1.1 (0.51-2.38)	0	1[177]	156	no	na	yes	no	no	+++
AMR: South/Latin	Immunosuppression	OR 1.85 (1.59-2.15)	0	1[233]	89,756	no	na	no	no	no	++++
EUR	Autoimmune condition	HR 1.08 (1.01-1.17)	0	1[131]	9,437	no	na	no	no	no	++++
EUR	Autoimmune condition	OR 1.3 (0.45-4.01)	0	1[166]	322	no	na	yes	no	no	+++
EUR	Organ transplant recipients	OR 3.4 (1.7-6.6)	0	1[229]	11,122	no	na	no	no	no	++++
EUR	Rheumatological disease	OR 1.5 (1.1-1.9)	0	1[229]	11,122	no	na	no	no	no	++++

AMR, regions of America; AFR, African region; HR, hazard ratio; EMR, Eastern Mediterranean region; EUR, European region; na, not applicable; OR, odds ratio; RR, risk ratio; WPR, Western Pacific region

Table 5. 6 Risk of hospitalisation in persons with neurological diseases or mental health disorders: meta-analysis and confidence in the estimate (GRADE)

Region	Condition	Estimate (95% CI)	I^2 (%)	Studies	Patients	Indirectness	Inconsistency	Imprecision	Bias	Publ. Bias	Quality
AMR: North	Cerebrovascular or stroke	OR 2.25 (1.42-3.58)	0.0	1[224]	3,703	no	na	no	no	no	++++
AMR: North	Dementia	OR 3.6 (2.12-6.09)	0.0	1[224]	3,703	no	na	no	no	no	++++
AMR: North	Depression	OR 0.97 (0.49-1.92)	0.0	1[171]	1,052	no	na	yes	no	no	+++
EUR	Cerebrovascular or stroke	OR 1.3 (1.08-1.56)	0.0	2[156,166]	11,444	no	no	no	no	no	++++
EUR	Dementia	HR range 0.59-1.2	93.4	2[131,159]	11,580	no	yes	yes	no	no	++
EUR	Dementia	OR range 0.5-1.52	50.0	3[151,156,166]	12,226	no	yes	yes	no	no	++
EUR	Depression	OR range 0.94-6.06	77.0	2[151,166]	1,104	no	yes	yes	no	no	++
EUR	Psychiatric disorder	OR 1.72 (1.05-2.84)	15.6	2[151,156]	11,904	no	no	no	no	no	++++

AMR, regions of America; AFR, African region; HR, hazard ratio; EMR, Eastern Mediterranean region; EUR, European region; na, not applicable; OR, odds ratio; RR, risk ratio; WPR, Western Pacific region

Table 5. 7 Risk of hospitalisation in persons with oncological diseases: meta-analysis and confidence in the estimate (GRADE)

Region	Condition	Estimate (95% CI)	I^2 (%)	Studies	Patients	Indirectness	Inconsistency	Imprecision	Bias	Publ. Bias	Quality
AMR: North	Cancer	OR 1.05 (0.87-1.27)	17.8	5[171,181,199,209,224]	11,522	no	no	yes	no	no	+++
EUR	Cancer	HR range 1.08-1.4	80.1	2[131,159]	11,580	no	yes	yes	no	no	++
EUR	Cancer	OR range 0.66-1.4	67.0	2[156,166]	11,444	no	yes	yes	no	no	++

AMR, regions of America; AFR, African region; HR, hazard ratio; EMR, Eastern Mediterranean region; EUR, European region; na, not applicable; OR, odds ratio; RR, risk ratio; WPR, Western Pacific region

Table 6. 1 Risk of death (in-hospital mortality) in persons with liver and metabolic diseases: meta-analysis and confidence in the estimate (GRADE)

Region	Condition	Estimate (95% CI)	I^2 (%)	Studies	Patients	Indirectness	Inconsistency	Imprecision	Bias	Publ. Bias	Quality
AFR	Chronic kidney disease	HR 1.51 (1.2-1.89)	0.0	1[121]	22,308	no	na	no	no	no	++++
AFR	Diabetes	HR 1.13 (0.83-1.55)	0.0	1[121]	22,308	no	na	yes	no	no	+++
AMR: North	Chronic kidney disease	HR range 0.88-1.61	71.9	4[170,205,209,226]	20,564	no	yes	yes	no	no	++
AMR: North	Chronic kidney disease	OR 1.39 (1.17-1.66)	19.7	11[169,173,183,188,193,207,210,214-216,224]	10,661	no	no	no	no	no*	++++
AMR: North	Chronic kidney disease	RR 1.33 (1.1-1.61)	0.0	1[192]	2,490	no	na	no	no	no	++++

Region	Condition	Estimate (95% CI)	I² (%)	Studies	Patients	Indirectness	Inconsistency	Imprecision	Bias	Publ. Bias	Quality
AMR: North	Chronic liver disease	HR 0.95 (0.46-1.95)	0.0	1[205]	5,776	no	na	yes	no	no	+++
AMR: North	Chronic liver disease	OR 1.62 (0.88-3)	0.0	3[185,215,216]	1,302	no	no	yes	no	no	+++
AMR: North	Chronic liver/Cirrhosis	HR 1.95 (0.62-6.17)	0.0	1[170]	841	no	na	yes	no	no	+++
AMR: North	Chronic liver/Cirrhosis	OR 5.96 (1.29-27.66)	29.1	2[185,214]	605	no	no	no	no	no	++++
AMR: North	Chronic liver/Non-cirrhotic	OR 1.47 (0.64-3.38)	0.0	1[185]	363	no	na	yes	no	no	+++
AMR: North	Diabetes	HR 1.05 (0.96-1.14)	0.0	5[170,200,205,209,226]	23,384	no	no	yes	no	no	+++
AMR: North	Diabetes	OR 1.33 (1.2-1.48)	0.0	12[169,173,188,193,197,207,210,214,224]	10,843	no	no	no	no	no**	++++
AMR: North	Diabetes	RR 1.18 (1.06-1.32)	0.0	3[192,204,221]	3,340	no	no	no	no	no	++++
AMR: North	Hepatitis	HR 0.61 (0.13-2.89)	0.0	1[170]	841	no	na	yes	no	no	+++
AMR: North	Dyslipidemia or hyperlipidemia	HR 0.95 (9.79-1.13)	0.0	1[209]	2,737	no	na	yes	no	no	+++
AMR: North	Dyslipidemia or hyperlipidemia	OR 1 (0.83-1.22)	0.0	3[185,193,210]	3,435	no	no	yes	no	no	+++
AMR: North	Dyslipidemia or hyperlipidemia	RR 0.75 (0.57-0.98)	0.0	1[221]	346	no	na	no	no	no	++++
AMR: South/Latin	Chronic kidney disease	HR range 1.19-1.36	74.6	2[228,234]	16,714	no	yes	no	no	no	+++
AMR: South/Latin	Chronic kidney disease	OR 1.68 (0.94-3.09)	0.0	1[235]	1,152	no	na	yes	no	no	+++
AMR: South/Latin	Chronic liver disease	HR 1.09 (0.83-1.44)	0.0	1[228]	11,321	no	na	yes	no	no	+++
AMR: South/Latin	Diabetes	HR range 1.09-1.18	73.7	2[228,234]	16,714	no	yes	no	no	no	+++
EMR	Diabetes	OR 1.64 (1.24-2.18)	0.0	1[123]	2,957	no	na	no	no	no	++++
EUR	Chronic kidney disease	HR range 0.97-5.09	94.5	9[131,134,138-141,147,157,160]	26,635	no	yes	no	no	no***	+++
EUR	Chronic kidney disease	OR 1.92 (1.57-2.36)	0.0	4[147,153,156,160]	4,914	no	no	no	no	no	++++
EUR	Chronic kidney disease	RR 1.13 (0.71-1.6)	0.0	1[136]	339	no	na	yes	no	no	+++
EUR	Chronic liver disease	HR 1.51 (1.21-1.88)	0.0	1[140]	15,194	no	na	no	no	no	++++
EUR	Chronic liver disease	OR 1.9 (0.9-3.7)	0.0	1[156]	2,254	no	na	yes	no	no	+++
EUR	Chronic liver/Cirrhosis	HR 2.03 (1.31-3.13)	0.0	1[127]	4,035	no	na	no	no	no	++++

Region	Condition	Estimate (95% CI)	I^2 (%)	Studies	Patients	Indirectness	Inconsistency	Imprecision	Bias	Publ. Bias	Quality
EUR	Chronic liver/Cirrhosis	OR 3.19 (0.95-10.76)	0.0	1[153]	614	no	na	yes	no	no	+++
EUR	Chronic liver/Non-cirrhotic	OR 0.99 (0.47-2.11)	0.0	1[153]	614	no	na	yes	no	no	+++
EUR	Diabetes	HR range 0.73-2.55	49.7	9[124,131,134,139-141,147,157,160]	26,814	no	yes	no	no	no	+++
EUR	Diabetes	OR 1.26 (1.02-1.56)	19.6	5[132,147,153,156,160]	5,006	no	no	no	no	no	++++
EUR	Diabetes	RR 1.23 (0.85-1.63)	0.0	1[136]	339	no	na	yes	no	no	+++
EUR	Dyslipidemia or hyperlipidemia	HR 1.03 (0.93-1.13)	0.0	1[131]	2,791	no	na	yes	no	no	+++
WPR	Chronic kidney disease	HR range 0.66-10.84	92.4	3[246,258,275]	4,293	no	yes	yes	no	no	++
WPR	Chronic kidney disease	OR 2.65 (1.45-4.84)	0.0	2[240,254]	5,974	no	no	no	no	no	++++
WPR	Chronic liver disease	HR 1.22 (0.46-3.21)	0.0	1[275]	1,000	no	na	yes	no	no	+++
WPR	Diabetes	HR 1.54 (1.29-1.85)	17.5	10[245,249,250,252,257,258,267,274,275,277]	11,628	no	no	no	no	no****	++++
WPR	Diabetes	OR range 0.71-5.45	84.8	4[242,254,255,269]	5,123	no	yes	yes	no	no	++
WPR	Hepatitis	OR 1.25 (0.44-3.54)	0.0	1[254]	2,665	no	na	yes	no	no	+++

AMR, regions of America; AFR, African region; HR, hazard ratio; EMR, Eastern Mediterranean region; EUR, European region; na, not applicable; OR, odds ratio; RR, risk ratio; WPR, Western Pacific region
* Beggs: tau=-0.3091,pval=0.2183; Egger: zval=-1.424,pval=0.1544
**Beggs: tau=-0.1212,pval=0.6384; Egger: zval=0.4612,pval=0.6446
*** Beggs: tau=0.3846,pval=0.0763; Egger: zval=1.1494,pval=0.2504
**** Beggs: tau=-0.0545,pval=0.8793; Egger: zval=0.4041,pval=0.6861

Table 6. 2 Risk of death (in-hospital mortality) in persons with respiratory diseases: meta-analysis and confidence in the estimate (GRADE)

Region	Condition	Estimate (95% CI)	I^2 (%)	Studies	Patients	Indirectness	Inconsistency	Imprecision	Bias	Publ. Bias	Quality
AFR	Respiratory disease	HR 0.68 (0.53-0.86)	0.0	1[121]	22,308	no	na	no	no	no	++++
AFR	Tuberculosis	HR 1.3 (1.05-1.63)	0.3	1[121]	22,308	no	na	no	no	no	++++
AMR: North	Asthma	HR range 0.91-1.35	79.1	2[205,226]	16,986	no	yes	yes	no	no	++
AMR: North	Asthma	OR 0.9 (0.71-1.13)	0.0	3[194,216,224]	8,782	no	no	yes	no	no	+++
AMR: North	COPD	HR 1.23 (1.03-1.47)	0.0	3[205,217,226]	17,479	no	no	no	no	no	++++
AMR: North	COPD	OR 1.46 (0.89-2.38)	37.9	5[169,195,207,216,224]	3,587	no	no	yes	no	no	+++
AMR: North	COPD	RR 1.41 (1.06-1.88)	0.0	1[221]	346	no	na	no	no	no	++++
AMR: North	COPD or asthma	HR 0.93 (0.76-1.15)	0.0	1[209]	2,737	no	na	yes	no	no	+++

Region	Condition	Estimate (95% CI)	I² (%)	Studies	Patients	Indirectness	Inconsistency	Imprecision	Bias	Publ. Bias	Quality
AMR: North	COPD or asthma	OR 0.8 (0.61-1.07)	0.0	3[188,214,215]	3,745	no	no	yes	no	no	+++
AMR: North	Interstitial lung disease	HR 2.17 (1.76-2.69)	0.0	1[205]	5,776	no	na	no	no	no	++++
AMR: North	Respiratory disease	HR 0.82 (0.55-1.2)	0.0	1[170]	841	no	na	yes	no	no	+++
AMR: North	Respiratory disease	OR 1.69 (1.04-2.74)	0.0	3[185,197,210]	980	no	no	no	no	no	++++
AMR: North	Respiratory disease	RR 1.31 (1.13-1.52)	0.0	1[192]	2,490	no	na	no	no	no	++++
AMR: South/Latin	Asthma	HR 0.93 (0.86-1)	0.0	2[228,234]	16,714	no	no	no	no	no	++++
AMR: South/Latin	COPD	HR 1.12 (1.07-1.18)	0.0	1[234]	5,393	no	na	no	no	no	++++
AMR: South/Latin	Respiratory disease	HR 1.21 (1.06-1.38)	0.0	1[228]	11,321	no	na	no	no	no	++++
EUR	Asthma	OR 0.42 (0.19-0.91)	0.0	1[153]	614	no	na	no	no	no	++++
EUR	COPD	HR 1.15 (1.03-1.29)	0.0	1[131]	2,791	no	na	no	no	no	++++
EUR	COPD	OR 0.78 (0.34-1.81)	0.0	1[153]	614	no	na	yes	no	no	+++
EUR	COPD	RR 1.45 (0.94-1.95)	0.0	1[136]	339	no	na	no	no	no	++++
EUR	Respiratory disease	HR 1.17 (1.09-1.26)	0.0	4[139-141,157]	20,043	no	no	no	no	no	++++
EUR	Respiratory disease	OR 1.9 (0.47-7.64)	30.7	2[229,132]	2,346	no	no	yes	no	no	+++
WPR	COPD	HR 1.74 (1.03-2.95)	0.0	3[258,264,275]	1,755	no	no	no	no	no	++++
WPR	COPD	OR 2.22 (1.55-3.19)	0.0	4 [240,242,254,273]	6,897	no	no	no	no	no	++++
WPR	Respiratory disease	HR range 2.51-13.66	61.2	2[249,257]	409	no	yes	yes	no	no	++
WPR	Respiratory disease	OR 3.24 (0.45-23.57)	0.0	1[259]	244	no	na	yes	no	no	+++
WPR	Tuberculosis	OR 1.55 (0.69-3.44)	0.0	1[254]	2,665	no	na	yes	no	no	+++
WPR	Tuberculosis	RR 2.25 (1.35-3.75)	0.0	1[260]	330	no	na	no	no	no	++++

AMR, regions of America; AFR, African region; HR, hazard ratio; EMR, Eastern Mediterranean region; EUR, European region; na, not applicable; OR, odds ratio; RR, risk ratio; WPR, Western Pacific region

Table 6. 3 Risk of death (in-hospital mortality) in persons with circulatory diseases: meta-analysis and confidence in the estimate (GRADE)

Region	Condition	Estimate (95% CI)	I² (%)	Studies	Patients	Indirectness	Inconsistency	Imprecision	Bias	Publ. Bias	Quality
AFR	Hypertension	HR 1.05 (0.88-1.27)	0.0	1[121]	22,308	no	na	yes	no	no	+++
AMR: North	Arrhythmia	OR 1.29 (1.02-1.62)	0.0	2[188,224]	5,101	no	no	no	no	no	++++

Region	Condition	Estimate (95% CI)	I^2 (%)	Studies	Patients	Indirectness	Inconsistency	Imprecision	Bias	Publ. Bias	Quality
AMR: North	Cardiovascular disease	HR 1.13 (0.96-1.33)	0.0	1[205]	5,776	no	na	yes	no	no	+++
AMR: North	Cardiovascular disease	OR 1.42 (0.92-2.17)	0.0	4[173,183,197,210]	1,552	no	no	yes	no	no	+++
AMR: North	Cardiovascular disease	RR 1.28 (1.03-1.58)	0.0	1[192]	2,490	no	na	no	no	no	++++
AMR: North	Coronary artery disease	HR 1.17 (1.07-1.28)	0.0	3[170,209,226]	14,788	no	no	no	no	no	++++
AMR: North	Coronary artery disease	OR 1.19 (1.03-1.38)	10.2	6[188,193,195,207,224]	9,335	no	no	no	no	no	++++
AMR: North	Heart disease	OR 0.98 (0.46-2.09)	0.0	1[185]	363	no	na	yes	no	no	+++
AMR: North	Heart failure	HR range 1.05-1.77	87.2	3[170,209,226]	14,788	no	yes	yes	no	no	++
AMR: North	Heart failure	OR 1.31 (1.08-1.6)	31.6	8[188,193,195,207,214,215,224]	9,994	no	no	no	no	no	++++
AMR: North	Hypertension	HR 0.88 (0.8-0.95)	0.0	4[170,200,205,209]	12,174	no	no	no	no	no	++++
AMR: North	Hypertension	OR range 0.3-17.02	68.6	12[169,173,185,188,193,195,197,210,214-216,224]	11,321	no	yes	yes	no	no*	++
AMR: North	Hypertension	RR range 0.81-1.07	54.5	2[192,204]	2,994	no	yes	yes	no	no	++
AMR: North	Peripheral vascular disease	OR 1.1 (0.72-1.68)	0.0	1[224]	2,015	no	na	yes	no	no	+++
AMR: North	V.thromboembolism	OR range 0.75-6	66.3	2[210,224]	2,253	no	yes	yes	no	no	++
AMR: South/Latin	Cardiovascular disease	HR 1.02 (0.95-1.1)	0.0	1[228]	11,321	no	na	yes	no	no	+++
AMR: South/Latin	Cardiovascular disease	OR 1.26 (0.95-1.67)	0.0	1[235]	1,152	no	na	yes	no	no	+++
AMR: South/Latin	Hypertension	HR 1.08 (1.05-1.11)	0.0	1[234]	5,393	no	na	no	no	no	++++
EMR	Cardiovascular disease	OR 1.1 (0.84-1.44)	0.0	1[123]	2,957	no	na	yes	no	no	+++
EUR	Arrhythmia	OR 1.37 (1.07-1.74)	0.0	2[153,156]	2,868	no	no	no	no	no	++++
EUR	Cardiovascular disease	HR 1.9 (1.19-3.01)	0.0	2[124,157]	608	no	no	no	no	no	++++
EUR	Cardiovascular disease	RR 0.84 (0.57-1.18)	0.0	1[136]	339	no	na	yes	no	no	+++
EUR	Coronary artery disease	HR range 1.19-2.93	83.3	4[134,137,141,147]	4,695	no	yes	no	no	no	+++
EUR	Coronary artery disease	OR range 1.0-1.51	55.8	3[147,153,156]	4,432	no	yes	yes	no	no	++
EUR	Heart disease	HR 1.2 (1.12-1.28)	33.8	2[131,140]	17,985	no	no	no	no	no	++++
EUR	Heart failure	HR range 1.05-3.1	80.9	2[138,139]	3,645	no	yes	yes	no	no	++

Region	Condition	Estimate (95% CI)	I² (%)	Studies	Patients	Indirectness	Inconsistency	Imprecision	Bias	Publ. Bias	Quality
EUR	Heart failure	OR 1.37 (1.02-1.84)	0.0	2[153,156]	2,868	no	no	no	no	no	++++
EUR	Hypertension	HR range 0.82-1.48	58.0	8[127,131,134,139,141,147,157,160]	15,285	no	yes	yes	no	no	++
EUR	Hypertension	OR 1.09 (0.88-1.34)	24.6	4[147,153,156,160]	4,914	no	no	yes	no	no	+++
EUR	Hypertension	RR 1.32 (0.91-1.81)	0.0	1[136]	339	no	na	yes	no	no	+++
EUR	Infarction	HR 1.21 (0.93-1.59)	0.0	1[139]	3,454	no	na	yes	no	no	+++
EUR	V.thromboembolism	OR 3.07 (0.65-14.4)	0.0	1[153]	614	no	na	yes	no	no	+++
WPR	Arrhythmia	HR 1.94 (0.24-3.62)	0.0	1[252]	596	no	na	yes	no	no	+++
WPR	Cardiovascular disease	HR 1.95 (1.46-2.61)	0.0	7[241,245,249,257,258,264,275]	4,091	no	no	no	no	no	++++
WPR	Cardiovascular disease	OR 1.4 (0.94-2.09)	0.0	2[240,262]	3,416	no	no	yes	no	no	+++
WPR	Coronary artery disease	HR range 0.972-4.28	63.7	3[241,252,275]	3,186	no	yes	yes	no	no	++
WPR	Coronary artery disease	OR 1.11 (0.72-1.72)	0.0	2[254,276]	2,836	no	no	yes	no	no	+++
WPR	Heart disease	OR 4.54 (1.47-14.01)	0.0	2[256,263]	2,825	no	no	no	no	no	++++
WPR	Heart failure	HR 3.3 (1.33-8.19)	0.0	1[246]	2,877	no	na	no	no	no	++++
WPR	Hypertension	HR 1.85 (1.48-2.3)	20.3	8[245,246,252,257,265,268,272,275]	8,794	no	no	no	no	no	++++
WPR	Hypertension	OR 1.11 (0.94-1.31)	0.0	9[239,240,242,248,254,259,262,263,269]	9,696	no	no	yes	no	no	+++
WPR	Infarction	HR 28.9 (10.64-78.51)	0.0	1[246]	2,877	no	na	no	no	no	++++

* Beggs: tau=0.2424,pval=0.3108; Egger: zval=1.9076,pval=0.0564
AMR, regions of America; AFR, African region; HR, hazard ratio; EMR, Eastern Mediterranean region; EUR, European region; na, not applicable; OR, odds ratio; RR, risk ratio; WPR, Western Pacific region

Table 6. 4 Risk of death (in-hospital mortality) in persons with overweight, obesity or underweight: meta-analysis and confidence in the estimate (GRADE)

Region	Condition	Estimate (95% CI)	I² (%)	Studies	Patients	Indirectness	Inconsistency	Imprecision	Bias	Publ. Bias	Quality
AMR: North	Obesity/BMI≥30	HR 1.02 (0.92-1.13)	0.0	4[180,205,209,226]	21,410	no	no	yes	no	no	+++
AMR: North	Obesity/BMI≥30	OR 1.14 (1.02-1.27)	0.0	11[169,185,188,193,197,207,210,211,215,216,224]	11,608	no	no	no	no	no*	++++
AMR: North	Obesity/BMI≥30	RR 1.16 (0.98-1.36)	15.9	2[192,204]	2,994	no	no	yes	no	no	+++
AMR: North	Obesity/BMI≥40	HR 1.41 (1.03-1.93)	0.0	2[180,209]	4,424	no	no	no	no	no	++++
AMR: North	Obesity/BMI≥40	OR 1.7 (1.26-2.29)	0.0	2[193,216]	3,356	no	no	no	no	no	++++
AMR: North	Overweight	HR 0.89 (0.77-1.02)	0.0	3[180,205,209]	10,200	no	no	yes	no	no	+++

Region	Condition	Estimate (95% CI)	I^2 (%)	Studies	Patients	Indirectness	Inconsistency	Imprecision	Bias	Publ. Bias	Quality
AMR: North	Overweight	OR 0.8 (0.62-1.03)	0.0	2[215,224]	2,432	no	no	yes	no	no	+++
AMR: North	Overweight	RR 1.4 (1.1-1.9)	0.0	1[204]	504	no	na	no	yes, NOS =4	no	+++
AMR: North	Underweight	HR range 1.15-2.37	87.2	2[180,205]	7,463	no	yes	yes	no	no	++
AMR: North	Underweight	OR 1.37 (0.52-3.64)	0.0	1[207]	200	no	na	yes	no	no	+++
AMR: South/Latin	Obesity/BMI≥30	HR range 1.08-1.29	79.5	2[228,234]	16,714	no	yes	yes	no	no	++
EUR	Obesity/BMI≥30	HR range 1.07-3.04	89.2	6[127,131,139,140,143,160]	26,189	no	yes	no	no	no	+++
EUR	Obesity/BMI≥30	OR range 1.2-12.1	75.5	3[126,156,160]	3,143	no	yes	no	no	no	+++

* Beggs: tau=0.4182,pval=0.0866; Egger: zval=2.1869,pval=0.0287
AMR, regions of America; AFR, African region; HR, hazard ratio; EMR, Eastern Mediterranean region; EUR, European region; na, not applicable; OR, odds ratio; RR, risk ratio; WPR, Western Pacific region

Table 6. 5 Risk of death (in-hospital mortality) in persons with immunodeficiency: meta-analysis and confidence in the estimate (GRADE)

Region	Condition	Estimate (95% CI)	I^2 (%)	Studies	Patients	Indirectness	Inconsistency	Imprecision	Bias	Publ. Bias	Quality
AFR	HIV	HR 1.45 (1.14-1.84)	0.0	1[121]	22,308	no	na	no	no	no	++++
AMR: North	Autoimmune condition	HR 1.21 (0.74-1.98)	0.0	1[205]	5,776	no	na	yes	no	no	+++
AMR: North	HIV	HR range 1.13-2.47	52.9	2[170,217]	1,334	no	yes	yes	no	no	++
AMR: North	HIV	OR 0.07 (0.03-0.52)	0.0	1[206]	251	no	na	no	no	no	++++
AMR: North	Inflammatory bowel disease	HR 1.18 (0.35-4.06)	0.0	1[170]	841	no	na	yes	no	no	+++
AMR: North	Immunosuppression	HR 1.57 (0.66-3.73)	0.0	1[170]	841	no	na	yes	no	no	+++
AMR: North	Immunosuppression	OR 3.6 (1.52-8.47)	0.0	1[216]	522	no	na	no	no	no	++++
AMR: North	Immunosuppression	RR 1.39 (1.13-1.7)	0.0	1[192]	2,490	no	na	no	no	no	++++
AMR: North	Organ transplant recipients	HR 0.99 (0.45-2.17)	0.0	1[170]	841	no	na	yes	no	no	+++
AMR: North	Rheumatological disease	HR 0.48 (0.17-1.34)	0.0	1[170]	841	no	na	yes	no	no	+++
AMR: North	Rheumatological disease	RR 0.87 (0.66-1.16)	0.0	1[192]	2,490	no	na	yes	no	no	+++

Region	Condition	Estimate (95% CI)	I^2 (%)	Studies	Patients	Indirectness	Inconsistency	Imprecision	Bias	Publ. Bias	Quality
AMR: South/Latin	Immunosuppression	HR 1.09 (1.03-1.16)	0.0	2[228,234]	16,714	no	no	no	no	no	++++
EUR	Autoimmune condition	HR 1.19 (1.06-1.33)	0.0	1[131]	2,791	no	na	no	no	no	++++
EUR	HIV	HR 1.5 (1.02-2.22)	0.0	1[142]	47,539	no	na	no	no	no	++++
EUR	HIV	OR 1.32 (0.24-7.36)	0.0	1[153]	614	no	na	yes	no	no	+++
EUR	Immunosuppression	OR 2.11 (1.08-4.09)	0.0	1[130]	302	no	na	no	no	no	++++
EUR	Organ transplant recipients	OR 4.2 (1.6-11.4)	0.0	1[156]	2,254	no	na	no	no	no	++++
EUR	Rheumatological disease	OR 1.2 (0.8-1.9)	0.0	1[156]	2,254	no	na	yes	no	no	+++

AMR, regions of America; AFR, African region; HR, hazard ratio; EMR, Eastern Mediterranean region; EUR, European region; na, not applicable; OR, odds ratio; RR, risk ratio; WPR, Western Pacific region

Table 6. 6 Risk of death (in-hospital mortality) in persons with neurological diseases or mental health disorders: meta-analysis and confidence in the estimate (GRADE)

Region	Condition	Estimate (95% CI)	I^2 (%)	Studies	Patients	Indirectness	Inconsistency	Imprecision	Bias	Publ. Bias	Quality
AMR: North	Cerebrovascular/Stroke	HR 1.3 (0.84-2.01)	0.0	1[170]	841	no	na	yes	no	no	+++
AMR: North	Cerebrovascular/Stroke	OR 1.06 (0.78-1.45)	0.0	3[195,210,224]	2,931	no	no	yes	no	no	+++
AMR: North	Dementia	OR 2.03 (1.46-2.83)	0.0	1[224]	2,015	no	na	no	no	no	++++
AMR: North	Neurological disease	RR 1.25 (1.04-1.5)	0.0	1[192]	2,490	no	na	no	no	no	++++
AMR: South/Latin	Neurological disease	HR 1.34 (1.16-1.54)	0.0	1[228]	11,321	no	na	no	no	no	++++
EUR	Cerebrovascular/Stroke	HR 2.12 (1.29-3.47)	0.0	1[160]	482	no	na	no	no	no	++++
EUR	Cerebrovascular/Stroke	OR range 0.89-3.41	79.8	3[153,156,160]	3,350	no	yes	yes	no	no	++
EUR	Dementia	HR range 1.26-1.64	75.1	3[127,131,140]	22,020	no	yes	no	no	no	+++
EUR	Dementia	OR range 1.32-15.81	49.7	4[129,132,153,156]	3,587	no	yes	no	no	no	+++
EUR	Neurological disease	HR 1.18 (1.08-1.28)	0.0	2[127,140]	19,229	no	no	no	no	no	++++
EUR	Psychiatric disorder	OR 2.9 (1.3-6.6)	0.0	1[156]	2,254	no	na	no	no	no	++++
WPR	Cerebrovascular/Stroke	HR 1.44 (0.89-2.35)	0.0	4[249,252,258,264]	1,454	no	no	yes	no	no	+++
WPR	Cerebrovascular/Stroke	OR range 1.25-4.257	61.1	3[239,240,254]	6,634	no	yes	no	no	no	+++
WPR	Dementia	HR 7.7 (1.5-39.61)	0.0	1[249]	103	no	na	no	no	no	++++

AMR, regions of America; AFR, African region; HR, hazard ratio; EMR, Eastern Mediterranean region; EUR, European region; na, not applicable; OR, odds ratio; RR, risk ratio; WPR, Western Pacific region

Table 6. 7 Risk of death (in-hospital mortality) in persons with oncological diseases: meta-analysis and confidence in the estimate (GRADE)

Region	Condition	Estimate (95% CI)	I^2 (%)	Studies	Patients	Indirectness	Inconsistency	Imprecision	Bias	Publ. Bias	Quality
AMR: North	Cancer	HR 1.19 (1.06-1.33)	0.0	4[200,205,209,226]	22,543	no	no	no	no	no	++++
AMR: North	Cancer	OR 1.02 (0.88-1.18)	0.0	5[188,193,210,216,224]	8,695	no	no	yes	no	no	+++
AMR: North	Cancer/Active	HR 1.01 (0.55-1.83)	0.0	1[170]	841	no	na	yes	no	no	+++
AMR: North	Cancer/Active	OR 1.57 (0.46-5.36)	0.0	1[215]	417	no	na	yes	no	no	+++
EUR	Cancer	HR 1.2 (1.11-1.3)	32.1	3[131,139,140]	21,439	no	no	no	no	no	++++
EUR	Cancer	OR 1.2 (0.9-1.6)	0.0	1[156]	2,254	no	na	yes	no	no	+++
EUR	Cancer/Active	HR 1.44 (1.16-1.79)	25.6	3[127,137,141]	5,602	no	no	no	no	no	++++
EUR	Cancer/Active	OR 4.68 (1.47-14.88)	0.0	1[126]	407	no	na	no	no	no	++++
EUR	Cancer/ Hematologic	HR 1.74 (1.28-2.37)	0.0	1[164]	1,183	no	na	no	no	no	++++
EUR	Cancer/Hematologic	OR range 1.24-6.65	64.8	2[153,162]	706	no	yes	yes	no	no	++
EUR	Cancer/Solid	OR 0.74 (0.4-1.35)	0.0	1[153]	614	no	na	yes	no	no	+++
WPR	Cancer	HR 1.37 (0.89-2.1)	8.9	4[252,253,258,275]	3,871	no	no	yes	no	no	+++
WPR	Cancer	OR 3.23 (2.33-4.48)	0.0	4[240,242,254,255]	6,967	no	no	no	no	no	++++

AMR, regions of America; AFR, African region; HR, hazard ratio; EMR, Eastern Mediterranean region; EUR, European region; na, not applicable; OR, odds ratio; RR, risk ratio; WPR, Western Pacific region

Table 7. 1 Risk of death (case mortality) in persons with liver and metabolic diseases: meta-analysis and confidence in the estimate (GRADE)

Region	Condition	Estimate (95% CI)	I^2 (%)	Studies	Patients	Indirectness	Inconsistency	Imprecision	Bias	Publ. Bias	Quality
AFR	Chronic kidney disease	HR 1.92 (1.51-2.45)	0.0	1[121]	22,308	no	na	no	no	no	++++
AFR	Diabetes	HR 2.02 (1.47-2.76)	0.0	1[121]	22,308	no	na	no	no	no	++++
AMR: North	Chronic kidney disease	HR 1.53 (1.32-1.78)	0.0	1[189]	5,902	no	na	no	no	no	++++
AMR: North	Chronic kidney disease	OR range 1.36-2.85	76.9	3[181,184,225]	39,857	no	yes	no	no	no	+++
AMR: North	Chronic kidney disease	RR 1.17 (0.83-1.65)	0.0	1[223]	6,916	no	na	yes	no	no	+++
AMR: North	Chronic liver disease	HR 1.16 (0.94-1.44)	0.0	1[189]	5,902	no	na	yes	no	no	+++
AMR: North	Chronic liver disease	OR range 0.9-2.62	71.3	2[181,184]	32,265	no	yes	yes	no	no	++
AMR: North	Chronic liver disease	RR 3 (1.5-6.0)	0.0	1[219]	500	no	na	no	no	no	++++

Region	Condition	Estimate (95% CI)	I^2 (%)	Studies	Patients	Indirectness	Inconsistency	Imprecision	Bias	Publ. Bias	Quality
AMR: North	Diabetes	HR 1.21 (1.06-1.4)	0.0	1[189]	5,902	no	na	no	no	no	++++
AMR: North	Diabetes	OR range 1.11-2.4	75.4	3[175,181,184]	32,869	no	yes	yes	no	no	++
AMR: North	Diabetes	RR 1.16 (0.89-1.52)	0.0	1[223]	6,916	no	na	yes	no	no	+++
AMR: North	Dyslipidemia or hyperlipidemia	RR 1.47 (1.02-2.11)	0.0	1[223]	6,916	no	na	no	no	no	++++
AMR: South/Latin	Chronic kidney disease	HR range 0.7-2.679	88.9	3[229,236,237]	61,200	no	yes	yes	no	no	++
AMR: South/Latin	Chronic kidney disease	OR 1.44 (1.01-2.06)	0.0	1[230]	10,544	no	na	no	no	no	++++
AMR: South/Latin	Diabetes	HR range 1.3-1.73	52.6	3[229,236,237]	61,200	no	yes	no	no	no	+++
AMR: South/Latin	Diabetes	OR 1.5 (1.13-1.98)	0.0	1[230]	10,544	no	na	no	no	no	++++
EUR	Chronic kidney disease	HR 1.31 (1.21-1.42)	0.0	2[131,159]	5,156	no	no	no	no	no	++++
EUR	Chronic kidney disease	OR 1.9 (1.4-2.6)	0.0	1[156]	11,122	no	na	no	no	no	++++
EUR	Chronic liver disease	OR 1.8 (1-3.3)	0.0	1[156]	11,122	no	na	yes	no	no	+++
EUR	Diabetes	HR 1.35 (1.18-1.54)	16.8	2[131,159]	5,156	no	no	no	no	no	++++
EUR	Diabetes	OR 1.6 (1.3-2)	0.0	1[156]	11,122	no	na	no	no	no	++++
EUR	Dyslipidemia or hyperlipidemia	HR range 1.01-1.4	48.6	2[131,159]	5,156	no	yes	yes	no	no	++
WPR	Chronic kidney disease	HR 1.67 (0.95-2.93)	0.0	2[247,251]	8,541	no	no	yes	no	no	+++
WPR	Diabetes	HR 1.44 (0.96-2.17)	0.0	1[251]	8,266	no	na	yes	no	no	+++

AMR, regions of America; AFR, African region; HR, hazard ratio; EMR, Eastern Mediterranean region; EUR, European region; na, not applicable; OR, odds ratio; RR, risk ratio; WPR, Western Pacific region

Table 7. 2 Risk of death (case mortality) in persons with respiratory diseases: meta-analysis and confidence in the estimate (GRADE)

Region	Condition	Estimate (95% CI)	I^2 (%)	Studies	Patients	Indirectness	Inconsistency	Imprecision	Bias	Publ. Bias	Quality
AFR	Respiratory disease	HR 0.92 (0.72-1.18)	0.0	1[121]	22,308	no	na	yes	no	no	+++
AFR	Tuberculosis	HR 1.57 (1.25-1.97)	0.0	1[121]	22,308	no	na	no	no	no	++++
AMR: North	Asthma	OR 0.63 (0.38-1.04)	0.0	1[225]	7,592	no	na	yes	no	no	+++
AMR: North	Asthma	RR 0.81 (0.54-1.21)	0.0	1[223]	6,916	no	na	yes	no	no	+++

Region	Condition	Estimate (95% CI)	I^2 (%)	Studies	Patients	Indirectness	Inconsistency	Imprecision	Bias	Publ. Bias	Quality
AMR: North	Respiratory disease	HR 1.01 (0.87-1.17)	0.0	1[189]	5,902	no	na	yes	no	no	+++
AMR: North	Respiratory disease	OR 1.24 (1.08-1.42)	0.0	2[181,184]	32,265	no	no	no	no	no	++++
AMR: North	Respiratory disease	RR 0.93 (0.6-1.42)	0.0	1[223]	6,916	no	na	yes	no	no	+++
AMR: South/Latin	Asthma	HR 0.78 (0.47-1.28)	0.0	1[236]	7,497	no	na	yes	no	no	+++
AMR: South/Latin	COPD	HR 1.49 (1.22-1.84)	39.8	2[229,236]	59,130	no	no	no	no	no	++++
AMR: South/Latin	COPD	OR 1.68 (1.22-2.31)	0.0	1[230]	10,544	no	na	no	no	no	++++
AMR: South/Latin	Respiratory disease	HR range 2.6-5.21	80.0	2[229,237]	53,703	no	yes	no	no	no	+++
EUR	COPD	HR 1.08 (0.96-1.22)	0.0	2[131,159]	5,156	no	no	yes	no	no	+++
EUR	Respiratory disease	OR 1.4 (1.1-1.8)	0.0	1[156]	11,122	no	na	no	no	no	++++
WPR	COPD	HR 1.43 (1.01-2.03)	0.0	2[247,251]	8,541	no	no	no	no	no	++++
WPR	Tuberculosis	RR 2.17 (1.4-3.37)	0.0	1[260]	530	no	na	no	no	no	++++

AMR, regions of America; AFR, African region; HR, hazard ratio; EMR, Eastern Mediterranean region; EUR, European region; na, not applicable; OR, odds ratio; RR, risk ratio; WPR, Western Pacific region

Table 7. 3 Risk of death (case mortality) in persons with circulatory diseases: meta-analysis and confidence in the estimate (GRADE)

Region	Condition	Estimate (95% CI)	I^2 (%)	Studies	Patients	Indirectness	Inconsistency	Imprecision	Bias	Publ. Bias	Quality
AFR	Hypertension	HR 1.02 (0.84-1.24)	0.0	1[121]	22,308	no	na	yes	no	no	+++
AMR: North	Cardiovascular disease	HR 1.16 (1-1.35)	0.0	1[189]	5,902	no	na	yes	no	no	+++
AMR: North	Cardiovascular disease	OR 1.52 (0.53-4.38)	0.0	1[181]	804	no	na	yes	no	no	+++
AMR: North	Heart failure	OR 1.42 (1.21-1.67)	0.0	1[184]	31,461	no	na	no	no	no	++++
AMR: North	Heart failure	RR 0.84 (0.56-1.27)	0.0	1[223]	6,916	no	na	yes	no	no	+++
AMR: North	Hypertension	HR 1 (0.87-1.16)	0.0	1[189]	5,902	no	na	yes	no	no	+++
AMR: North	Hypertension	OR 1 (0.42-2.38)	0.0	1[175]	604	no	na	yes	no	no	+++
AMR: North	Hypertension	RR 1.38 (0.93-2.06)	0.0	1[223]	6,916	no	na	yes	no	no	+++

Region	Condition	Estimate (95% CI)	I² (%)	Studies	Patients	Indirectness	Inconsistency	Imprecision	Bias	Publ. Bias	Quality
AMR: North	Infarction	OR 1.97 (1.64-2.35)	0.0	1[184]	31,461	no	na	no	no	no	++++
AMR: North	Infarction	RR 1.66 (1.04-2.64)	0.0	1[223]	6,916	no	na	no	no	no	++++
AMR: North	Peripheral vascular disease	OR 0.89 (0.74-1.07)	0.0	1[184]	31,461	no	na	yes	no	no	+++
AMR: North	Peripheral vascular disease	RR 1.31 (0.89-1.91)	0.0	1[223]	6,916	no	na	yes	no	no	+++
AMR: South/Latin	Cardiovascular disease	HR range 0.872-8.9	98.2	2[236,237]	9,567	no	yes	yes	no	no	++
AMR: South/Latin	Hypertension	HR 1.38 (1.09-1.75)	0.0	1[236]	7,497	no	na	no	no	no	++++
AMR: South/Latin	Hypertension	OR range 1.49-3.284	70.7	2[230,232]	10,651	no	yes	yes	no	no	++
EUR	Arrhythmia	HR 1.8 (1.3-2.5)	0.0	1[159]	2,362	no	na	no	no	no	++++
EUR	Arrhythmia	OR 1.6 (1.2-2)	0.0	1[156]	11,122	no	na	no	no	no	++++
EUR	Cardiovascular disease	HR 1.2 (0.6-2.2)	0.0	1[159]	2,362	no	na	yes	no	no	+++
EUR	Coronary artery disease	HR 1.7 (1.2-2.5)	0.0	1[159]	2,362	no	na	no	no	no	++++
EUR	Coronary artery disease	OR 1.1 (0.9-1.4)	0.0	1[229]	11,122	no	na	yes	no	no	+++
EUR	Heart disease	HR 1.03 (0.95-1.11)	0.0	1[131]	2,794	no	na	yes	no	no	+++
EUR	Heart failure	HR 2.3 (1.6-3.2)	0.0	1[159]	2,362	no	na	no	no	no	++++
EUR	Heart failure	OR 1.8 (1.3-2.4)	0.0	1[156]	11,122	no	na	no	no	no	++++
EUR	Hypertension	HR range 0.95-1.6	91.9	2[131,159]	5,156	no	yes	yes	no	no	++
EUR	Hypertension	OR 1.3 (1.1-1.6)	0.0	1[156]	11,122	no	na	no	no	no	++++
WPR	Coronary artery disease	HR range 1.129-3.01	87.7	2[247,251]	8,541	no	yes	yes	no	no	++
WPR	Hypertension	HR 1.25 (0.73-2.14)	0.0	1[251]	8,266	no	na	yes	no	no	+++

AMR, regions of America; AFR, African region; HR, hazard ratio; EMR, Eastern Mediterranean region; EUR, European region; na, not applicable; OR, odds ratio; RR, risk ratio; WPR, Western Pacific region

Table 7. 4 Risk of death (case mortality) in persons with overweight, obesity or underweight: meta-analysis and confidence in the estimate (GRADE)

Region	Condition	Estimate (95% CI)	I² (%)	Studies	Patients	Indirectness	Inconsistency	Imprecision	Bias	Publ. Bias	Quality
AMR: North	Obesity/BMI≥30	HR 1.42 (1.19-1.7)	0.0	1[189]	5,902	no	na	no	no	no	++++
AMR: North	Obesity/BMI≥30	OR 1.43 (0.82-2.48)	5.6	2[175,181]	1,408	no	no	yes	no	no	+++

Region	Condition	Estimate (95% CI)	I² (%)	Studies	Patients	Indirectness	Inconsistency	Imprecision	Bias	Publ. Bias	Quality
AMR: North	Obesity/BMI≥30	RR 1.15 (0.92-1.44)	0.0	2[218,223]	11,834	no	no	yes	no	no	+++
AMR: North	Obesity/BMI≥40	RR 3.29 (2.07-5.22)	0.0	1[223]	6,916	no	na	no	no	no	++++
AMR: North	Overweight	OR 0.91 (0.3-2.79)	0.0	1[181]	804	no	na	yes	no	no	+++
AMR: North	Overweight	RR 0.91 (0.62-1.35)	0.0	1[223]	6,916	no	na	yes	no	no	+++
AMR: North	Underweight	HR 0.97 (0.66-1.41)	0.0	1[189]	5,902	no	na	yes	no	no	+++
AMR: North	Underweight	OR 3.74 (0.21-66.6)	0.0	1[181]	804	no	na	yes	no	no	+++
AMR: North	Underweight	RR 1.81 (0.99-3.3)	0.0	1[223]	6,916	no	na	yes	no	no	+++
AMR: South/Latin	Obesity/BMI≥30	HR range 1.25-1.777	77.0	3[229,236,237]	61,200	no	yes	no	no	no	+++
AMR: South/Latin	Obesity/BMI≥30	OR 1.74 (1.35-2.26)	0.0	1[230]	10,544	no	na	no	no	no	++++
EUR	Obesity/BMI≥30	HR 0.98 (0.9-1.07)	0.0	2[131,159]	5,156	no	no	yes	no	no	+++
EUR	Obesity/BMI≥30	OR 1.5 (1.1-2)	0.0	1[156]	11,122	no	na	no	no	no	++++

AMR, regions of America; AFR, African region; HR, hazard ratio; EMR, Eastern Mediterranean region; EUR, European region; na, not applicable; OR, odds ratio; RR, risk ratio; WPR, Western Pacific region

Table 7. 5 Risk of death (case mortality) in persons with immunodeficiency: meta-analysis and confidence in the estimate (GRADE)

Region	Condition	Estimate (95% CI)	I² (%)	Studies	Patients	Indirectness	Inconsistency	Imprecision	Bias	Publ. Bias	Quality
AFR	HIV	HR 1.7 (1.32-2.18)	0.0	1[121]	22,308	no	na	no	no	no	++++
AMR: North	Autoimmune condition	OR 0.84 (0.38-1.86)	0.0	1[181]	804	no	na	yes	no	no	+++
AMR: North	HIV	HR 0.93 (0.56-1.54)	0.0	1[189]	5,902	no	na	yes	no	no	+++
AMR: North	HIV	OR 1.71 (1-2.93)	0.0	1[184]	31,461	no	na	yes	no	no	+++
AMR: North	Immunosuppression	RR 1.44 (0.92-2.25)	0.0	1[223]	6,916	no	na	yes	no	no	+++
AMR: North	Organ transplant recipients	RR 6.54 (2.66-16.12)	0.0	1[223]	6,916	no	na	no	no	no	++++
AMR: North	Rheumatological disease	OR 1.18 (0.87-1.61)	0.0	2[177,184]	31,617	no	no	yes	no	no	+++
AMR: South/Latin	Immunosuppression	HR range 1.27-1.696	43.4	2[229,236]	59,130	no	yes	no	no	no	+++

Region	Condition	Estimate (95% CI)	I^2 (%)	Studies	Patients	Indirectness	Inconsistency	Imprecision	Bias	Publ. Bias	Quality
AMR: South/Latin	Immunosuppression	OR 1.7 (1.13-2.55)	0.0	1[230]	10,544	no	na	no	no	no	++++
EUR	Autoimmune condition	HR 1.1 (0.97-1.24)	0.0	1[131]	2,794	no	na	yes	no	no	+++
EUR	Organ transplant recipients	OR 3.2 (1.3-8.4)	0.0	1[156]	11,122	no	na	no	no	no	++++
EUR	Rheumatological disease	OR 1 (0.8-1.6)	0.0	1[156]	11,122	no	na	yes	no	no	+++

AMR, regions of America; AFR, African region; HR, hazard ratio; EMR, Eastern Mediterranean region; EUR, European region; na, not applicable; OR, odds ratio; RR, risk ratio; WPR, Western Pacific region

Table 7. 6 Risk of death (case mortality) in persons with neurological diseases or mental health disorders: meta-analysis and confidence in the estimate (GRADE)

Region	Condition	Estimate (95% CI)	I2	Studies	Patients	Indirectness	Inconsistency	Imprecision	Bias	Publ. Bias	Quality
AMR: North	Cerebrovascular/Stroke	OR 1.07 (0.9-1.28)	0.0	1[184]	31,461	no	na	yes	no	no	+++
AMR: North	Cerebrovascular/Stroke	RR 1.37 (0.93-2.03)	0.0	1[223]	6,916	no	na	yes	no	no	+++
AMR: North	Dementia	HR 1.47 (1.21-1.78)	0.0	1[189]	5,902	no	na	no	no	no	++++
AMR: North	Dementia	OR 1.29 (1.07-1.56)	0.0	1[184]	31,461	no	na	no	no	no	++++
AMR: South/Latin	Neurological disease	HR 3.9 (1.9-7.8)	0.0	1[237]	2,070	no	na	no	no	no	++++
EUR	Cerebrovascular/Stroke	OR 1.4 (1.1-1.8)	0.0	1[156]	11,122	no	na	no	no	no	++++
EUR	Dementia	HR 1.92 (1.77-2.08)	0.0	2[131,159]	5,156	no	no	no	no	no	++++
EUR	Dementia	OR 2 (1.5-2.6)	0.0	1[156]	11,122	no	na	no	no	no	++++
EUR	Psychiatric disorder	OR 2.5 (1.2-5.1)	0.0	1[156]	11,122	no	na	no	no	no	++++
WPR	Cerebrovascular/Stroke	HR 1.38 (0.82-2.3)	31.9	2[247,251]	8,541	no	no	yes	no	no	+++

AMR, regions of America; AFR, African region; HR, hazard ratio; EMR, Eastern Mediterranean region; EUR, European region; na, not applicable; OR, odds ratio; RR, risk ratio; WPR, Western Pacific region

Table 7. 7 Risk of death (case mortality) in persons with oncological diseases: meta-analysis and confidence in the estimate (GRADE)

Region	Condition	Estimate (95% CI)	I2	Studies	Patients	Indirectness	Inconsistency	Imprecision	Bias	Publ. Bias	Quality
AMR: North	Cancer	HR 0.93 (0.75-1.14)	0.0	1[189]	5,902	no	na	yes	no	no	+++
AMR: North	Cancer	OR range 0.87-1.59	66.7	2[181,184]	32,265	no	yes	yes	no	no	++

Region	Condition	Estimate (95% CI)	I2	Studies	Patients	Indirectness	Inconsistency	Imprecision	Bias	Publ. Bias	Quality
AMR: North	Cancer	RR 1.06 (0.64-1.75)	0.0	1[223]	6,916	no	na	yes	no	no	+++
AMR: North	Cancer/Solid	OR 1.7 (1.19-2.43)	0.0	1[184]	31,461	no	na	no	no	no	++++
EUR	Cancer	HR 1.25 (1.15-1.36)	0.0	2[131,159]	5,156	no	no	no	no	no	++++
EUR	Cancer	OR 1.3 (1-1.7)	0.0	1[156]	11,122	no	na	yes	no	no	+++
WPR	Cancer	HR 1.02 (0.57-1.81)	0.0	1[251]	8,266	no	na	yes	no	no	+++

AMR, regions of America; AFR, African region; HR, hazard ratio; EMR, Eastern Mediterranean region; EUR, European region; na, not applicable; OR, odds ratio; RR, risk ratio; WPR, Western Pacific region

Table 8. 1 Risk of ICU admission in persons with liver and metabolic diseases: meta-analysis and confidence in the estimate (GRADE)

Region	Condition	Estimate (95% CI)	I^2 (%)	Studies	Patients	Indirectness	Inconsistency	Imprecision	Bias	Publ. Bias	Quality
AMR: North	Chronic kidney disease	OR range 0.89-2	76.9	2[215,222]	899	no	yes	yes	no	no	++
AMR: North	Chronic kidney disease	RR 1.05 (0.94-1.16)	0.0	1[192]	2,490	no	na	yes	no	no	+++
AMR: North	Chronic liver disease	OR 1.49 (0.97-2.28)	2.0	2[185,215]	799	no	no	yes	no	no	+++
AMR: North	Diabetes	OR 1.4 (1.12-1.75)	0.0	6[178,185,190,197,215,222]	1,958	no	no	no	no	no	++++
AMR: North	Diabetes	RR 1.13 (1.03-1.24)	0.0	1[192]	2,490	no	na	no	no	no	++++
AMR: North	Dyslipidemia or hyperlipidemia	OR 1.01 (0.63-1.63)	0.0	1[185]	363	no	na	yes	no	no	+++
AMR: South/Latin	Diabetes	OR 1.87 (1.41-4.26)	0.0	1[231]	3,844	no	na	no	no	no	++++
EMR	Diabetes	OR 5.49 (3.13-9.65)	0.0	1[122]	1,158	no	na	no	no	no	++++
EUR	Chronic kidney disease	HR range 0.9-2.83	86.1	2[141,160]	1,639	no	yes	yes	no	no	++
EUR	Chronic kidney disease	OR 4.8 (1.83-12.6)	0.0	1[160]	482	no	na	no	no	no	++++
EUR	Diabetes	HR 1.45 (1.1-1.91)	0.0	2[141,160]	1,639	no	no	no	no	no	++++
EUR	Diabetes	OR 1.83 (1.12-2.98)	0.0	2[124,160]	808	no	no	no	no	no	++++
WPR	Diabetes	HR 4.7 (1.6-14.1)	0.0	1[238]	420	no	na	no	no	no	++++
WPR	Diabetes	OR 1.45 (1.81-4.7)	0.0	1[242]	641	no	na	yes	no	no	+++

AMR, regions of America; AFR, African region; HR, hazard ratio; EMR, Eastern Mediterranean region; EUR, European region; na, not applicable; OR, odds ratio; RR, risk ratio; WPR, Western Pacific region

Table 8. 2 Risk of ICU admission in persons with respiratory diseases: meta-analysis and confidence in the estimate (GRADE)

Region	Condition	Estimate (95% CI)	I^2 (%)	Studies	Patients	Indirectness	Inconsistency	Imprecision	Bias	Publ. Bias	Quality
AMR: North	COPD or asthma	OR 0.71 (0.47-1.09)	0	2[178,215]	650	no	no	yes	no	no	+++
AMR: North	Respiratory disease	OR 0.94 (0.64-1.38)	0	3[185,190,197]	845	no	no	yes	no	no	+++
AMR: North	Respiratory disease	RR 1.17 (1-1.37)	0	1[192]	2,490	no	na	yes	no	no	+++
EUR	Respiratory disease	HR 1.26 (0.86-1.85)	0	1[141]	1,157	no	na	yes	no	no	+++
WPR	COPD	OR 3.27 (1.09-7.63)	0	1[242]	641	no	na	no	no	no	++++
WPR	Respiratory disease	HR 6.2 (1.9-20.1)	0	1[238]	420	no	na	no	no	no	++++

AMR, regions of America; AFR, African region; HR, hazard ratio; EMR, Eastern Mediterranean region; EUR, European region; na, not applicable; OR, odds ratio; RR, risk ratio; WPR, Western Pacific region

Table 8. 3 Risk of ICU admission in persons with circulatory diseases: meta-analysis and confidence in the estimate (GRADE)

Region	Condition	Estimate (95% CI)	I^2 (%)	Studies	Patients	Indirectness	Inconsistency	Imprecision	Bias	Publ. Bias	Quality
AMR: North	Cardiovascular disease	OR 0.7 (0.38-1.3)	0	1[197]	379	no	na	yes	no	no	+++
AMR: North	Cardiovascular disease	RR 0.98 (0.88-1.09)	0	1[192]	2,490	no	na	yes	no	no	+++
AMR: North	Coronary artery disease	OR 1.1 (0.6-2)	0	1[222]	463	no	na	yes	no	no	+++
AMR: North	Heart disease	OR 0.99 (0.59-1.67)	0	2[185,190]	466	no	no	yes	no	no	+++
AMR: North	Heart failure	OR 1.32 (0.78-2.23)	0	2[178,215]	650	no	no	yes	no	no	+++
AMR: North	Hypertension	OR 0.96 (0.75-1.22)	0	6[178,185,190,197,215,222]	1,958	no	no	yes	no	no	+++
AMR: North	Hypertension	RR 0.92 (0.79-1.07)	0	1[192]	2,490	no	na	yes	no	no	+++
AMR: South/Latin	Hypertension	OR 1.77 (1.37-2.29)	0	1[231]	3,844	no	na	no	no	no	++++
EMR	Hypertension	OR 0.63 (0.32-1.26)	0	1[122]	1,158	no	na	yes	no	no	+++
EUR	Coronary artery disease	HR 0.88 (0.51-1.52)	0	1[141]	1,157	no	na	yes	no	no	+++
EUR	Hypertension	HR 1.37 (1.02-1.83)	0	2[141,160]	1,639	no	no	no	no	no	++++
EUR	Hypertension	OR 1.61 (0.8-3.25)	0	1[160]	482	no	na	yes	no	no	+++
WPR	Hypertension	HR 4.6 (1.8-11.6)	0	1[238]	420	no	na	no	no	no	++++
WPR	Hypertension	OR 1.47 (1.27-4.22)	0	1[242]	641	no	na	yes	no	no	+++

AMR, regions of America; AFR, African region; HR, hazard ratio; EMR, Eastern Mediterranean region; EUR, European region; na, not applicable; OR, odds ratio; RR, risk ratio; WPR, Western Pacific region

Table 8. 4 Risk of ICU admission in persons with overweight, obesity or underweight: meta-analysis and confidence in the estimate (GRADE)

Region	Condition	Estimate (95% CI)	I^2 (%)	Studies	Patients	Indirectness	Inconsistency	Imprecision	Bias	Publ. Bias	Quality
AMR: North	Obesity/BMI≥30	OR 1.46 (1.06-2.02)	27.6	5[178,185,190,197,215]	1,495	no	no	no	no	no	++++
AMR: North	Obesity/BMI≥30	RR 1.31 (1.16-1.47)	0.0	1[192]	2,490	no	na	no	no	no	++++
AMR: North	Obesity/BMI≥40	OR 2 (1.4-3.6)	0.0	1[222]	463	no	na	no	no	no	++++
AMR: North	Overweight	OR 1.53 (0.89-2.65)	0.0	2[190,215]	539	no	no	yes	no	no	+++
AMR: South/Latin	Obesity/BMI≥30	OR 1.43 (1.11-1.83)	0.0	1[231]	3,844	no	na	no	no	no	++++
EMR	Obesity/BMI≥30	OR 2.27 (1.15-4.47)	0.0	1[122]	1,158	no	na	no	no	no	++++
EMR	Obesity/BMI≥40	OR 3.95 (1-15.2)	0.0	1[122]	1,158	no	na	no	no	no	++++
EMR	Overweight	OR 1.91 (0.94-3.84)	0.0	1[122]	1,158	no	na	yes	no	no	+++
EUR	Obesity/BMI≥30	HR 4.22 (3.02-5.91)	0.0	1[160]	482	no	na	no	no	no	++++
EUR	Obesity/BMI≥30	OR range 2.16-6.58	56.2	2[135,160]	822	no	yes	no	no	no	+++

AMR, regions of America; AFR, African region; HR, hazard ratio; EMR, Eastern Mediterranean region; EUR, European region; na, not applicable; OR, odds ratio; RR, risk ratio; WPR, Western Pacific region

Table 8. 5 Risk of ICU admission in persons with immunodeficiency: meta-analysis and confidence in the estimate (GRADE)

Region	Condition	Estimate (95% CI)	I^2 (%)	Studies	Patients	Indirectness	Inconsistency	Imprecision	Bias	Publ. Bias	Quality
AMR: North	Immunosuppression	RR 1.29 (1.13-1.47)	0	1[192]	2,490	no	na	no	no	no	++++
EUR	HIV	OR 1.22 (0.8-1.87)	0	1[142]	47,539	no	na	yes	no	no	+++

AMR, regions of America; EUR, European region; na, not applicable; OR, odds ratio; RR, risk ratio; WPR, Western Pacific region

Table 8. 6 Risk of ICU admission in persons with neurological diseases or mental health disorders: meta-analysis and confidence in the estimate (GRADE)

Region	Condition	Estimate (95% CI)	I^2 (%)	Studies	Patients	Indirectness	Inconsistency	Imprecision	Bias	Publ. Bias	Quality
AMR: North	Neurological disease	RR 0.85 (0.7-1.04)	0	1[192]	2,490	no	na	yes	no	no	+++
EUR	Cerebrovascular/Stroke	HR 0.54 (0.19-1.56)	0	1[160]	482	no	na	yes	no	no	+++

Region	Condition	Estimate (95% CI)	I^2 (%)	Studies	Patients	Indirectness	Inconsistency	Imprecision	Bias	Publ. Bias	Quality
EUR	Cerebrovascular/Stroke	OR 0.52 (0.13-2.05)	0	1[160]	482	no	na	yes	no	no	+++
WPR	Cerebrovascular/Stroke	HR 21.7 (1.6-300.5)	0	1[238]	420	no	na	no	no	no	++++

AMR, regions of America; AFR, African region; HR, hazard ratio; EMR, Eastern Mediterranean region; EUR, European region; na, not applicable; OR, odds ratio; RR, risk ratio; WPR, Western Pacific region

Table 8. 7 Risk of ICU admission in persons with oncological diseases: meta-analysis and confidence in the estimate (GRADE)

Region	Condition	Estimate (95% CI)	I^2 (%)	Studies	Patients	Indirectness	Inconsistency	Imprecision	Bias	Publ. Bias	Quality
AMR: North	Cancer	OR 1.9 (1-3.9)	0	1[222]	463	no	na	yes	no	no	+++
AMR: North	Cancer/Active	OR 0.6 (0.18-1.94)	0	1[215]	436	no	na	yes	no	no	+++
EUR	Cancer/Active	HR 0.81 (0.42-1.56)	0	1[141]	1,157	no	na	yes	no	no	+++
WPR	Cancer	OR 3.13 (0.38-5.88)	0	1[242]	641	no	na	yes	no	no	+++

AMR, regions of America; AFR, African region; HR, hazard ratio; EMR, Eastern Mediterranean region; EUR, European region; na, not applicable; OR, odds ratio; RR, risk ratio; WPR, Western Pacific region

Table 9. 1 Risk of intubation in persons with liver and metabolic diseases: meta-analysis and confidence in the estimate (GRADE)

Region	Condition	Estimate (95% CI)	I^2 (%)	Studies	Patients	Indirectness	Inconsistency	Imprecision	Bias	Publ. Bias	Quality
AMR: North	Chronic kidney disease	HR 0.7 (0.44-1.1)	0.0	1[170]	841	no	na	yes	no	no	+++
AMR: North	Chronic kidney disease	OR range 0.2-2.4	72.5	5[174,214,215,222,224]	3,268	no	yes	yes	no	no	++
AMR: North	Chronic liver disease	OR range 1.1-2.08	48.0	2[185,215]	799	no	yes	yes	no	no	++
AMR: North	Chronic liver/Cirrhosis	HR 1.16 (0.39-3.5)	0.0	1[170]	841	no	na	yes	no	no	+++
AMR: North	Chronic liver/Cirrhosis	OR 2.66 (0.4-17.95)	0.0	1[214]	242	no	na	yes	no	no	+++
AMR: North	Diabetes	HR 1.12 (0.84-1.49)	0.0	1[170]	841	no	na	yes	no	no	+++
AMR: North	Diabetes	OR 1.44 (1.25-1.67)	0.0	11[174,178,185,186,190,197,207,214,215,222,224]	5,013	no	no	no	no	no*	++++
AMR: North	Diabetes	RR 1.2 (0.87-1.7)	0.0	1[204]	504	no	na	yes	yes, NOS =4	no	++
AMR: North	Hepatitis	HR 0.94 (0.35-2.54)	0.0	1[170]	841	no	na	yes	no	no	+++

Region	Condition	Estimate (95% CI)	I² (%)	Studies	Patients	Indirectness	Inconsistency	Imprecision	Bias	Publ. Bias	Quality
AMR: North	Dyslipidemia or hyperlipidemia	OR 1.23 (0.81-1.85)	0.0	2[185,207]	563	no	no	yes	no	no	+++
EUR	Diabetes	OR 1.6 (0.44-5.83)	0.0	1[165]	124	no	na	yes	no	no	+++
EUR	Dyslipidemia or hyperlipidemia	OR 0.68 (0.24-1.97)	0.0	1[165]	124	no	na	yes	no	no	+++
WPR	Diabetes	OR 2.19 (1.76-6.14)	0.0	1[242]	641	no	na	no	no	no	++++

*Beggs: tau=0.0545,pval=0.8793; Egger: zval=0.4946,pval=0.6209
AMR, regions of America; AFR, African region; HR, hazard ratio; EMR, Eastern Mediterranean region; EUR, European region; na, not applicable; OR, odds ratio; RR, risk ratio; WPR, Western Pacific region

Table 9. 2 Risk of intubation in persons with respiratory diseases: meta-analysis and confidence in the estimate (GRADE)

Region	Condition	Estimate (95% CI)	I² (%)	Studies	Patients	Indirectness	Inconsistency	Imprecision	Bias	Publ. Bias	Quality
AMR: North	Asthma	OR 1.13 (0.82-1.55)	0	1[224]	2,015	no	na	yes	no	no	+++
AMR: North	COPD	OR 1.13 (0.75-1.7)	0	1[224]	2,015	no	na	yes	no	no	+++
AMR: North	COPD or asthma	OR 0.67 (0.44-1.02)	0	3[178,214,215]	892	no	no	yes	no	no	+++
AMR: North	Obstructive sleep apnea	OR 1.15 (0.4-3.35)	0	1[207]	200	no	na	yes	no	no	+++
AMR: North	Respiratory disease	HR 1.07 (0.77-1.48)	0	1[170]	841	no	na	yes	no	no	+++
AMR: North	Respiratory disease	OR 1.04 (0.7-1.54)	0	3[185,190,197]	845	no	no	yes	no	no	+++
EUR	COPD	OR 2.52 (0.35-17.81)	0	1[155]	145	no	na	yes	no	no	+++

AMR, regions of America; AFR, African region; HR, hazard ratio; EMR, Eastern Mediterranean region; EUR, European region; na, not applicable; OR, odds ratio; RR, risk ratio; WPR, Western Pacific region

Table 9. 3 Risk of intubation in persons with circulatory diseases: meta-analysis and confidence in the estimate (GRADE)

Region	Condition	Estimate (95% CI)	I² (%)	Studies	Patients	Indirectness	Inconsistency	Imprecision	Bias	Publ. Bias	Quality
AMR: North	Arrhythmia	OR 1.06 (0.75-1.48)	0.0	1[224]	2,015	no	na	yes	no	no	+++
AMR: North	Cardiovascular disease	OR 0.54 (0.28-1.04)	0.0	1[197]	379	no	na	yes	no	no	+++
AMR: North	Coronary artery disease	HR 0.86 (0.56-1.33)	0.0	1[170]	841	no	na	yes	no	no	+++
AMR: North	Coronary artery disease	OR 0.89 (0.61-1.28)	18.1	3[174,222,224]	2,590	no	no	yes	no	no	+++
AMR: North	Heart disease	OR range 0.64-3.41	83.5	2[185,190]	466	no	yes	yes	no	no	++

Region	Condition	Estimate (95% CI)	I^2 (%)	Studies	Patients	Indirectness	Inconsistency	Imprecision	Bias	Publ. Bias	Quality
AMR: North	Heart failure	HR 0.81 (0.59-1.11)	0.0	1[170]	841	no	na	yes	no	no	+++
AMR: North	Heart failure	OR 1.1 (0.84-1.44)	0.0	5[178,214,215,222,224]	3,370	no	no	yes	no	no	+++
AMR: North	Hypertension	HR 1.59 (1.1-2.3)	0.0	1[170]	841	no	na	no	no	no	++++
AMR: North	Hypertension	OR 0.88 (0.74-1.04)	0.0	9[174,178,185,190,197,214,215,222,224]	4,327	no	no	yes	no	no	+++
AMR: North	Hypertension	RR 0.76 (0.48-1.2)	0.0	1[204]	504	no	na	yes	yes, NOS =4	no	++
AMR: North	Peripheral vascular disease	OR 1.08 (0.69-1.7)	0.0	1[224]	2,015	no	na	yes	no	no	+++
AMR: North	V.thromboembolism	OR 0.76 (0.44-1.3)	0.0	1[224]	2,015	no	na	yes	no	no	+++
EUR	Hypertension	OR range 0.27-2.29	84.2	2[155,165]	269	no	yes	yes	no	no	++
WPR	Hypertension	OR 1.9 (1.48-5.27)	0.0	1[242]	641	no	na	no	no	no	++++

AMR, regions of America; AFR, African region; HR, hazard ratio; EMR, Eastern Mediterranean region; EUR, European region; na, not applicable; OR, odds ratio; RR, risk ratio; WPR, Western Pacific region

Table 9. 4 Risk of intubation in persons with overweight, obesity or underweight: meta-analysis and confidence in the estimate (GRADE)

Region	Condition	Estimate (95% CI)	I^2 (%)	Studies	Patients	Indirectness	Inconsistency	Imprecision	Bias	Publ. Bias	Quality
AMR: North	Obesity/BMI≥30	OR 1.54 (1.31-1.82)	0.0	10[174,178,185,186,190,195,197,207,215,224]	4,986	no	no	no	no	yes*	+++
AMR: North	Obesity/BMI≥30	RR 1.92 (1.49-2.46)	10.7	2[204,218]	5,422	no	no	no	no	no	++++
AMR: North	Obesity/BMI≥40	OR 2.57 (1.57-4.22)	6.5	2[186,222]	949	no	no	no	no	no	++++
AMR: North	Overweight	OR 1.15 (0.79-1.66)	21.9	3[190,215,224]	2,554	no	no	yes	no	no	+++
AMR: North	Overweight	RR 2 (1.2-3.3)	0.0	1[204]	504	no	na	no	yes, NOS =4	no	+++
AMR: North	Underweight	OR 0.76 (0.26-2.22)	0.0	1[207]	200	no	na	yes	no	no	+++
EUR	Obesity/BMI≥30	OR 4.93 (1.75-13.88)	0.0	1[165]	124	no	na	no	no	no	++++
EUR	Overweight	OR 1.69 (0.52-5.48)	0.0	1[165]	124	no	na	yes	no	no	+++

*Beggs: tau=0.6,pval=0.0099; Egger: zval=3.3577,pval=8e-04

AMR, regions of America; AFR, African region; HR, hazard ratio; EMR, Eastern Mediterranean region; EUR, European region; na, not applicable; OR, odds ratio; RR, risk ratio; WPR, Western Pacific region

Table 9. 5 Risk of intubation in persons with immunodeficiency: meta-analysis and confidence in the estimate (GRADE)

Region	Condition	Estimate (95% CI)	I^2 (%)	Studies	Patients	Indirectness	Inconsistency	Imprecision	Bias	Publ. Bias	Quality
AMR: North	HIV	HR 1.05 (0.46-2.4)	0	1[170]	841	no	na	yes	no	no	+++
AMR: North	Inflammatory bowel disease	HR 0.34 (0.05-2.43)	0	1[170]	841	no	na	yes	no	no	+++
AMR: North	Immunosuppression	HR 0.96 (0.33-2.8)	0	1[170]	841	no	na	yes	no	no	+++
AMR: North	Organ transplant recipients	HR 1.03 (0.53-2.04)	0	1[170]	841	no	na	yes	no	no	+++
AMR: North	Rheumatological disease	HR 1.09 (0.54-2.18)	0	1[170]	841	no	na	yes	no	no	+++

AMR, regions of America; HR, hazard ratio; na, not applicable

Table 9. 6 Risk of intubation in persons with neurological diseases or mental health disorders: meta-analysis and confidence in the estimate (GRADE)

Region	Condition	Estimate (95% CI)	I^2 (%)	Studies	Patients	Indirectness	Inconsistency	Imprecision	Bias	Publ. Bias	Quality
AMR: North	Cerebrovascular/Stroke	HR 0.96 (0.57-1.62)	0	1[170]	841	no	na	yes	no	no	+++
AMR: North	Cerebrovascular/Stroke	OR 0.81 (0.56-1.18)	0	1[224]	2,015	no	na	yes	no	no	+++
AMR: North	Dementia	OR 0.52 (0.34-0.8)	0	1[224]	2,015	no	na	no	no	no	++++

AMR, regions of America; HR, hazard ratio; na, not applicable; OR, odds ratio

Table 9. 7 Risk of intubation in persons with oncological diseases: meta-analysis and confidence in the estimate (GRADE)

Region	Condition	Estimate (95% CI)	I^2 (%)	Studies	Patients	Indirectness	Inconsistency	Imprecision	Bias	Publ. Bias	Quality
AMR: North	Cancer	OR range 0.82-2.5	86.9	2[222,224]	2,478	no	yes	yes	no	no	++
AMR: North	Cancer/Active	HR 0.65 (0.33-1.3)	0.0	1[170]	841	no	na	yes	no	no	+++
AMR: North	Cancer/Active	OR 0.55 (0.15-2.05)	0.0	1[215]	436	no	na	yes	no	no	+++
WPR	Cancer	OR 2.71 (0.45-5.87)	0.0	1[242]	641	no	na	yes	no	no	+++

AMR, regions of America; HR, hazard ratio; na, not applicable; OR, odds ratio; WPR, Western Pacific region

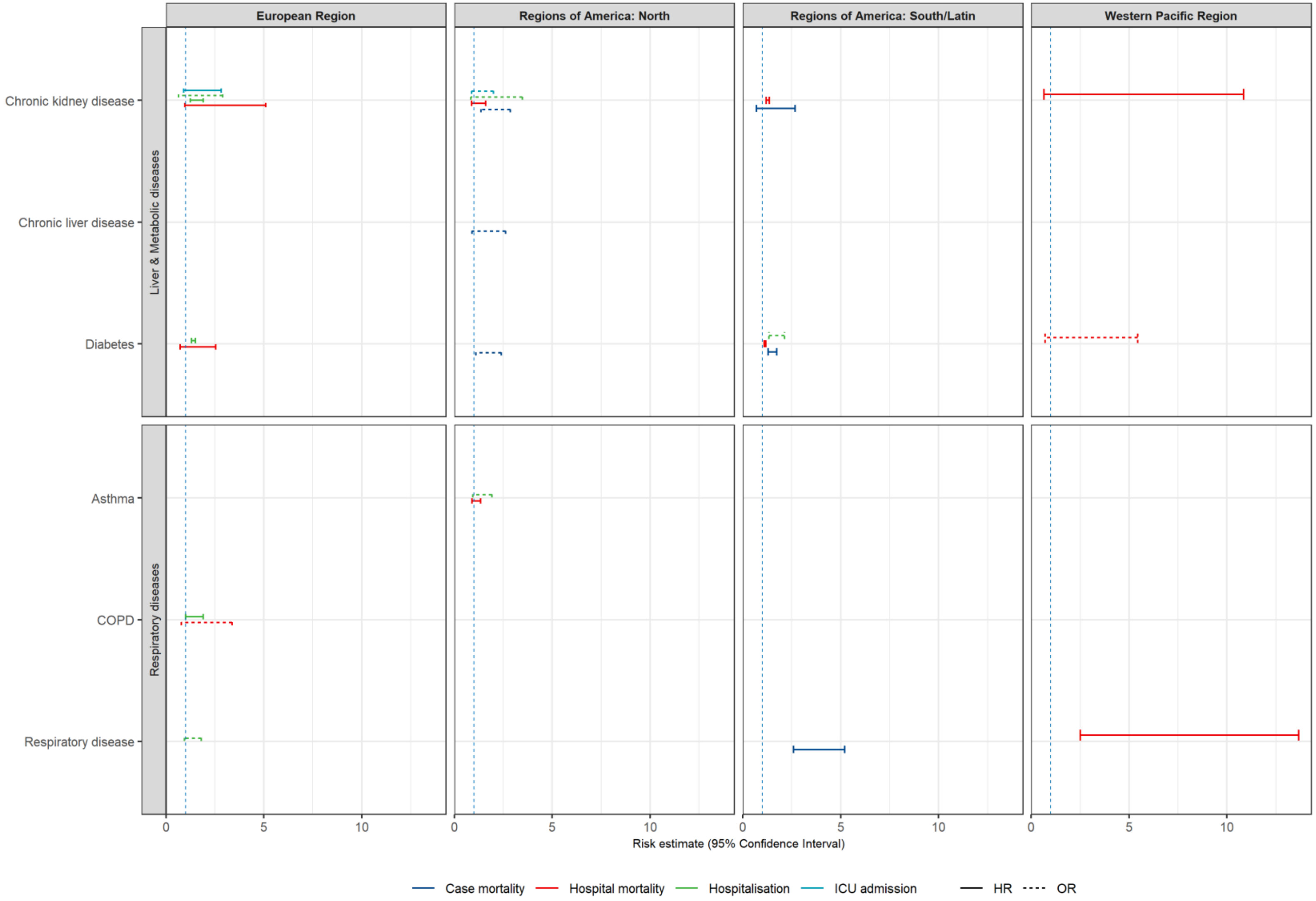

Figure 4. Ranges of the estimates with considerable between-study heterogeneity for respiratory and liver and metabolic diseases

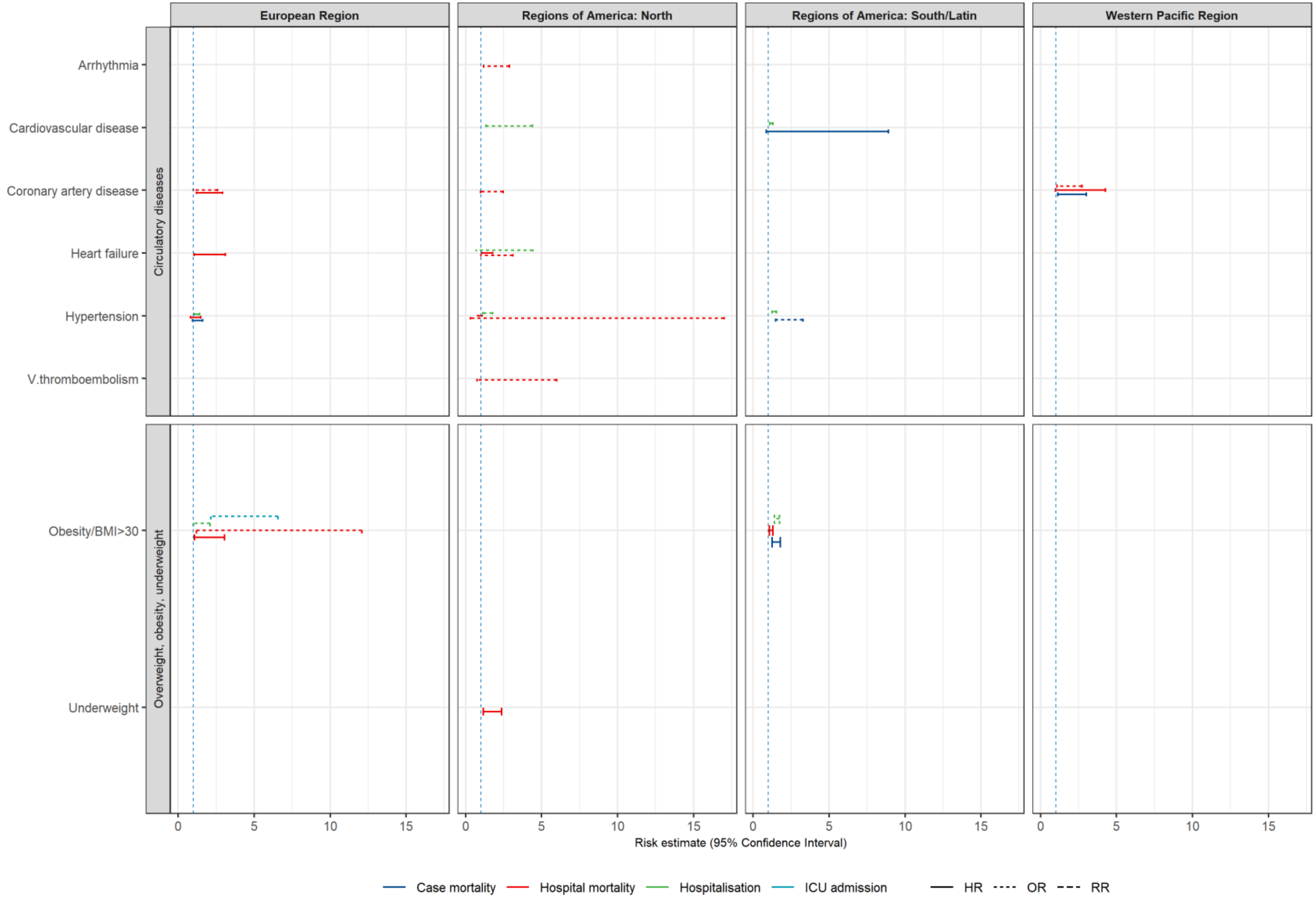

Figure 5. Ranges of the estimates with considerable between-study heterogeneity for circulatory diseases, overweight, obesity, and underweight

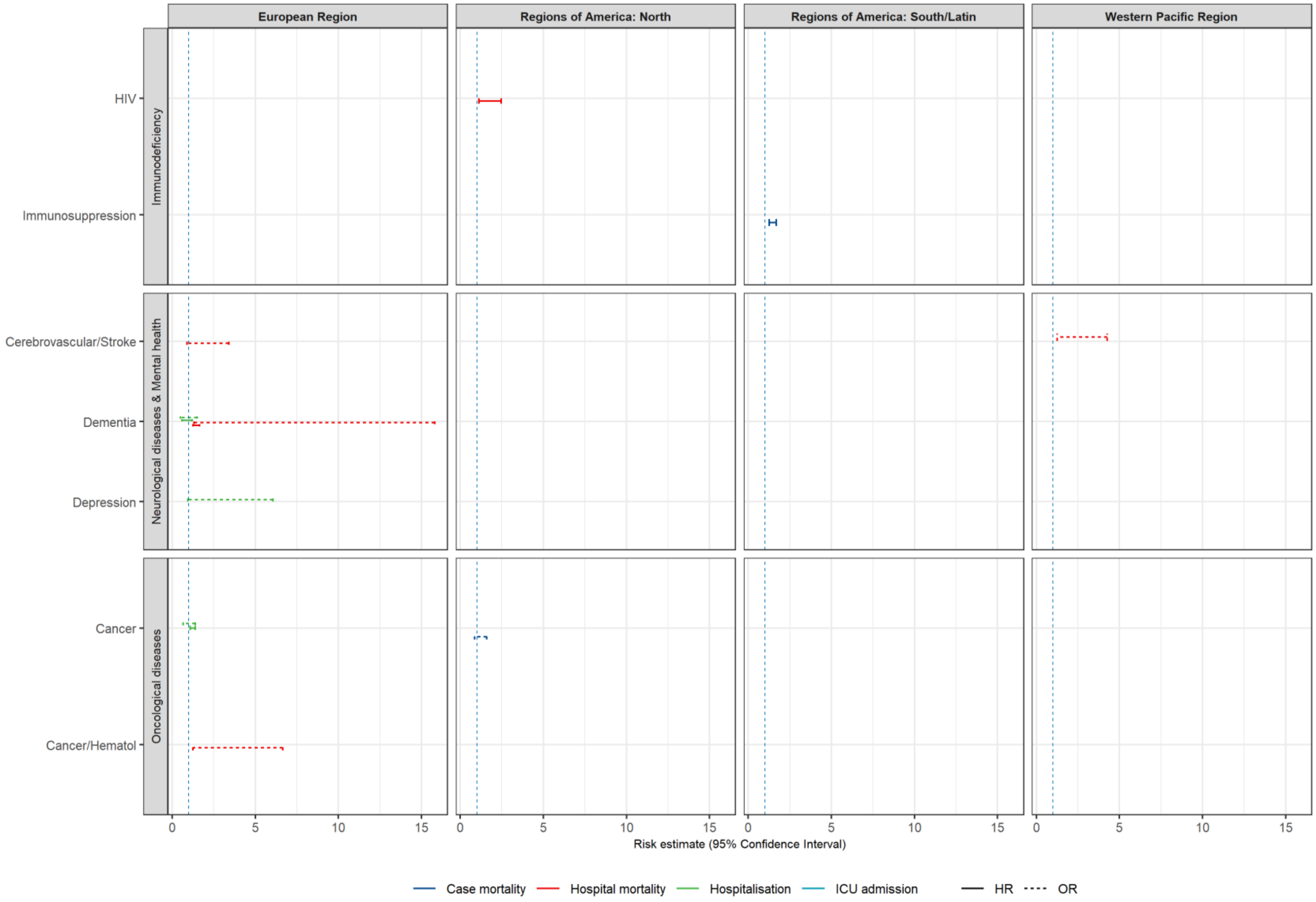

Figure 6. Ranges of the estimates with considerable between-study heterogeneity for immunodeficiency, oncological disease, neurological and mental health disorders

2.6. Estimated associations supported by high quality of evidence

Table 10 Estimated associations supported by high quality of evidence (GRADE) presented for each pre-existing condition and outcome across the WHO-regions

Pre-existing condition	African Region		Regions of America: North				Regions of America: South/Latin				Eastern Mediterranean Region		European Region				Western Pacific Region			
	Hospital mortality	Case mortality	Hospitalisation	ICU admission	Hospital mortality	Case mortality	Hospitalisation	ICU admission	Hospital mortality	Case mortality	ICU admission	Hospital mortality	Hospitalisation	ICU admission	Hospital mortality	Case mortality	Hospitalisation	ICU admission	Hospital mortality	Case mortality
Chronic kidney disease	HR 1.51 (1.2-1.89) [121]	HR 1.92 (1.51-2.45) [121]	-	-	RR 1.33 (1.1-1.61) [192]	HR 1.53 (1.32-1.78) [189]	pOR 2.21 (1.94-2.51) [230,233,235]	-	-	OR 1.44 (1.01-2.06) [230]	-	-	-	OR 4.8 (1.83-12.6) [160]	pOR 1.92 (1.57-2.36) [147,153,156,160]	pHR 1.31 (1.21-1.42) [131,159], OR 1.9 (1.4-2.6) [156]	-	-	pOR 2.65 (1.45-4.84) [240,254]	-
Chronic liver disease	-	-	RR 1.3 (1.1-1.6) [219]	-	-	RR 3 (1.5-6) [219]	-	-	-	-	-	-	-	-	HR 1.51 (1.21-1.88) [140]	-	-	-	-	-
Chronic liver/Cirrhosis	-	-	-	-	pOR 5.96 (1.29-27.66) [185,214]	-	-	-	-	-	-	-	-	-	HR 2.03 (1.31-3.13) [127]	-	-	-	-	-
Diabetes	-	HR 2.02 (1.47-2.76) [121]	pOR 2.03 (1.73-2.38) [171,178,191,199,209,212,224,290]	pOR 1.4 (1.12-1.75) [178,185,190,197,215,222], RR 1.13 (1.03-1.24) [192]	pRR 1.18 (1.06-1.32) [192,204,221]	HR 1.21 (1.06-1.4) [189]	-	OR 1.87 (1.41-4.26) [231]	-	OR 1.5 (1.13-1.98) [230]	OR 5.49 (3.13-9.65) [122]	OR 1.64 (1.24-2.18) [123]	pOR 1.77 (1.52-2.07) [151,156,166]	pHR 1.45 (1.1-1.91) [141,160], pOR 1.83 (1.12-2.98) [124,160]	pOR 1.26 (1.02-1.56) [132,147,153,156,160]	pHR 1.35 (1.18-1.54) [131,159], OR 1.6 (1.3-2) [156]	-	HR 4.7 (1.6-14.1) [238]	-	-
Dyslipidemia or hyperlipidemia	-	-	-	-	RR 0.75 (0.57-0.98) [221]	RR 1.47 (1.02-2.11) [223]	-	-	-	-	-	-	-	-	-	-	-	-	-	-
Asthma	-	-	-	-	-	-	OR 0.73 (0.65-0.81) [233]	-		-	-	-	-	-	OR 0.42 (0.19-0.91) [153]	-	-	-	-	-
COPD	-	-	pOR 1.72 (1.23-2.4) [171,199,212,224]	-	pHR 1.23 (1.03-1.47) [205,217,226], RR 1.41 (1.06-1.88) [221]	-	pOR 1.47 (1.3-1.67) [230,233]	-	HR 1.12 (1.07-1.18) [234]	pHR 1.49 (1.22-1.84) [229,236], OR 1.68 (1.22-2.31) [230]	-	-	-	-	HR 1.15 (1.03-1.29) [131]	-	-	OR 3.27 (1.09-7.63) [242]	pHR 1.74 (1.03-2.95) [258,264,275], pOR 2.22 (1.55-3.19) [240,242,254,273]	pHR 1.43 (1.01-2.03) [247,251]
Interstitial lung disease	-	-	-	-	HR 2.17 (1.76-2.69) [205]	-	-	-	-	-	-	-	-	-	-	-	-	-	-	-
Obstructive sleep apnea	-	-	RR 1.23 (1.01-1.49) [172]	-	-	-	-	-	-	-	-	-	-	-	-	-	-	-	-	-
Respiratory disease	HR 0.68 (0.53-0.86) [121]	-	-	-	pOR 1.69 (1.04-2.74) [185,197,210], RR 1.31 (1.13-1.52) [192]	pOR 1.24 (1.08-1.42) [184,290]	OR 1.46 (1.12-1.9) [235]	-	HR 1.21 (1.06-1.38) [228]	-	-	-	-	-	pHR 1.17 (1.09-1.26) [139-141,157]	OR 1.4 (1.1-1.8) [156]	-	HR 6.2 (1.9-20.1) [238]	-	-
Tuberculosis	HR 1.3 (1.05-1.63) [121]	HR 1.57 (1.25-1.97) [121]	-	-	-	-	-	-	-	-	-	-	-	-	-	-	RR 1.2 (1.04-1.38) [260]	-	RR 2.25 (1.35-3.75) [260]	RR 2.17 (1.4-3.37) [260]

Pre-existing condition	African Region		Regions of America: North				Regions of America: South/Latin				Eastern Mediterranean Region		European Region				Western Pacific Region			
	Hospital mortality	Case mortality	Hospitalisation	ICU admission	Hospital mortality	Case mortality	Hospitalisation	ICU admission	Hospital mortality	Case mortality	ICU admission	Hospital mortality	Hospitalisation	ICU admission	Hospital mortality	Case mortality	Hospitalisation	ICU admission	Hospital mortality	Case mortality
Arrhythmia	-	-	OR 1.49 (1.03-2.14) [224]	-	pOR 1.29 (1.02-1.62) [188,224]	-	-	-	-	-	-	-	HR 1.5 (1.2-1.9) [159], pOR 1.39 (1.17-1.65) [156,166]	-	pOR 1.37 (1.07-1.74) [153,156]	HR 1.8 (1.3-2.5) [159], OR 1.6 (1.2-2) [156]	-	-	-	-
Cardiovascular disease	-	-	-	-	RR 1.28 (1.03-1.58) [192]	-	-	-	-	-	-	-	-	-	pHR 1.9 (1.19-3.01) [124,157]	-	-	-	pHR 1.95 (1.46-2.61) [241,245,249,257,258,264,275]	-
Coronary artery disease	-	-	-	-	pOR 1.19 (1.03-1.38) [188,193,195,207,216,224], pHR 1.17 (1.07-1.28) [170,209,226]	-	-	-	-	-	-	-	OR 1.4 (1.2-1.7) [156]	-	-	HR 1.7 (1.2-2.5) [159]	-	-	-	-
Heart disease	-	-	-	-	-	-	-	-	-	-	-	-	HR 1.1 (1.05-1.16) [131]	-	pHR 1.2 (1.12-1.28) [131,140]	-	-	-	pOR 4.54 (1.47-14.01) [256,263]	-
Heart failure	-	-	-	-	pOR 1.31 (1.08-1.6) [188,193,195,207,214-216,224]	OR 1.42 (1.21-1.67) [184]	-	-	-	-	-	-	HR 1.6 (1.2-2.1) [159], OR 2.6 (2-3.4) [156]	-	pOR 1.37 (1.02-1.84) [153,156]	HR 2.3 (1.6-3.2) [159], OR 1.8 (1.3-2.4) [156]	-	-	HR 3.3 (1.33-8.19) [246]	-
Hypertension	-	-	-	-	pHR 0.88 (0.8-0.95) [170,200,205,209]	-	-	OR 1.77 (1.37-2.29) [231]	HR 1.08 (1.05-1.11) [234]	HR 1.38 (1.09-1.75) [236]	-	-	pOR 1.69 (1.51-1.89) [151,156,166]	pHR 1.37 (1.02-1.83) [141,160]	-	OR 1.3 (1.1-1.6) [156]	-	HR 4.6 (1.8-11.6) [238]	pHR 1.85 (1.48-2.3) [245,246,252,257,265,268,272,275]	-
Infarction	-	-	-	-	-	OR 1.97 (1.64-2.35) [184], RR 1.66 (1.04-2.64) [223]	-	-	-	-	-	-	-	-	-	-	-	-	-	-
Obesity/BMI>30	-	-	pOR 1.63 (1.43-1.84) [178,181,191,199,209,211,224], pRR 1.12 (1.04-1.21) [172,218]	pOR 1.46 (1.06-2.02) [178,185,190,197,215], RR 1.31 (1.16-1.47) [192]	-	HR 1.42 (1.19-1.7) [189]	-	OR 1.43 (1.11-1.83) [231]	-	OR 1.74 (1.35-2.26) [230]	OR 2.27 (1.15-4.47) [122]	-	pHR 1.59 (1.52-1.66) [131,159]	HR 4.22 (3.02-5.91) [160]	-	OR 1.5 (1.1-2) [156]	-	-	-	-
Obesity/BMI>40	-	-	OR 2.45 (1.78-3.36) [209]	OR 2.0 (1.4-3.6) [222]	pOR 1.7 (1.26-2.29) [193,216], pHR 1.41 (1.03-1.93) [180,209]	RR 3.29 (2.07-5.22) [223]	-	-	-	-	-	-	-	-	-	-	-	-	-	-
Overweight	-	-	pOR 1.33 (1.16-1.53) [181,209,224]	-	-	-	-	-	-	-	-	-	-	-	-	-	-	-	-	-
Cerebrovascular/Stroke	-	-	OR 2.25 (1.42-3.58) [224]	-	-	-	-	-	-	-	-	-	pOR 1.3 (1.08-1.56) [156,166]	-	HR 2.12 (1.29-3.47) [160]	OR 1.4 (1.1-1.8) [156]	-	-	-	-

Pre-existing condition	African Region		Regions of America: North				Regions of America: South/Latin				Eastern Mediterranean Region		European Region				Western Pacific Region			
	Hospital mortality	Case mortality	Hospitalisation	ICU admission	Hospital mortality	Case mortality	Hospitalisation	ICU admission	Hospital mortality	Case mortality	ICU admission	Hospital mortality	Hospitalisation	ICU admission	Hospital mortality	Case mortality	Hospitalisation	ICU admission	Hospital mortality	Case mortality
Dementia	-	-	OR 3.6 (2.12-6.09) [224]	-	OR 2.03 (1.46-2.83) [224]	OR 1.29 (1.07-1.56) [184], HR 1.47 (1.21-1.78) [189]	-	-	-	-	-	-	-	-	-	pHR 1.92 (1.77-2.08) [131,159], OR 2 (1.5-2.6) [156]	-	-	HR 7.7 (1.5-39.61) [249]	-
Neurological disease	-	-	-	-	RR 1.25 (1.04-1.5) [192]	-	-	-	HR 1.34 (1.16-1.54) [228]	HR 3.9 (1.9-7.8) [237]	-	-	-	-	pHR 1.18 (1.08-1.28) [127,140]	-	-	-	-	-
Psychiatric disorder	-	-	-	-	-	-	-	-	-	-	-	-	pOR 1.72 (1.05-2.84) [151,156]	-	OR 2.9 (1.3-6.6) [156]	OR 2.5 (1.2-5.1) [156]	-	-	-	-
Autoimmune condition	-	-	-	-	-	-	-	-	-	-	-	-	HR 1.08 (1.01-1.17) [131]	-	HR 1.19 (1.06-1.33) [131]	-	-	-	-	-
HIV	HR 1.45 (1.14-1.84) [121]	HR 1.7 (1.32-2.18) [121]	-	-	OR 0.07 (0.03-0.52) [206]	-	-	-	-	-	-	-	-	-	HR 1.5 (1.02-2.22) [142]	-	-	-	-	-
Immunosuppression	-	-	-	RR 1.29 (1.13-1.47) [192]	OR 3.6 (1.52-8.47) [216], RR 1.39 (1.13-1.7) [192]	-	OR 1.85 (1.59-2.15) [233]	-	pHR 1.09 (1.03-1.16) [228,234]	OR 1.7 (1.13-2.55) [230]	-	-	-	-	OR 2.11 (1.08-4.09) [130]	-	-	-	-	-
Organ transplant recipients	-	-	-	-	-	RR 6.54 (2.66-16.12) [223]	-	-	-	-	-	-	OR 3.4 (1.7-6.6) [156]	-	OR 4.2 (1.6-11.4) [156]	OR 3.2 (1.3-8.4) [156]	-	-	-	-
Rheumatological disease	-	-	-	-	-	-	-	-	-	-	-	-	OR 1.5 (1.1-1.9) [156]	-	-	-	-	-	-	-
Cancer	-	-	-	-	pHR 1.19 (1.06-1.33) [200,205,209,226]	-	-	-	-	-	-	-	-	-	pHR 1.2 (1.11-1.3) [131,139,140]	pHR 1.25 (1.15-1.36) [131,159]	-	-	pOR 3.23 (2.33-4.48) [240,242,254,255]	-
Cancer/Active	-	-	-	-	-	-	-	-	-	-	-	-	-	-	pHR 1.44 (1.16-1.79) [127,137,141], OR 4.68 (1.47-14.88) [126]	-	-	-	-	-
Cancer/Hematological	-	-	-	-	-	-	-	-	-	-	-	-	-	-	HR 1.74 (1.28-2.37) [164]	-	-	-	-	-
Cancer/Solid	-	-	-	-	-	OR 1.7 (1.19-2.43) [184]	-	-	-	-	-	-	-	-	-	-	-	-	-	-

HR, hazard ratio; OR, odds ratio; pHR; pooled hazard ratio; pOR, pooled odds ratio; RR, risk ratio

2.7. Age-stratified estimates

Table 11 Age-stratified estimates extracted from single studies (as reported by primary studies)

Pre-existing condition	Outcome	Age group	Estimate	Region	Sample size	Study
Coronary artery disease	Hospital mortality	<=50	OR 0.6 (0.2-2.1)	Regions of America: North	572	Klang et al.[193]
Coronary artery disease	Hospital mortality	>50	OR 1.3 (1.1-1.6)	Regions of America: North	2,834	Klang et al. [193]
Cardiovascular disease	Hospital mortality	<65	OR 1.14 (0.6-2.16)	Eastern Mediterranean Region	2,957	Rastad et al.[123]
Cardiovascular disease	Hospital mortality	>=65	OR 1.2 (0.8-1.78)	Eastern Mediterranean Region	2,957	Rastad et al. [123]
Heart failure	Hospital mortality	<=50	OR 4.0 (1.6-10.4)	Regions of America: North	572	Klang et al. [193]
Heart failure	Hospital mortality	<65	HR 1.38 (0.56-3.37)	European Region	1,761	Di Castelnuovo et al.[139]
Heart failure	Hospital mortality	>50	OR 1.0 (0.8-1.3)	Regions of America: North	2,834	Klang et al. [193]
Heart failure	Hospital mortality	65-74	HR 1.12 (0.66-1.92)	European Region	808	Di Castelnuovo et al. [139]
Heart failure	Hospital mortality	>=75	HR 0.95 (0.74-1.23)	European Region	1,315	Di Castelnuovo et al. [139]
Infarction	Hospital mortality	<65	HR 1.19 (0.43-3.28)	European Region	1,761	Di Castelnuovo et al. [139]
Infarction	Hospital mortality	65-74	HR 1.28 (0.75-2.2)	European Region	808	Di Castelnuovo et al. [139]
Infarction	Hospital mortality	>=75	HR 1.06 (0.82-1.39)	European Region	1,315	Di Castelnuovo et al. [139]
Hypertension	Intubation	45-64	RR 0.91 (0.4-2.0)	Regions of America: North	188	Nakeshbandi et al.[204]
Hypertension	Intubation	>=65	RR 0.69 (0.38-1.3)	Regions of America: North	316	Nakeshbandi et al. [204]
Hypertension	Hospital mortality	<=50	OR 0.5 (0.2-1.1)	Regions of America: North	572	Klang et al. [193]
Hypertension	Hospital mortality	45-64	RR 0.73 (0.34-1.5)	Regions of America: North	188	Nakeshbandi et al.
Hypertension	Hospital mortality	<65	HR 0.85 (0.54-1.33)	European Region	1,761	Di Castelnuovo et al. [139]
Hypertension	Hospital mortality	>50	OR 1.1 (0.9-1.3)	Regions of America: North	2,834	Klang et al. [193]
Hypertension	Hospital mortality	65-74	HR 0.92 (0.61-1.39)	European Region	808	Di Castelnuovo et al. [139]
Hypertension	Hospital mortality	>=65	RR 0.81 (0.64-1.0)	Regions of America: North	316	Nakeshbandi et al. [204]
Hypertension	Hospital mortality	>=75	HR 0.87 (0.67-1.14)	European Region	1,315	Di Castelnuovo et al. [139]

Pre-existing condition	Outcome	Age group	Estimate	Region	Sample size	Study
Chronic kidney disease	Hospital mortality	<=50	OR 3.3 (1.4-7.7)	Regions of America: North	572	Klang et al. [193]
Chronic kidney disease	Hospital mortality	>50	OR 1.7 (1.4-2.1)	Regions of America: North	2,834	Klang et al. [193]
CKD/eGFR60-89	Hospital mortality	<65	HR 1.76 (0.94-3.32)	European Region	1,761	Di Castelnuovo et al. [139]
CKD/eGFR60-89	Hospital mortality	65-74	HR 1.49 (0.86-2.59)	European Region	808	Di Castelnuovo et al. [139]
CKD/eGFR60-89	Hospital mortality	>=75	HR 1.1 (0.61-2.01)	European Region	1,315	Di Castelnuovo et al. [139]
CKD/eGFR59-45	Hospital mortality	<65	HR 3.3 (1.46-7.46)	European Region	1,761	Di Castelnuovo et al. [139]
CKD/eGFR59-45	Hospital mortality	65-74	HR 1.93 (1.11-3.37)	European Region	808	Di Castelnuovo et al. [139]
CKD/eGFR59-45	Hospital mortality	>=75	HR 1.84 (1.02-3.32)	European Region	1,315	Di Castelnuovo et al. [139]
CKD/eGFR44-30	Hospital mortality	<65	HR 3.24 (1.41-7.45)	European Region	1,761	Di Castelnuovo et al. [139]
CKD/eGFR44-30	Hospital mortality	65-74	HR 3.33 (1.78-6.23)	European Region	808	Di Castelnuovo et al. [139]
CKD/eGFR44-30	Hospital mortality	>=75	HR 2.36 (1.38-4.02)	European Region	1,315	Di Castelnuovo et al. [139]
CKD/eGFR15-29	Hospital mortality	<65	HR 13.89 (3.09-62.5)	European Region	1,761	Di Castelnuovo et al. [139]
CKD/eGFR15-29	Hospital mortality	65-74	HR 4.86 (2.18-10.82)	European Region	808	Di Castelnuovo et al. [139]
CKD/eGFR15-29	Hospital mortality	>=75	HR 3 (1.62-5.54)	European Region	1,315	Di Castelnuovo et al. [139]
CKD/eGFR<15	Hospital mortality	<65	HR 6.65 (1.66-26.73)	European Region	1,761	Di Castelnuovo et al. [139]
CKD/eGFR<15	Hospital mortality	65-74	HR 4.77 (1.63-13.9)	European Region	808	Di Castelnuovo et al. [139]
CKD/eGFR<15	Hospital mortality	>=75	HR 3.65 (1.92-6.96)	European Region	1,315	Di Castelnuovo et al. [139]
Diabetes	Intubation	45-64	RR 1.2 (0.69-2.1)	Regions of America: North	188	Nakeshbandi et al. [204]
Diabetes	Intubation	>=65	RR 1.2 (0.79-1.9)	Regions of America: North	316	Nakeshbandi et al. [204]
Diabetes	Hospital mortality	<=50	OR 1.3 (0.7-2.6)	Regions of America: North	572	Klang et al. [193]
Diabetes	Hospital mortality	45-64	RR 1.6 (0.92-2.8)	Regions of America: North	188	Nakeshbandi et al. [204]
Diabetes	Hospital mortality	<65	HR 2.0 (1.15-3.5)	European Region	1,761	Di Castelnuovo et al. [139]

Pre-existing condition	Outcome	Age group	Estimate	Region	Sample size	Study
Diabetes	Hospital mortality	<65	OR 1.7 (0.97-2.97)	Eastern Mediterranean Region	2,957	Rastad et al. [123]
Diabetes	Hospital mortality	>50	OR 1.4 (1.2-1.7)	Regions of America: North	2,834	Klang et al. [193]
Diabetes	Hospital mortality	65-74	HR 0.86 (0.59-1.25)	European Region	808	Di Castelnuovo et al. [139]
Diabetes	Hospital mortality	>=65	RR 1.0 (0.84-1.2)	Regions of America: North	316	Nakeshbandi et al. [204]
Diabetes	Hospital mortality	>=65	OR 1.6 (1.01-2.52)	Eastern Mediterranean Region	2,957	Rastad et al. [123]
Diabetes	Hospital mortality	>=75	HR 0.96 (0.78-1.19)	European Region	1,315	Di Castelnuovo et al. [139]
Diabetes	Case mortality	< 40	HR 2.86 (2.19-3.76)	Regions of America: South Latin	51,633	Bello-Chavolla et al.[229]
Respiratory disease	Hospital mortality	<65	HR 1.09 (0.41-2.88)	European Region	1,761	Di Castelnuovo et al. [139]
Respiratory disease	Hospital mortality	65-74	HR 1.76 (1.16-2.67)	European Region	808	Di Castelnuovo et al. [139]
Respiratory disease	Hospital mortality	>=75	HR 1.11 (0.89-1.39)	European Region	1,315	Di Castelnuovo et al. [139]
Asthma	Hospitalisation	<=50	OR 0.98 (0.58-1.66)	Regions of America: North	502	Mahdavinia et al.[196]
Asthma	Hospitalisation	50-65	OR 1.17 (0.62-2.19)	Regions of America: North	278	Mahdavinia et al. [196]
Asthma	Hospitalisation	>=65	OR 1.37 (0.63-3.01)	Regions of America: North	155	Mahdavinia et al. [196]
Asthma	Intubation	<=50	OR 1.04 (0.34-2.62)	Regions of America: North	502	Mahdavinia et al. [196]
Asthma	Intubation	50-65	OR 1.24 (1.0-1.5)	Regions of America: North	278	Mahdavinia et al. [196]
Asthma	Intubation	>=65	OR 1.14 (0.4-3.25)	Regions of America: North	155	Mahdavinia et al. [196]
Asthma	Case mortality	50-65	OR 1.19 (0.21-6.67)	Regions of America: North	278	Mahdavinia et al. [196]
Cancer	Intubation	<=50	RR 1.48 (0.37-5.9)	Regions of America: North	2,088	Miyashita. H et al.[202]
Cancer	Intubation	50-65	RR 1.31 (0.66-2.6)	Regions of America: North	1,641	Miyashita. H et al.[202]
Cancer	Intubation	65-80	RR 1.76 (1.15-2.7)	Regions of America: North	1,134	Miyashita. H et al.[202]
Cancer	Intubation	>80	RR 1.17 (0.49-2.83)	Regions of America: North	625	Miyashita. H et al.[202]
Cancer	Hospital mortality	<=50	OR 2.5 (1.0-6.5)	Regions of America: North	572	Klang et al. [193]
Cancer	Hospital mortality	<65	HR 4.76 (2.46-9.21)	European Region	1,761	Di Castelnuovo et al. [139]

Pre-existing condition	Outcome	Age group	Estimate	Region	Sample size	Study
Cancer	Hospital mortality	>50	OR 1.0 (0.8-1.2)	Regions of America: North	2,834	Klang et al. [193]
Cancer	Hospital mortality	65-74	HR 1.65 (0.95-2.86)	European Region	808	Di Castelnuovo et al. [139]
Cancer	Hospital mortality	>=65	HR 1.12 (0.56-2.24)	Western Pacific Region	660	Li.Q et al.[253]
Cancer	Hospital mortality	>=75	HR 1.1 (0.86-1.4)	European Region	1,315	Di Castelnuovo et al. [139]
Cancer	Case mortality	50-65	RR 0.63 (0.24-1.68)	Regions of America: North	1,641	Miyashita. H et al.[202]
Cancer	Case mortality	65-80	RR 0.72 (0.44-1.19)	Regions of America: North	1,134	Miyashita. H et al.[202]
Cancer	Case mortality	>=65	RR 5.01 (1.55-16.2)	Regions of America: North	2,088	Miyashita. H et al.[202]
Cancer	Case mortality	>80	RR 0.94 (0.6-1.48)	Regions of America: North	625	Miyashita. H et al.[202]
HIV	Intubation	<=50	RR 2.97 (1.29-6.84)	Regions of America: North	137	Miyashita. H et al.[201]
HIV	Intubation	50-65	RR 1.33 (0.74-2.42)	Regions of America: North	234	Miyashita. H et al.[201]
HIV	Intubation	>=65	RR 0.86 (0.34-2.19)	Regions of America: North	381	Miyashita. H et al.[201]
HIV	ICU	<=50	RR 1.6 (0.81-3.14)	Regions of America: North	351	Miyashita. H et al.[201]
HIV	ICU	50-65	RR 1.08 (0.76-1.54)	Regions of America: North	660	Miyashita. H et al.[201]
HIV	ICU	>=65	RR 0.5 (0.24-1.06)	Regions of America: North	971	Miyashita. H et al.[201]
HIV	Case mortality	<=50	RR 4.36 (1.43-13.3)	Regions of America: North	57	Miyashita. H et al.[201]
HIV	Case mortality	50-65	RR 1.4 (0.82-2.38)	Regions of America: North	269	Miyashita. H et al.[201]
HIV	Case mortality	>=65	RR 0.7 (0.38-1.31)	Regions of America: North	932	Miyashita. H et al.[201]
Dementia	Hospitalisation	60-79	RR 1.09 (0.86-1.4)	Regions of America: North	514	Miyashita. S et al.[203]
Dementia	Hospitalisation	>80	RR 1.15 (1.02-1.29)	Regions of America: North	514	Miyashita. S et al.[203]
Dementia	Intubation	>80	RR 0.54 (0.2-1.46)	Regions of America: North	514	Miyashita. S et al.[203]
Dementia	ICU	60-79	RR 1.03 (0.59-1.8)	Regions of America: North	514	Miyashita. S et al.[203]
Dementia	ICU	>80	RR 0.96 (0.61-1.52)	Regions of America: North	514	Miyashita. S et al.[203]
Dementia	Case mortality	60-79	RR 2.3 (1.41-3.76)	Regions of America: North	514	Miyashita. S et al.[203]

Pre-existing condition	Outcome	Age group	Estimate	Region	Sample size	Study
Dementia	Case mortality	>80	RR 1.21 (0.88-1.64)	Regions of America: North	514	Miyashita. S et al.[203]
Overweight	Intubation	45-64	RR 2.3 (0.72-7.1)	Regions of America: North	188	Nakeshbandi et al. [204]
Overweight	Intubation	>=65	RR 1.8 (0.97-3.2)	Regions of America: North	316	Nakeshbandi et al. [204]
Overweight	Hospital mortality	45-64	RR 1.05 (0.44-2.5)	Regions of America: North	188	Nakeshbandi et al. [204]
Overweight	Hospital mortality	>=65	RR 1.5 (1.2-2.0)	Regions of America: North	316	Nakeshbandi et al. [204]
Obesity/BMI>30	Intubation	45-64	RR 3.0 (1.1-8.0)	Regions of America: North	188	Nakeshbandi et al. [204]
Obesity/BMI>30	Intubation	>=65	RR 2.1 (1.1-3.8)	Regions of America: North	316	Nakeshbandi et al. [204]
Obesity/BMI>30	Hospital mortality	<=45	OR 1.35 (1.07-1.7)	Western Pacific Region	53	Zhang.F et al.[270]
Obesity/BMI>30	Hospital mortality	<=50	OR 1.1 (0.5-2.3)	Regions of America: North	572	Klang et al. [193]
Obesity/BMI>30	Hospital mortality	45-64	RR 1.5 (0.77-2.9)	Regions of America: North	188	Nakeshbandi et al. [204]
Obesity/BMI>30	Hospital mortality	<65	HR 1.36 (0.75-2.46)	European Region	1,761	Di Castelnuovo et al. [139]
Obesity/BMI>30	Hospital mortality	>50	OR 1.1 (0.9-1.3)	Regions of America: North	2,834	Klang et al. [193]
Obesity/BMI>30	Hospital mortality	65-74	HR 1.5 (0.92-2.45)	European Region	808	Di Castelnuovo et al. [139]
Obesity/BMI>30	Hospital mortality	>=65	RR 1.3 (0.94-1.7)	Regions of America: North	316	Nakeshbandi et al. [204]
Obesity/BMI>30	Hospital mortality	>=75	HR 1.05 (0.73-1.52)	European Region	1,315	Di Castelnuovo et al. [139]
Obesity/BMI>40	Hospital mortality	<=50	OR 5.1 (2.3-11.1)	Regions of America: North	572	Klang et al. [193]
Obesity/BMI>40	Hospital mortality	>50	OR 1.6 (1.2-2.3)	Regions of America: North	2,834	Klang et al. [193]
Hyperlipidemia	Hospital mortality	<=50	OR 0.8 (0.3-2.1)	Regions of America: North	572	Klang et al. [193]
Hyperlipidemia	Hospital mortality	>50	OR 1.00 (0.8-1.2)	Regions of America: North	2,834	Klang et al. [193]

CKD, chronic kidney disease; eGFR, Estimated glomerular filtration rate; HR, hazard ratio; OR, odds ratio; RR, risk ratio

2.8. Summary of evidence for specific population groups

Twenty studies[128,133,138,144,149,150,152,154,161,168,198,208,213,227,257,271,272,278-280] in our pool selected populations based on a pre-existing condition and therefore, could not be included in the meta-analysis.

Of those, 12 studies [149,150,152,154,161,168,198,213,271,278-280] included patients with cancer and estimated risk factors for the COVID-19 outcomes considered in this review. Among these twelve studies, seven studies included cancer patients,[150,154,161,198,213,271,279] others selected groups of cancer patients: two studies focused cancer patients receiving active treatment[149,168], and one each, patients with haematological malignancy[152], patients with chronic lymphocytic leukemia[280], and patients with thoracic malignancies[278].

Other specific populations are patients with IBD[128], hypertensive patients[138,272], patients with diabetes [133,257], hemodialysis patients[144], intubated patients[227], and patients who receive anticoagulants[208].

In this section, we report only statistically significant risk estimates.

Patients with cancer

Seven studies included patients with cancer who were tested positive for SARS-CoV-2. Pinato et al.[154] estimated an increased risk for death among cancer patients who had additionally two and more other concurrent diseases (HR 1.47 (CI 1.13-1.92)) or who had active malignancy (HR 1.81 (CI 1.35-2.44)) compared with patients with cancer in remission (or no measurable disease). Evidence provided by Kuderer et al.[279] also shows an increased risk of death with an increasing number of comorbidities in cancer patients (OR 4.5 (1.33-15.28) - OR 5.04 (1.42-17.93)). However, the evidence is not consistent for >4 comorbidities. Considerably high risk for death was reported in cancer patients with progressive disease comparing with those in remission (OR 5.2 (2.77-9.77)). Others indicated that among the patients with cancer and COVID-19, the patients with haematologic malignancy at higher risk for hospitalisation (OR 2.49 (1.35-4.67))[213] and death (OR 2.25 (1.13-4.57))[150].

Among the types of hematologic cancer, SARS-CoV-2-positive patients with leukaemia had a higher risk for mortality (OR 2.25 (1.13–4.57)) (compared with patients with non-colorectal cancer of digestive organs)[150]. There was no significant association with mortality for other types of hematologic cancer in this study. Among the patients with hematologic malignancy, Passamonti et al.[152] reported an increased risk for death in patients with progressive status (OR 2.1 (CI 1.41-3.12)), acute myeloid leukaemia (OR 3.49 (CI 1.56-7.81)), indolent lymphomas (OR 2.19 (CI 1.07-4.48)), aggressive lymphomas (OR 2.56 (CI 1.34-4.89)), and plasma cell neoplasms (OR 2.48 (CI 1.31-4.69)).

Among comorbidities, asthma was associated with death (HR 2.5 (1.1-5.8)) in patients with chronic lymphocytic leukaemia[280].

Patients with Inflammatory Bowel Disease (IBD)

Bezzio et al.[128] reported an increased risk of case mortality among IBD patients with a Charlson Comorbidity Index Score>1 (OR 16.66 (1.8-153.9)) or those who had any IBD activity (OR 8.45 (1.26-56.56)).

Patients with Hypertension

Conversano et al.[138] suggested an increased risk of in-hospital mortality for SARS-CoV-2-positive patients suffering from hypertension with the pre-existing condition chronic heart failure (OR2.8 (1.1-6.9)).

Hemodialysis patients

Goicoechea et al.[144] reported an increased risk of in-hospital mortality for SARS-CoV-2-positive hemodialysis patients with longer dialysis vintage per month on hemodialysis (OR 1.008 (1.001-1.015)).

Patients with diabetes

Shi Q et al.[257] found that the risk for in-hospital mortality was increased for SARS-CoV-2-positive diabetes patients with the pre-existing condition hypertension (OR 3.10 (1.14-8.44)).

2.9. Risk of bias evaluation

Table 12 Results of risk of bias evaluation with the Newcastle-Ottawa Scale (NOS) (n=17)

Study	Selection 1 representativeness of the exposed cohort	Selection 2 selection of the non exposed cohort	Selection 3 ascertainment of exposure	Selection 4 demonstration that outcome of interest was not present at start of study meant is not death but the disease incident	Comparability 1 on the basis of the design study controls for	Outcome 1 assessment of outcome	Outcome 2 follow up long enough for outcomes to occur	Outcome 3 adequacy of follow up of cohorts	TOTAL
Rossi, A; Italy[158]	1b (1 point): somewhat representative since patients were consecutively included into study (ICU admission); but single center	2a (1 point): drawn from the same community as the exposed cohort	3a (1point): secure record of the exposure	4a (1 point): no doubt about temporal order between exposure and outcome	1a (1 point): study controls for age, sex, smoking status, comorbidities (obesity = variable of interest, coronary heart disease, CHF, HTN, DM, COPD, chronic renal failure (CKD or reduced renal function?), Immun, immunodeficiency or immunosuppressed state and CANC)	1b (1point): record linkage (registry data)	2a (1 point): follow up adequate to determine death following COVID-19 (28 day)	3a (1 point): complete follow up	8
Bellan; Italy[126]	1b (1 point): somewhat representative of the average COVID-19 patient admitted to hospital, since these patients were all from Northern Italy and from three centers only; only 486 out of 1697 had complete data available for the analysis, however missing at random is assumed; 79 out of 486 were still in hospital at the end of the observation period; proportion of males / age seems a bit high in comparison to other studies	2a (1 point): drawn from the same community as the exposed cohort	3a (1 point): secure record of the exposure	4a (1 point): no doubt about temporal order between exposure and outcome	1a (1point): study controls for age, CANCact, obesity, smoking	1b (1 point): record linkage	2a (1 point): follow up adequate to determine death following COVID-19	3b (1 point): subjects lost to follow up unlikely to introduce bias	8

Study	Selection 1 representativeness of the exposed cohort	Selection 2 selection of the non exposed cohort	Selection 3 ascertainment of exposure	Selection 4 demonstration that outcome of interest was not present at start of study meant is not death but the disease incident	Comparability 1 on the basis of the design study controls for	Outcome 1 assessment of outcome	Outcome 2 follow up long enough for outcomes to occur	Outcome 3 adequacy of follow up of cohorts	TOTAL
Reilev; Denmark[156]	1a (1 point): truly representative of Danish patients diagnosed with COVID-19 - Danish administrative and health registries allow complete nationwide capture of an unselected cohort of all individuals tested for SARS-CoV-2 without restricting to those treated at hospitals and irrespective of socio-economic difference; long time frame from February til May; however: obesity and dementia, may be under-reported in the Danish hospital and prescription registries,thus causing an underestimation of the prevalence of these specific diseases; prioritized testing of individuals in hospitals	2a (1 point): drawn from the same community as the exposed cohort	3a (1 point): secure record of the exposure	4a (1 point): no doubt about temporal order between exposure and outcome	1a (1 point): age, sex, 1 of the following comorbidities: ART, CANC, CHF, CKD, CLD, CRB, DEM, DM, HTN, IHD, obesity, Organ, Resp, Rheuma	1b (1 point): record linkage	2a (1 point): follow up adequate to determine death following COVID-19 (2 days before the index date and 30 days of follow up were available for the entire cohort)	3a (1 point): complete follow up	8
Merzon; Israel[151]	1b (1 point): somewhat representative since it is a population based study, using a health service database and pulling individuals within a specific time frame, however, it is unclear how many COVID-19 positive patients without info on the exposure were excluded from that sample due to missing data (some had no prior vit D test)- it is stated that 6215 out of 14022 positively and negatively tested subjects had to be excluded due to missing data on plasma 25(OH)D levels (=exposure)	2a (1 point): drawn from the same community as the exposed cohort	3a (1 point): secure record of the exposure	4a (1 point): no doubt about temporal order between exposure and outcome	1a (1 point): study controls for age (<50), sex, comorbidities (Depression, schizophrenia, DEM, DM, HTN, CVD, chronic lung disorder), BMI, smoking, lab values	1b (1 point): record linkage	2a (1 point): follow up adequate to determine hospitalisation following COVID-19	3a (1 point): complete follow up	8
Gu, T, Mack; USA[181]	1b (1 point): somewhat representative since all patients tested for COVID-19 at univeristy of michigan medicine were selected within certain time frame; single center;	2a (1 point): drawn from the same community as the exposed cohort	3a (1 point): secure record of the exposure	4a (1 point): no doubt about temporal order between exposure and outcome	1b (1 point): model 1) age, race/ethnicity, sex, 2) adjustment (1)+SES, and 3) adjustment (2)+comorbidity score	1b (1 point): record linkage	2a (1 points): cases from March 10, April 22, follow up until July	3a (1 point): complete follow up	8

Study	Selection 1 representativeness of the exposed cohort	Selection 2 selection of the non exposed cohort	Selection 3 ascertainment of exposure	Selection 4 demonstration that outcome of interest was not present at start of study meant is not death but the disease incident	Comparability 1 on the basis of the design study controls for	Outcome 1 assessment of outcome	Outcome 2 follow up long enough for outcomes to occur	Outcome 3 adequacy of follow up of cohorts	TOTAL
Giannouchos; Mexico[233]	1a (1 point): truly representative of COVID-19 positive patients that were reported to a nation-level dataset (Mexico)	2a (1 point): drawn from the same community as the exposed cohort	3a (1 point): secure record of the exposure	4a (1 point): no doubt about temporal order between exposure and outcome	1b (1 point): age, sex, Mexican nationality, smoking, comorbidities (obesity, DM, HTN, Immun, CVD, AST, COPD, CRD	1b (1 point): record linkage	2a (1 point): follow up adequate to determine outcomes following COVID-19	3a (1 point): complete follow up	8
Denova-Gutiérrez; Mexico[231]	1a (1 point): truly representative of COVID-19 positive patients that may develop severe adverse COVID-19-related outcomes at hospital admission; nationwide data from hospital surveillance and sentinel surveillance	2a (1 point): drawn from the same community as the exposed cohort	3a (1 point): secure record of the exposure	4a (1 point): no doubt about temporal order between exposure and outcome	1b (1 point): adjusted for age, sex, smoking status, obesity history of chronic diseases (HTN, DM, Obesity, CVD, CKD, immunosuppression), place of care, USMER2, and drug treatment	1b (1 point): record linkage	2a (1 point): follow up adequate to determine outcome (it is severe COVID-19 on admission, not death)	3a (1 point): complete follow up (it states that info on hospial admission and status of treatment and admission to ICU were availlable for all cases)	8
Li, J; China[252]	1b (1 point): somewhat representative, since COVID-19 positive inpatients were included from one area only (Wuhan) and from 2 centers only	2a (1 point): drawn from the same community as the exposed cohort	3a (1 point): secure record of the exposure	4a (1 point): no doubt about temporal order between exposure and outcome	1b (1 point): study controls for additional factors besides the most important one: sex, age, comorbidities (DM, malignancy, HTN, coronary heart disease, ART, CVD)	1b (1 point): record linkage	2a (1 point): follow up adequate to determine hospitalisation following COVID-19	3a (1 point): complete follow up; they seem to have chosen all patients in a certain time frame without exlcuding any of them	8

Study	Selection 1 representativeness of the exposed cohort	Selection 2 selection of the non exposed cohort	Selection 3 ascertainment of exposure	Selection 4 demonstration that outcome of interest was not present at start of study meant is not death but the disease incident	Comparability 1 on the basis of the design study controls for	Outcome 1 assessment of outcome	Outcome 2 follow up long enough for outcomes to occur	Outcome 3 adequacy of follow up of cohorts	TOTAL
Xu, J; China[266]	1b (1 point): somewhat representative, since COVID-19 positive inpatients were included from one area only (Wuhan) and from 3 centers only; patients that died within 48 hours were excluded (because their durations in ICUs were too short to reveal the effectiveness of treatments receied in ICUs and to eliminate the bias on data collection of organ function or complications)	2a (1 point): drawn from the same community as the exposed cohort	3a (1 point): secure record of the exposure by medical records	4a (1 point): no doubt about temporal order between exposure and outcome	1b (1 point): age, comorbidities (malignancy, liver dsyfunction), lab values, complications	1c (0 points): patients discharged were called to record their living status	2a (1 point): follow up adequate to determine hospitalisation following COVID-19 (2 months)	3a (1 point): follow up was complete	7
Tai; China[261]	1b (1 point): somewhat representative, since COVID-19 positive inpatients were included from one area (Changsha) and from 1 center only; 43 patients out of 394 patients had no available medial information or duplicated records and were excluded (we can assume missing at random?)	2a (1 point): drawn from the same community as the exposed cohort	3a (1 point): secure record of the exposure by medical records	4a (1 point): no doubt about temporal order between exposure and outcome	1b (1 point): age, sex, comorbidities (CVD, DM, lung diseases), chest tightness	1b (1 point): record linkage	2a (1 point): follow up adequate to determine discharge alive and referral to the designated hospital for intensive care, minimum 15 days	3a (1 point): follow up was complete for sample (referral and discharge could be evaluated by the hospital itself)	8
Sy; Philippines[260]	1a (1 point): truly representative, all reported COVID -19 cases in the Phillipines as of May 2020 were included in the study	2a (1 point): drawn from the same community as the exposed cohort	3b (1 point): structured interviews by the physician or nurse to fill out form on exposure (TB)	4a (1 point): no doubt about temporal order between exposure and outcome	1b (1 point): matching design with age, sex, and other comorbid conditions	1b (1 point): record linkage, outcomes reported by the Department of Health	2a (1 point): yes, minimum 1 month, sufficient to assess death and recovery	3a (1 point): adequate follow up; no cases were lost to follow up (some cases had covariates missing, however missing at random)	8

Study	Selection 1 representativeness of the exposed cohort	Selection 2 selection of the non exposed cohort	Selection 3 ascertainment of exposure	Selection 4 demonstration that outcome of interest was not present at start of study meant is not death but the disease incident	Comparability 1 on the basis of the design study controls for	Outcome 1 assessment of outcome	Outcome 2 follow up long enough for outcomes to occur	Outcome 3 adequacy of follow up of cohorts	TOTAL
Al-Salameh; France[124]	1b (1 point): somewhat representative, adult patients with confirmed COVID-19 consecutively admitted to hospital, however single center	2a (1 point): drawn from the same community as the exposed cohort	3a (1 point): secure record of the exposure	4a (1 point): no doubt about temporal order between exposure and outcome	1b (1 point): model ICU admission: age, sex, BMI and comorbidities (DM), lab value; model in-hospital mortality: age, sex, lab values, comorbidities (DM, CVD), lab value	1b (1 point): record linkage	2b (0 points): cases were included until April 21st, censoring took place on May 1st, there were 47 still hospitalised; sample sizes used in the models were admission ICU=326 and hospital mortality 370; they say 433 initially were included	2c (0 points): 433 individuals were included, the number used in the model is N (ICU admission=326) and N (hospital mortality = 370) - they state 47 were still hospitalised at the time of censoring - follow up rate is <80%	6
Conversano; Italy[138]	1a (1 point): somewhat representative, because patients consecutively admitted to hospital with COVID-19 within certain time frame were from single center and one area in Italy; 21 were excluded due to incomplete data and lost ot follow up (no specification how many lost due to one or the other)	2a (1 point): drawn from the same community as the exposed cohort	3a (1 point): secure record of the exposure	4a (1 point): no doubt about temporal order between exposure and outcome	1b (1 point): model 1: age, Comorbidities (HTN, CHF, DM), ACE inhibitor/ARBs; model 2: age, Comorbidities (CKD, COPD, CANC), ?-blocker	1b (1 point): record linkage	2a (1 point): yes, because the median follow up was 28 with a range of 21-32	2b (1 point): 21 out of 212 were lost due to incomplete data or follow up, nos specification here. If we assume worst case that 20 are lost due to follow up, then that would translate to a nr lost of 9,4%	8

Study	Selection 1 representativeness of the exposed cohort	Selection 2 selection of the non exposed cohort	Selection 3 ascertainment of exposure	Selection 4 demonstration that outcome of interest was not present at start of study meant is not death but the disease incident	Comparability 1 on the basis of the design study controls for	Outcome 1 assessment of outcome	Outcome 2 follow up long enough for outcomes to occur	Outcome 3 adequacy of follow up of cohorts	TOTAL
Kim, M; Korea[250]	1b (1 point): somewhat representative, multi center study, 5 centers in Daegu, in Korea; however, HbA1c values for a considerable proportion of the participants were missing; also 65.6% of the confirmed cases of COVID-19 in Daegu were associated with a single religious group. so as to the authors it is unlikely that the data collected are representative of the general population	2a (1 point): drawn from the same community as the exposed cohort	3a (1 point): secure record of the exposure	4a (1 point): no doubt about temporal order between exposure and outcome	1b (1point): age, sex, comorbidities (DM, HTN, CRD, CVD, CKD, Resp, CANC)	1b (1 point): record linkage	2a (1 point): authors make a statement about the number of deaths in the entire cohort, however the follow up period is not stated	2a (1 point): all subjects in the study were accounted for	8
Singh (a); USA[218]	1a (1 point): truly representative; multiple large healthcare organizations in United States.identified COVID-19 patients; files from individuals Jan til May, long time period	2a (1 point): drawn from the same community as the exposed cohort	3a (1 point): secure record of the exposure	4a (1 point): no doubt about temporal order between exposure and outcome	1b (1 point): age, race, ethnicity, comorbidities used for matching; comorbidity of interest in analysis: obesity	1b (1 point): record linkage	2a (1 point): outcomes were assessed from diagnosis to 30 days after the index event	2a (1 point): all subjects in the study were accounted for	8
Zhu; China[277]	1a (1 point): truly representative of patient with COVID-19 that was admitted to hospital that would either have or have not type 2 diabetes, multi center in Hubei Province, no systematic missing of cases	2a (1 point): drawn from the same community as the exposed cohort	3a (1 point): secure record of the exposure	4a (1 point): no doubt about temporal order between exposure and outcome	1b (1 point): age, sex, comorbidities (DM), hospital site	1b (1 point): record linkage	2a (1 point): 28 day follow up after admission	2a (1 point): all subjects in the study were accounted for	8
Antwi-Amoabeng; USA[169]	1b (1 point): somewhat representative, single center in USA, Nevada, all patients tested positive between 12. Mar-8. Mar were included	2a (1 point): drawn from the same community as the exposed cohort	3a (1 point): secure record of the exposure	4a (1 point): no doubt about temporal order between exposure and outcome	1b (1 point): age, sex, ethnicity, comorbidities (DM, HTN, obesity, CKD, COPD), ICU stay	1b (1 point): record linkage	2a (1 point): follow up is not mentioned, however, the outcomes for all included cases were recorded	2a (1 point): all subjects in the study were accounted for	8

2.10. Systematic reviews excluded after eligibility assessment

Pre-print duplication:

1. Katzenschlager S, Zimmer A, Gottschalk C, et al. Can we predict the severe course of COVID-19 – a systematic review and meta-analysis of indicators of clinical outcome?; 2020.
2. Parohan M, Yaghoubi S, Seraji A, Javanbakht MH, Sarraf P, Djalali M. Risk factors for mortality in patients with Coronavirus disease 2019 (COVID-19) infection: a systematic review and meta-analysis of observational studies. medRxiv 2020: 2020.04.09.20056291.
3. Salunke A, Nandy K, Pathak S, et al. Coronavirus Disease (COVID-19) in Cancer Patients: A Systematic Review and Meta-Analysis to Evaluate Severity and Fatal Outcomes. SSRN Electronic Journal 2020.
4. Soeroto AY, Soetedjo NN, Purwiga A, et al. Association of BMI and Obesity with Composite poor outcome in COVID-19 adult patients: A Systematic Review and Meta-Analysis. medRxiv 2020: 2020.06.28.20142240.
5. Ssentongo P, Ssentongo AE, Heilbrunn ES, Ba DM, Chinchilli VM. The association of cardiovascular disease and other pre-existing comorbidities with COVID-19 mortality: A systematic review and meta-analysis. medRxiv 2020: 2020.05.10.20097253.

No citations for included primary studies:

1. Fang X, Li S, Yu H, Wang P, Zhang Y, Chen Z, et al. Epidemiological, comorbidity factors with severity and prognosis of COVID-19: a systematic review and meta-analysis. Aging. 2020;12(13):12493-503
2. Pranata R, Huang I, Lim MA, Wahjoepramono EJ, July J. Impact of cerebrovascular and cardiovascular diseases on mortality and severity of COVID-19–systematic review, meta-analysis, and meta-regression. Journal of Stroke and Cerebrovascular Diseases. 2020;29(8):104949
3. Huang I, Lim MA, Pranata R. Diabetes mellitus is associated with increased mortality and severity of disease in COVID-19 pneumonia – A systematic review, meta-analysis, and meta-regression. Diabetes & Metabolic Syndrome: Clinical Research & Reviews. 2020;14(4):395-403
4. Seidu S, Gillies C, Zaccardi F, Kunutsor SK, Hartmann-Boyce J, Yates T, et al. The impact of obesity on severe disease and mortality in people with SARS-CoV-2: A systematic review and meta-analysis. Endocrinology, Diabetes & Metabolism. 2021;4(1):e00176

Citations do not match given references for primary studies:

1. Varikasuvu SR, Dutt N, Thangappazham B, Varshney S. Diabetes and COVID-19: A pooled analysis related to disease severity and mortality. Primary Care Diabetes. 2021;15(1):24-7.
2. Hussain A, Mahawar K, Xia Z, Yang W, El-Hasani S.: Obesity and mortality of COVID-19. Meta-analysis. Obesity Research & Clinical Practice. 2020;14(4):295-300. Currently retracted

Estimates of prevalence:

1. Kulkarni AV, Kumar P, Tevethia HV, Premkumar M, Arab JP, Candia R, et al. Systematic review with meta-analysis: liver manifestations and outcomes in COVID-19. Alimentary pharmacology & therapeutics. 2020;52(4):584-99.

Full-text was not retrievable:

1. Plasencia-Urizarri, T. M., Aguilera-Rodríguez, R. and Almaguer-Mederos, L. E. [Comorbidities and clinical severity of COVID-19: systematic review and meta-analysis]

2.11. Excluded primary studies

Excluded based on the outcome

Composite outcome: severe disease (n=36)

1. Cai Q, Chen F, Wang T, Luo F, Liu X, Wu Q, et al. Obesity and COVID-19 Severity in a Designated Hospital in Shenzhen, China. Diabetes care. 2020.
2. Cai Q, Huang D, Ou P, Yu H, Zhu Z, Xia Z, et al. 2019-nCoV Pneumonia in a Normal Work Infectious Diseases Hospital Besides Hubei Province, China. SSRN. 2020.
3. Cai Q, Huang D, Ou P, Yu H, Zhu Z, Xia Z, et al. COVID-19 in a Designated Infectious Diseases Hospital Outside Hubei Province, China. Allergy. 2020.
4. Cen Y, Chen X, Shen Y, Zhang XH, Lei Y, Xu C, et al. Risk factors for disease progression in patients with mild to moderate coronavirus disease 2019—a multi-centre observational study. Clinical Microbiology and Infection. 2020;26(9):1242-7.
5. Chung SM, Lee YY, Ha E, Yoon JS, Won KC, Lee HW, et al. The Risk of Diabetes on Clinical Outcomes in Patients with Coronavirus Disease 2019: A Retrospective Cohort Study. Diabetes & metabolism journal. 2020;44(3):405-13.
6. Feng Z, Li J, Yao S, Yu Q, Zhou W, Mao X, et al. The Use of Adjuvant Therapy in Preventing Progression to Severe Pneumonia in Patients with Coronavirus Disease 2019: A Multicenter Data Analysis. medRxiv. 2020:2020.04.08.20057539.
7. Gao F, Zheng KI, Wang XB, Sun QF, Pan KH, Wang TY, et al. Obesity Is a Risk Factor for Greater COVID-19 Severity. Diabetes care. 2020.
8. Gao F, Zheng KI, Wang XB, Yan HD, Sun QF, Pan KH, et al. Metabolic associated fatty liver disease increases COVID-19 disease severity in non-diabetic patients. Journal of gastroenterology and hepatology. 2020.
9. Grandbastien M, Piotin A, Godet J, Abessolo-Amougou I, Ederlé C, Enache I, et al. SARS-CoV-2 Pneumonia in Hospitalized Asthmatic Patients Did Not Induce Severe Exacerbation. J Allergy Clin Immunol Pract. 2020;8(8):2600-7.
10. Hu L, Chen S, Fu Y, Gao Z, Long H, Wang JM, et al. Risk Factors Associated with Clinical Outcomes in 323 COVID-19 Hospitalized Patients in Wuhan, China. Clinical infectious diseases: an official publication of the Infectious Diseases Society of America. 2020
11. Huang R, Zhu L, Xue L, Liu L, Yan X, Wang J, et al. Clinical findings of patients with coronavirus disease 2019 in Jiangsu province, China: A retrospective, multi-center study. PLoS neglected tropical diseases. 2020;14(5):e0008280.
12. Hultcrantz M, Richter J, Rosenbaum C, Patel D, Smith E, Korde N, et al. COVID-19 infections and outcomes in patients with multiple myeloma in New York City: a cohort study from five academic centers. medRxiv : the preprint server for health sciences. 2020:2020.06.09.20126516.
13. Jang JG, Hur J, Choi EY, Hong KS, Lee W, Ahn JH. Prognostic Factors for Severe Coronavirus Disease 2019 in Daegu, Korea. J Korean Med Sci. 2020;35(23):e209-e.
14. Jee J, Foote MB, Lumish M, Stonestrom AJ, Wills B, Narendra V, et al. Chemotherapy and COVID-19 Outcomes in Patients With Cancer. J Clin Oncol. 2020;38(30):3538-46.
15. Ji W, Huh K, Kang M, Hong J, Bae GH, Lee R, et al. Effect of Underlying Comorbidities on the Infection and Severity of COVID-19 in Korea: a Nationwide Case-Control Study. J Korean Med Sci. 2020;35(25):e237-e.
16. Liang W, Guan W, Chen R, Wang W, Li J, Xu K, et al. Cancer patients in SARS-CoV-2 infection: a nationwide analysis in China. The Lancet Oncology. 2020.
17. Liang W, Liang H, Ou L, Chen B, Chen A, Li C, et al. Development and Validation of a Clinical Risk Score to Predict the Occurrence of Critical Illness in Hospitalized Patients With COVID-19. JAMA internal medicine. 2020;180(8):1081-9.
18. Liu X, Zhou H, Zhou Y, Wu X, Zhao Y, Lu Y, et al. Risk factors associated with disease severity and length of hospital stay in COVID-19 patients. Journal of Infection. 2020;81(1):e95-e7.
19. Romero-Sánchez CM, Díaz-Maroto I, Fernández-Díaz E, Sánchez-Larsen Á, Layos-Romero A, García-García J, et al. Neurologic manifestations in hospitalized patients with COVID-19: The ALBACOVID registry. Neurology. 2020;95(8):e1060-e70.
20. Scarfò L, Chatzikonstantinou T, Rigolin GM, Quaresmini G, Motta M, Vitale C, et al. COVID-19 severity and mortality in patients with chronic lymphocytic leukemia: a joint study by ERIC, the European Research Initiative on CLL, and CLL Campus. Leukemia. 2020;34:1-10.
21. Schalekamp S, Huisman M, van Dijk RA, Boomsma MF, Freire Jorge PJ, de Boer WS, et al. Model-based Prediction of Critical Illness in Hospitalized Patients with COVID-19. Radiology. 2020;0(0):202723.

22. Shi Y, Yu X, Zhao H, Wang H, Zhao R, Sheng J. Host susceptibility to severe COVID-19 and establishment of a host risk score: findings of 487 cases outside Wuhan. Critical care (London, England). 2020;24(1):108.
23. Targher G, Mantovani A, Wang X-B, Yan H-D, Sun Q-F, Pan K-H, et al. Patients with diabetes are at higher risk for severe illness from COVID-19. Diabetes & Metabolism. 2020.
24. Tian J, Yuan X, Xiao J, Zhong Q, Yang C, Liu B, et al. Clinical characteristics and risk factors associated with COVID-19 disease severity in patients with cancer in Wuhan, China: a multicentre, retrospective, cohort study. The Lancet Oncology. 2020;21(7):893-903.
25. Wang G, Wu C, Zhang Q, Wu F, Yu B, Lv J, et al. C-Reactive Protein Level May Predict the Risk of COVID-19 Aggravation. Open Forum Infectious Diseases. 2020;7(5).
26. Wei YY, Wang RR, Zhang DW, Tu YH, Chen CS, Ji S, et al. Risk factors for severe COVID-19: evidence from 167 hospitalized patients in Anhui, China. The Journal of infection. 2020.
27. Yang Y, Ding L, Zou X, Shen Y, Hu D, Hu X, et al. Visceral Adiposity and High Intramuscular Fat Deposition Independently Predict Critical Illness in Patients with SARS-CoV-2. Obesity (Silver Spring). 2020;28(11):2040-8.
28. Yu C, Lei Q, Li W, Wang X, Li W, Liu W. Epidemiological and clinical characteristics of 1663 hospitalized patients infected with COVID-19 in Wuhan, China: a single-center experience. Journal of infection and public health. 2020;13(9):1202-9.
29. Yu X, Sun X, Cui P, Pan H, Lin S, Han R, et al. Epidemiological and Clinical Characteristics of 333 Confirmed Cases with Coronavirus Disease 2019 in Shanghai, China. Transbound Emerg Dis. 2020.
30. Zhang J, Lu S, Wang X, Jia X, Li J, Lei H, et al. Do underlying cardiovascular diseases have any impact on hospitalised patients with COVID-19? Heart (British Cardiac Society). 2020;106(15):1148-53.
31. Zhang L, Zhu F, Xie L, Wang C, Wang J, Chen R, et al. Clinical characteristics of COVID-19-infected cancer patients: a retrospective case study in three hospitals within Wuhan, China. Annals of oncology : official journal of the European Society for Medical Oncology. 2020;31(7):894-901.
32. Zhang S-Y, Lian J-S, Hu J-H, Zhang X-L, Lu Y-F, Cai H, et al. Clinical characteristics of different subtypes and risk factors for the severity of illness in patients with COVID-19 in Zhejiang, China. Infectious Diseases of Poverty. 2020;9(1):85.
33. Zhang YT, Deng AP, Hu T, Chen XG, Zhuang YL, Tan XH, et al. Clinical outcomes of COVID-19 cases and influencing factors in Guangdong province. Zhonghua liu xing bing xue za zhi = Zhonghua liuxingbingxue zazhi. 2020;41(0):E057.
34. Zheng KI, Gao F, Wang X-B, Sun Q-F, Pan K-H, Wang T-Y, et al. Obesity as a risk factor for greater severity of COVID-19 in patients with metabolic associated fatty liver disease. Metabolism. 2020:154244.
35. Zhou Y-J, Zheng KI, Wang X-B, Sun Q-F, Pan K-H, Wang T-Y, et al. Metabolic-associated fatty liver disease is associated with severity of COVID-19. Liver International. 2020;40(9):2160-3.
36. Zhou Y-J, Zheng KI, Wang X-B, Yan H-D, Sun Q-F, Pan K-H, et al. Younger patients with MAFLD are at increased risk of severe COVID-19 illness: A multicenter preliminary analysis. Journal of hepatology. 2020;73(3):719-21.

Outcome: incidence or a positive test result (n=16)

1. Alam MR, Kabir MR, Reza S. Comorbidities might be a risk factor for the incidence of COVID-19: Evidence from a web-based survey of 780,961 participants. medRxiv. 2020:2020.06.22.20137422.
2. Cho ER, Slutsky AS, Jha P. Smoking and the risk of COVID-19 infection in the UK Biobank Prospective Study. medRxiv. 2020:2020.05.05.20092445.
3. Darling AL, Ahmadi KR, Ward KA, Harvey NC, Couto Alves A, Dunn-Waters DK, et al. Vitamin D status, body mass index, ethnicity and COVID-19: Initial analysis of the first-reported UK Biobank COVID-19 positive cases (n 580) compared with negative controls (n 723). medRxiv. 2020:2020.04.29.20084277.
4. de Lusignan S, Dorward J, Correa A, Jones N, Akinyemi O, Amirthalingam G, et al. Risk factors for SARS-CoV-2 among patients in the Oxford Royal College of General Practitioners Research and Surveillance Centre primary care network: a cross-sectional study. Lancet Infect Dis. 2020.
5. Gubatan J, Levitte S, Balabanis T, Patel A, Sharma A, Habtezion A. SARS-CoV-2 Testing, Prevalence, and Predictors of COVID-19 in Patients with Inflammatory Bowel Disease in Northern California. Gastroenterology. 2020;159(3):1141-4.e2.
6. Hernández-Garduño E. Obesity is the comorbidity more strongly associated for Covid-19 in Mexico. A case-control study. Obesity Research & Clinical Practice. 2020;14(4):375-9.

7. Ho FK, Celis-Morales CA, Gray SR, Katikireddi SV, Niedzwiedz CL, Hastie C, et al. Modifiable and non-modifiable risk factors for COVID-19, and comparison to risk factors for influenza and pneumonia: results from a UK Biobank prospective cohort study. BMJ Open. 2020;10(11):e040402.
8. Huang J, Xie N, Hu X, Yan H, Ding J, Liu P, et al. Epidemiological, virological and serological features of COVID-19 cases in people living with HIV in Wuhan City: A population-based cohort study. Clinical infectious diseases : an official publication of the Infectious Diseases Society of America. 2020.
9. Leung NY, Bulterys MA, Bulterys PL. Predictors of COVID-19 incidence, mortality, and epidemic growth rate at the country level. medRxiv. 2020:2020.05.15.20101097.
10. Martin CA, Jenkins DR, Minhas JS, Gray LJ, Tang J, Williams C, et al. Socio-demographic heterogeneity in the prevalence of COVID-19 during lockdown is associated with ethnicity and household size: Results from an observational cohort study. EClinicalMedicine. 2020;25:100466.
11. Prats-Uribe A, Paredes R, Prieto-Alhambra D. Ethnicity, comorbidity, socioeconomic status, and their associations with COVID-19 infection in England: a cohort analysis of UK Biobank data. medRxiv. 2020:2020.05.06.20092676.
12. Raisi-Estabragh Z, McCracken C, Ardissino M, Bethell M, Cooper J, Cooper C, et al. Non-white ethnicity, male sex and higher body mass index, but not medication acting on the renin-angiotensin system are associated with coronavirus disease 2019 (COVID-19) hospitalisation: review of the first 669 cases from the UK bio bank. MedRxiv. 2020.
13. Rozenfeld Y, Beam J, Maier H, Haggerson W, Boudreau K, Carlson J, et al. A model of disparities: risk factors associated with COVID-19 infection. Int J Equity Health. 2020;19(1):126.
14. Sakowicz A, Ayala AE, Ukeje CC, Witting CS, Grobman WA, Miller ES. Risk factors for severe acute respiratory syndrome coronavirus 2 infection in pregnant women. American journal of obstetrics & gynecology MFM. 2020;2(4):100198.
15. Taxonera C, Sagastagoitia I, Alba C, Mañas N, Olivares D, Rey E. 2019 novel coronavirus disease (COVID-19) in patients with inflammatory bowel diseases. Alimentary pharmacology & therapeutics. 2020;52(2):276-83.
16. Vahidy FS, Nicolas JC, Meeks JR, Khan O, Pan A, Jones SL, et al. Racial and ethnic disparities in SARS-CoV-2 pandemic: analysis of a COVID-19 observational registry for a diverse US metropolitan population. BMJ Open. 2020;10(8):e039849

Composite outcome: ICU or death (n=9)

1. Almazeedi S, Al-Youha S, Jamal MH, Al-Haddad M, Al-Muhaini A, Al-Ghimlas F, et al. Characteristics, risk factors and outcomes among the first consecutive 1096 patients diagnosed with COVID-19 in Kuwait. EClinicalMedicine. 2020;24.
2. Antonio-Villa NE, Bello-Chavolla OY, Vargas-Vázquez A, Fermín-Martínez CA, Márquez-Salinas A, Bahena-López JP. Health-care workers with COVID-19 living in Mexico City: clinical characterization and related outcomes. Clinical infectious diseases : an official publication of the Infectious Diseases Society of America. 2020.
3. Cecconi M, Piovani D, Brunetta E, Aghemo A, Greco M, Ciccarelli M, et al. Early Predictors of Clinical Deterioration in a Cohort of 239 Patients Hospitalized for Covid-19 Infection in Lombardy, Italy. Journal of clinical medicine. 2020;9(5).
4. Colombi D, Bodini FC, Petrini M, Maffi G, Morelli N, Milanese G, et al. Well-aerated Lung on Admitting Chest CT to Predict Adverse Outcome in COVID-19 Pneumonia. Radiology. 2020:201433.
5. Feng X, Li P, Ma L, Liang H, Lei J, Li W, et al. Clinical Characteristics and Short-Term Outcomes of Severe Patients With COVID-19 in Wuhan, China. Frontiers in medicine. 2020;7:491.
6. Guan WJ, Liang WH, Zhao Y, Liang HR, Chen ZS, Li YM, et al. Comorbidity and its impact on 1590 patients with Covid-19 in China: A Nationwide Analysis. The European respiratory journal. 2020.
7. Hajifathalian K, Kumar S, Newberry C, Shah S, Fortune B, Krisko T, et al. Obesity is associated with worse outcomes in COVID-19: Analysis of Early Data From New York City. Obesity (Silver Spring). 2020.
8. Kaeuffer C, Le Hyaric C, Fabacher T, Mootien J, Dervieux B, Ruch Y, et al. Risk Factors Associated with Severe COVID-19 in Eastern France: Analysis of 1045 Cases. SSRN. 2020.
9. Piano S, Dalbeni A, Vettore E, Benfaremo D, Mattioli M, Gambino CG, et al. Abnormal liver function tests predict transfer to intensive care unit and death in COVID-19. Liver international : official journal of the International Association for the Study of the Liver. 2020;40(10):2394-406.

Outcome: other (n=28)

Composite: COVID-19 progression

1. Choi MH, Ahn H, Ryu HS, Kim B-J, Jang J, Jung M, et al. Clinical Characteristics and Disease Progression in Early-Stage COVID-19 Patients in South Korea. Journal of clinical medicine. 2020;9(6):1959.
2. Ji D, Qin E, Xu J, Zhang D, Cheng G, Wang Y, et al. Non-alcoholic fatty liver diseases in patients with COVID-19: A retrospective study. Journal of hepatology. 2020;73(2):451-3.

Composite: ICU or intubation

3. Lighter J, Phillips M, Hochman S, Sterling S, Johnson D, Francois F, et al. Obesity in patients younger than 60 years is a risk factor for Covid-19 hospital admission. Clinical infectious diseases : an official publication of the Infectious Diseases Society of America. 2020.

Composite: ICU or intubation or death

4. Xiong TY, Huang FY, Liu Q, Peng Y, Xu YN, Wei JF, et al. Hypertension is a risk factor for adverse outcomes in patients with coronavirus disease 2019: a cohort study. Annals of medicine. 2020;52(7):361-6.
5. Yan X, Wang C, Peng D, Han X, Fan Y, Fang Z, et al. Clinical Features, Treatment and Outcomes of 218 Patients with COVID-19: A Retrospective, Multicenter Study Based on Clinical Classification. Treatment and Outcomes of. 2020;218
6. Ye C, Zhang S, Zhang X, Cai H, Gu J, Lian J, et al. Impact of comorbidities on patients with COVID-19: A large retrospective study in Zhejiang, China. Journal of medical virology. 2020.
7. Zhang Y, Li H, Zhang J, Cao Y, Zhao X, Yu N, et al. The clinical characteristics and outcomes of patients with diabetes and secondary hyperglycaemia with coronavirus disease 2019: A single-centre, retrospective, observational study in Wuhan. Diabetes, obesity & metabolism. 2020;22(8):1443-54.

Complications

8. Yanover C, Mizrahi B, Kalkstein N, Marcus K, Akiva P, Barer Y, et al. What Factors Increase the Risk of Complications in SARS-CoV-2-Infected Patients? A Cohort Study in a Nationwide Israeli Health Organization. JMIR Public Health Surveill. 2020;6(3):e20872-e.

Myocardial injury:

9. Chen C, Chen C, Yan JT, Zhou N, Zhao JP, Wang DW. Analysis of myocardial injury in patients with COVID-19 and association between concomitant cardiovascular diseases and severity of COVID-19. Zhonghua xin xue guan bing za zhi. 2020;48(0):E008.
10. Shi S, Qin M, Cai Y, Liu T, Shen B, Yang F, et al. Characteristics and clinical significance of myocardial injury in patients with severe coronavirus disease 2019. European heart journal. 2020;41(22):2070-9.
11. Wei JF, Huang FY, Xiong TY, Liu Q, Chen H, Wang H, et al. Acute myocardial injury is common in patients with COVID-19 and impairs their prognosis. Heart (British Cardiac Society). 2020;106(15):1154-9.

Lymphocyte reduction

12. Fei J, Fu L, Li Y, Xiang H-X, Xiang Y, Li M-D, et al. Reduction of lymphocyte at early stage elevates severity and death risk of COVID-19 patients: a hospital-based case-cohort study. medRxiv. 2020:2020.04.02.20050955.

Cholestasis; Hypoproteinemia; Hepatocellular injury

13. Fu L, Fei J, Xu S, Xiang H-X, Xiang Y, Tan Z-X, et al. Acute liver injury and its association with death risk of patients with COVID-19: a hospital-based prospective case-cohort study. medRxiv. 2020:2020.04.02.20050997.

Gastrointestinal and Hepatic Manifestations:

14. Hajifathalian K, Krisko T, Mehta A, Kumar S, Schwartz R, Fortune B, et al. Gastrointestinal and Hepatic Manifestations of 2019 Novel Coronavirus Disease in a Large Cohort of Infected Patients From New York: Clinical Implications. Gastroenterology. 2020;159(3):1137-40.e2.

Acute kidney injury

15. Hirsch JS, Ng JH, Ross DW, Sharma P, Shah HH, Barnett RL, et al. Acute kidney injury in patients hospitalized with COVID-19. Kidney international. 2020;98(1):209-18.
16. Xu S, Fu L, Fei J, Xiang H-X, Xiang Y, Tan Z-X, et al. Acute kidney injury at early stage as a negative prognostic indicator of patients with COVID-19: a hospital-based retrospective analysis. medRxiv. 2020:2020.03.24.20042408.

Venous thromboembolism

17. Middeldorp S, Coppens M, van Haaps TF, Foppen M, Vlaar AP, Müller MCA, et al. Incidence of venous thromboembolism in hospitalized patients with COVID-19. Journal of thrombosis and haemostasis : JTH. 2020;18(8):1995-2002.

Refractory COVID-19 pneumonia

18. Mo P, Xing Y, Xiao Y, Deng L, Zhao Q, Wang H, et al. Clinical characteristics of refractory COVID-19 pneumonia in Wuhan, China. Clinical Infectious Diseases. 2020.

COVID-19 pneumonia

19. Pongpirul WA, Wiboonchutikul S, Charoenpong L, Panitantum N, Vachiraphan A, Uttayamakul S, et al. Clinical course and potential predictive factors for pneumonia of adult patients with Coronavirus Disease 2019 (COVID-19): A retrospective observational analysis of 193 confirmed cases in Thailand. PLoS neglected tropical diseases. 2020;14(10):e0008806-e.

ARDS

20. Yu T, Cai S, Zheng Z, Cai X, Liu Y, Yin S, et al. Association Between Clinical Manifestations and Prognosis in Patients with COVID-19. Clinical therapeutics. 2020.

Proteinuria remission

21. Pei G, Zhang Z, Peng J, Liu L, Zhang C, Yu C, et al. Renal Involvement and Early Prognosis in Patients with COVID-19 Pneumonia. Journal of the American Society of Nephrology. 2020;31(6):1157-65.

Prevalence estimates

22. Rugge M, Zorzi M, Guzzinati S. SARS-CoV-2 infection in the Italian Veneto region: adverse outcomes in patients with cancer. Nature Cancer. 2020;1(8):784-8.

Association between tocilizumab and survival

23. Somers EC, Eschenauer GA, Troost JP, Golob JL, Gandhi TN, Wang L, et al. Tocilizumab for treatment of mechanically ventilated patients with COVID-19. Clinical infectious diseases : an official publication of the Infectious Diseases Society of America. 2020.

Chest Radiograph Severity Score

24. Toussie D, Voutsinas N, Finkelstein M, Cedillo MA, Manna S, Maron SZ, et al. Clinical and Chest Radiography Features Determine Patient Outcomes in Young and Middle-aged Adults with COVID-19. Radiology. 2020;297(1):E197-e206.

Olfactory loss

25. Yan CH, Faraji F, Prajapati DP, Ostrander BT, DeConde AS. Self-reported olfactory loss associates with outpatient clinical course in Covid-19. International forum of allergy & rhinology. 2020.

Discharge within two weeks

26. Zhao W, Yu S, Zha X, Wang N, Pang Q, Li D, et al. Clinical characteristics and durations of hospitalized patients with COVID-19 in Beijing: a retrospective cohort study. medRxiv. 2020:2020.03.13.20035436

Transfer to high-level hospital and clinical death

27. Zhou X, Zhu J, Xu T. Clinical characteristics of coronavirus disease 2019 (COVID-19) patients with hypertension on renin–angiotensin system inhibitors. Clinical and Experimental Hypertension. 2020;42(7):656-60.

Death during admission to hospital

28. Yang K, Sheng Y, Huang C, Jin Y, Xiong N, Jiang K, et al. Clinical characteristics, outcomes, and risk factors for mortality in patients with cancer and COVID-19 in Hubei, China: a multicentre, retrospective, cohort study. The Lancet Oncology. 2020;21(7):904-13.

Excluded based on the study population or a risk factor:

Irrespective of SARS-CoV-2 status (n=9)

1. Atkins JL, Masoli JAH, Delgado J, Pilling LC, Kuo C-L, Kuchel GA, et al. Preexisting Comorbidities Predicting COVID-19 and Mortality in the UK Biobank Community Cohort. The Journals of Gerontology: Series A. 2020;75(11):2224-30.
2. Bhaskaran K, Rentsch C, MacKenna B, Schultz A, Mehrkar A, Bates C, et al. HIV infection and COVID-19 death: population-based cohort analysis of UK primary care data and linked national death registrations within the OpenSAFELY platform. medRxiv. 2020:2020.08.07.20169490.
3. Del Amo J, Polo R, Moreno S, Díaz A, Martínez E, Arribas JR, et al. Incidence and Severity of COVID-19 in HIV-Positive Persons Receiving Antiretroviral Therapy : A Cohort Study. Annals of internal medicine. 2020;173(7):536-41.
4. Hamer M, Gale CR, Kivimäki M, Batty GD. Overweight, obesity, and risk of hospitalization for COVID-19: A community-based cohort study of adults in the United Kingdom. Proceedings of the National Academy of Sciences. 2020:202011086.
5. Hamer M, Kivimäki M, Gale CR, Batty GD. Lifestyle risk factors, inflammatory mechanisms, and COVID-19 hospitalization: A community-based cohort study of 387,109 adults in UK. Brain Behav Immun. 2020;87:184-7.
6. Khawaja AP, Warwick AN, Hysi PG, Kastner A, Dick A, Khaw PT, et al. Associations with covid-19 hospitalisation amongst 406,793 adults: the UK Biobank prospective cohort study. medRxiv. 2020:2020.05.06.20092957.
7. Lassale C, Gaye B, Hamer M, Gale CR, Batty GD. Ethnic disparities in hospitalisation for COVID-19 in England: The role of socioeconomic factors, mental health, and inflammatory and pro-inflammatory factors in a community-based cohort study. Brain Behav Immun. 2020;88:44-9.
8. Patel AP, Paranjpe MD, Kathiresan NP, Rivas MA, Khera AV. Race, Socioeconomic Deprivation, and Hospitalization for COVID-19 in English participants of a National Biobank. medRxiv. 2020:2020.04.27.20082107.
9. Williamson EJ, Walker AJ, Bhaskaran K, Bacon S, Bates C, Morton CE, et al. Factors associated with COVID-19-related death using OpenSAFELY. Nature. 2020;584(7821):430-6.

Patients undergoing surgery

1. Nepogodiev D, Glasbey J, Li E, Omar O, Simoes J, Abbott T. Mortality and pulmonary complications in patients undergoing surgery with perioperative SARS-CoV-2 infection: an international cohort study. Lancet (London, England). 2020;396(10243):27-38

Risk: fasting blood glucose

1. Chen Y, Yang D, Cheng B, Chen J, Peng A, Yang C, et al. Clinical Characteristics and Outcomes of Patients With Diabetes and COVID-19 in Association With Glucose-Lowering Medication. Diabetes care. 2020;43(7):1399-407
2. Zhu B, Jiang C, Feng X, Zheng Y, Yang J, Wang F, et al. Correlation between Fasting Blood Glucose Level at Admission and Mortality in COVID-19 Patients: A Retrospective Study. Research Square. 2020

Risk: treatment with Tocilizumab

1. Ramaswamy M, Mannam P, Comer R, Sinclair E, McQuaid DB, Schmidt ML. Off-Label Real World Experience Using Tocilizumab for Patients Hospitalized with COVID-19 Disease in a Regional Community Health System: A Case-Control Study. medRxiv. 2020:2020.05.14.20099234

Risk: visceral fat

1. Battisti S, Pedone C, Napoli N, Russo E, Agnoletti V, Nigra SG, et al. Computed Tomography Highlights Increased Visceral Adiposity Associated With Critical Illness in COVID-19. Diabetes care. 2020;43(10):e129-e30.
2. Watanabe M, Caruso D, Tuccinardi D, Risi R, Zerunian M, Polici M, et al. Visceral fat shows the strongest association with the need of intensive care in patients with COVID-19. Metabolism. 2020;111:154319

Risk: CRP and CD4 in HIV patients

1. Karmen-Tuohy S, Carlucci PM, Zervou FN, Zacharioudakis IM, Rebick G, Klein E, et al. Outcomes Among HIV-Positive Patients Hospitalized With COVID-19. Journal of acquired immune deficiency syndromes (1999). 2020;85(1):6-10.

Simulated population

1. Caramelo F, Ferreira N, Oliveiros B. Estimation of risk factors for COVID-19 mortality - preliminary results. medrxiv. 2020:10.1101.
2. Souza FSH, Hojo-Souza NS, Santos EB, Silva CM, Guidoni DL. Predicting the disease outcome in COVID-19 positive patients through Machine Learning: a retrospective cohort study with Brazilian data. medRxiv. 2020:2020.06.26.20140764.

Other reasons:

Retracted

1. Mehra MR, Desai SS, Kuy S, Henry TD, Patel AN. Cardiovascular Disease, Drug Therapy, and Mortality in Covid-19. New England Journal of Medicine. 2020;382(25):e102.

Lack of reporting

2. Caraballo C, McCullough M, Fuery MA, Chouairi F, Keating C, Ravindra NG, et al. COVID-19 infections and outcomes in a live registry of heart failure patients across an integrated health care system. PloS one. 2020;15(9):e0238829
3. Wang Z, Zheutlin AB, Kao Y-H, Ayers KL, Gross SJ, Kovatch P, et al. Analysis of hospitalized COVID-19 patients in the Mount Sinai Health System using electronic medical records (EMR) reveals important prognostic factors for improved clinical outcomes. medRxiv. 2020:2020.04.28.20075788.

Not-age adjusted (excluded after data extraction)

4. Patel M, Gangemi A, Marron R, Chowdhury J, Yousef I, Zheng M, et al. Retrospective analysis of high flow nasal therapy in COVID-19-related moderate-to-severe hypoxaemic respiratory failure. BMJ Open Respir Res. 2020;7(1).

Report separate outcomes for men and women

5. Poblador-Plou B, Carmona-Pírez J, Ioakeim-Skoufa I, Poncel-Falcó A, Bliek-Bueno K, Cano-Del Pozo M, et al. Baseline Chronic Comorbidity and Mortality in Laboratory-Confirmed COVID-19 Cases: Results from the PRECOVID Study in Spain. Int J Environ Res Public Health. 2020;17(14):5171.

3. References

1. Aggarwal G, Cheruiyot I, Aggarwal S, et al. Association of Cardiovascular Disease With Coronavirus Disease 2019 (COVID-19) Severity: A Meta-Analysis. *Current problems in cardiology* 2020; **45**(8): 100617.
2. Awortwe C, Cascorbi I. Meta-analysis on outcome-worsening comorbidities of COVID-19 and related potential drug-drug interactions. *Pharmacological Research* 2020: 105250.
3. Bellou V, Tzoulaki I, Evangelou E, Belbasis L. Risk factors for adverse clinical outcomes in patients with COVID-19: A systematic review and meta-analysis. *MedRxiv* 2020.
4. Biswas M, Rahaman S, Biswas TK, Haque Z, Ibrahim B. Association of Sex, Age, and Comorbidities with Mortality in COVID-19 Patients: A Systematic Review and Meta-Analysis. *Intervirology* 2020: 1-12.
5. Biswas M. Effects of Sex, Age and Comorbidities on the Risk of Infection and Death Associated with COVID-19: A Meta-Analysis of 47807 Confirmed Cases. *Lancet* 2020.
6. Chang TH, Chou CC, Chang LY. Effect of obesity and body mass index on coronavirus disease 2019 severity: A systematic review and meta-analysis. *Obesity reviews : an official journal of the International Association for the Study of Obesity* 2020.
7. Chen Y, Gong X, Wang L, Guo J. Effects of hypertension, diabetes and coronary heart disease on COVID-19 diseases severity: a systematic review and meta-analysis. *MedRxiv* 2020.
8. Cheruiyot I, Kipkorir V, Ngure B, Misiani M, Munguti J. Cancer is associated with coronavirus disease (COVID-19) severity and mortality: A pooled analysis. *The American Journal of Emergency Medicine* 2020.
9. Chidambaram V, Tun NL, Haque W, et al. Factors Associated with Disease Severity and Mortality among Patients with Coronavirus Disease 2019: A Systematic Review and Meta-Analysis. medRxiv; 2020.
10. Chu Y, Yang J, Shi J, Zhang P, Wang X. Obesity is associated with increased severity of disease in COVID-19 pneumonia: a systematic review and meta-analysis. *European journal of medical research* 2020; **25**(1): 64.
11. Das P, Samad N, Seidu A-A, Aboagye RG, Tetteh JK, Ahinkorah BO. Obesity as a Predictor for Adverse Outcomes Among COVID-19 Patients: A Meta-Analysis. ResearchSquare; 2020.
12. de Almeida-Pititto B, Dualib PM, Zajdenverg L, et al. Severity and mortality of COVID 19 in patients with diabetes, hypertension and cardiovascular disease: a meta-analysis. *Diabetology & metabolic syndrome* 2020; **12**: 75.
13. Degarege A, Naveed Z, Kabayundo J, Brett-Major D. Risk factors for severe illness and death in COVID-19: a systematic review and meta-analysis. *medRxiv* 2020.
14. Dorjee K, Kim H, Bonomo E, Dolma R. Prevalence and predictors of death and severe disease in patients hospitalized due to COVID-19: A comprehensive systematic review and meta-analysis of 77 studies and 38,000 patients. *PloS one* 2020; **15**(12): e0243191.
15. Du Y, Lv Y, Zha W, Zhou N, Hong X. Association of Body mass index (BMI) with Critical COVID-19 and in-hospital Mortality: a dose-response meta-analysis. *Metabolism* 2020: 154373.
16. Elgohary G. The risk and prognosis of COVID-19 infection in cancer patients: A systematic review and meta-analysis. *Hematology/Oncology and Stem Cell Therapy* 2020.
17. Figliozzi S, Masci PG, Ahmadi N, et al. Predictors of Adverse Prognosis in Covid-19: A Systematic Review and Meta-analysis. *European journal of clinical investigation* 2020: e13362.
18. Florez-Perdomo WA, Serrato-Vargas SA, Bosque-Varela P, et al. Relationship between the history of cerebrovascular disease and mortality in COVID-19 patients: a systematic review and meta-analysis. *Clinical Neurology and Neurosurgery* 2020: 106183.
19. Földi M, Farkas N, Kiss S, et al. Obesity is a risk factor for developing critical condition in COVID-19 patients: A systematic review and meta-analysis. *Obesity reviews : an official journal of the International Association for the Study of Obesity* 2020.
20. Gao Y, Liu M, Shi S, et al. Cancer is associated with the severity and mortality of patients with COVID-19: a systematic review and meta-analysis. *medRxiv* 2020: 2020.05.01.20087031.
21. Giannakoulis VG, Papoutsi E, Siempos, II. Effect of Cancer on Clinical Outcomes of Patients With COVID-19: A Meta-Analysis of Patient Data. *JCO global oncology* 2020; **6**: 799-808.
22. Guo L, Shi Z, Zhang Y, et al. Comorbid diabetes and the risk of disease severity or death among 8807 COVID-19 patients in China: a meta-analysis. *Diabetes Research and Clinical Practice* 2020: 108346.
23. Hariyanto TI, Putri C, Arisa J, Situmeang RFV, Kurniawan A. Dementia and outcomes from coronavirus disease 2019 (COVID-19) pneumonia: A Systematic Review and Meta-Analysis. *Archives of gerontology and geriatrics* 2020: 104299.
24. Hessami A, Shamshirian A, Heydari K, Alizadeh-Navaei R, Moosazadeh M, Abrotan S. Cardiovascular Diseases and COVID-19 Mortality and Intensive Care Unit Admission: A Systematic Review and Meta-analysis. *MedRxiv* 2020.
25. Huang Y, Lu Y, Huang Y-M, et al. Obesity in patients with COVID-19: a systematic review and meta-analysis. *Metabolism: clinical and experimental* 2020; **113**: 154378-.

26. Hussain S, Baxi H, Chand Jamali M, Nisar N, Hussain MS. Burden of diabetes mellitus and its impact on COVID-19 patients: A meta-analysis of real-world evidence. *Diabetes & Metabolic Syndrome: Clinical Research & Reviews* 2020.
27. Islam MS, Barek MA, Aziz MA, Aka TD, Jakaria M. Association of age, sex, comorbidities, and clinical symptoms with the severity and mortality of COVID-19 cases: a meta-analysis with 85 studies and 67299

cases. *MedRxiv* 2020.
28. Izcovich A, Ragusa M. Prognostic Factors for Severity and Mortality in Patients Infected with COVID-19: A Systematic Review. *SSRN* 2020.
29. Kahathuduwa C, Dhanasekara C, Chin S-H. Severity and case fatality rates of COVID-19: a systematic review, meta-analysis and an exploratory meta-regression of risk factors. *Lancet* 2020.
30. Khan M, Khan MN, Mustagir MG, Rana J, Islam MS, Kabir MI. Effects of underlying morbidities on the occurrence of deaths in COVID-19 patients: A systematic review and meta-analysis. *medRxiv* 2020.
31. Khunti K, Singh AK, Gillies CL, et al. The prevalence of comorbidities and their association with mortality in patients with COVID-19: A Systematic Review and Meta-analysis. *Lancet Diabetes Endocrinol* 2020.
32. Kovalic AJ, Satapathy SK, Thuluvath PJ. Prevalence of chronic liver disease in patients with COVID-19 and their clinical outcomes: a systematic review and meta-analysis. *Hepatology international* 2020: 1-9.
33. Kumar A, Arora A, Sharma P, et al. Clinical Features of COVID-19 and Factors Associated with Severe Clinical Course: A Systematic Review and Meta-Analysis. *Ssrn* 2020a: 3566166.
34. Kumar A, Arora A, Sharma P, et al. Is diabetes mellitus associated with mortality and severity of COVID-19? A meta-analysis. *Diabetes and Metabolic Syndrome: Clinical Research and Reviews* 2020b; **14**(4): 535-45.
35. Li B, Yang J, Zhao F, et al. Prevalence and impact of cardiovascular metabolic diseases on COVID-19 in China. *Clinical Research in Cardiology* 2020; **109**(5): 531-8.
36. Li J, Huang DQ, Zou B, et al. Epidemiology of COVID-19: A Systematic Review and Meta-analysis of Clinical Characteristics, Risk factors and Outcomes. *Journal of medical virology* 2020.
37. Li X, Guan B, Su T, et al. Impact of cardiovascular disease and cardiac injury on in-hospital mortality in patients with COVID-19: a systematic review and meta-analysis. *Heart (British Cardiac Society)* 2020.
38. Lippi G, Wong J, Henry BM. Hypertension in patients with coronavirus disease 2019 (COVID-19): A pooled analysis. *Polish Archives of Internal Medicine* 2020; **130**(4): 304-9.
39. Liu H, Chen S, Liu M, Nie H, Lu H. Comorbid Chronic Diseases are Strongly Correlated with Disease Severity among COVID-19 Patients: A Systematic Review and Meta-Analysis. *Aging and disease* 2020; **11**(3): 668-78.
40. Liu N, Sun J, Wang X, Zhao M, Huang Q, Li H. The Impact of Dementia on the Clinical Outcome of COVID-19: A Systematic Review and Meta-Analysis. *Journal of Alzheimer's disease : JAD* 2020.
41. Liu Y, Lu H, Wang W, Liu Q, Zhu C. Clinical risk factors for mortality in patients with cancer and COVID-19: a systematic review and meta-analysis of recent observational studies. *Expert review of anticancer therapy* 2020.
42. Liu Y-F, Zhang Z, Pan X-L, et al. The Chronic Kidney Disease and Acute Kidney Injury Involvement in COVID-19 Pandemic: A Systematic Review and Meta-analysis. *MedRxiv* 2020.
43. Luo L, Fu M, Li Y, et al. The potential association between common comorbidities and severity and mortality of coronavirus disease 2019: A pooled analysis. *Clinical Cardiology* 2020; **n/a**(n/a).
44. Malik P, Patel U, Patel K, et al. Obesity a predictor of outcomes of COVID-19 hospitalized patients- A systematic Review and Meta-Analysis. *Journal of medical virology* 2020.
45. Mantovani A, Byrne CD, Zheng M-H, Targher G. Diabetes as a risk factor for greater COVID-19 severity and in-hospital death: a meta-analysis of observational studies. *Nutrition, Metabolism and Cardiovascular Diseases* 2020.
46. Matsushita K, Ding N, Kou M, et al. The relationship of COVID-19 severity with cardiovascular disease and its traditional risk factors: A systematic review and meta-analysis. *MedRxiv* 2020.
47. Mehraeen E, Karimi A, Barzegary A, et al. Predictors of mortality in patients with COVID-19 – a systematic review. *European Journal of Integrative Medicine* 2020: 101226.
48. Mellor M, Bast A, Jones N, et al. Risk of adverse COVID-19 outcomes for people living with HIV: a rapid review and meta-analysis. *medRxiv* 2020.
49. Meng M, Zhao Q, Kumar R, Bai C, Deng Y, Wan B. Impact of cardiovascular and metabolic diseases on the severity of COVID-19: a systematic review and meta-analysis. *Aging* 2020; **12**.
50. Mesas AE, Cavero-Redondo I, Álvarez-Bueno C, et al. Predictors of in-hospital COVID-19 mortality: A comprehensive systematic review and meta-analysis exploring differences by age, sex and health conditions. *PloS one* 2020; **15**(11): e0241742.

51. Momtazmanesh S, Shobeiri P, Hanaei S, Mahmoud-Elsayed H, Dalvi B, Malakan Rad E. Cardiovascular disease in COVID-19: a systematic review and meta-analysis of 10,898 patients and proposal of a triage risk stratification tool. *Egypt Heart J* 2020; **72**(1): 41-.
52. Moula AI, Micali LR, Matteucci F, et al. Quantification of Death Risk in Relation to Sex, Pre-Existing Cardiovascular Diseases and Risk Factors in COVID-19 Patients: Let's Take Stock and See Where We Are. *Journal of clinical medicine* 2020; **9**(9).
53. Nandy K, Salunke A, Pathak SK, et al. Coronavirus disease (COVID-19): A systematic review and meta-analysis to evaluate the impact of various comorbidities on serious events. *Diabetes & Metabolic Syndrome: Clinical Research & Reviews* 2020.
54. Noor FM, Islam MM. Prevalence and Associated Risk Factors of Mortality Among COVID-19 Patients: A Meta-Analysis. *Journal of community health* 2020.
55. Ofori-Asenso R, Ogundipe O, Agyeman AA, et al. Cancer is associated with severe disease in COVID-19 patients: a systematic review and meta-analysis. *Ecancermedicalscience* 2020; **14**: 1047.
56. Palaiodimos L, Chamorro-Pareja N, Karamanis D, et al. Diabetes is associated with increased risk for in-hospital mortality in patients with COVID-19: a systematic review and meta-analysis comprising 18,506 patients. *MedRxiv* 2020.
57. Pan L, Huang P, Xie X, Xu J, Guo D, Jiang Y. Metabolic associated fatty liver disease increases the severity of COVID-19: A meta-analysis. *Digestive and Liver Disease* 2020.
58. Park R, Chidharla A, Mehta K, Sun W, Wulff-Burchfield E, Kasi A. Sex-bias in COVID-19-associated illness severity and mortality in cancer patients: A systematic review and meta-analysis. *EClinicalMedicine* 2020: 100519.
59. Park R, Lee SA, Kim SY, de Melo AC, Kasi A. Association of active oncologic treatment and risk of death in cancer patients with COVID-19: a systematic review and meta-analysis of patient data. *Acta oncologica (Stockholm, Sweden)* 2020: 1-7.
60. Parohan M, Yaghoubi S, Seraji A, Javanbakht MH, Sarraf P, Djalali M. Risk factors for mortality in patients with Coronavirus disease 2019 (COVID-19) infection: a systematic review and meta-analysis of observational studies. *The aging male : the official journal of the International Society for the Study of the Aging Male* 2020: 1-9.
61. Parveen R, Sehar N, Bajpai R, Bharal Agarwal N. Association of diabetes and hypertension with disease severity in covid-19 patients: a systematic literature review and exploratory meta-analysis. *Diabetes research and clinical practice* 2020: 108295.
62. Patel U, Malik P, Shah D, Patel A, Dhamoon M, Jani V. Pre-existing cerebrovascular disease and poor outcomes of COVID-19 hospitalized patients: a meta-analysis. *Journal of neurology* 2020a: 1-8.
63. Patel U, Malik P, Usman MS, et al. Age-Adjusted Risk Factors Associated with Mortality and Mechanical Ventilation Utilization Amongst COVID-19 Hospitalizations—a Systematic Review and Meta-Analysis. *SN Comprehensive Clinical Medicine* 2020b.
64. Popkin BM, Du S, Green WD, et al. Individuals with obesity and COVID-19: A global perspective on the epidemiology and biological relationships. *Obesity reviews : an official journal of the International Association for the Study of Obesity* 2020.
65. Pranata R, Supriyadi R, Huang I, et al. The Association Between Chronic Kidney Disease and New Onset Renal Replacement Therapy on the Outcome of COVID-19 Patients: A Meta-analysis. *Clinical Medicine Insights: Circulatory, Respiratory and Pulmonary Medicine* 2020; **14**.
66. Pranata R, Soeroto AY, Huang I, et al. Effect of chronic obstructive pulmonary disease and smoking on the outcome of COVID-19. *The international journal of tuberculosis and lung disease : the official journal of the International Union against Tuberculosis and Lung Disease* 2020; **24**(8): 838-43.
67. Pranata R, Huang I, Raharjo SB. Incidence and impact of cardiac arrhythmias in coronavirus disease 2019 (COVID-19): A systematic review and meta-analysis. *Indian Pacing and Electrophysiology Journal* 2020.
68. Pranata R, Lim MA, Huang I, Raharjo SB, Lukito AA. Hypertension is associated with increased mortality and severity of disease in COVID-19 pneumonia: A systematic review, meta-analysis and meta-regression. *Journal of the renin-angiotensin-aldosterone system : JRAAS* 2020a; **21**(2): 1470320320926899.
69. Pranata R, Lim MA, Yonas E, et al. Body Mass Index and Outcome in Patients with COVID-19: A Dose-Response Meta-Analysis. *Diabetes & Metabolism* 2020b.
70. Rahman A, Sathi NJ. Risk Factors of the Severity of COVID-19: A Meta-Analysis. *MedRxiv* 2020.
71. Roncon L, Zuin M, Rigatelli G, Zuliani G. Diabetic patients with COVID-19 infection are at higher risk of ICU admission and poor short-term outcome. *Journal of clinical virology : the official publication of the Pan American Society for Clinical Virology* 2020; **127**: 104354.
72. Sabatino J, De Rosa S, Di Salvo G, Indolfi C. Impact of cardiovascular risk profile on COVID-19 outcome. A meta-analysis. *PloS one* 2020; **15**(8): e0237131.
73. Salunke AA, Nandy K, Pathak SK, et al. Impact of COVID -19 in cancer patients on severity of disease and fatal outcomes: A systematic review and meta-analysis. *Diabetes & Metabolic Syndrome: Clinical Research & Reviews* 2020.

74. Sanchez-Ramirez DC, Mackey D. Underlying respiratory diseases, specifically COPD, and smoking are associated with severe COVID-19 outcomes: A systematic review and meta-analysis. *Respiratory Medicine* 2020: 106096.
75. Sepandi M, Taghdir M, Alimohamadi Y, Afrashteh S, Hosamirudsari H. Factors Associated with Mortality in COVID-19 Patients: A Systematic Review and Meta-Analysis. *Iranian journal of public health* 2020; **49**(7): 1211-21.
76. Shang L, Shao M, Guo Q, et al. Diabetes Mellitus is Associated with Severe Infection and Mortality in Patients with COVID-19: A Systematic Review and Meta-analysis. *Archives of Medical Research* 2020.
77. Shi C, Wang L, Ye J, et al. Predictors of mortality in patients with coronavirus disease 2019: a systematic review and meta-analysis. ResearchSquare; 2020.
78. Singh AK, Jena A, Kumar MP, Sharma V, Sebastian S. Risk and outcomes of coronavirus disease (COVID-19) in patients with inflammatory bowel disease: a systematic review and meta-analysis. *United European gastroenterology journal* 2020: 2050640620972602.
79. Soeroto AY, Soetedjo NN, Purwiga A, et al. Effect of increased BMI and obesity on the outcome of COVID-19 adult patients: A systematic review and meta-analysis. *Diabetes & Metabolic Syndrome: Clinical Research & Reviews* 2020; **14**(6): 1897-904.
80. Sreenivasan J, Khan MS, Anker SD, et al. Cardiovascular Risk Factors and Complications in Patients Infected with COVID-19: A Systematic Review. *Lancet*.
81. Ssentongo P, Ssentongo AE, Heilbrunn ES, Ba DM, Chinchilli VM. Association of cardiovascular disease and 10 other pre-existing comorbidities with COVID-19 mortality: A systematic review and meta-analysis. *PloS one* 2020; **15**(8): e0238215.
82. Su Q, Hu J-x, Lin H-s, et al. Prevalence and risks of severe events for cancer patients with COVID-19 infection: a systematic review and meta-analysis. *medrxiv* 2020.
83. Sze S, Pan D, Nevill CR, et al. Ethnicity and clinical outcomes in COVID-19: A systematic review and meta-analysis. *EClinicalMedicine* 2020: 100630.
84. Tabrizi R, Lankarani KB, Nowrouzi-sohrabi P, et al. The role of comorbidities and clinical predictors of severe disease in COVID-19: a systematic review and meta-analysis. *MedRxiv* 2020.
85. Tamara A, Tahapary DL. Obesity as a predictor for a poor prognosis of COVID-19: A systematic review. *Diabetes & Metabolic Syndrome: Clinical Research & Reviews* 2020.
86. Tamuzi JL, Ayele BT, Shumba CS, et al. Implications of COVID-19 in high burden countries for HIV/TB: A systematic review of evidence. *BMC infectious diseases* 2020; **20**(1): 744.
87. Tavan H, Shams M, Karimian M, Kalvandi G, Borji M. An evaluation on the frequencies of underlying diseases and symptoms along with the mortality rate of COVID-19, a systematic review and meta-analysis. *Lancet* 2020.
88. Tian W, Jiang W, Yao J, et al. Predictors of mortality in hospitalized COVID-19 patients: A systematic review and meta-analysis. *Journal of medical virology* 2020; **92**(10): 1875-83.
89. Tian Y, Qiu X, Wang C, et al. Cancer associates with risk and severe events of COVID-19: A systematic review and meta-analysis. *International journal of cancer* 2020.
90. Tian Y, Wu Q, Li, et al. Distinct symptoms and underlying comorbidities with latitude and longitude in COVID-19: A systematic review and meta-analysis. ResearchSquare; 2020.
91. Toraih EA, Elshazli RM, Hussein MH, et al. Association of cardiac biomarkers and comorbidities with increased mortality, severity, and cardiac injury in COVID-19 patients: A meta-regression and Decision tree analysis. *Journal of medical virology* 2020.
92. Varikasuvu SR, Dutt N, Thangappazham B, Varshney S. Diabetes and COVID-19: A pooled analysis related to disease severity and mortality. *Primary Care Diabetes* 2020.
93. Venkatesulu BP, Chandrasekar VT, Girdhar P, et al. A systematic review and meta-analysis of cancer patients affected by a novel coronavirus. *medRxiv* 2020.
94. Vijenthira A, Gong IY, Fox TA, et al. Outcomes of patients with hematologic malignancies and COVID-19: A systematic review and meta-analysis of 3377 patients. *Blood* 2020.
95. Fernandez Villalobos NV, Ott JJ, Klett-Tammen CJ, et al. Quantification of the association between predisposing health conditions, demographic, and behavioural factors with hospitalisation, intensive care unit admission, and death from COVID-19: a systematic review and meta-analysis. medRxiv; 2020.
96. Wang B, Li R, Lu Z, Huang Y. Does comorbidity increase the risk of patients with COVID-19: evidence from meta-analysis. *Aging* 2020; **12**.
97. Wang X, Fang X, Cai Z, et al. Comorbid Chronic Diseases and Acute Organ Injuries Are Strongly Correlated with Disease Severity and Mortality among COVID-19 Patients: A Systemic Review and Meta-Analysis. *Research (Washington, DC)* 2020; **2020**: 2402961.
98. Wang Y, Ao G, Qi X, Xie B. The association between COVID-19 and asthma: a systematic review and meta-analysis. *Clinical and experimental allergy : journal of the British Society for Allergy and Clinical Immunology* 2020.

99. Wang Y, Chen J, Chen W, et al. Does Asthma Increase the Mortality of Patients with COVID-19?: A Systematic Review and Meta-Analysis. *International archives of allergy and immunology* 2020: 1-7.
100. Wang Z, Deng H, Ou C, et al. Clinical symptoms, comorbidities and complications in severe and non-severe patients with COVID-19: A systematic review and meta-analysis without cases duplication. *Medicine* 2020; **99**(48): e23327.
101. Wingert A, Pillay J, Gates M, et al. Risk factors for severe outcomes of COVID-19: a rapid review. *medRxiv* 2020: 2020.08.27.20183434.
102. Wu J, Zhang J, Sun X, et al. Influence of diabetes mellitus on the severity and fatality of SARS-CoV-2 infection. *Diabetes, obesity & metabolism* 2020.
103. Wu ZH, Tang Y, Cheng Q. Diabetes increases the mortality of patients with COVID-19: a meta-analysis. *Acta diabetologica* 2020.
104. Xu J, Xiao W, Liang X, et al. The Association of Cerebrovascular Disease with Adverse Outcomes in COVID-19 Patients: A Meta-Analysis Based on Adjusted Effect Estimates. *Journal of Stroke and Cerebrovascular Diseases* 2020; **29**(11): 105283.
105. Xu L, Mao Y, Chen G. Risk factors for 2019 novel coronavirus disease (COVID-19) patients progressing to critical illness: a systematic review and meta-analysis. *Aging* 2020; **12**.
106. Yang J, Hu J, Zhu C. Obesity aggravates COVID-19: a systematic review and meta-analysis. *Journal of medical virology* 2020.
107. Yang J, Tian C, Chen Y, Zhu C, Chi H, Li J. Obesity aggravates COVID-19: an updated systematic review and meta-analysis. *Journal of medical virology* 2020.
108. Yang J, Zheng Y, Gou X, et al. Prevalence of comorbidities in the novel Wuhan coronavirus (COVID-19) infection: a systematic review and meta-analysis. *International Journal of Infectious Diseases* 2020.
109. Yang S, Chen C, Yan D, et al. Risk factors associated with fatal outcomes of novel coronavirus infection pneumonia (COVID-19): A systematic review and meta-analysis. *ResearchSquare* 2020.
110. Yekedüz E, Utkan G, Ürün Y. A Systematic Review and Meta-Analysis: The Effect of Active Cancer Treatment on Severity of COVID-19. *European Journal of Cancer* 2020.
111. Yonas E, Alwi I, Pranata R, et al. Effect of heart failure on the outcome of COVID-19 — A meta analysis and systematic review. *The American Journal of Emergency Medicine* 2020.
112. Youssef M, Hussein M, Attia AS, et al. COVID-19 and Liver Dysfunction: a systematic review and meta-analysis of retrospective studies. *Journal of medical virology* 2020.
113. Yu JN, Wu BB, Yang J, Lei XL, Shen WQ. Cardio-Cerebrovascular Disease is Associated With Severity and Mortality of COVID-19: A Systematic Review and Meta-Analysis. *Biological research for nursing* 2020: 1099800420951984.
114. Zaki N, Mohamed EA, Ibrahim S, Khan G. The influence of comorbidity on the severity of COVID-19 disease: A systematic review and analysis. *ResearchSquare* 2020.
115. Zhang H, Han H, He T, et al. Clinical Characteristics and Outcomes of COVID-19-Infected Cancer Patients: A Systematic Review and Meta-Analysis. *Journal of the National Cancer Institute* 2020.
116. Zhang J, Wu J, Sun X, et al. Associations of hypertension with the severity and fatality of SARS-CoV-2 infection: A meta-analysis. *Epidemiology and infection* 2020: 1-19.
117. Zhao Q, Meng M, Kumar R, et al. The impact of COPD and smoking history on the severity of Covid-19: A systemic review and meta-analysis. *Journal of medical virology* 2020.
118. Zheng Z, Peng F, Xu B, et al. Risk factors of critical & mortal COVID-19 cases: A systematic literature review and meta-analysis. *Journal of Infection* 2020.
119. Zhou Y, Yang Q, Chi J, et al. Comorbidities and the risk of severe or fatal outcomes associated with coronavirus disease 2019: A systematic review and meta-analysis. *International Journal of Infectious Diseases* 2020.
120. Zuin M, Guasti P, Roncon L, Cervellati C, Zuliani G. Dementia and the risk of death in elderly patients with COVID-19 infection: Systematic review and meta-analysis. *International Journal of Geriatric Psychiatry* 2020.
121. Boulle A, Davies M-A, Hussey H, et al. Risk factors for COVID-19 death in a population cohort study from the Western Cape Province, South Africa. *Clinical infectious diseases : an official publication of the Infectious Diseases Society of America* 2020: ciaa1198.
122. Al-Sabah S, Al-Haddad M, Al-Youha S, Jamal M, Almazeedi S. COVID-19: Impact of obesity and diabetes on disease severity. *Clinical Obesity* 2020; **10**(6): e12414.
123. Rastad H, Karim H, Ejtahed H-S, et al. Risk and predictors of in-hospital mortality from COVID-19 in patients with diabetes and cardiovascular disease. *Diabetology & metabolic syndrome* 2020; **12**(1): 57.
124. Al-Salameh A, Lanoix JP, Bennis Y, et al. Characteristics and outcomes of COVID-19 in hospitalized patients with and without diabetes. *Diabetes Metab Res Rev* 2020: e3388.
125. Amit M, Sorkin A, Chen J, et al. Clinical Course and Outcomes of Severe Covid-19: A National Scale Study. *Journal of clinical medicine* 2020; **9**(7).

126. Bellan M, Patti G, Hayden E, et al. Fatality rate and predictors of mortality in an Italian cohort of hospitalized COVID-19 patients. *Scientific Reports* 2020; **10**(1): 20731.
127. Berenguer J, Ryan P, Rodríguez-Baño J, et al. Characteristics and predictors of death among 4035 consecutively hospitalized patients with COVID-19 in Spain. *Clinical microbiology and infection : the official publication of the European Society of Clinical Microbiology and Infectious Diseases* 2020; **26**(11): 1525-36.
128. Bezzio C, Saibeni S, Variola A, et al. Outcomes of COVID-19 in 79 patients with IBD in Italy: an IG-IBD study. *Gut* 2020; **69**(7): 1213-7.
129. Bianchetti A, Rozzini R, Guerini F, et al. Clinical Presentation of COVID19 in Dementia Patients. *J Nutr Health Aging* 2020; **24**(6): 560-2.
130. Borghesi A, Zigliani A, Golemi S, et al. Chest X-ray severity index as a predictor of in-hospital mortality in coronavirus disease 2019: A study of 302 patients from Italy. *International Journal of Infectious Diseases* 2020; **96**: 291-3.
131. Burn E, Tebe C, Fernandez-Bertolin S, et al. The natural history of symptomatic COVID-19 in Catalonia, Spain: a multi-state model including 109,367 outpatient diagnoses, 18,019 hospitalisations, and 5,585 COVID-19 deaths among 5,627,520 people. *medRxiv* 2020: 2020.07.13.20152454.
132. Busetto L, Bettini S, Fabris R, et al. Obesity and COVID-19: an Italian snapshot. *Obesity (Silver Spring)* 2020.
133. Cariou B, Hadjadj S, Wargny M, et al. Phenotypic characteristics and prognosis of inpatients with COVID-19 and diabetes: the CORONADO study. *Diabetologia* 2020; **63**(8): 1500-15.
134. Carter B, Collins JT, Barlow-Pay F, et al. Nosocomial COVID-19 infection: examining the risk of mortality. The COPE-Nosocomial Study (COVID in Older PEople). *J Hosp Infect* 2020; **106**(2): 376-84.
135. Caussy C, Pattou F, Wallet F, et al. Prevalence of obesity among adult inpatients with COVID-19 in France. *Lancet Diabetes Endocrinol* 2020.
136. Ciardullo S, Zerbini F, Perra S, et al. Impact of diabetes on COVID-19-related in-hospital mortality: a retrospective study from Northern Italy. *J Endocrinol Invest* 2020.
137. Ciceri F, Castagna A, Rovere-Querini P, et al. Early predictors of clinical outcomes of COVID-19 outbreak in Milan, Italy. *Clin Immunol* 2020; **217**: 108509.
138. Conversano A, Melillo F, Napolano A, et al. Renin-Angiotensin-Aldosterone System Inhibitors and Outcome in Patients With SARS-CoV-2 Pneumonia: A Case Series Study. *Hypertension* 2020; **76**(2): e10-e2.
139. Di Castelnuovo A, Bonaccio M, Costanzo S, et al. Common cardiovascular risk factors and in-hospital mortality in 3,894 patients with COVID-19: survival analysis and machine learning-based findings from the multicentre Italian CORIST Study. *Nutr Metab Cardiovasc Dis* 2020; **30**(11): 1899-913.
140. Docherty AB, Harrison EM, Green CA, et al. Features of 20 133 UK patients in hospital with covid-19 using the ISARIC WHO Clinical Characterisation Protocol: prospective observational cohort study. *Bmj* 2020; **369**: m1985.
141. Galloway JB, Norton S, Barker RD, et al. A clinical risk score to identify patients with COVID-19 at high risk of critical care admission or death: An observational cohort study. *Journal of Infection* 2020; **81**(2): 282-8.
142. Geretti AM, Stockdale AJ, Kelly SH, et al. Outcomes of COVID-19 related hospitalization among people with HIV in the ISARIC WHO Clinical Characterization Protocol (UK): a prospective observational study. *Clinical infectious diseases : an official publication of the Infectious Diseases Society of America* 2020.
143. Giacomelli A, Ridolfo AL, Milazzo L, et al. 30-day mortality in patients hospitalized with COVID-19 during the first wave of the Italian epidemic: a prospective cohort study. *medRxiv* 2020: 2020.05.02.20088336.
144. Goicoechea M, Sánchez Cámara LA, Macías N, et al. COVID-19: clinical course and outcomes of 36 hemodialysis patients in Spain. *Kidney international* 2020; **98**(1): 27-34.
145. Grasselli G, Greco M, Zanella A, et al. Risk Factors Associated With Mortality Among Patients With COVID-19 in Intensive Care Units in Lombardy, Italy. *JAMA internal medicine* 2020; **180**(10): 1345-55.
146. Halasz G, Leoni ML, Villani GQ, Nolli M, Villani M. Obesity, overweight and survival in critically ill patients with SARS-CoV-2 pneumonia: is there an obesity paradox? Preliminary results from Italy. *Eur J Prev Cardiol* 2020: 2047487320939675.
147. Hewitt J, Carter B, Vilches-Moraga A, et al. The effect of frailty on survival in patients with COVID-19 (COPE): a multicentre, European, observational cohort study. *The Lancet Public Health* 2020; **5**(8): e444-e51.
148. Khalil K, Agbontaen K, McNally D, et al. Clinical characteristics and 28-day mortality of medical patients admitted with COVID-19 to a central London teaching hospital. *The Journal of infection* 2020; **81**(3): e85-e9.
149. Lee LYW, Cazier J-B, Angelis V, et al. COVID-19 mortality in patients with cancer on chemotherapy or other anticancer treatments: a prospective cohort study. *The Lancet* 2020; **395**(10241): 1919-26.
150. Lee LYW, Cazier J-B, Starkey T, et al. COVID-19 prevalence and mortality in patients with cancer and the effect of primary tumour subtype and patient demographics: a prospective cohort study. *The Lancet Oncology* 2020; **21**(10): 1309-16.

151. Merzon E, Tworowski D, Gorohovski A, et al. Low plasma 25(OH) vitamin D level is associated with increased risk of COVID-19 infection: an Israeli population-based study. *FEBS J* 2020: 10.1111/febs.15495.
152. Passamonti F, Cattaneo C, Arcaini L, et al. Clinical characteristics and risk factors associated with COVID-19 severity in patients with haematological malignancies in Italy: a retrospective, multicentre, cohort study. *The Lancet Haematology* 2020; **7**.
153. Perez-Guzman PN, Daunt A, Mukherjee S, et al. Clinical characteristics and predictors of outcomes of hospitalized patients with COVID-19 in a multi-ethnic London NHS Trust: a retrospective cohort study. *Clinical infectious diseases : an official publication of the Infectious Diseases Society of America* 2020.
154. Pinato DJ, Zambelli A, Aguilar-Company J, et al. Clinical portrait of the SARS-CoV-2 epidemic in European cancer patients. *Cancer Discov* 2020; **10**(10): 1465-74.
155. Regina J, Papadimitriou-Olivgeris M, Burger R, et al. Epidemiology, risk factors and clinical course of SARS-CoV-2 infected patients in a Swiss university hospital: An observational retrospective study. *PloS one* 2020; **15**(11): e0240781.
156. Reilev M, Kristensen KB, Pottegård A, et al. Characteristics and predictors of hospitalization and death in the first 11 122 cases with a positive RT-PCR test for SARS-CoV-2 in Denmark: a nationwide cohort. *Int J Epidemiol* 2020: dyaa140.
157. Rivera-Izquierdo M, Del Carmen Valero-Ubierna M, R-delAmo JL, et al. Sociodemographic, clinical and laboratory factors on admission associated with COVID-19 mortality in hospitalized patients: A retrospective observational study. *PloS one* 2020; **15**(6): e0235107-e.
158. Rossi A, Gottin L, Donadello K. Obesity as a risk factor for unfavourable outcomes in critically ill patients affected by Covid-19 related respiratory failure. *Research Square* 2020.
159. Rossi PG, Marino M, Formisano D, Venturelli F, Vicentini M, Grilli R. Characteristics and outcomes of a cohort of SARS-CoV-2 patients in the Province of Reggio Emilia, Italy. *PloS one* 2020; **15(8)**: e0238281.
160. Rottoli M, Bernante P, Belvedere A, et al. How important is obesity as a risk factor for respiratory failure, intensive care admission and death in hospitalised COVID-19 patients? Results from a single Italian centre. *European journal of endocrinology* 2020.
161. Russell B, Moss C, Papa S, et al. Factors Affecting COVID-19 Outcomes in Cancer Patients: A First Report From Guy's Cancer Center in London. *Frontiers in Oncology* 2020; **10**(1279).
162. Sanchez-Pina JM, Rodríguez Rodriguez M, Castro Quismondo N, et al. Clinical course and risk factors for mortality from COVID-19 in patients with haematological malignancies. *European Journal of Haematology* 2020; **105**(5): 597-607.
163. Sapey E, Gallier S, Mainey C, et al. Ethnicity and risk of death in patients hospitalised for COVID-19 infection in the UK: an observational cohort study in an urban catchment area. *BMJ Open Respiratory Research* 2020; **7**(1): e000644.
164. Shah V, Ko Ko T, Zuckerman M, et al. Poor outcome and prolonged persistence of SARS-CoV-2 RNA in COVID-19 patients with haematological malignancies; King's College Hospital experience. *Br J Haematol* 2020; **190**(5): e279-e82.
165. Simonnet A, Chetboun M, Poissy J, et al. High Prevalence of Obesity in Severe Acute Respiratory Syndrome Coronavirus-2 (SARS-CoV-2) Requiring Invasive Mechanical Ventilation. *Obesity (Silver Spring)* 2020; **28**(7): 1195-9.
166. Sisó-Almirall A, Kostov B, Mas-Heredia M, et al. Prognostic factors in Spanish COVID-19 patients: A case series from Barcelona. *PloS one* 2020; **15**(8): e0237960-e.
167. Wendel Garcia P, Fumeaux T, Guerci P, et al. Prognostic factors associated with mortality risk and disease progression in 639 critically ill patients with COVID-19 in Europe: Initial report of the international RISC-19-ICU prospective observational cohort. *EClinicalMedicine* 2020; **25**: 100449.
168. Yarza R, Bover M, Paredes D, et al. SARS-CoV-2 infection in cancer patients undergoing active treatment: analysis of clinical features and predictive factors for severe respiratory failure and death. *Eur J Cancer* 2020; **135**: 242-50.
169. Antwi-Amoabeng D, Beutler B, Awad M, et al. Sociodemographic predictors of outcomes in COVID-19: examining the impact of ethnic disparities in Northern Nevada. *Cureus* 2020; **13(2)**: e13128.
170. Argenziano M, Bruce S, Slater C. Characterization and Clinical Course of 1000 Patients with COVID-19 in New York: retrospective case series. *medrxiv* 2020: 10.1101.
171. Azar KMJ, Shen Z, Romanelli RJ, et al. Disparities In Outcomes Among COVID-19 Patients In A Large Health Care System In California. *Health affairs (Project Hope)* 2020; **39**(7): 1253-62.
172. Chhiba KD, Patel GB, Vu THT, et al. Prevalence and characterization of asthma in hospitalized and nonhospitalized patients with COVID-19. *J Allergy Clin Immunol* 2020; **146**(2): 307-14.e4.
173. Chilimuri S, Sun H, Alemam A, et al. Predictors of Mortality in Adults Admitted with COVID-19: Retrospective Cohort Study from New York City. *West J Emerg Med* 2020; **21**(4): 779-84.
174. Costa Monteiro AC, Suri R, Emeruwa IO, et al. Obesity and Smoking as Risk Factors for Invasive Mechanical Ventilation in COVID-19: a Retrospective, Observational Cohort Study. *PLOS ONE* 2020; **15(12)**: e0238552.

175. Crouse AB, Grimes T, Li P, Might M, Ovalle F, Shalev A. Metformin Use Is Associated With Reduced Mortality in a Diverse Population With COVID-19 and Diabetes. *Frontiers in Endocrinology* 2021; **11**(1081).
176. Cummings MJ, Baldwin MR, Abrams D, et al. Epidemiology, clinical course, and outcomes of critically ill adults with COVID-19 in New York City: a prospective cohort study. *Lancet* 2020.
177. D'Silva KM, Serling-Boyd N, Wallwork R, et al. Clinical characteristics and outcomes of patients with coronavirus disease 2019 (COVID-19) and rheumatic disease: a comparative cohort study from a US 'hot spot'. *Annals of the Rheumatic Diseases* 2020; **79**(9): 1156-62.
178. Ebinger JE, Achamallah N, Ji H, et al. Pre-existing traits associated with Covid-19 illness severity. *PloS one* 2020; **15**(7): e0236240-e.
179. Gayam V, Chobufo MD, Merghani MA, Lamichhane S, Garlapati PR, Adler MK. Clinical characteristics and predictors of mortality in African-Americans with COVID-19 from an inner-city community teaching hospital in New York. *Journal of medical virology* 2020; **n/a**(n/a).
180. Goyal P, Ringel JB, Rajan M, et al. Obesity and COVID-19 in New York City: A Retrospective Cohort Study. *Annals of internal medicine* 2020; **173**(10): 855-8.
181. Gu T, Mack JA, Salvatore M, et al. COVID-19 outcomes, risk factors and associations by race: a comprehensive analysis using electronic health records data in Michigan Medicine. *medRxiv : the preprint server for health sciences* 2020: 2020.06.16.20133140.
182. Gupta S, Hayek SS, Wang W, et al. Factors Associated With Death in Critically Ill Patients With Coronavirus Disease 2019 in the US. *JAMA internal medicine* 2020; **180**(11): 1-12.
183. Harmouch F, Shah K, Hippen JT, Kumar A, Goel H. Is it all in the heart? Myocardial injury as major predictor of mortality among hospitalized COVID-19 patients. *Journal of medical virology* 2020; **n/a**(n/a).
184. Harrison SL, Fazio-Eynullayeva E, Lane DA, Underhill P, Lip GYH. Comorbidities associated with mortality in 31,461 adults with COVID-19 in the United States: A federated electronic medical record analysis. *PLOS Medicine* 2020; **17**(9): e1003321.
185. Hashemi N, Viveiros K, Redd WD, et al. Impact of chronic liver disease on outcomes of hospitalized patients with COVID-19: A multicentre United States experience. *Liver international : official journal of the International Association for the Study of the Liver* 2020; **40**(10): 2515-21.
186. Hur K, Price C, Gray E, et al. Factors Associated With Intubation and Prolonged Intubation in Hospitalized Patients With COVID-19. *Otolaryngology Head and Neck Surgery* 2020; **163**: 019459982092964.
187. Imam Z, Odish F, Gill I, et al. Older age and comorbidity are independent mortality predictors in a large cohort of 1305 COVID-19 patients in Michigan, United States. *Journal of internal medicine* 2020; **288**(4): 469-76.
188. Jun T, Nirenberg S, Kovatch P, Huang K-l. Sex-specificity of mortality risk factors among hospitalized COVID-19 patients in New York City: prospective cohort study. *medRxiv* 2020: 2020.07.29.20164640.
189. Kabarriti R, Brodin NP, Maron MI, et al. Association of Race and Ethnicity With Comorbidities and Survival Among Patients With COVID-19 at an Urban Medical Center in New York. *JAMA Netw Open* 2020; **3**(9): e2019795-e.
190. Kalligeros M, Shehadeh F, Mylona EK, et al. Association of Obesity with Disease Severity Among Patients with Coronavirus Disease 2019. *Obesity* 2020; **28**(7): 1200-4.
191. Killerby M, Link-Gelles R, Haight S, et al. Characteristics Associated with Hospitalization Among Patients with COVID-19 — Metropolitan Atlanta, Georgia, March–April 2020. *MMWR Morbidity and mortality weekly report* 2020; **69**.
192. Kim L, Garg S, O'Halloran A, et al. Risk Factors for Intensive Care Unit Admission and In-hospital Mortality Among Hospitalized Adults Identified through the US Coronavirus Disease 2019 (COVID-19)-Associated Hospitalization Surveillance Network (COVID-NET). *Clinical Infectious Diseases* 2020.
193. Klang E, Kassim G, Soffer S, Freeman R, Levin MA, Reich DL. Severe Obesity as an Independent Risk Factor for COVID-19 Mortality in Hospitalized Patients Younger than 50. *Obesity (Silver Spring)* 2020.
194. Lieberman-Cribbin W, Rapp J, Alpert N, Tuminello S, Taioli E. The Impact of Asthma on Mortality in Patients With COVID-19. *Chest* 2020.
195. Magleby R, Westblade LF, Trzebucki A, et al. Impact of SARS-CoV-2 Viral Load on Risk of Intubation and Mortality Among Hospitalized Patients with Coronavirus Disease 2019. *Clinical infectious diseases : an official publication of the Infectious Diseases Society of America* 2020: ciaa851.
196. Mahdavinia M, Foster KJ, Jauregui E, et al. Asthma prolongs intubation in COVID-19. *J Allergy Clin Immunol Pract* 2020; **8**(7): 2388-91.
197. McCarty TR, Hathorn KE, Redd WD, et al. How Do Presenting Symptoms and Outcomes Differ by Race/Ethnicity Among Hospitalized Patients with COVID-19 Infection? Experience in Massachusetts. *Clinical infectious diseases : an official publication of the Infectious Diseases Society of America* 2020: ciaa1245.
198. Mehta V, Goel S, Kabarriti R, et al. Case Fatality Rate of Cancer Patients with COVID-19 in a New York Hospital System. *Cancer Discov* 2020.
199. Mendy A, Apewokin S, Wells A, Morrow A. Factors Associated with Hospitalization and Disease Severity in a Racially and Ethnically Diverse Population of COVID-19 Patients. *medrxiv* 2020: 10.1101.

200. Mikami T, Miyashita H, Yamada T, et al. Risk Factors for Mortality in Patients with COVID-19 in New York City. *Journal of general internal medicine* 2020: 1-10.
201. Miyashita H, Kuno T. Prognosis of coronavirus disease 2019 (COVID-19) in patients with HIV infection in New York City. *HIV medicine* 2020.
202. Miyashita H, Mikami T, Chopra N, et al. Do Patients with Cancer Have a Poorer Prognosis of COVID-19? An Experience in New York City. *Annals of Oncology* 2020.
203. Miyashita S, Yamada T, Mikami T, Miyashita H, Chopra N, Rizk D. Impact of dementia on clinical outcomes in elderly patients with coronavirus 2019 (COVID-19): an experience in New York. *Geriatrics & Gerontology International* 2020; **20**(7): 732-4.
204. Nakeshbandi M, Maini R, Daniel P, et al. The impact of obesity on COVID-19 complications: a retrospective cohort study. *International journal of obesity (2005)* 2020; **44**(9): 1832-7.
205. Narain S, Stefanov DG, Chau AS, et al. Comparative Survival Analysis of Immunomodulatory Therapy for Coronavirus Disease 2019 Cytokine Storm. *Chest* 2020: S0012-3692(20)34901-1.
206. Okoh AK, Sossou C, Dangayach NS, et al. Coronavirus disease 19 in minority populations of Newark, New Jersey. *Int J Equity Health* 2020; **19**(1): 93-.
207. Palaiodimos L, Kokkinidis DG, Li W, et al. Severe obesity, increasing age and male sex are independently associated with worse in-hospital outcomes, and higher in-hospital mortality, in a cohort of patients with COVID-19 in the Bronx, New York. *Metabolism* 2020; **108**: 154262.
208. Patel NG, Bhasin A, Feinglass JM, et al. Clinical Outcomes of Hospitalized Patients with COVID-19 on Therapeutic Anticoagulants. *medRxiv* 2020: 2020.08.22.20179911.
209. Petrilli CM, Jones SA, Yang J, et al. Factors associated with hospital admission and critical illness among 5279 people with coronavirus disease 2019 in New York City: prospective cohort study. *Bmj* 2020; **369**: m1966.
210. Pettit NN, MacKenzie EL, Ridgway J, et al. Obesity is Associated with Increased Risk for Mortality Among Hospitalized Patients with COVID-19. *Obesity (Silver Spring)* 2020.
211. Price-Haywood EG, Burton J, Fort D, Seoane L. Hospitalization and Mortality among Black Patients and White Patients with Covid-19. *N Engl J Med* 2020.
212. Rentsch CT, Kidwai-Khan F, Tate JP, et al. Covid-19 Testing, Hospital Admission, and Intensive Care Among 2,026,227 United States Veterans Aged 54-75 Years. *medRxiv : the preprint server for health sciences* 2020: 2020.04.09.20059964.
213. Robilotti EV, Babady NE, Mead PA, et al. Determinants of COVID-19 disease severity in patients with cancer. *Nature Medicine* 2020; **26**(8): 1218-23.
214. Salacup G, Lo KB, Gul F, et al. Characteristics and clinical outcomes of COVID-19 patients in an underserved-inner city population: A single tertiary center cohort. *Journal of medical virology* 2020; **n/a**(n/a).
215. Seiglie J, Platt J, Cromer SJ, et al. Diabetes as a Risk Factor for Poor Early Outcomes in Patients Hospitalized With COVID-19. *Diabetes care* 2020; **43**(12): 2938-44.
216. Shah P, Owens J, Franklin J, et al. Demographics, comorbidities and outcomes in hospitalized Covid-19 patients in rural southwest Georgia. *Annals of medicine* 2020; **52**(7): 354-60.
217. Sigel K, Swartz T, Golden E, et al. Covid-19 and People with HIV Infection: Outcomes for Hospitalized Patients in New York City. *Clinical infectious diseases : an official publication of the Infectious Diseases Society of America* 2020: ciaa880.
218. Singh S, Bilal M, Khan A, et al. Outcomes of COVID-19 in Patients with Obesity in United States: A Large Research Network Study. *SSRN Electronic Journal* 2020.
219. Singh S, Khan A. Clinical Characteristics and Outcomes of Coronavirus Disease 2019 Among Patients With Preexisting Liver Disease in the United States: A Multicenter Research Network Study. *Gastroenterology* 2020; **159**(2): 768-71.e3.
220. Singh S, Khan A, Chowdhry M, Bilal M, Kochhar GS, Clarke K. Risk of Severe Coronavirus Disease 2019 in Patients With Inflammatory Bowel Disease in the United States: A Multicenter Research Network Study. *Gastroenterology* 2020; **159**(4): 1575-8.e4.
221. Smith AA, Fridling J, Ibrahim D, Porter PS, Jr. Identifying Patients at Greatest Risk of Mortality due to COVID-19: A New England Perspective. *West J Emerg Med* 2020; **21**(4): 785-9.
222. Suleyman G, Fadel RA, Malette KM, et al. Clinical Characteristics and Morbidity Associated With Coronavirus Disease 2019 in a Series of Patients in Metropolitan Detroit. *JAMA Netw Open* 2020; **3**(6): e2012270-e.
223. Tartof SY, Qian L, Hong V, et al. Obesity and Mortality Among Patients Diagnosed With COVID-19: Results From an Integrated Health Care Organization. *Annals of internal medicine* 2020; **173**(10): 773-81.
224. van Gerwen M, Alsen M, Little C, et al. Risk factors and outcomes of COVID-19 in New York City; a retrospective cohort study. *Journal of medical virology* 2020.
225. Wang A-L, Zhong X, Hurd Y. Comorbidity and Sociodemographic determinants in COVID-19 Mortality in an US Urban Healthcare System. *medRxiv* 2020: 2020.06.11.20128926.

226. Yehia BR, Winegar A, Fogel R, et al. Association of Race With Mortality Among Patients Hospitalized With Coronavirus Disease 2019 (COVID-19) at 92 US Hospitals. *JAMA Netw Open* 2020; **3**(8): e2018039-e.
227. Zimmerman P, Stroever S, Burton T, et al. Mortality Associated With Intubation and Mechanical Ventilation in Patients with COVID-19. *medRxiv* 2020: 2020.08.13.20174524.
228. Baqui P, Bica I, Marra V, Ercole A, van der Schaar M. Ethnic and regional variations in hospital mortality from COVID-19 in Brazil: a cross-sectional observational study. *The Lancet Global health* 2020; **8**(8): e1018-e26.
229. Bello-Chavolla OY, Bahena-López JP, Antonio-Villa NE, et al. Predicting Mortality Due to SARS-CoV-2: A Mechanistic Score Relating Obesity and Diabetes to COVID-19 Outcomes in Mexico. *J Clin Endocrinol Metab* 2020; **105**(8): dgaa346.
230. Carrillo-Vega MF, Salinas-Escudero G, García-Peña C, Gutiérrez-Robledo LM, Parra-Rodríguez L. Early estimation of the risk factors for hospitalization and mortality by COVID-19 in Mexico. *PloS one* 2020; **15**(9): e0238905.
231. Denova-Gutiérrez E, Lopez-Gatell H, Alomia-Zegarra JL, et al. The Association of Obesity, Type 2 Diabetes, and Hypertension with Severe Coronavirus Disease 2019 on Admission Among Mexican Patients. *Obesity (Silver Spring, Md)* 2020; **28**(10): 1826-32.
232. Escalera-Antezana JP, Lizon-Ferrufino NF, Maldonado-Alanoca A, et al. Risk factors for mortality in patients with Coronavirus Disease 2019 (COVID-19) in Bolivia: An analysis of the first 107 confirmed cases. *Le infezioni in medicina* 2020; **28**(2): 238-42.
233. Giannouchos TV, Sussman RA, Mier JM, Poulas K, Farsalinos K. Characteristics and risk factors for COVID-19 diagnosis and adverse outcomes in Mexico: an analysis of 89,756 laboratory-confirmed COVID-19 cases. *The European respiratory journal* 2020.
234. Murillo-Zamora E, Hernandez-Suarez CM. Survival in adult inpatients with COVID-19. *Public Health* 2020.
235. Soares RdCM, Mattos LR, Raposo LM. Risk Factors for Hospitalization and Mortality due to COVID-19 in Espírito Santo State, Brazil. *Am J Trop Med Hyg* 2020; **103**(3): 1184-90.
236. Solís P, Carreňo H. COVID-19 Fatality and Comorbidity Risk Factors among Diagnosed Patients in Mexico. *MedRxiv* 2020.
237. Sousa GJB, Garces TS, Cestari VRF, Florêncio RS, Moreira TMM, Pereira MLD. Mortality and survival of COVID-19. *Epidemiology and infection* 2020; **148**: e123.
238. Bi Q, Hong C, Meng J, et al. Characterizing clinical progression of COVID-19 among patients in Shenzhen, China: an observational cohort study. *medRxiv* 2020: 2020.04.22.20076190.
239. Chen F, Sun W, Sun S, Li Z, Wang Z, Yu L. Clinical characteristics and risk factors for mortality among inpatients with COVID-19 in Wuhan, China. *Clinical and Translational Medicine* 2020; **10**(2): e40.
240. Chen J, Bai H, Liu J, et al. Distinct Clinical Characteristics and Risk Factors for Mortality in Female Inpatients With Coronavirus Disease 2019 (COVID-19): A Sex-stratified, Large-scale Cohort Study in Wuhan, China. *Clinical Infectious Diseases* 2020.
241. Chen R, Liang W, Jiang M, et al. Risk Factors of Fatal Outcome in Hospitalized Subjects With Coronavirus Disease 2019 From a Nationwide Analysis in China. *Chest* 2020; **158**(1): 97-105.
242. Dai M, Liu D, Liu M, et al. Patients with Cancer Appear More Vulnerable to SARS-CoV-2: A Multicenter Study during the COVID-19 Outbreak. *Cancer discovery* 2020; **10**(6): 783-91.
243. Du R, Liang L, Yang C, et al. Patient Predisposition at Hospital Admission Indirectly Dictates Disease Severity, Clinical Course and Outcomes of COVID-19 Pneumonia Patients in Wuhan, China. *SSRN* 2020.
244. Du RH, Liang LR, Yang CQ, et al. Predictors of Mortality for Patients with COVID-19 Pneumonia Caused by SARS-CoV-2: A Prospective Cohort Study. *The European respiratory journal* 2020.
245. Feng Y, Ling Y, Bai T, et al. COVID-19 with Different Severity: A Multi-center Study of Clinical Features. *American journal of respiratory and critical care medicine* 2020.
246. Gao C, Cai Y, Zhang K, et al. Association of hypertension and antihypertensive treatment with COVID-19 mortality: a retrospective observational study. *European heart journal* 2020; **41**(22): 2058-66.
247. Gu T, Chu Q, Yu Z, et al. History of coronary heart disease increased the mortality rate of patients with COVID-19: a nested case–control study. *BMJ Open* 2020; **10**(9): e038976.
248. Huang S, Wang J, Liu F, et al. COVID-19 patients with hypertension have more severe disease: a multicenter retrospective observational study. *Hypertension Research* 2020; **43**(8): 824-31.
249. Hwang J-M, Kim J-H, Park J-S, Chang MC, Park D. Neurological diseases as mortality predictive factors for patients with COVID-19: a retrospective cohort study. *Neurol Sci* 2020; **41**(9): 2317-24.
250. Kim MK, Jeon JH, Kim SW, et al. The Clinical Characteristics and Outcomes of Patients with Moderate-to-Severe Coronavirus Disease 2019 Infection and Diabetes in Daegu, South Korea. *Diabetes & metabolism journal* 2020; **44**(4): 602-13.
251. Lee H-Y, Ahn J, Kang CK, et al. Association of Angiotensin II Receptor Blockers and Angiotensin-Converting Enzyme Inhibitors on COVID-19-Related Outcome. *SSRN Electronic Journal* 2020.

252. Li J, Guo T, Dong D, et al. Defining heart disease risk for death in COVID-19 infection. *QJM: An International Journal of Medicine* 2020.
253. Li Q, Chen L, Li Q, et al. Cancer increases risk of in-hospital death from COVID-19 in persons <65 years and those not in complete remission. *Leukemia* 2020; **34**(9): 2384-91.
254. Meng Y, Lu W, Guo E, et al. Cancer history is an independent risk factor for mortality in hospitalized COVID-19 patients: a propensity score-matched analysis. *Journal of Hematology & Oncology* 2020; **13**(1): 75.
255. Moon S-S, Lee K, Park J, Yun S, Lee YS, Lee DS. Clinical Characteristics and Mortality Predictors of COVID-19 Patients Hospitalized at Nationally-Designated Treatment Hospitals. *J Korean Med Sci* 2020; **35**(36).
256. Shang Y, Liu T, Wei Y, et al. Scoring systems for predicting mortality for severe patients with COVID-19. *EClinicalMedicine* 2020; **24**: 100426.
257. Shi Q, Zhang X, Jiang F, et al. Clinical Characteristics and Risk Factors for Mortality of COVID-19 Patients With Diabetes in Wuhan, China: A Two-Center, Retrospective Study. *Diabetes care* 2020; **43**(7): 1382-91.
258. Shi S, Qin M, Shen B, et al. Association of Cardiac Injury With Mortality in Hospitalized Patients With COVID-19 in Wuhan, China. *JAMA Cardiology* 2020.
259. Sun H, Ning R, Tao Y, et al. Risk Factors for Mortality in 244 Older Adults With COVID-19 in Wuhan, China: A Retrospective Study. *Journal of the American Geriatrics Society* 2020; **68**(6): E19-e23.
260. Sy KTL, Haw NJL, Uy J. Previous and active tuberculosis increases risk of death and prolongs recovery in patients with COVID-19. *Infectious diseases (London, England)* 2020; **52**(12): 902-7.
261. Tai S, Tang J, Yu B, et al. Association between Cardiovascular Burden and Requirement of Intensive Care among Patients with Mild COVID-19. *Cardiovascular Therapeutics* 2020; **2020**: 9059562.
262. Wang D, Yin Y, Hu C, et al. Clinical course and outcome of 107 patients infected with the novel coronavirus, SARS-CoV-2, discharged from two hospitals in Wuhan, China. *Critical care (London, England)* 2020; **24**(1): 188.
263. Wang K, Zuo P, Liu Y, et al. Clinical and Laboratory Predictors of In-hospital Mortality in Patients With Coronavirus Disease-2019: A Cohort Study in Wuhan, China. *Clinical infectious diseases : an official publication of the Infectious Diseases Society of America* 2020; **71**(16): 2079-88.
264. Wang L, He W, Yu X, et al. Coronavirus disease 2019 in elderly patients: Characteristics and prognostic factors based on 4-week follow-up. *The Journal of infection* 2020; **80**(6): 639-45.
265. Xie J, Covassin N, Fan Z, et al. Association Between Hypoxemia and Mortality in Patients With COVID-19. *Mayo Clinic Proceedings* 2020; **95**(6): 1138-47.
266. Xu J, Yang X, Yang L, et al. Clinical course and predictors of 60-day mortality in 239 critically ill patients with COVID-19: a multicenter retrospective study from Wuhan, China. *Critical care (London, England)* 2020; **24**(1): 394.
267. Yan Y, Yang Y, Wang F, et al. Clinical characteristics and outcomes of patients with severe covid-19 with diabetes. *BMJ Open Diabetes Research & Care* 2020; **8**(1): e001343.
268. Yang Q, Zhou Y, Wang X, et al. Effect of hypertension on outcomes of adult inpatients with COVID-19 in Wuhan, China: a propensity score-matching analysis. *Respir Res* 2020; **21**(1): 172.
269. Yu C, Lei Q, Li W, et al. Clinical Characteristics, Associated Factors, and Predicting COVID-19 Mortality Risk: A Retrospective Study in Wuhan, China. *Am J Prev Med* 2020; **59**(2): 168-75.
270. Zhang F, Xiong Y, Wei Y, et al. Obesity predisposes to the risk of higher mortality in young COVID-19 patients. *Journal of medical virology* 2020.
271. Zhang H, Wang L, Chen Y, et al. Outcomes of novel coronavirus disease 2019 (COVID-19) infection in 107 patients with cancer from Wuhan, China. *Cancer* 2020; **126**.
272. Zhang P, Zhu L, Cai J, et al. Association of Inpatient Use of Angiotensin Converting Enzyme Inhibitors and Angiotensin II Receptor Blockers with Mortality Among Patients With Hypertension Hospitalized With COVID-19. *Circulation research* 2020.
273. Zhang X, Guo W, Hua J, et al. The Incidence, Risk Factors and Clinical Outcomes of Acute Kidney Injury in Critically Ill Patients with COVID-19: A Multicenter Study. *SSRN* 2020.
274. Zhang Y, Cui Y, Shen M, et al. Association of diabetes mellitus with disease severity and prognosis in COVID-19: A retrospective cohort study. *Diabetes research and clinical practice* 2020; **165**: 108227-.
275. Zhao M, Wang M, Zhang J, et al. Comparison of clinical characteristics and outcomes of patients with coronavirus disease 2019 at different ages. *Aging* 2020; **12**(11): 10070-86.
276. Zhou F, Yu T, Du R, et al. Clinical course and risk factors for mortality of adult inpatients with COVID-19 in Wuhan, China: a retrospective cohort study. *The Lancet* 2020; **395**(10229): 1054-62.
277. Zhu L, She Z-G, Cheng X, et al. Association of Blood Glucose Control and Outcomes in Patients with COVID-19 and Pre-existing Type 2 Diabetes. *Cell Metabolism* 2020; **31**(6): 1068-77.e3.
278. Garassino M, Whisenant J, Huang L-C, et al. COVID-19 in patients with thoracic malignancies (TERAVOLT): first results of an international, registry-based, cohort study. *The Lancet Oncology* 2020; **21**.
279. Kuderer N, Choueiri T, Shah D, et al. Clinical impact of COVID-19 on patients with cancer (CCC19): a cohort study. *The Lancet* 2020; **395**.

280. Mato AR, Roeker LE, Lamanna N, et al. Outcomes of COVID-19 in patients with CLL: a multicenter international experience. *Blood* 2020; **136**(10): 1134-43.
281. Atkins JL, Masoli JAH, Delgado J, et al. Preexisting Comorbidities Predicting COVID-19 and Mortality in the UK Biobank Community Cohort. *The Journals of Gerontology: Series A* 2020; **75**(11): 2224-30.
282. Bhaskaran K, Rentsch C, MacKenna B, et al. HIV infection and COVID-19 death: population-based cohort analysis of UK primary care data and linked national death registrations within the OpenSAFELY platform. *medRxiv* 2020: 2020.08.07.20169490.
283. Del Amo J, Polo R, Moreno S, et al. Incidence and Severity of COVID-19 in HIV-Positive Persons Receiving Antiretroviral Therapy : A Cohort Study. *Annals of internal medicine* 2020; **173**(7): 536-41.
284. Hamer M, Gale CR, Kivimäki M, Batty GD. Overweight, obesity, and risk of hospitalization for COVID-19: A community-based cohort study of adults in the United Kingdom. *Proceedings of the National Academy of Sciences* 2020: 202011086.
285. Hamer M, Kivimäki M, Gale CR, Batty GD. Lifestyle risk factors, inflammatory mechanisms, and COVID-19 hospitalization: A community-based cohort study of 387,109 adults in UK. *Brain Behav Immun* 2020; **87**: 184-7.
286. Khawaja AP, Warwick AN, Hysi PG, et al. Associations with covid-19 hospitalisation amongst 406,793 adults: the UK Biobank prospective cohort study. *medRxiv* 2020: 2020.05.06.20092957.
287. Lassale C, Gaye B, Hamer M, Gale CR, Batty GD. Ethnic disparities in hospitalisation for COVID-19 in England: The role of socioeconomic factors, mental health, and inflammatory and pro-inflammatory factors in a community-based cohort study. *Brain Behav Immun* 2020; **88**: 44-9.
288. Patel AP, Paranjpe MD, Kathiresan NP, Rivas MA, Khera AV. Race, Socioeconomic Deprivation, and Hospitalization for COVID-19 in English participants of a National Biobank. *medRxiv* 2020: 2020.04.27.20082107.
289. Williamson EJ, Walker AJ, Bhaskaran K, et al. Factors associated with COVID-19-related death using OpenSAFELY. *Nature* 2020; **584**(7821): 430-6.
290. Gu T, Mack JA, Salvatore M, et al. Characteristics Associated With Racial/Ethnic Disparities in COVID-19 Outcomes in an Academic Health Care System. *JAMA Netw Open* 2020; **3**(10): e2025197-e.
